# Berry-derived gold nanoparticles induce integrated ROS-mediated apoptosis, immune modulation, and transcriptomic remodeling in 4T1 triple-negative cancer cells

**DOI:** 10.1038/s41420-026-03023-z

**Published:** 2026-04-10

**Authors:** Oladapo F. Fagbohun, Adewale O. Oladipo, Chengyu Gao, Babatunde Olawoye, Rachel S. Berry, Jaylah C. Captain, Olive Iragena, Xavier McDougle, Randy J. Harris, Amanda Rollins, Jitcy S. Joseph, Olatomide A. Fadare, Russell Kincaid

**Affiliations:** 1 Department of Biology, Center for Agriculture and Natural Sciences, Wilmington College, Wilmington, OH USA; 2 Department of Mechanical, Bioresources, and Biomedical Engineering, University of South Africa, Florida Science Campus, Roodepoort, South Africa; 3 Mass Spectrometry and Proteomics Facility, The Ohio State University, Columbus, OH USA; 4 Department of Food Science and Technology, Abiola Ajimobi Technical University, Ibadan, Oyo Nigeria; 5 Department of Biochemistry, National Institute for Occupational Health, Division of National Health Laboratory Services, Johannesburg, South Africa; 6 Department of Life and Consumer Sciences, College of Agriculture and Environmental Sciences, University of South Africa, Johannesburg, Republic of South Africa; 7 Organic Chemistry Research Laboratory, Department of Chemistry, Obafemi Awolowo University, Ile-Ife, Osun Nigeria

**Keywords:** Transcriptomics, Breast cancer, Drug delivery, Checkpoint signalling, Cancer metabolism

## Abstract

Comprehensive molecular and phenotypic characterization of tumor models is still needed for a robust understanding of breast cancer mechanisms and therapies. Here, we explore the genome, transcriptome, and proteome of treated and untreated 4T1 triple-negative breast cancer cells to integrate genomic vulnerabilities and mutational profiling with novel treatment-induced delivery, signaling, and apoptotic responses. Nanoencapsulation (AuNPs) of berry-derived polyphenolic compounds was influenced by limited clinical use due to poor stability and bioavailability. Several physicochemical characterizations employed include TEM, FTIR, and targeted UPLC/MS-QQQ assays. We identified significant mutations to breast cancer-related tumor suppressor genes (TP53, BRCA2, BARD1, CDH1, NF1, and CHEK2) and deciphered the functional consequences leveraging the higher throughput Illumina NovaSeq X and NextSeq sequencing and the highly accurate predictive power of AlphaFold. We found ~5,700,000 single-nucleotide variations (SNVs) and 329448 indels, achieving an important upgrade over existing literature data. Multiple sequence alignment with WT mouse and human protein sequences demonstrated that mutations present in 4T1 cells are within highly conserved motifs of key tumor suppressors, emphasizing their relevance to human breast cancer biology. Key findings from differentially expressed gene enrichment analyses (GSEA) revealed positive gene enrichments of DNA repair regulators and TGF-β signaling, while having negative enrichments of cell adhesion, cadherin and MAPK signaling via PI3K/AKT/MAPK/Wnt pathways, potentially influencing apoptosis and immune evasion intrinsic to cancer. Notably, decreased expression of PIK3CG, PALLD, PTPRZ1, and CDH8 and increased expression of SEMA6C, WWOX, NHEJ1, and MAML3 suggested suppression of epithelial-to-mesenchymal transition (EMT) and metastatic potential. Further assessment of immunohistochemical, immunofluorescent, and flow cytometric data revealed that berry-derived nanoparticles are associated with the modulation of oncogenic transcription factors and linked to induced caspase-dependent execution-phase ROS-mediated apoptosis through pPAK1^Thr212^ dephosphorylation, downregulation of pPI3K^p85αγ(Tyr467/199)^/pAKT1^Thr450^/mTOR signaling, and modulation of pJAK3^Tyr785^/STAT3 pathway supporting transcriptomic and transcriptional reprogramming of 4T1 treated cells. Together, our findings uncover a new strategy to capture berry-derived polyphenols required to regulate apoptosis, autophagy, immune response, and metastasis-related gene networks in breast cancer, thereby underscoring the therapeutic potential of functionalized AuNPs as delivery platforms for dietary phytochemicals.

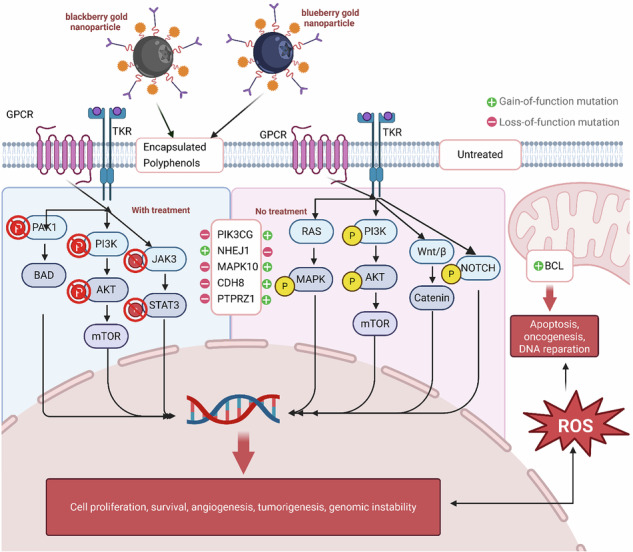

## Introduction

Cancer remains the leading cause of death worldwide, with about 10 million associated deaths in 2020 [1, 2]. Understanding the magnitude of cancer burden and its modifiable risk factors is a molecular catalyst for its effective prevention. Between 2019 and 2025, the total number of cancer deaths globally exceeded 4.45 million [2, 3]. One major type of cancer that remains a complex and prevalent health concern affecting millions worldwide is breast cancer [4, 5]. Despite advances, the functional impact of mutations in breast cancer-associated genes, including BRCA1, BRCA2, PALB2, CHEK2, CDH1, PTEN, STK11, and TP53, remains to be fully elucidated in cancer patient-derived samples. These associated genes play critical roles in DNA repair, genomic stability, cell cycle, apoptosis, and epithelial integrity, leading to disruption in essential processes that increase cancer risks and shape tumor aggressiveness, therapeutic response, and patient outcome [2, 4].

Whether invasive or non-invasive, breast cancer starts when DNA within breast cells becomes damaged or mutated, with the presence of either hormone receptors or human epidermal growth factor 2 (HER2) receptors. Triple-negative (TNBC) and inflammatory breast (IBC) cancers are the most aggressive invasive forms due to their faster growth, infection-mimicking symptoms, and non-existent HER2 and ER receptors. For breast cancer, the regulation of oxidative stress is a crucial factor in its development because excessive reactive oxidative stress (ROS) can lead to DNA damage and DNA replication stress found in activated estrogen, progesterone, and HER2 receptors [6, 7]. There is a link between the signaling pathways controlling tumorigenesis and the regulation of ROS through direct or indirect metabolic mechanisms that influence tumor progression [8–10]. Key pathways include PI3K/AKT, MAPK/ERK, NRF2, PAK1, JAK-STAT, and NFκB, each contributing to cellular proliferation, survival, inflammation, and immune modulation.

Polyphenols are the most prominent natural antioxidants with high health benefits for consumers [11], and berries’ components are laden with high concentrations of these natural health benefits [12–16]. Recent studies have found that polyphenol contributed about 170 mg per 100 g on a dry weight basis in berries [17, 18]. These polyphenols can scavenge free radicals and prevent oxidative stress connected to the development of cancer, especially the inhibitory effects on estrogen receptor-positive (ER + ) breast cancer cells [19–21]. They mainly regulate signaling pathways in cancer metastasis and strengthen the immune system’s ability to identify and eliminate cancerous cells.

Polyphenols are grouped into flavonoids, phenolic acids, stilbenes, and polyphenolic amides and are identified as anticancer agents in recent studies [22]. Specifically, resveratrol was shown to induce p53-dependent apoptosis and reverse DNA methylation and TGF-B1-induced epithelial-mesenchymal transition (EMT) in MDA-MB-231 cells [23, 24]. A study by Nguyen, Lee [25], Sultan, Khalil [26], Kıyga, Şengelen [27], and Srinivasan, Thangavel [28] revealed that quercetin can target PI3K/AKT, STAT, β-catenin, and MAPK signaling pathways leading to the induction of mitochondrial-mediated apoptosis, while kaempferol can activate caspases and increased expression of H2AX and p-ATM in MDA-MB-231 cells. Combined with apigenin, kaempferol has been shown to suppress proliferation, induce cell cycle arrest at G2/M leading to DNA damage correlated with NFκB inhibition and mitochondrial-mediated apoptosis in cancer cells [29, 30]. Genistein, gallic acid, and epigallocatechin gallate are other polyphenols that have been studied to induce G1 cell cycle arrest via the suppression of cell cycle proteins and inhibition of Notch-1/GPR30/AKT/ERK signaling pathways [31–33]. All these polyphenolic compounds can be found in both blueberry and blackberry extracts.

Here, we studied the transcriptomic remodeling in 4T1 cells via RNA-Seq after treatment with berry extracts as well as after berry encapsulation with gold nanoparticles to determine the various changes in the gene-level and transcript-level expression profiles. We highlighted how nanoparticle delivery can modulate key signaling pathways. The concentrations of the different types of polyphenols were measured using a targeted UPLC-QQQ/MS analysis. Moreover, we performed a whole-genome sequence of the 4T1 cells, isolated all the mutated breast cancer-specific genes, and predicted the three-dimensional conformations of the corresponding wild-type and mutant protein isoforms to provide mechanistic insights into how these mutations have contributed to oncogenic signaling and cellular dysregulations. We further expanded the newly discovered 4T1 breast cancer mutational landscape to human breast cancer biology via multiple sequence alignment (MSA). To the best of our knowledge, this is the first report on the application of RNA Seq to measure the expression profiles of blueberry and blackberry AuNPs in 4T1 cells and the relevance of the mutational pattern correlating to human breast cancer therapy.

## Results

### Genomic alterations and variant distribution across the 4T1 tumor genome

**Fig. 1:**
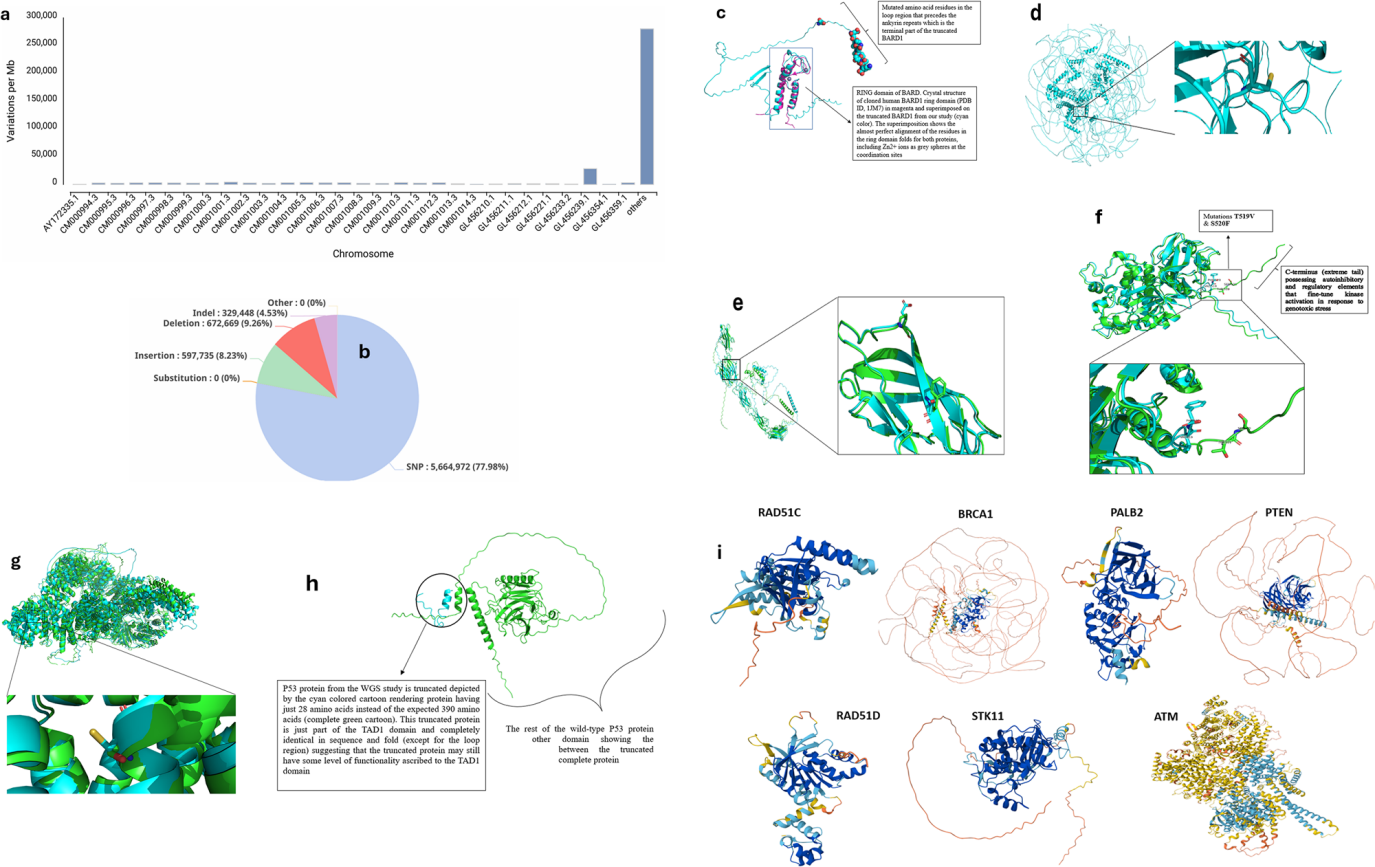
Chromosomal variation density and structural modeling of the mutated breast cancer-predisposition genes in 4T1 cells. **a** Bar graph depicting the density of genomic variations per megabase (Mb) across annotated chromosomes in the 4T1 mouse mammary carcinoma genome. **b** Pie chart illustrating the relative proportions of detected variant types in the 4T1 mouse mammary carcinoma genome. **c** The multiple sequence alignment of the human BARD1 (WT), along with that of mouse (WT), and the truncated protein with the ring domain highlighted in a rectangle at the top of the alignment. The 3D model of the truncated protein from AlphaFold superimposed on the recombinant BARD1 domain (human) shows the similarity in structure of the ring domain of the truncated protein with that of a normal protein. **d** The BRCA2 protein 3D model from AlphaFold with all the associated loop regions and the protein-level mutation G3059C. The Cysteine residue side chain zoomed-in image of the protein. This Cysteine replaces Glycine in the DNA-binding domain (DBD) – specifically within the OBfold region (OB3/DBD core) of BRCA2 (the DBD in mouse BRCCA2 spans roughly residues ~2470–3170). **e** The 3D model of E-cadherin (CDH1). The wild type is the green cartoon, while the mutant protein is the cyan cartoon (both superimposed on each other). The EC3 domain, having two mutations, is covered in the rectangular box. The zoomed out EC3 domain showing the mutant amino acid residues side by side with the wild type residue, P267E and F272S. **f** Superposition of wild-type checkpoint kinase 2 protein from CHEK2 gene (green cartoon) and the truncated mutant protein (cyan colored cartoon), with the mutated residues shown at the truncated end of the protein. Zoomed out graphics of the mutated residues with the distinct residues at the truncated end are more visible. The alignment of the rest of the protein is shown to be very close, indicating that the other parts of the protein are similar and closely aligned. **g** Cartoon representation of the wild-type and mutated neurofibromin, a large cytoplasmic protein that functions primarily as a Ras GTPase-activating protein (Ras-GAP). It converts active Ras-GTP to inactive Ras-GDP, thereby downregulating Ras/MAPK signaling pathways involved in cell proliferation and differentiation. The wild type is green cartoon while the mutant with S331C is cyan cartoon. The region with the mutation is highlighted in black. The expanded region where the mutation occurred, Position 331, falls in the N-terminal region of neurofibromin (well upstream of the GAP-related domain, GRD, which is ~residues 1200–1550). So, this change is unlikely to directly disrupt Ras-GAP catalytic activity, but it *could* affect regulation, stability, or interactions mediated by the N-terminus. Though the residues are structurally similar, the chemistry of the protein at position 331 will change significantly, and this might affect the function, particularly in cancer. **h** Comparison of the truncated TP53 protein (cyan colored) with the complete protein (green colored) shows that the truncated protein is just a fragment of the whole. **i** Predicted 3D structures of seven breast cancer-associated proteins in 4T1 cells. Notably, these genes were not found to be mutated in the 4T1 murine breast cancer model used in this study, suggesting preserved structural integrity and potential functional relevance in the context of therapeutic targeting or resistance mechanisms.

A total of 68,059,294 reads aligned to GCA_000001635_GRCm39 genomic RefSeq, yielding 17140456858 mapped bases and 74,000,000 unique reads (71.02%) from 301 average input read length (Fig. 1a, b). Among 7,044,834 processed variants, 77.98% were SNVs and 4.53% were INDELs, with multi-allelic frequencies of 6,849,701 (2 alleles), 170,276 (3 alleles), and 24,857 (4 alleles). Structural variants totaled 10,700, including 5510 deletions and 4855 insertions. The copy number variant metrics had 563 duplications and 2530 deletions. SNPs/INDELs exhibited downstream, intergenic, and upstream genetic expressions (Table 1, Fig. 1, and Fig. S1).

**Table 1:** Summary of WGS variant counts and effects in 4T1 triple-negative breast cancer samples.

Variant type	Count total	(% total)	Frequency	Effects of the variant	Count total	(% severe)
Total Variant	7,044,834	100	-	Downstream gene variant	16	0.9
SNP	5,664,972	77.98	5,627,248	Intergenic variant	7,264,821	96.97
DEL	672,669	9.26	-	Non-coding transcript exon variant	1	100
INS	597,735	8.23	-	Upstream gene variant	16	12.50
INDEL	329,448	4.53	1,417,586			
Overlapped Genes	1	-	-			
Overlapped Transcripts	24	-	-			
No. of Genotype	14,089,668	-	-			
No. of Heterozygotes	2,329,553	16.53	-			
**Population genetics (ts/tv ratio) and actg content**						
**Change**	**Fraction**			**Sample**	**Reads**	**(% Read)**
AC	0.090					
AG	0.324			A	3,785,036,436	27.853
AT	0.103			C	2,803,481,848	20.639
CG	0.070			T	3,726,890,402	27.459
CT	0.323			G	3,281,708,630	24.049
GT	0.090			GC (%)	-	44.685
Overall Ts/Tv ratio	1.830					

### AlphaFold-based structural modeling of mutated proteins derived from WGS of 4T1 cells

Mutated protein sequences were derived by aligning 4T1 WGS consensus variants with GRCm39/mm39 RefSeq sequences (Fig. S1). Among 13 established breast cancer-predisposition genes (ATM, BARD1, BRCA1, BRCA2, CDH1, CHEK2, NF1, PALB2, PTEN, RAD51C, RAD51D, STK11, TP53), no mutations were detected in BRCA1, ATM, PALB2, PTEN, RAD51C, RAD51D, or STK11. Other genes (PI3KCA, MYC, ERBB2, MAP3K1, EGFR, and FGFR1), identified recently in breast cancer [34, 35], were differentially expressed in our study. BARD1 exhibited a truncating mutation eliminating the ankyrin repeat and tandem BRCT domains (Fig. 1c), which is crucial for DNA damage response and tumor suppression [36, 37]. On the other hand, BRCA2 was found to be mutated at p.Gly3059Cys, located within the OB3 subdomain of the DNA-binding domain (residues 2470–3170; Fig. 1d), representing a missense mutation occurring within the DNA-binding domain of the murine BRCA2 protein, corresponding to a region critical for its interaction with single-stranded DNA (ssDNA) and the DSS1 cofactor [38].

In addition, CDH1 with transcript MN_009864.3 showed two missense mutations, P267E and F272S, both located in the Ca²⁺-binding region of the extracellular cadherin repeats (Fig. 1e), suggesting a loss-of-function phenotype consistent with its established role as a tumor suppressor protein [39, 40]. CHEK2 displayed a C-terminal truncation, loses the last 26 amino acids including the regulatory tail (residues 521-546) (Fig. 1f). Moreover, NF1 gene within the mRNA transcript NM_010897.2 coding for neurofibromin was found mutated at Ser331Cys, in the N-terminal region upstream of the GAP-related domain (Fig. 1g). TP53, a critical tumor suppressor gene on chromosome 17, harbored a frameshift deletion causing a premature stop codon, resulting in a truncated protein at residues 29 – 390 leaving only the residues 1 – 28Lys (Fig. 1h) This finding aligns with Schrörs, Boegel [41], who reported a similar functional impact via a frameshift insertion of “A”. Moreover, we detected mutations to BARD1, BRCA2, CDH1, CHEK2, and NF1 in contrast to Schrörs, Boegel [41], who only detected mutations in TP53 and PIK3CG from breast cancer-predisposition genes. AlphaFold-predicted 3D structures of the non-mutated predisposition genes (ATM, PALB2, PTEN, RAD51C, RAD51D, STK11) are shown in Fig. 1i.

### Protein sequence alignment for identification of conserved and mutant functional motifs

To extend our analysis to a human breast cancer context, we conducted a high‑resolution alignment of wild‑type mouse and human protein sequences against the corresponding mutant variants identified in 4T1 cells, enabling precise identification of residue‑level disruptions within conserved functional motifs (Fig. S1). Multiple sequence alignment of human, wild‑type mouse, and 4T1‑derived mutant BARD1, BRCA2, and CDH1 proteins revealed extensive conservation between human and murine sequences, whereas the mutant variants exhibited residue substitutions and truncations within conserved domains, consistent with loss of canonical tumor suppressor functions. Across BARD1, BRCA2, and CDH1, the alignment shows a strong justification that 4T1 is a relevant model for cancer research. In BARD1, loss of C-terminal regions contained within the BRCT and regulatory motifs is a site for post-translational modifications supporting loss-of-function tumor suppressors found in human breast cancer [42, 43]. The mutation found in CDH1 was found in the conserved extracellular or cytoplasmic region that can weaken cell–cell adhesion, widely studied to promote invasion, metastasis, and epithelial – mesenchymal transition (EMT), central to aggressive breast cancer behavior (Fig. S1) [44].

### Characterization of the berry encapsulated nanoparticles

**Fig. 2:**
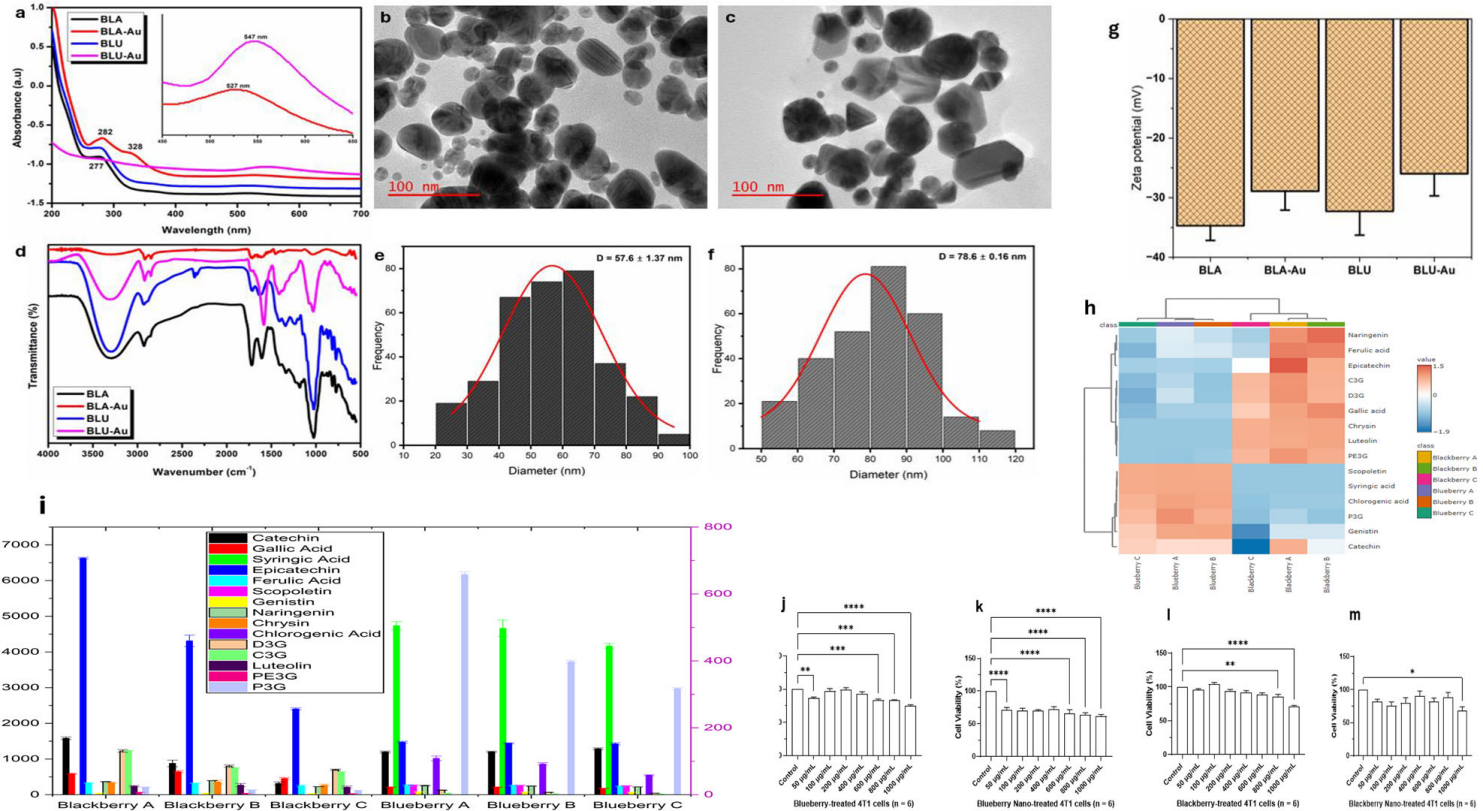
Physicochemical characterization and bioactivity profiling of berries and berry-derived nanoparticles. **a** UV–Vis absorption spectra of blackberry (BLA), blueberry (BLU), and berry nanoparticle formulations (BLU-A and BLA-A). The inset highlights the surface plasmon resonance (SPR) region (500–700 nm), confirming nanoparticle formation and optical properties consistent with gold nanoparticle synthesis. **b** Transmission electron microscopy (TEM) images of blackberry nanoparticles (BLA-A). Spherical morphology and uniform dispersion are observed, with scale bars representing 100 nm from FEI Tecnai G2 Spirit BioTwin Electron Microscope. **c** Transmission electron microscopy (TEM) images of blueberry nanoparticles (BLU-A). Spherical morphology and uniform dispersion are observed, with scale bars representing 100 nm from FEI Tecnai G2 Spirit BioTwin Electron Microscope. Image acquisition was facilitated by a Macrofire digital CCD camera integrated with AMT image capture software version 5.42. **d** Fourier-transform infrared (FTIR) spectra of BLA, BLU, BLU-A, and BLA-A samples. Peaks corresponding to hydroxyl, carbonyl, and aromatic functional groups indicate successful capping and stabilization of nanoparticles by phytochemicals. **e**, **f** Size distribution histograms of BLA-A (e) and BLU-Au (f) nanoparticles. Gaussian fitting reveals average diameters of 82.4 ± 1.37 nm and 78.6 ± 1.65 nm, respectively, indicating size reduction upon gold incorporation. **g** Zeta potential measurements of BLA, BLA-A, BLU, and BLU-A nanoparticles. Surface charge values reflect colloidal stability, with BLU-A exhibiting the highest negative potential, suggesting enhanced dispersion and reduced aggregation. **h** Heatmap of targeted phytochemical profiling in blueberry (BLU) and blackberry (BLA) extracts. Quantification was performed using the Agilent 6495D triple quadrupole LC/MS system operating in dMRM mode. Relative abundances of key phytochemicals, including anthocyanins, flavonols, phenolic acids, and stilbenes, are color-coded across two sample groups in triplicate. Red indicates higher abundance, while blue indicates lower abundance. **i** Extracted ion chromatogram of the total 15 targeted compounds and Kynurenic acid – d5 internal standard on UPLC/MS-QQQ. **j**–**m** MTT-based cell viability assays of 4T1 breast cancer cells treated with berry-derived nanoparticle formulations. Panels represent independent biological replicates (*n* = 6) for each berry type. Bar charts display the distribution and density of cell viability percentages across treatment groups, with statistical significance indicated by asterisks (*p < 0.05, **p < 0.01, ***p < 0.001, ****p < 0.0001, ns = not significant). All treatments were normalized to untreated controls. Blueberry and blackberry formulations exhibited differential cytotoxicity profiles, with select samples inducing significant viability reduction, suggesting phytochemical-dependent anti-proliferative effects. The sample size (*n*) is the number of biological replicates and is specified where applicable on each figure, and for all unspecified replicates, *n* = 3.

Figure 2a–d showed the absorption spectra of the BLA and BLU extracts as well as their corresponding Au nanoparticles. The Au plasmon peaks at 527 and 547 nm for BLA-A and BLU-A nanoparticles, respectively, indicating the formation of Au nanoparticles (Fig. S2). The calculated loading efficiencies for blackberry (BLA-A) and blueberry (BLU-A) nanoparticles were 61.2 ± 1.21% and 67.3 ± 2.15%, respectively. Additional peaks at 282 nm and 328 nm from the BLA-A and a peak around 275 nm from the BLU-A were observed, thus demonstrating a strong interaction between the phytochemicals from the BLA and BLU extracts and the formed nanoparticles. The TEM images showed that the BLU-A nanoparticles were mostly spherical in shape (Fig. 2b), while the BLA-A nanoparticles were polydisperse with a mixture of spheres, hexagons, triangles, and cubes (Fig. 2c). The mixed shapes observed from the TEM images of the BLU-A could be responsible for shifting the plasmon peak to 547 nm. Also, the average particle diameter calculated from TEM images for BLA-A (Fig. 2e) and BLU-A (Fig. 2f) was 57.6 ± 1.37 nm and 78.6 ± 0.16 nm, respectively (Fig. S2).

A significant interaction between the BLA and BLU extracts and the prepared BLA-A and BLU-A nanoparticles was observed from the infrared spectra data (Fig. 2d). Noticeable peaks of BLA were observed at 3303, 2925, 2928, 1720, 1609, and 1026 cm^-1^ as well as BLU peaks at 3308, 2934, 2882, 1709, 1626, and 1029 cm^-1^ corresponding to the -OH groups, -C-H groups, C=O or C=N groups and -C-O groups, respectively. Typical bands from the BLA and BLU extracts were found on both the BLA-A and BLU-A nanoparticles spectra, particularly the O-H (alcohols/phenols), C-H (alkanes), C=O (carbonyl), and a strong peak C-O (ether/ester/alcohol), indicating a direct participation of these functional groups in the effective reduction and stabilization of the nanoparticles. The colloidal stability of the nanoparticles was measured using zeta potential, and as shown in Fig. 2g, the average values for BLA-A and BLU-A were determined to be −28.9 ± 1.71 mV and −25.9 ± 1.41 mV compared to -34.7 ± 1.24 mV and -32.3 ± 2.11 mV for BLA and BLU, respectively.

### Quantification of the phytochemicals in blueberry and blackberry extracts

Using the Agilent 6495D triple quadrupole UPLC/MS system in dMRM mode, we achieved high-sensitivity quantification of targeted phytochemicals in berry extracts. Calibration curves for all analytes exhibited excellent linearity (*R*^2^ ≥ 0.991) with a limit of detection ranging from 0.05 to 2 ng/mL (Fig. S2). Additionally, the Hierarchical clustering revealed distinct phytochemical signatures between blueberry and blackberry extracts (Fig. 2h). The bar chart and extracted ion chromatogram (Fig. 2i, S2) displayed well-resolved peaks corresponding to each of the 15 target polyphenols. BLU had higher levels of scopoletin, syringic acid, chlorogenic acid, P3G, genistin, and catechin, and BLA showed higher levels of the remaining nine polyphenols, including epicatechin (2.4–6.6 mg/kg) and gallic acid (0.5–0.6 mg/kg). Extracted ion chromatograms displayed baseline-separated peaks for all 15 polyphenols (Fig. 2i), with C3G, catechin, chlorogenic acid, D3G, epicatechin, and PE3G eluting at 0.9–2.5 min, and gallic acid, P3G, syringic acid, ferulic acid, scopoletin, and genistin at 2.5–4.5 min. Luteolin, naringenin, and chrysin showed sharp, symmetrical peaks. All analytes had Gaussian shapes and S/N > 10:1 (Fig. S2).

### Cytotoxicity profiling

MTT assay results indicated that the BLU (Fig. 2j) and BLA (Fig. 2k) showed a significant reduction in 4T1 cell viability in a dose-dependent manner (*n* = 6), with BLU showing a larger median decrease than BLA. BLU-A and BLA-A further reduced viability compared to crude extracts (Fig. 2l, m), achieving the lowest median values (*p* < 0.05– < 0.0001). Vehicle control showed no direct MTT reduction. From the dose-response curve, we unified the 200 µg/mL concentration across treatments to allow us to capture mechanistic responses.

### Transcriptomic remodeling of the differentially expressed breast cancer genes (DEGs)

**Fig. 3:**
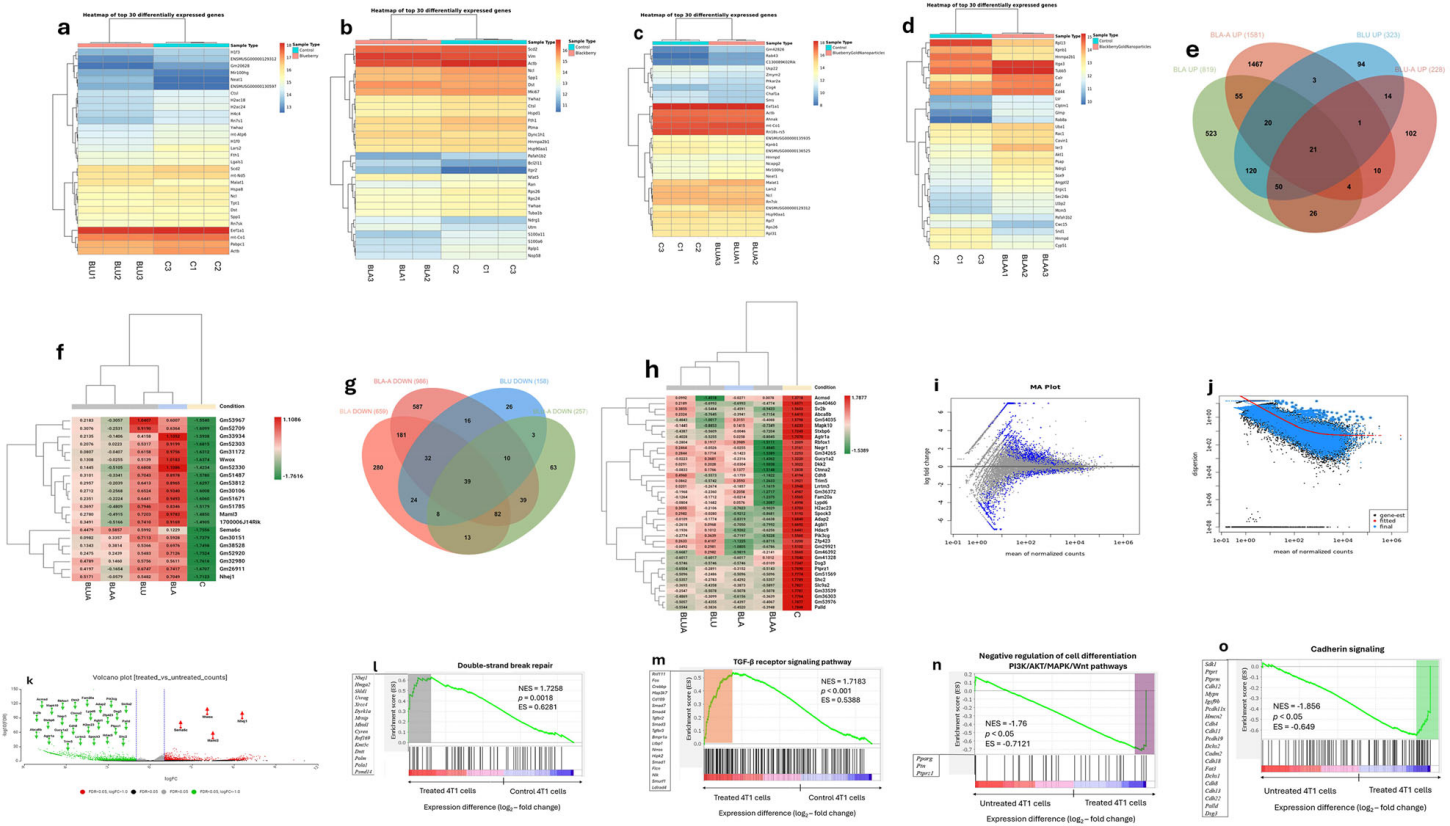
Gene set enrichment (GSEA) transcriptomic profiles of modulated oncogenic, apoptotic, and DNA repair transcription factors in berry-derived treatments in 4T1 triple-negative breast cancer cells. **a**, **b** Heatmaps of the top 30 differentially expressed genes across control and berry extract treatment in 4T1 cells. Samples are hierarchically clustered based on expression profiles. A shows control (C) vs blueberry (BLU), while B shows control (C) vs blackberry (BLA). Red indicates upregulation, blue indicates downregulation. These profiles highlight distinct transcriptional responses to natural products from blackberry and blueberry treatments. **c**, **d** Heatmaps of the top 30 differentially expressed genes across control and berry-derived nanoparticle treatment in 4T1 cells. Samples are hierarchically clustered based on expression profiles. C shows control (C) vs blueberry nanoparticle (BLUA), while D shows control (C) vs blackberry nanoparticle (BLAA). Red indicates upregulation, blue indicates downregulation. Hierarchical clustering reveals consistent transcriptional shifts in genes, suggesting antioxidant and differentiation-related effects of treatment. **e** Venn diagram of upregulated genes across four berry-related treatment conditions: Blackberry nanoparticle formulations have 1581 upregulated genes, while blackberry extract has 819 upregulated genes. Meanwhile, blueberry nanoparticle formulations had 323 upregulated genes, while blueberry extract had 228 upregulated genes. Overlapping regions indicate shared transcriptional responses, with 21 genes common to all groups. **f** Heatmap of upregulated genes across the treatment groups. Gene expression is scaled from low (green) to high (red), with hierarchical clustering revealing distinct berry-specific transcriptional signatures. **g** Venn diagram of downregulated genes across four conditions: Blackberry nanoparticle formulations have 986 downregulated genes while blackberry extract has 659 downregulated genes. Meanwhile, blueberry nanoparticle formulations had 257 downregulated genes, while blueberry extract had 158 downregulated genes. Thirty-nine genes are commonly downregulated across all treatments, suggesting conserved suppressive effects. **h** Heatmap of downregulated genes across the treatment groups. Expression values range from −1.7877 to +1.5389, with clustering highlighting treatment-specific repression patterns. **i** MA plot showing log₂ fold change versus mean normalized counts for all genes. Genes above and below the *y* = 0 line represent upregulated and downregulated transcripts, respectively. **j** Dispersion plot from DESeq2 modeling. Black dots represent gene-wise dispersion estimates, blue dots show fitted values, and the red curve indicates the final dispersion trend used for differential expression analysis. **k** Volcano plot of treated versus untreated 4T1 cells. Genes with significant differential expression (FDR < 0.05) are color-coded: red (upregulated), green (downregulated), black (moderate), and gray (non-significant). **l** GSEA plot for double-strand break repair (GO:0006303). Enrichment scores indicate significant upregulation of homologous recombination repair genes, suggesting enhanced DNA repair capacity in berry-treated cells. **m** GSEA plot for TGF-β receptor signaling pathway (GO:0007179). Upregulation of Smad3, Tgfbr2, and Zeb2 suggests activation of EMT and immune modulation. **n** GSEA plot for negative regulation of cell differentiation via PI3K/AKT/MAPK/Wnt pathways. NES = −1.76, ES = −0.7121. Downregulation of differentiation suppressors indicates enhanced plasticity and stem-like features. **o** GSEA plot for cadherin signaling (GO:0007156). NES = −1.856, ES = −0.649. Repression of cadherin-mediated adhesion genes supports EMT induction and metastatic potential. The sample size (*n*) is the number of biological replicates and is specified where applicable on each figure, and for all unspecified replicates, *n* = 3.

To decipher the treatment-specific reprogramming of 4T1 triple-negative breast cancer cells, we performed bulk RNA transcriptomic analyses of untreated and treated cells. RNA-seq of untreated and treated 4T1 cells yielded 68 million reads per sample with adapter bases. Mapping to GCA_000001635_GRCm39 achieved a greater than 92% mapping rate (Fig. S3). BLA-treated cells showed 82.486% uniquely mapped reads (16 million splices), while BLU-treated cells had 82.964% uniquely mapped reads (20 million splices). BLA-A-treated cells yielded 129,957,416 total reads with 31 million splices, and BLU-A-treated cells had 101,383,412 reads with 24 million splices. Heat maps showing the number of DEGs (Fig. 3, S3) revealed induced stronger transcriptional remodeling and changes in 4T1 breast cancer cells. Figure 3a, b, c, and d showed the top 30 DEGs in 4T1 cells compared with untreated cells.

Of 40,402 RefSeq features, 18,243 passed filtering, with 2793 genes being differentially expressed (FDR < 0.05) where 1348 genes were upregulated (logFC>1), and 1445 genes were downregulated (logFC<1). Specifically, BLA-treated cells had 1348 DEGs (768 upregulated, 580 downregulated compared to untreated 4T1 cells (Fig. 3b). On the other hand, BLU had 595 DEGs (366 upregulated, 229 downregulated) (Fig. 3a). BLA-A induced 2,373 DEGs (1581 upregulated, 792 downregulated), while BLU-A altered 397 DEGs (193 upregulated, 204 downregulated) (Fig. 3c, d). Across treatments, 21 genes were commonly upregulated and 39 downregulated (Fig. 3e, g). Heatmaps showed the strongest remodeling with blackberry-Au NPs (Fig. 3f, h). MA plots (Fig. 3i) and dispersion analysis (Fig. 3j) confirmed transcriptomic stability with subsets of significant changes, mostly in high-abundance genes. Volcano plot (Fig. 3k) revealed predominant gene up- and downregulations. Interestingly, one of the highlights of our study is the downregulation of PIK3CG (Fig. 3h), a critical isoform of PI3K that encodes the catalytic subunit p110γ of class IB phosphoinositide-3-kinase. It is usually upregulated in cancer [45–48], and its downregulation leads to the suppression of PI3K/AKT/mTOR, MAPK/ERK, and chemokine signaling. This correlates with reduced immune infiltration and better response to checkpoint inhibitors in cancer. These findings provide a foundation for downstream functional annotation and pathway enrichment analysis to elucidate the biological roles of differentially expressed genes in the 4T1 breast cancer model.

### Modulation of oncogenic transcription factors by berry extracts

Moreover, the genes related to tumor suppressor RBI proteins, including GM53967, NHEJ1, WWOX, MAML3, and SEMA6C genes, were found to be upregulated in all treatments (Fig. 3f, k, S3). Oncogenic factors such as AGTR1, HDAC9, PIK3CG, and DSG3 were found differentially downregulated, while MAPK10, RBFOX1, DKK2, and CTNNA2 were found differentially downregulated in all the treatments (Fig. 3h, S3). In addition, genes such as NEAT1, MIR100HG, CTSL, LGALS1, SPP1, TPT1, NCL, ACTB, AHNAK, YWHZ, FTH1, and MT-ATP6 (Fig. 3a, c) were differentially expressed in BLU and BLU-A treated cells. While some of the notable genes were also found differentially expressed in BLA-BLA-A treated cells (Fig. 3b, d), some other genes were found specific to BLA treatments, and they are VIM, MK67, BCL2L11, AXL, CD44, AKT1, RAC1, and SOX9 associated in apoptotic and oncogenic transcriptions while some are widely used to assess tumor growth rate [49–54]. Interestingly, activation of the oncogenic transcription factors is associated with control of cell cycle, apoptosis, migration, and cell differentiation, while the gain-of-function modulation of these genes is linked to the presence of natural products (Fig. 2h, i, S3) [55].

### GSEA transcriptomic (GSEA) profiling revealed apoptotic DNA repair and TGF-β upregulation in cancer

We next employed transcriptomic profiling strategies to elucidate the components of the programmed cell death and DNA repair networks. The current study identified the network of gene sets from the DEGs after berry treatments (Fig. 3a–h, S3) by carrying out GSEA. Firstly, gene sets of DNA damage signal and repair pathways were activated after treatments (Fig. 3l, S3). Key genes are XRCC4, MBD1, HMGA2, DYRK1B, and MRNIP. All treatments also modulate transforming growth factor (TGF-β) receptor signaling pathway by statistically significant positive enrichment gene set (Fig. 3m, S3) and key genes are MAP3K7, SMAD4, FOS, SMURF1, NRROS, TGFBR2, CD109, RNF111, CREBBP, SMAD7, TGFBR3, FLCN, and NLK. In contrast, treated cells were statistically enriched with genes for apoptotic signaling pathway involvement via negative regulation of cell differentiation with downregulation of PPARγ, PTN, and PTPR21 (Fig. 3n, S3). Treated cells had zero crossing at rank 12,821, indicating the gene sets were downregulated in the experimental condition and suggesting suppression of adhesion-related transcripts via cadherin signaling. key genes are CDH12, PCDH11X, CDH4, CDH11, PCDH19, CDH18, CDH8, CDH13, CDH22, PALLD, and DSG3 (Fig. 3o, S3).

### Berry extracts and nanoparticles induce ROS-mediated apoptosis via early apoptosis but not necrosis

**Fig. 4:**
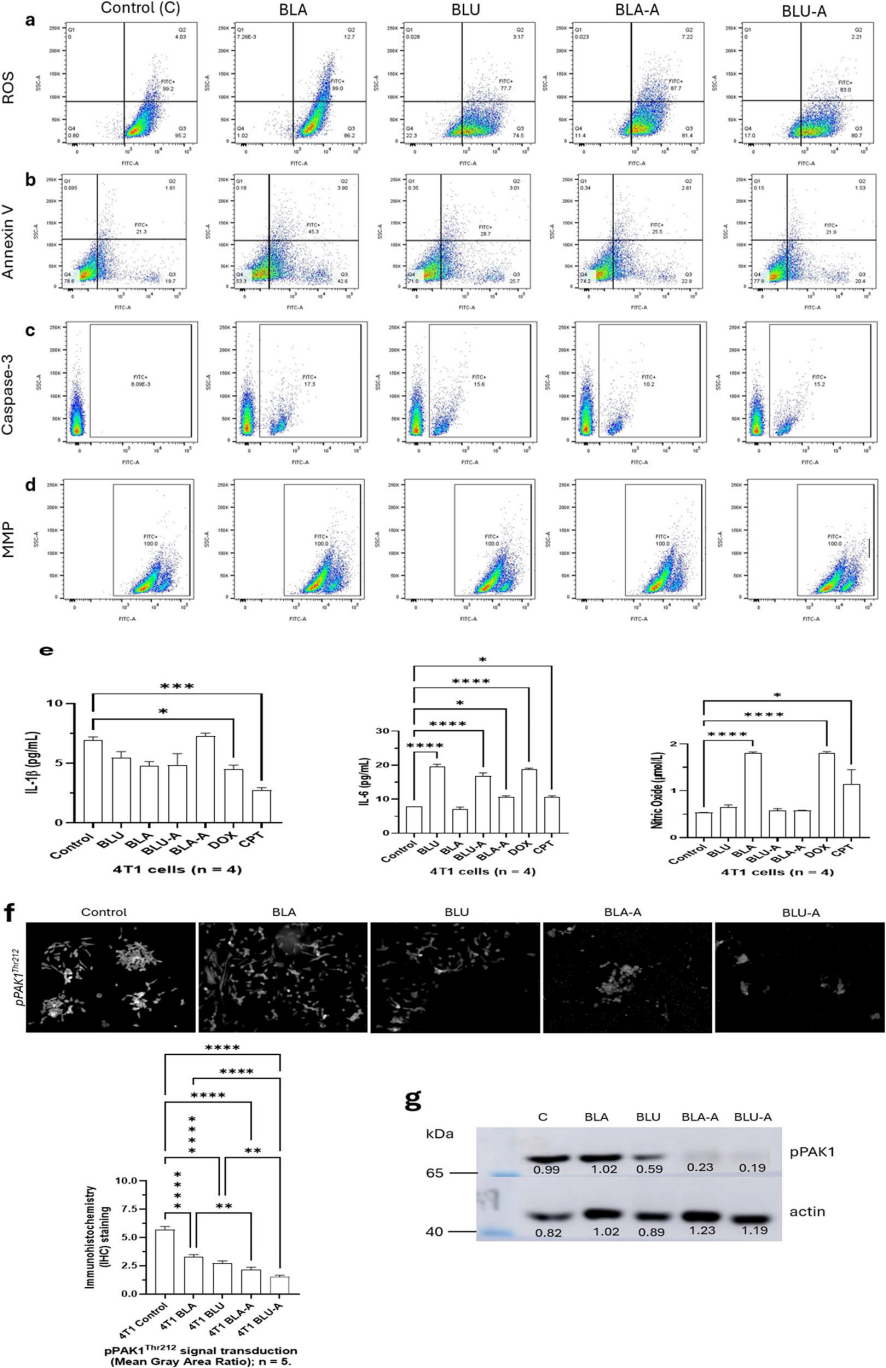
Expression of oxidative stress, apoptosis, mitochondrial integrity, and inflammatory signaling in 4T1 breast cancer cells. **a** Flow cytometric dot plots showing intracellular reactive oxygen species (ROS) detected by CellROX^®^ ROS detection reagents. FITC-A represents CellROX, while SSC-A represents SYTOX. Stained samples were analyzed on a BD LSRFortessa X-20 cell analyzer flow cytometer. **b** Flow cytometric dot plots showing Annexin V and 7-AAD dual staining detected by CF^®^ 488 A Annexin V and 7-AAD Apoptosis Kit. CF^®^488 A Annexin V spectral was detected at Ex/Em 490/515 nm (FITC channel) while 7-AAD was detected at Ex/Em 548/648 nm (with DNA)(PE-Cy^®^ or PerCP channel). Stained samples were analyzed on a BD LSRFortessa X-20 cell analyzer flow cytometer. **c** Flow cytometric dot plots showing caspase-3/7 activity detected by NucView^®^ 488 Caspase-3Assay Kit for live cells. NucView^®^ 488 Caspase-3 spectral detected at Ex/Em 500/530 nm (after cleavage with DNA). Stained samples were analyzed on a BD LSRFortessa X-20 cell analyzer flow cytometer. **d** Flow cytometric dot plots showing mitochondrial membrane potential detected by JC-1 Mitochondrial Membrane Potential Detection Kit. The cytoplasm was detected at Ex/Em 510/527 nm (green) while the polarized mitochondrial was detected at Ex/Em 585/590 nm (red). Stained samples were analyzed on a BD LSRFortessa X-20 cell analyzer flow cytometer. **e** Bar charts quantifying IL-6 (pg/mL), IL-1β (pg/mL), and nitric oxide (µmol/L) activities. Cytokine levels were determined by Invitrogen^TM^ Mouse ELISA kits (Cat: 5017218 and 5017219) while nitric oxide level was detected by Elabscience^®^ NO Colorimetric Assay Kit (E-BC-K035-M). **f** Representative immunohistochemical images and quantification of pPAK1^Thr212^ signal intensity. pPAK1^Thr212^ was upregulated in 4T1 breast cancer cells, while 4T1 cells treated with BLU, BLA, BLA-A, and BLA-A had downregulation of pPAK1^Thr212^. Scale Bar is 20 µm. **g** Immunoblots showing the protein expression of pPAK1^Thr212^ probed with β-actin for loading control. The data shown is representative of duplicate independent experiments. The sample size (*n*) is the number of biological replicates and is specified where applicable on each figure, and for all unspecified replicates, n = 3.

Flow cytometry analysis of CellROX and SYTOX staining revealed baseline oxidative stress in untreated cells, with 95.2% in Q3 (high ROS, stressed) and 0.80% in Q4 (low ROS, healthy (Fig. 4a, S4). Treatment with BLA and BLA-A (200 µg/mL) reduced ROS formation at 86.2% and 81.4%, respectively. Meanwhile, BLU and BLU-A extract (200 µg/mL) induced strong and sustained ROS reduction resulting in viable cells at 22.3 and 17.0% (Fig. 4a), respectively. Annexin V (CF®488 A)/7-AAD staining showed early apoptosis (Annexin V⁺/7-AAD⁻) in 42.6% and 22.9% of BLA and BLA-A extracts, respectively. However, BLU and BLU-A were consistent with less prominent early apoptotic response at 25.7 and 20.4%, respectively (Fig. 4b, S4), suggesting sustained apoptotic signaling with minimal necrosis and higher cell viability, validating phosphatidylserine externalization in TGF-β signaling as seen in Fig. 3m [56–58]. Although overall ROS levels were reduced at the measured time point, this pattern is consistent with a transient early ROS surge that initiates apoptotic signaling before subsequently declining. The decreased ROS detected by CellROX, therefore, likely reflects a later phase of the apoptotic program or activation of endogenous antioxidant defenses following treatment.

### Sustained apoptotic signaling in berry-related treatments of 4T1 cancer cells revealed execution-phase apoptosis and intact mitochondrial membrane potential (ΔΨm)

Moreover, flow cytometry assessment of caspase-3/7 activity after 24 h treatment with berry extracts at 200 µg/mL revealed BLA had the strongest apoptotic induction (Fig. 4c, S4) with 17.3% cells, while all other treatments had elevated caspase-3. No significant SSC-A shifts were observed, indicating apoptosis without necrosis or pyroptosis. Mitochondrial membrane potential analysis indicated a gated region consistently capturing cells with high FITC fluorescence with preserved MMP in all treated cells, reflecting minimal depolarization (Fig. 4d, S4). On the other hand, BLU extract showed the strongest ROS reduction, with preserved MMP and BLU-A extracts showed reduced oxidative stress with apoptotic commitment.

### Berry-derived nanoparticle formulations induce a modulated immune response

ELISA analysis (Fig. 4e) revealed the levels of IL1β, IL-6, and nitric oxide (NO). We found out that camptothecin abrogated IL-6 induction, suggesting immunosuppressive interference. There was no significant difference between the levels of IL-1β BLA, BLU, BLU-A, and BLA-A treated cells when compared to the untreated control, although there was a marked reduction in the levels of IL-1β in BLU, BLA, and BLU-A, while BLA-A had a marked increase in IL-1β level. Moreover, the levels of IL-6 were found to be reduced in BLA and BLA-A treated cells, in contrast to the significantly increased levels found in BLU and BLU-A. Nitric oxide levels were markedly increased by BLA (****p < 0.0001), doxorubicin (****p < 0.0001), and camptothecin (*p < 0.05), while BLU, BLU-A and doxorubicin-treated cells showed no statistical differences.

### Berry nanoparticle formulations are possible PAK1 inhibitors through PAK1 dephosphorylation

Immunohistochemical analysis showed BLA and BLU extracts are associated with reduced pPAK1 (p21-activated kinase 1) intensity, which is usually upregulated in most cancer cases [59], with further significant decreases in BLA-A and BLU-A treatments (Fig. 4f, g), influencing MAPK/ERK and NFκB pathways. To further understand the role of PAK1 in berry-induced apoptosis, we investigated the phosphorylation site of PAK1 via Western blot. The nanoparticles were revealed to consistently interfere with the phosphorylation of PAK1 at pPAK1^Thr212^, suggesting that encapsulation of plant-derived phytochemicals is associated with conformational changes in protein structures of PAK1/ERK1/2 kinase complex, which have been long postulated to induce apoptosis in targeted cancer therapy [60, 61].

### Berry-derived phytochemicals and nanoencapsulates are associated with changes in PI3K/AKT/mTOR, JAK/STAT, and JNK/NRF2/ERK1/2 complexes

**Fig. 5:**
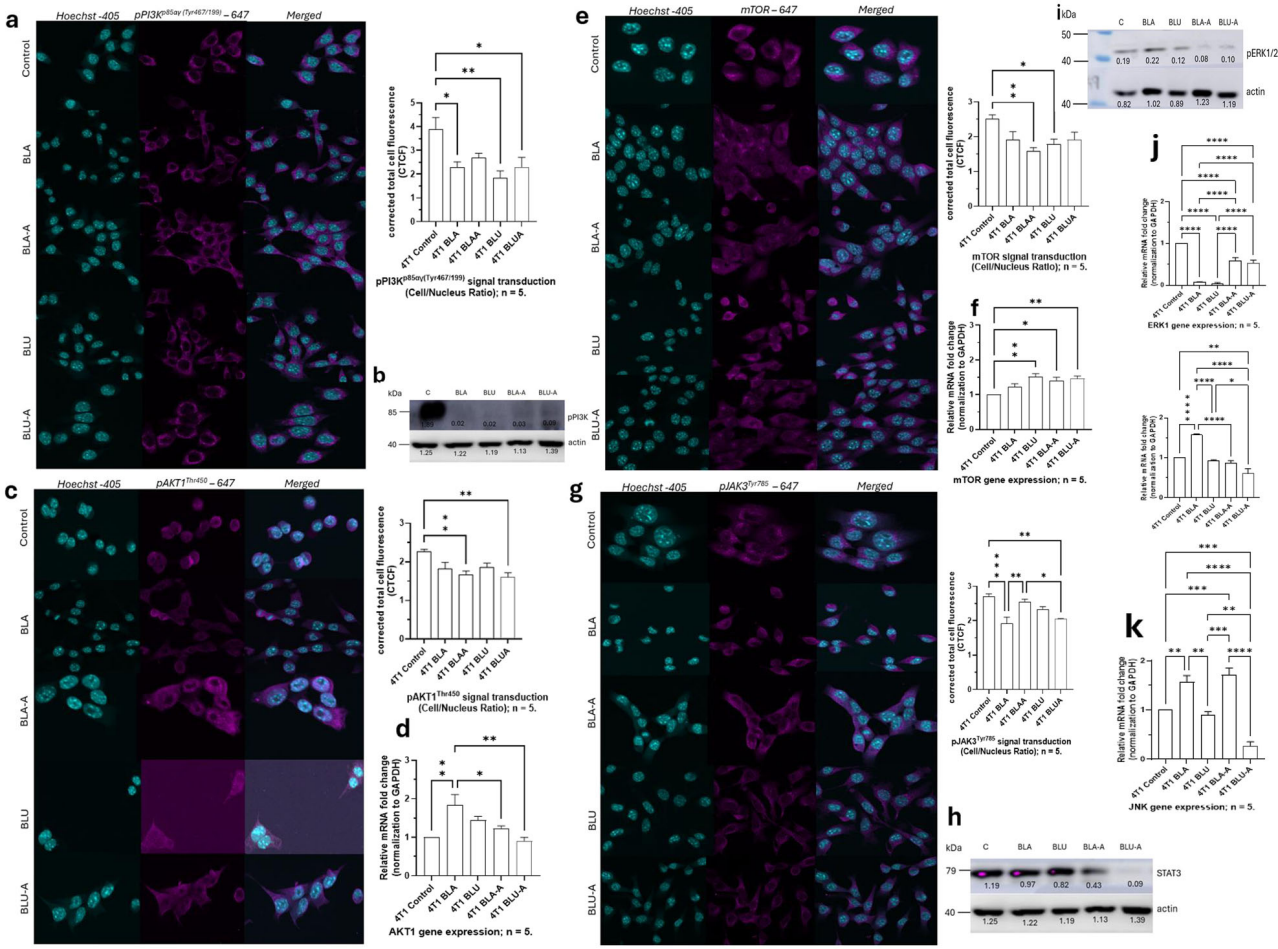
Modulation of dual PI3K/AKT/mTOR and JAK/STAT/MAPK signaling cascades in 4T1 breast cancer cells. **a** Immunofluorescence detection of pPI3K^p85αγ (Tyr467/199)^ (Alexa Fluor 647) with Hoechst nuclear counterstain. All treatments markedly decrease PI3K phosphorylation compared to control. Scale Bar is 20 µm. **b** Immunoblots validating the reduction of pPI3K at Tyr 467/199 and actin as loading control. **c** Immunofluorescence analysis of pAKT1^Thr450^ signal intensity confirming downstream PI3K/AKT pathway activation. Scale Bar is 20 µm. **d** Gene expression confirming downregulation of the AKT1 pathway. **e** Immunofluorescence analysis of mTOR signal intensity confirms downstream PI3K/AKT/mTOR signal downregulation. Scale Bar is 20 µm. **f** Gene expression for the relative mRNA fold change for the mTOR pathway. **g** Immunofluorescence detection of pJAK3^Tyr785^ signal. BLA and BLU-A treatments exhibited JAK3 dephosphorylation, implicating cytokine-driven survival signaling. Scale Bar is 20 µm. **h** Immunoblots validating the reduced STAT3 pathway. **i** Western blot analysis of the phosphorylated mitogen-activated protein kinase (MAPK1/MAPK3). Reduction of the expression in the nanoparticle formulations suggests reduced inflammatory signaling. **j** Relative mRNA fold change for the expression of the ERK1/ERK2 gene. **k** Relative mRNA fold change for the expression of JNK1/2/3 gene, validating the reduced inflammatory pathway. The sample size (*n*) is the number of biological replicates and is specified where applicable on each figure, and for all unspecified replicates, *n* = 3.

Here, we studied the complex kinase network regulated by our berry and berry nanoparticles using corrected total cell fluorescence (CTCF) from the mean fluorescent intensity (MFI) in a region of interest (ROI). In our study, we discovered that phosphorylation of PI3K was correspondingly inhibited at the regulatory subunit, pPI3K^p85αγ (Tyr467/199)^. All our treatments (Fig. 5a, b) were associated with suppression of PI3K signaling. Furthermore, it was revealed that BLA-A and BLU-A treated cells were associated with reduced expression of AKT1 (serine/threonine protein kinase) by dephosphorylation at the pAKT1^Thr450^ subunit (Fig. 5c, d). Interestingly, mTOR2 encodes mTOR, RICTOR, mLST8, PROTOR1/2, DEPTOR, and mSIN1 and interacts with PDK1 to activate AKT via phosphorylation. Our study revealed that mTOR signaling was slightly lowered in all the treatments (Fig. 5e, f).

We next probed the expression of the JAK/STAT pathway. It was discovered that BLU-A was associated with suppression of the JAK/STAT pathway (Fig. 5g, h). Other treatments corresponded with reduced pJAK3/STAT3 activity, suggestive of better response or reduced metastatic risk. Our results indicate that suppression of JAK3/STAT3 signaling may enhance the efficacy of PI3K/AKT/mTOR inhibitors, immune checkpoint blockade, and oxidative stress-inducing agents. These results are further validated in the downregulation of other known signaling pathways usually disrupted in cancer (Fig. 5i).

## Discussion

**Fig. 6:**
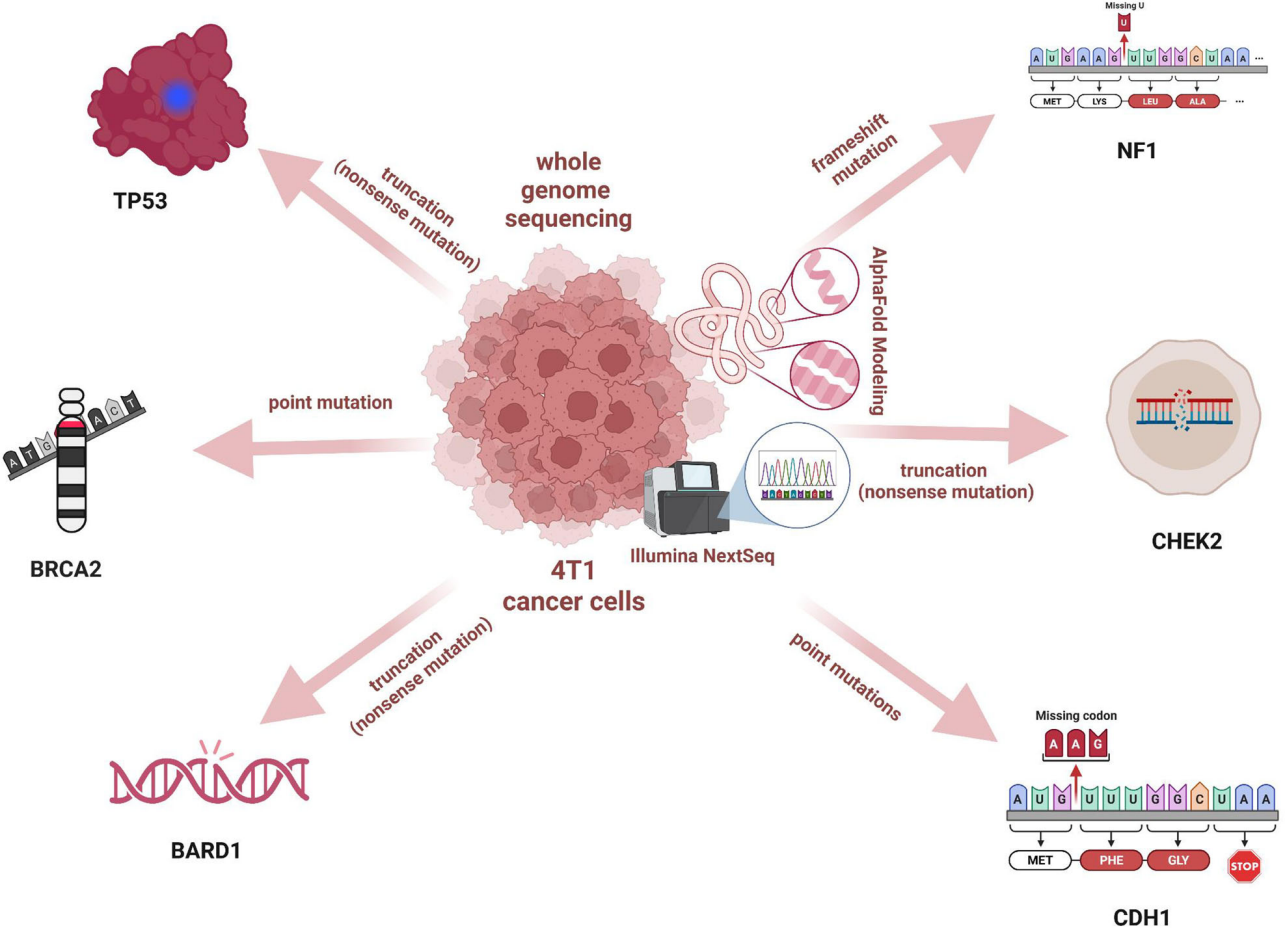
High-impact somatic mutations in tumor suppressor and DNA repair genes identified in 4T1 triple-negative breast cancer cells via whole genome sequencing and AlphaFold structural modeling. Key genetic alterations were discovered in 4T1 murine breast cancer cells using whole genome sequencing (WGS) on the Illumina NextSeq 1000 platform. Six genes (TP53, NF1, CHEK2, CDH1, BARD1, and BRCA2) were found to harbor distinct mutation types, each contributing to the aggressive phenotype of this triple-negative breast cancer model. Mutations were annotated using Sequencher software and structurally modeled via AlphaFold to assess functional impact. By integrating AlphaFold predictions with multi-omics data, we have contextualized structural integrity within broader functional landscapes, enhancing our understanding of mutated tumor suppressors in aggressive breast cancer models. This mutation map was generated to establish the baseline genomic vulnerabilities of 4T1 cells prior to treatment with berry-derived phytochemical formulations. These mutations informed the selection of key signaling nodes (PI3K, AKT, mTOR, JAK3, ERK1/2) for downstream immunofluorescence and immunoblot analyses.

Despite advancements in high-throughput sequencing technologies, our understanding of the molecular mechanisms underlying breast cancer pathogenesis is still evolving. Here, we leveraged newer models of Illumina NextSeq and NovaSeq X instruments to interrogate mutations and DEGs in the 4T1 breast cancer model across 13 predisposition genes. Our results have identified 5 new mutations (BARD1, BRCA2, CDH1, CHEK2, and NF1) in addition to TP53 and PIK3CG genes that were previously identified [41]. Furthermore, we have employed AlphaFold structural modeling to decipher the mutational pattern of these breast cancer-predisposition genes. Truncations in TP53 and BARD1 disrupted key domains essential for DNA damage response, while BRCA2 G3059C substitution perturbed the OB3 subdomain critical for RAD51 filament stabilization [36, 38, 62]. CDH1 mutations (P267E, F272S) within the Ca²⁺-binding region impaired epithelial cohesion and promoted epithelial-to-mesenchymal transition [40]. CHEK2 truncation eliminated its regulatory tail, possibly altering nuclear targeting and post-translational modifications [63]. NF1 S331C introduced a redox-reactive cysteine upstream of the GAP domain, affecting Ras/MAPK signaling and neurofibromin stability [64, 65] as summarized in Fig. 6. As shown, by comparing wild‑type mouse and human protein sequences with the mutant forms present in 4T1 cells, we were able to place the observed alterations within a broader evolutionary and clinical framework, highlighting conserved motifs that may hold relevance for human breast cancer biology. These conserved, truncating, and missense mutations in BARD1, BRCA2, and CDH1 likely contribute to homologous recombination deficiency and impaired epithelial adhesion in 4T1 cells, thereby shaping both their aggressive phenotype and their susceptibility to phytochemical‑loaded gold nanoparticles. The cross‑species conservation of the affected regions supports the translational relevance of our findings to human breast cancers with analogous genomic lesions. This comparative alignment underscores the potential translational value of these alterations, suggesting that conserved residue‑level disruptions may represent mechanistic nodes worth exploring in future human TNBC models. Since these mutations correspond to genomic vulnerabilities via disruption to DNA damage response, we essentially employed a strategy that can be associated with modulating and remodeling ROS-mediated apoptosis and immune functions [41, 66].

To explore these vulnerabilities, we developed gold nanoparticle formulations encapsulated with blackberry (BLA) and blueberry (BLU) phytochemicals. Targeted UPLC/MS-QQQ profiling confirmed the presence of flavonoids and phenolic acids, in which the polyphenols have been reported to be associated with induced G1 cell cycle arrest via the suppression of cell cycle proteins and inhibition of Notch-1/GPR30/AKT/ERK signaling pathways, leading to the restoration of the expression of the levels of p27, PTEN, and ERα proteins [30, 67]. TEM further revealed spherical (BLUA) and polydisperse (BLAA) shape suggesting nanoparticle stability and surface capping. Next, we employed GSEA and integrated RNA-seq to probe DEGs. The downregulation of PIK3CG (Fig. 3h), a critical isoform of PI3K that encodes the catalytic subunit p110γ of class IB phosphoinositide-3-kinase and is usually upregulated in cancer, is particularly striking since it is an important candidate for the activation of PI3K/AKT/mTOR/ERK signaling, a hallmark pathway in cancer proliferation and progression [45]. Other gene set enrichments in our study include apoptotic and DNA repair pathways, highlighting activation of NHEJ1 and TGF-β signaling.

Functionally, nanoparticle formulations are associated with induced early apoptosis without mitochondrial collapse as revealed by preserved membrane potential, annexin V positivity, and caspase-3 activation. The observed reduction in ROS does not contradict a ROS‑mediated apoptotic mechanism, as many polyphenol‑based treatments induce a short‑lived oxidative burst that triggers downstream caspase activation before ROS levels normalize or fall. This temporal dynamic, widely reported in the literature, suggests that our measurements captured a post‑initiation phase in which antioxidant pathways or mitochondrial dysfunction contribute to the lower ROS signal [68, 69]. Immunomodulatory analyses revealed that nanoparticle formulations correspond with reduced IL-6 and IL1β cytokine responses as evidenced by downregulation of pPAK1^Thr212^ and pPI3K^p85αγ (Tyr467/199)^/pAKT1^Thr450^/mTOR signaling. Collectively, our integrative approach demonstrated synergy with intrinsic tumor suppressor defects to induce apoptosis, suppress oncogenic signaling, and remodel transcriptional landscapes in breast cancer cells. While our genomic, transcriptomic, and biochemical data are strongly associated with signaling pathway modulation, precise mechanistic and molecular hierarchies warrant more investigations that would further strengthen our narrative results. Despite the strength of our multi‑omics approach, this study is limited by its reliance on in vitro models, which cannot fully recapitulate the complexity of the tumor microenvironment or immune interactions in vivo. We are currently exploring the syngeneic mouse models to validate the observed transcriptional and signaling changes, assess nanoparticle biodistribution, and examine treatment‑induced modulation of the immune landscape.

## Conclusion

Our study shed more light on the mutational pattern of the triple-negative breast cancer model widely used in cancer research. 4T1 cancer cells harbor critical mutations in tumor suppressor genes that compromise DNA repair, adhesion, and checkpoint control. By integrating AlphaFold modeling with genomic, immunohistochemical, flow cytometric, immunofluorescence, and transcriptional profiling, we propose that berry-derived nano-formulations correspond to early apoptosis to modulate oncogenic immune suppression in a promising therapeutic avenue (PI3K/AKT/mTOR, JAK/STAT, and PAK1) for targeting aggressive breast cancers alongside conventional therapies.

## Materials and methods

### Chemicals

Catechin (APB0713), gallic acid (APB0434), syringic acid (APB1040), (-)-epicatechin (AOB0404), ferulic acid (APB0096), scopoletin (APB1024), genistin (AOB5041), luteolin (AOB5669), naringenin (APB0677), chrysin (APB0115), delphinidin-3-glucoside chloride (D3G) (ATN1564), chlorogenic acid (APB0405), pelargonidin-3-glucoside chloride (PE3G) (CFN92134), cyanidin-3-glucoside chloride (C3G) (AOB6740), and peonidin-3-glucoside chloride (P3G) (CFN92046) were obtained from Aobious, Inc. (Gloucester, MA, USA). All other chemicals were of HPLC analytical grade.

### Blueberry and blackberry extracts preparation

Blueberry (Cat:2024072031) and blackberry (Cat:2024067746) extracts were obtained from OliveNation LLC (Avon, MA, USA). The freeze-dried fruit powder was extracted in 70% ethanol and concentrated using Hei-VAP advantage rotary evaporator coupled with Heidolph Rotachill (Heidolph Instruments GmbH, Germany) as described by Fagbohun, Hui [70]. The extracts were kept at −20 °C for further analysis.

### Cell preparation and culture

4T1 cancer cells (ATCC CRL-2539^TM^) were purchased from the American Type Culture Collection (ATCC, Manassas, VA, USA) and cultured immediately according to instructions. Before the study, the cells were cultured into T75 flask in ATCC-formulated RPMI-1640 medium (ATCC 30-2001) containing 10% heat-inactivated fetal bovine serum (FBS) (FBS002-HI) (Neuromics Inc., Edna, MN, USA) and 1% penicillin-streptomycin solution (ATCC, Manassas, VA, USA) and incubated at 37 °C with 5% CO_2_. As the cells reached about 85% confluency, the flasks were washed with phosphate-buffered saline (PBS) and trypsinized with 0.25% (w/v) Trypsin EDTA (ATCC, Manassas, VA, USA) until the cells were detached. The cells were seeded in 6-well plates at 1.0 × 10^5^ cells/mL for the entire study.

### Cell viability study

For the 3-(4,5-dimethylthiazol-2-yl)-2,5-diphenyl tetrazolium bromide (MTT) assay, the cells were seeded into 96-well plates. After cell culture, the cells were treated with berry extracts. A total of 10 µL of 5 mg/mL MTT (MedChemExpress, Monmouth, NJ, USA; Cat: HY-15924) was added to the well and incubated for 4 h at 37 °C. The plate was then shaken gently 30 s on a plate shaker at room temperature, and 100 µL DMSO was added to each well. The cell viability was measured after the absorbance was monitored at 562 nm. For all other analyses, we designated blackberry-treated cells as BLA, blueberry-treated cells as BLU, blackberry-nanoparticle-treated cells as BLA-A, and blueberry-nanoparticle-treated cells as BLU-A.

### Green gold nanoparticle synthesis

Blueberry (BLU-A) and blackberry (BLA-A) encapsulated gold nanoparticles were produced by mixing 25 mL aqueous solutions of the berry extracts with 250 mL of 1 mM HAuCl_4_·3H_2_O in a fixed volume ratio (1:10 v/v %) and temperature (25 and 50°C ). The reaction mixture was observed for a color change within the first 10 min and stirred for 1 h under a magnetic stirrer at 85 °C. The heat was turned off, and the solution was stirred for another 5 min until a stable color was observed. After allowing the solutions to cool, they were collected in a flacon tube and centrifuged at 4400 rpm for 30 min. The synthesized AuNPs were washed with Milli-Q water twice to remove excess Au^3+^ solution and uncapped phytochemicals by means of centrifugation at 10,000 × *g* for 20 min. The AuNP pellets were dried at 50 °C overnight to obtain powdered nanoparticles and stored at 4 °C until further analyses.

### UV-Visible absorption, hydrodynamic diameter, and zeta potential measurements

The optical properties of the biosynthesized berry AuNPs were interrogated via ultraviolet-visible (UV-Vis) spectrophotometry using the NanoDrop™ 2000c spectrophotometer (Thermo Electron Scientific Instruments LLC, Madison, WI, USA). Spectral acquisition was performed across the 300–800 nm wavelength domain to elucidate the nanoparticles’ surface plasmon resonance (SPR) signature, which is the hallmark of colloidal gold systems, indicative of particle size, shape, and aggregation state. Also, the loading efficiencies were measured at 277 nm and calculated as the difference between the absorbances between the extract and supernatants extrapolated to percentages. To further delineate the physicochemical attributes of the colloidal dispersion, dynamic light scattering (DLS) and electrophoretic mobility analyses were conducted using the Malvern Zetasizer™ Nano ZS particle size analyzer (Malvern Instruments Ltd, Worcestershire, UK). These measurements yielded critical parameters including the hydrodynamic diameter, zeta potential, and polydispersity index (PDI), which collectively inform on the colloidal stability, surface charge distribution, and size heterogeneity of the nanoparticulate ensemble. The zeta potential serves as a proxy for electrostatic repulsion forces governing particle dispersion, while the PDI reflects the uniformity of the nanoparticle population.

### Fourier transform infrared spectroscopy (FTIR)

Spectral data were acquired using Fourier Transform Infrared Spectroscopy (FTIR) on a Thermo Fisher Nicolet iS5 spectrometer (Thermo Electron Scientific Instruments LLC, Madison, WI, USA), equipped with Attenuated Total Reflectance (ATR) iD5 diamond crystal, Deuterated Triglycine Sulfate (DTGS) KBr detector, and ZnSe lens for better transmission range across the mid-infrared spectrum, making it ideal for focusing infrared light with the FTIR machine. About 200 mg of finely ground samples were placed in the ATR diamond crystal sampling plate, and data were acquired in the 4000–500 cm^-1^ range at room temperature and 32 scans as described by Osorio, Haughey [71] and Traciak, Sobczak [72].

### Transmission electron microscopy (TEM)

To investigate the morphological characteristics of the encapsulated blueberry and blackberry extracts at the nanoscale, samples were deposited onto glow-discharged copper TEM grids pre-coated with a formvar-carbon support film (Cat: FCF200-Cu-50; Electron Microscopy Sciences, Hatfield, PA, USA). The glow discharge treatment was employed to enhance hydrophilicity and promote uniform sample adhesion across the grid surface. Following air-drying under ambient conditions, the grids were subjected to high-resolution imaging using a FEI Tecnai G2 Spirit BioTwin transmission electron microscope (ThermoFisher Scientific, Waltham, MA, USA), operated at an accelerating voltage of 80 kV to optimize contrast and resolution for biological and organic nanostructures. Image acquisition was facilitated by a Macrofire digital CCD camera (Optronics Inc., Chelmsford, MA, USA), integrated with AMT image capture software version 5.42 (Advanced Microscopy Techniques, Woburn, MA, USA), enabling precise visualization and documentation of nanoparticle morphology, encapsulation integrity, and dispersion patterns.

### Targeted UPLC\MS-QQQ analysis

Targeted metabolomics was carried out on the Agilent1290 infinity II Binary UPLC system coupled to 6495D triple quadrupole mass spectrometer with Agilent Jet Stream electrospray ionization source (AJS)(Agilent Technologies Inc., Santa Clara, CA, USA). The separation was optimized and performed on Accucore C18 2.1 × 100 mm; 2.6 µm column with binary solvents of (A) 0.1% formic acid in nanopure water and (B) 0.1% formic acid in 95% acetonitrile (acetonitrile/nanopure water; 95:5/*v*:*v*). The LC flow rate was 0.4 mL/min with gradient was as follows: 0-0.5 min, hold at 5% B; 0.5-5.5 min, 5% to 60% B; 5.5-6 min, 60% to 80% B; 6-8 min, 80% to 100% B; 8-10 min, hold at 100% B; 10.01-12 min, equilibration at 5% B. Column temperature was set at 45 °C. The injection volume was set at 5 µL, and the autosampler temperature was set at 10 °C. The mass spectrometer parameters were optimized as follows: capillary voltage of 3000 V in positive ESI (electro spray ionization) mode and 1500 V in negative mode, nozzle voltage of 1500 V in positive ESI mode and 500 V in negative ESI mode, gas temperature of 290 °C, gas flow of 14 L/min, nebulizer of 30 psi, sheath gas temperature of 350 °C, sheath gas flow of 12 L/min. Dynamic multiple reaction monitoring (dMRM) was performed to quantify a total of 15 polyphenolic compounds, comprising flavonoids, phenolic acids, coumarin, and anthocyanins from blueberry and blackberry extract using kynurenic acid-d5 as the internal standard. The dMRM transitions of each compound and corresponding collision energy were optimized (Fig. S2). The samples were analyzed in triplicate following the reverse-phase LC-MS/MS method reported in the Agilent application note 5994-2156EN. The quantitative data analysis was processed using MassHunter quantitative analysis B12.2. The concentrations of 15 compounds are provided in supplementary files (Fig. S2). The analysis was carried out at the Ohio State University Campus Chemical Instrument Center (CCIC-MSP).

### RNA extraction, cDNA synthesis, and RT-qPCR

Cells were harvested after treatment at the indicated time point in Trizol reagent. Total RNA was extracted via Norgen Biotek^TM^ total RNA purification kit (Alkali Scientific, Fort Lauderdale, FL, USA). DNase treatment and RNA cleanup were performed on the extracted RNA samples to generate high RNA quality with RNA integrity number between 9.70 – 10. RNA samples were evaluated with Fluorometry by DeNovix QFX Fluorometer using the Qubit RNA BR Assay and the 2100 Bioanalyzer using the Agilent RNA 6000 Nano Kit. The primers used in the PCR experiment are highlighted in the supplementary methods. RT-qPCR was carried out using the PowerUp^TM^ SYBR^TM^ Green master mix (ThermoFisher Scientific, Waltham, MA, USA; Cat: A25743) on the Applied Biosystems StepOne Plus PCR system (ThermoFisher Scientific, Waltham, MA, USA) in triplicate. The gene expression was normalized to endogenous controls, GAPDH. Fold change in expression was calculated by dividing the normalized expression in the test sample by that of the control, which was set to 1.0 unless otherwise noted. Genes with a fold change ≥ 1.0 were considered upregulated, while those with a fold change ≤ 1.0 were considered downregulated. For genes with expression values ≤ 0.5 relative to the mock control, the degree of downregulation was expressed as the inverse (1/expression ratio) to clearly represent the magnitude of change, while the expression values≥1.5 relative to the mock control represent a higher degree of upregulation.

### Total transcriptome analysis (RNA Sequencing)

RNA sequencing was carried out by the Genomics Shared Resource of the Ohio State University Comprehensive Cancer Center (The James, OSUCCC, Columbus, OH, USA). mRNA libraries were prepared using the following reagents from QIASeq FastSelect RNA Lib HMR and QIA UX96 Index UDI-B (96) 331825 Kits (Qiagen Inc., Santa Clarita, CA, USA; Cat: 334235) with primers N6-T RT and ODT-RT. The quality of the libraries was evaluated with fluorometry by DeNovix QFX fluorometer (DeNovix Inc., Wilmington, DE, USA; S/N: S-04083) and with 2100 Bioanalyzer (Agilent Technologies Inc., Santa Clara, CA, USA; S/N: DE72903137). A total of 100 ng total RNA was used for the sequencing. Ribosomal RNA was removed by enzymatic digestion and then fragmented for 3 min. The libraries were sequenced with Illumina NovaSeq X plus 10B (Illumina Inc., San Diego, CA, USA), with sequence coverage ranging between PF clusters 35 million to 50 million paired reads. Raw data were converted into FASTQ format, demultiplexed, and filtered for reads passing QC filters. Adapter and low-quality sequences were trimmed, and only sequences with Q30 or higher quality were used. RNA Seq FASTQ files were processed and mapped to the reference. RNA sequencing reads were aligned to the published *Mus musculus* reference genome. Mapping rates and coverage statistics of the samples were reported.

### RNA seq data processing

Raw RNA sequencing expression data were subjected to rigorous pre-processing to ensure analytical robustness. Transcripts with low expression levels (CPM) < 1 were filtered out to eliminate noise and improve statistical power. The remaining data were normalized using the DESeq function within the DESeq2 R package (v. 1.36.0), with normalization parameters adjusted to account for biological treatment conditions associated with pathogen infection. A generalized linear model (GLM) design matrix was constructed to incorporate experimental conditions and relevant biological covariates. This modeling framework enabled the differentiation of true treatment effects from potential confounding variables. To further stabilize variance across the dynamic range of mean expression values, a variance stabilizing transformation (VST) was applied using the VST function in DESeq2, following recommended guidelines. Differential gene expression analysis was performed using DESeq2, NOISeq, and edgeR (OmicsBox app, BioBam Bioinformatics S.L., Valencia, 46006, Spain; v3.4.6), with statistical significance assessed via the Benjamini–Hochberg procedure to control the false discovery rate (FDR). Genes exhibiting an adjusted *p*-value < 0.05 and absolute log2 fold change (|log2FC | ) > 1 were classified as differentially expressed. The normalized expression matrix comprising pre-processed and variance-stabilized read as the input for principal component analysis (PCA) to visualize sample clustering and variance structure. All graphical outputs, including box plots, volcano plots, and hierarchical clustering heatmaps, were generated using R software.

### Gene set enrichment analysis (GSEA)

Gene Set Enrichment Analysis (GSEA) was performed to identify biologically relevant pathways and molecular signatures associated with differential gene expression profiles. Normalized expression data were ranked based on signal-to-noise ratio or log₂ fold-change between experimental conditions. GSEA was conducted using the OmicsBox app (BioBam Bioinformatics S.L., Valencia, 46006, Spain; v3.4.6). Enrichment scores (ES) were calculated using 1000 gene set permutations to assess statistical significance. The normalized enrichment score (NES), false discovery rate (FDR q-value), and nominal *p*-value were used to determine pathway significance. Gene sets with FDR q-value < 0.25 and NES > 1.5 were considered significantly enriched. Leading-edge subsets were extracted to identify core genes driving enrichment. Visualization of enriched pathways was performed using Enrichment Map and custom scripts in the R programming app (R Foundation, Vienna, Austria; v4.2.2) for heatmaps and ridge plots.

### Whole-genome next-generation sequencing (WG-NGS)

Genomic DNA was extracted from 4T1 triple-negative breast cancer cells using QuickExtract DNA extraction solution. The cell lysate was heated in a Veriti thermal cycler (Thermo Fisher Scientific, Waltham, MA, USA) at 65 °C for 10 min followed by 98 °C for 5 min. Genomic DNA was QC’ed, used to construct the NGS library (Illumina Phix control library), and was subjected to whole-genome next-generation sequencing (WG-NGS) on Illumina NextSeq 1000 (Illumina, Inc., San Diego, CA, USA; S/N: VL00482) at ACGT Inc. (ACGT DNA Sequencing Services, Wheeling, IL, USA, ACGT ID: 855012, Illumina NextSeq Run ID: 241030_VL00482_40_AACJNGWM5). The Illumina NextSeq 1000 was equipped with P1 reagents and XLEAP-SBS chemistry on a 600-cycle run (2 ×300 bp) to ensure an accurate quality score of ≥85% of bases higher than Q30.

### DNA seq data processing

Raw DNA high-quality sequencing reads were subjected wo quality control to assess base quality scores, GC content, and adapter contamination. They were further trimmed to remove low-quality bases and sequencing adapters, followed by alignment to the reference genome (GCA_000001635.9/27_GRCm39). The genomic structural, small nucleotide, and copy number variants from the Illumina WGS raw reads were assessed, integrated, and evaluated by two analytic pipelines: Illumina Dragen BaseSpace Germline and Somatic apps (Illumina Inc., San Diego, CA, USA; Dragen BaseSpace v4.4) [73–75] and OmicsBox app (BioBam Bioinformatics S.L., Valencia, 46006, Spain; OmicsBox v3.4.6) [76]. The resulting SAM files were sorted to BAM format, where duplicate reads were Picard-marked. Valling calling was conducted, followed by joint genotyping across the reads in VCF format. Variants were filtered based on depth, quality-by-depth, and strand bias metrics. The variants were then annotated to assign functional consequences, including gene context, coding impact, and known clinical significance, as well as predicted pathogenicity scores. Somatic variant calling was performed to infer copy number alterations, and where applicable, downstream visualization and integrative analysis were conducted. To better understand and characterize the variations, we performed DNA alignment using BWA 0.7.17, SAMtools 1.10, and QualiMap 2.2.1 [77–79], variant calling and annotations using Picard 3.2.0, SAMtools 1.15.1, and BCFtools 1.15.1 [80].

### Prediction of protein structures from mutated breast cancer genes using AlphaFold

The mutated genes implicated in breast cancer were determined according to published literature [81–85]. The amino acid sequences of the expressed proteins were retrieved from the WGS data using Sequencher 5.4.5 software (Gene Codes Corporation, Ann Arbor, MI, 48108, USA) by translating the resulting open reading frames (ORFs) in FASTA format and submitted to the AlphaFold server for structure prediction [86]. The resulting models were obtained in Crystallographic Information File (CIF) format and subsequently imported into PyMOL (PyMOL by Schrodinger, Portland, OR, USA) for three-dimensional visualization. Cartoon-style renderings of the predicted structures were generated and ray-traced within PyMOL to produce high-resolution images for inclusion in the manuscript. Where structural comparisons were required, protein superpositions were performed using PyMOL’s aligned module to facilitate accurate spatial alignment and comparative analysis.

### Multiple sequence alignment (MSA) of wild‑type, mutant, and human protein sequences

Protein sequence alignments were performed using Clustal Pro (Clustal Omega, EMBL-BI, Hinxton, UK), which applies a progressive alignment algorithm optimized for large and divergent datasets [87]. Human reference protein sequences were obtained from UniProt, and wild‑type mouse sequences were retrieved from the NCBI RefSeq database. Mutant protein sequences identified in 4T1 cells were translated from the corresponding coding variants and included in the alignment. All sequences were aligned using default gap‑opening and gap‑extension penalties, with the Gonnet substitution matrix applied to score residue similarity. The resulting multiple sequence alignments were visualized and manually inspected to identify conserved domains, residue‑level substitutions, and truncation events. Regions of divergence or premature termination in the 4T1‑derived mutant proteins were annotated relative to conserved motifs shared between mouse and human orthologs.

### Protein extraction and Western blotting

Cell lysates were lysed in RIPA buffer containing PMSF and Na_3_VO_4_ (Elabscience, Houston, TX, USA) and supplemented with protease (Epigentek Group Inc., Farmingdale, NY, USA; R-1101-1) and phosphatase inhibitor cocktail (Sigma-Aldrich, Saint Louis, MO, USA; 0000215517). Cell lysates were vortexed and left on ice for 30 min. The supernatant was obtained by centrifugation for 10 min at 13,500 × *g* at 4 °C. Lysate protein concentration was determined using the bicinchoninic acid (BCA) method using the BCA standards as described in the manufacturer’s protocol (Elabscience, Houston, TX, USA), and all lysates were diluted into equal concentration before Western blot assay. After the Western blot procedure, the membrane was blocked with 5% non-fat dry skimmed milk in TBST and blotted with primary antibodies overnight. After incubation with secondary antibodies, the membranes were imaged on an Odyssey FC imager (LiCOR Biosciences, Lincoln, NE, USA). The primary antibodies used can be found in the supplementary tables. The experiments were conducted in triplicates while for some immunoblot experiments involving multiple overlapping targets, the membranes were stripped, rinsed, blocked, and re-probed.

### Immunohistochemistry

The cultured cells in a chamber were fixed with 4% paraformaldehyde (PFA) in phosphate-buffered saline (PBS) for 60min at room temperature and washed gently with PBS three times. Then, the cells were permeabilized with 0.1% TX-100 in PBS for 5 min, followed by two washes with PBS. The cells were blocked with 0.5% BSA/PBS or normal serum for 10 min, followed by adding primary antibody, incubating overnight at 4 °C. The primary antibody used was phospho-PAK1^Thr212^ antibody (Elabscience Bioinovation Inc., Texas, USA; Cat: E-AB-21179). Afterward, the fixed cells were gently washed with PBS three times, then incubated with biotinylated-secondary antibody with 2% normal horse serum at room temperature for 60 min. After washing, the secondary antibody with PBS three times, ABC solution (Avidin Biotin Complex, Vector Labs) was added and incubated for 60 min at room temperature. After three times washing with PBS, the signal was developed using NovaRed solution (Vector Labs). Color development was observed under the microscope. When red enough, the cells were placed in PBS to stop the reaction. After a thorough wash with water, the cells were counterstained with Hematoxylin for 15 s. The cells were then dehydrated briefly with graded alcohols from 70%–100% and transferred to the chamber slides into xylenes and sealed the slides with mounting media and glass coverslips in a fume hood.

### Immunofluorescence

4T1 breast cancer cells were cultured and treated with berry extracts and encapsulated berry AuNPs prior to immunofluorescence analysis. Cells were washed with 1× PBS, fixed in 4% paraformaldehyde for 15 min, and permeabilized with 0.1% Triton X-100 for 15 min. After sequential PBS and PBB (PBS + BSA) washes, cells were blocked with 5% normal goat serum (ThermoFisher Scientific, Waltham, MA, USA) in PBB for 45 min. Primary antibodies: phospho-JAK3^Tyr785^ Cat: E-AB-21215, phospho-AKT1^Thr450^ Cat: E-AB-20804, mTOR Cat: E-AB-15789, phospho-P13K^p85αγ (Tyr467/199)^ Cat: E-AB-20966 (Elabscience Bioinovation Inc., Texas, USA) were applied for ≥60 min, followed by five PBB washes and incubation with Alexa Fluor™ 647-conjugated goat anti-rabbit IgG (Thermo Fisher; Cat#: 2994031) for 60 min. Nuclei were stained with Hoechst 33258 (Sigma-Aldrich, St. Louis, MO, USA) for 30 s, and cells were mounted using ProLong™ Gold antifade medium. Samples were stored at 4 °C in the dark until imaging. Confocal microscopy was performed at the CMIF, The Ohio State University Comprehensive Cancer Center, using an Olympus FV3000 point-scanning confocal system integrated with an Olympus IX83 inverted microscope (Model: IX3-LHLEDC) and a 20 × 0.85 NA oil immersion objective (PlanAOPN*SC, NA 1.40) with IMMOIL-F30CC oil (refractive index 1.518 at 23 °C). Imaging parameters included 2× zoom, 3× integration, 512 × 512 resolution, and 4.0 μs/pixel scan speed. Spectral separation used a DM405/488/561/640 dichroic mirror in VBP mode with a 217 μm confocal aperture. Fluorescence was detected using cooled GaAsP PMTs (SD1, HSD3/4). Hoechst was excited at 405 nm (0.3% power), Alexa Fluor™ 594 at 594 nm (5% power), and Alexa Fluor™ 647 at 640 nm (0.5–1.1% power), with emissions captured in cyan, green, and red channels, respectively. Five representative fields per coverslip were imaged, and final images were saved in .oir format and exported as 24-bit RGB TIFFs.

### Flow cytometry

The cultured cells were analyzed for reactive oxygen species (ROS) formation, caspase 3/7 activity, annexin V apoptosis assay, and loss of mitochondrial membrane potential analysis on BD LSRFortessa X-20 cell analyzer flow cytometer (BD Biosciences, Franklin Lakes, NJ, USA; S/N: R647800L6068) at the flow cytometry shared resource (FCSR), the Ohio State University Comprehensive Cancer Center, USA. ROS was developed in the cultured cells using CellROX^®^ green flow cytometry assay kits (Life Technologies, Waltham, MA, USA; Cat: C10492) according to the manufacturer’s instructions, in which the fluorescence emission was collected with 530/30 BP and 660/20 BP for CellROX^®^ green and SYTOX^®^ red reagents, respectively, within 120 min of incubation. On the other hand, caspase 3/7, annexin V apoptosis, and mitochondrial membrane potential activities were measured using NucView^®^ 488 Caspase-3, CF®488 A Annexin V and 7-AAD Apoptosis, and JC-1 mitochondrial membrane potential detection kits (Biotium Inc., Fremont, CA, USA). The fluorescence was measured in the green detection channel (fluorescein isothiocyanate; FITC) with excitation and emission of 485 and 515 nm, respectively, to profile the apoptotic cell population based on caspase 3/7 activity. Furthermore, CF®488 A Annexin V fluorescence was detected in the FITC channel while the 7-AAD fluorescence was detected in the PE-Cy®5 channel. Moreover, cationic dye- 5,5^’^,6,6^’^-tetrachloro-1,1^’^,3,3^’^-tetraethylbenzimidazolylcarbocyanine iodide was used to detect loss of mitochondrial membrane potential according to the manufacturer’s instructions.

### ELISA assay

According to the manufacturer’s instructions, the ELISA assay was used to evaluate IL-1β, IL-6, and NO levels. IL-1β and IL6 cytokine levels were measured in a plate reader at 405 nm (Thermo Fisher Scientific, Waltham, MA, USA), while nitric oxide (NO) level was determined colorimetrically according to the manufacturer’s instructions (Elabscience Bioinovation Inc., Texas, USA; Cat: E-BC-K035-S). The standard calibration curves were extrapolated and used to determine the cytokine concentrations (pg/mL).

### Statistical analysis

Quantitative data are presented as mean values accompanied by the standard error of the mean (SEM), providing a measure of variability within each dataset. Statistical comparisons between experimental groups were performed using either unpaired two-tailed Student’s t-tests or one-way analysis of variance (ANOVA), depending on the experimental design and number of groups involved. Post hoc analyses were applied where appropriate to further delineate group differences following ANOVA. All statistical computations and graphical representations were carried out using GraphPad Prism version 10 (GraphPad Software Inc., Boston, MA, USA), Fiji (ImageJ, USA), XLSTAT (Addinsoft Inc., Paris, France), and MetaboloAnalyst 6.0. Statistical significance was determined based on *p*-values, with thresholds defined as follows: *p < 0.05 (statistically significant), **p < 0.01 (highly significant), and ***p < 0.001 (very highly significant). These levels of significance are denoted in figures and tables using asterisks. In all experiments, n refers to independent biological replicates and for all unspecified replicates, *n* = 3. Technical replicates were used only to ensure measurement precision and were not treated as independent samples for statistical analysis.

## Supplementary information

Figure S1

Figure S2

Figure S3

Figure S4

Figure S5

Supplementary methods

Uncropped western blot

## Data Availability

Raw data, uncropped western blot, original IHC and IF microscopical images are attached in the supplementary files and are publicly available as of the date of publication. DNA-Seq and RNA-seq data generated in this publication have been deposited in NCBI’s Gene Expression Omnibus (GEO), Sequence Read Archive (SRA) and are accessible through the BioProject accession number PRJNA1359542 for whole genome and bulk RNA sequences and GSE310210 for gene counts, DEGs, and GSEA. Any additional information required to the data reported in this paper is available from the lead contact (Dr. Oladapo Fagbohun) upon request (oladapo.fagbohun@wilmington.edu).
