# Supplementary material for: Berry-derived gold nanoparticles induce integrated ROS-mediated apoptosis, immune modulation, and transcriptomic remodeling in 4T1 triple-negative cancer cells
Source: Cell Death Discov. 2026 Apr 10;12:225. doi: 10.1038/s41420-026-03023-z (PMC13184259; doi:10.1038/s41420-026-03023-z)
Supplement: Supplementary file 1 — Figure S1 [file 41420_2026_3023_MOESM1_ESM.pdf]

Figure S1: 4T1 Protein Sequences for Multiple Sequence Alignment

Gene Name	Gene Description	Genomic Coordinates
ATM	ataxia telangiectasia mutated	NC_000075.7:53348422-53448125
BARD1	BRCA1 associated RING domain 1	NC_000067.7:71066694-71142300
BRCA2	breast cancer 2, early onset	NC_000071.7:150445759-150493612
CDH1	cadherin 1	NC_000074.7:107329982-107396879
CHEK2	checkpoint kinase 2	NC_000071.7:110987668-111022006
NF1	neurofibromin 1	NC_000077.7:79223541-79472435
PALB2	partner and localizer of BRCA2	NC_000073.7:121706485-121732203
PTEN	phosphatase and tensin homolog	NC_000085.7:32734977-32803560
RAD51C	RAD51 paralog C	NC_000077.7:87267471-87295780
RAD51D	RAD51 paralog D	NC_000077.7:82762786-82781571
STK11	serine/threonine kinase 11	NC_000076.7:79951602-79966513
TP53	transformation related protein 53	NC_000077.7:69471174-69482699
BRCA1	Breast cancer 1, early onset	NC_000077.6

ATM GRCm39 – WT (CDS region obtained from m-RNA transcript NM_007499.3)

MSLALNDLLICCRQLEHDRATERRKEVDKFKRLIQDPETVQHLDRHSDSKQGKYLNWDAVFRFLQKYIQK
EMESLRTAKSNVSATTQSSRQKKMQEISSLVRYFIKCANKRAPRLKCQDLLNYVMDTVKDSSNGLTYGAD
CSNILLKDILSVRKYWCEVSQQQWLELFSLYFRLYLKPSQDINRVLVARIIHAVTRGCCSQTDGLPSKFL
DLFSKAIQYARQEKSSPGLSHILAALNIFLKSLAVNFRKRVCEAGDEILPTLLYIWTQHRLNDSLKEVII
ELIQLQIYIHHPQGARAPEEGAYESMKWKSILYNLYDLLVNEISHIGSRGKYSSGSRNIAVKENLIDLMA
DICYQLFDADTRSVEISQSYVTQRESTDYSVPCKRRKIDVGWEVIKDYLQKSQSDFDLVPWLQITTRLIS
KYPSSLPNCELSPLILILYQLLPQQRRGERIPYVLRCLKEVALCQGKKSNLESSQKSDLLKLWIKIWSIT
FRGISSGQTQTENFGLLEAIIQGSLVELDREFWKLFTGSACKPSSPSVCCLTLALSICVVPDAIKMGTEQ
SVCEANRSFSVKESIMRWLLFYQLEDDLEDSTELPPILQSNFPHLVVEKILVSLTMKNSKAAMKFFQSVP
ECEQHCEDKEEPSFSEVEELFLQTTFDKMDFLTTVKEYAVEKFQSSVGFSVQQNLKESLDHYLLGLSEQL
LSNYSSEITSSETLVRCSSLLVGVLGCYCYMGIITEDEAHKSELFQKAKSLMQCAGESISLFKNKTNEES
RIGSLRNVMHLCTSCLCIHTKHTPNKIASGFFLRLLTSKLMNDIADICKSLASCTKKPLDHGVHPGEDDE
DGGGCDSLMEAEGPSSTGLSTAYPASSVSDANDYGENQNAVGAMSPLAADYLSKQDHLLLDMLRFLGRSV
TASQSHTVSFRGADIRRKLLLLLDSSILDLMKPLHLHMYLVLLKDLPGNEHSLPMEDVVELLQPLSLVCS
LHRRDQDVCKTILSNVLHIVTNLGQGSVDMESTRIAQGHFLTVMGAFWHLTKEKKCVFSVRMALVKCLQT
LLEADPYSEWAILNVKGQDFPVNEAFSQFLADDHHQVRMLAAGSVNRLFQDMRQGDFSRSLKALPLKFQQ
TSFNNAYTTAEAGIRGLLCDSQNPDLLDEIYNRKSVLLMMIAVVLHCSPVCEKQALFALCKSVKENRLEP
HLVKKVLEKVSESFGCRSLEDFMISHLDYLVLEWLNLQDTEYSLSSFPFMLLNYTSIEDFYRSCYKILIP
HLVIRSHFDEVKSIANQIQKCWKSLLVDCFPKILVHILPYFAYEGTRDSYVSQKRETATKVYDTLKGEDF
LGKQIDQVFISNLPEIVVELLMTLHETADSADSDASQSATALCDFSGDLDPAPNPPYFPSHVIQATFAYI
SNCHKTKFKSILEILSKIPDSYQKILLAICEQAAETNNVFKKHRILKIYHLFVSLLLKDIQSGLGGAWAF
VLRDVIYTLIHYINKRSSHFTDVSLRSFSLCCDLLSRVCHTAVTQCKDALESHLHVIVGTLIPLVDYQEV
QEQVLDLLKYLVIDNKDNKNLSVTIKLLDPFPDHVIFKDLRLTQQKIKYSGGPFSLLEEINHFLSVSAYN
PLPLTRLEGLKDLRRQLEQHKDQMLDLLRASQDNPQDGIVVKLVVSLLQLSKMAVNQTGEREVLEAVGRC
LGEIGPLDFSTIAVQHNKDVSYTKAYGLPEDRELQWTLIMLTALNNTLVEDSVKIRSAAATCLKNILATK
IGHIFWENYKTSADPMLTYLQPFRTSRKKFLEVPRSVKEDVLEGLDAVNLWVPQSESHDIWIKTLTCAFL
DSGGINSEILQLLKPMCEVKTDFCQMLLPYLIHDVLLQDTHESWRTLLSAHVRGFFTSCFKHSSQASRSA
TPANSDSESENFLRCCLDKKSQRTMLAVVDYLRRQKRPSSGTAFDDAFWLDLNYLEVAKVAQSCSAHFTA
LLYAEIYSDKKSTDEQEKRSPTFEEGSQGTTISSLSEKSKEETGISLQDLLLEIYRSIGEPDSLYGCGGG
KMLQPLTRIRTYEHEATWEKALVTYDLETSISSSTRQSGIIQALQNLGLSHILSVYLKGLDYERREWCAE
LQELRYQAAWRNMQWGLCASAGQEVEGTSYHESLYNALQCLRNREFSTFYESLRYARVKEVEELSKGSLE
SVYSLYPTLSRLQAIGELENSGELFSRSVTDRERSEAYWKWQKHSQLLKDSDFSFQEPLMALRTVILETL
VQKEMERSQGACSKDILTKHLVEFSVLARTFKNTQLPERAIFKIKQYNSAICGISEWHLEEAQVFWAKKE
QSLALSILKQMIKKLDSSFKDKENDAGLKVIYAECLRVCGSWLAETCLENPAVIMQTYLEKAVKVAGSYD
GNSRELRNGQMKAFLSLARFSDTQYQRIENYMKSSEFENKQTLLKRAKEEVGLLREHKIQTNRYTVKVQR
ELELDECALRALREDRKRFLCKAVENYINCLLSGEEHDLWVFRLCSLWLENSGVSEVNGMMKKDGMKISS

YKFLPLMYQLAARMGTKMTGGLGFHEVLNNLISRISLDHPHHTLFIILALANANKDEFLSKPETTRRSRI
TKSTSKENSHLDEDRTEAATRIIHSIRSKRCKMVKDMEALCDAYIILANMDASQWRAQRKGINIPANQPI
TKLKNLEDVVVPTMEIKVDPTGEYENLVTIKSFKTEFRLAGGLNLPKIIDCVGSDGKERRQLVKGRDDLR
QDAVMQQVFQMCNTLLQRNTETRKRKLTICTYKVVPLSQRSGVLEWCTGTVPIGEYLVNSEDGAHRRYRP
NDFSANQCQKKMMEVQKKSFEEKYDTFMTICQNFEPVFRYFCMEKFLDPAVWFEKRLAYTRSVATSSIVG
YILGLGDRHVQNILINEQSAELVHIDLGVAFEQGKILPTPETVPFRLSRDIVDGMGITGVEGVFRRCCEK
TMEVMRSSQETLLTIVEVLLYDPLFDWTMNPLKALYLQQRPEDESDLHSTPNADDQECKQSLSDTDQSFN
KVAERVLMRLQEKLKGVEEGTVLSVGGQVNLLIQQAMDPKNLSRLFPGWKAWV.

855012 Sample Consensus for ATM→ No Change

MSLALNDLLICCRQLEHDRATERRKEVDKFKRLIQDPETVQHLDRHSDSKQGKYLNWDAVFRFLQKYIQKEM
ESLRTAKSNVSATTQSSRQKKMQEISSLVRYFIKCANKRAPRLKCQDLLNYVMDTVKDSSNGLTYGADCSNILL
KDILSVRKYWCEVSQQQWLELFSLYFRLYLKPSQDINRVLVARIIHAVTRGCCSQTDGLPSKFLDLFSKAIQYAR
QEKSSPGLSHILAALNIFLKSLAVNFRKRVCEAGDEILPTLLYIWTQHRLNDSLKEVIIELIQLQIYIHHPQGARAPE
EGAYESMKWKSILYNLYDLLVNEISHIGSRGKYSSGSRNIAVKENLIDLMADICYQLFDADTRSVEISQSYVTQRE
STDYSVPCKRRKIDVGWEVIKDYLQKSQSDFDLVPWLQITTRLISKYPSSLPNCELSPLILILYQLLPQQRRGERIP
YVLRCLKEVALCQGKKSNLESSQKSDLLKLWIKIWSITFRGISSGQTQTENFGLLEAIIQGSLVELDREFWKLFTG
SACKPSSPSVCCLTLALSICVVPDAIKMGTEQSVCEANRSFSVKESIMRWLLFYQLEDDLEDSTELPPILQSNFP
HLVVEKILVSLTMKNSKAAMKFFQSVPECEQHCEDKEEPSFSEVEELFLQTTFDKMDFLTTVKEYAVEKFQSSV
GFSVQQNLKESLDHYLLGLSEQLLSNYSSEITSSETLVRCSSLLVGVLGCYCYMGIITEDEAHKSELFQKAKSLMQ
CAGESISLFKNKTNEESRIGSLRNVMHLCTSCLCIHTKHTPNKIASGFFLRLLTSKLMNDIADICKSLASCTKKPLD
HGVHPGEDDEDGGGCDSLMEAEGPSSTGLSTAYPASSVSDANDYGENQNAVGAMSPLAADYLSKQDHLLLD
MLRFLGRSVTASQSHTVSFRGADIRRKLLLLLDSSILDLMKPLHLHMYLVLLKDLPGNEHSLPMEDVVELLQPLS
LVCSLHRRDQDVCKTILSNVLHIVTNLGQGSVDMESTRIAQGHFLTVMGAFWHLTKEKKCVFSVRMALVKCL
QTLLEADPYSEWAILNVKGQDFPVNEAFSQFLADDHHQVRMLAAGSVNRLFQDMRQGDFSRSLKALPLKFQ
QTSFNNAYTTAEAGIRGLLCDSQNPDLLDEIYNRKSVLLMMIAVVLHCSPVCEKQALFALCKSVKENRLEPHLV
KKVLEKVSESFGCRSLEDFMISHLDYLVLEWLNLQDTEYSLSSFPFMLLNYTSIEDFYRSCYKILIPHLVIRSHFDEV
KSIANQIQKCWKSLLVDCFPKILVHILPYFAYEGTRDSYVSQKRETATKVYDTLKGEDFLGKQIDQVFISNLPEIVV
ELLMTLHETADSADSDASQSATALCDFSGDLDPAPNPPYFPSHVIQATFAYISNCHKTKFKSILEILSKIPDSYQKIL
LAICEQAAETNNVFKKHRILKIYHLFVSLLLKDIQSGLGGAWAFVLRDVIYTLIHYINKRSSHFTDVSLRSFSLCCD
LLSRVCHTAVTQCKDALESHLHVIVGTLIPLVDYQEVQEQVLDLLKYLVIDNKDNKNLSVTIKLLDPFPDHVIFKD
LRLTQQKIKYSGGPFSLLEEINHFLSVSAYNPLPLTRLEGLKDLRRQLEQHKDQMLDLLRASQDNPQDGIVVKLV
VSLLQLSKMAVNQTGEREVLEAVGRCLGEIGPLDFSTIAVQHNKDVSYTKAYGLPEDRELQWTLIMLTALNNTL
VEDSVKIRSAAATCLKNILATKIGHIFWENYKTSADPMLTYLQPFRTSRKKFLEVPRSVKEDVLEGLDAVNLWVP
QSESHDIWIKTLTCAFLDSGGINSEILQLLKPMCEVKTDFCQMLLPYLIHDVLLQDTHESWRTLLSAHVRGFFTS
CFKHSSQASRSATPANSDSESENFLRCCLDKKSQRTMLAVVDYLRRQKRPSSGTAFDDAFWLDLNYLEVAKVA
QSCSAHFTALLYAEIYSDKKSTDEQEKRSPTFEEGSQGTTISSLSEKSKEETGISLQDLLLEIYRSIGEPDSLYGCGG

GKMLQPLTRIRTYEHEATWEKALVTYDLETSISSSTRQSGIIQALQNLGLSHILSVYLKGLDYERREWCAELQELR
YQAAWRNMQWGLCASAGQEVEGTSYHESLYNALQCLRNREFSTFYESLRYARVKEVEELSKGSLESVYSLYPT
LSRLQAIGELENSGELFSRSVTDRERSEAYWKWQKHSQLLKDSDFSFQEPLMALRTVILETLVQKEMERSQGA
CSKDILTKHLVEFSVLARTFKNTQLPERAIFKIKQYNSAICGISEWHLEEAQVFWAKKEQSLALSILKQMIKKLDSS
FKDKENDAGLKVIYAECLRVCGSWLAETCLENPAVIMQTYLEKAVKVAGSYDGNSRELRNGQMKAFLSLARFS
DTQYQRIENYMKSSEFENKQTLLKRAKEEVGLLREHKIQTNRYTVKVQRELELDECALRALREDRKRFLCKAVE
NYINCLLSGEEHDLWVFRLCSLWLENSGVSEVNGMMKKDGMKISSYKFLPLMYQLAARMGTKMTGGLGFH
EVLNNLISRISLDHPHHTLFIILALANANKDEFLSKPETTRRSRITKSTSKENSHLDEDRTEAATRIIHSIRSKRCKM
VKDMEALCDAYIILANMDASQWRAQRKGINIPANQPITKLKNLEDVVVPTMEIKVDPTGEYENLVTIKSFKTEF
RLAGGLNLPKIIDCVGSDGKERRQLVKGRDDLRQDAVMQQVFQMCNTLLQRNTETRKRKLTICTYKVVPLSQ
RSGVLEWCTGTVPIGEYLVNSEDGAHRRYRPNDFSANQCQKKMMEVQKKSFEEKYDTFMTICQNFEPVFRY
FCMEKFLDPAVWFEKRLAYTRSVATSSIVGYILGLGDRHVQNILINEQSAELVHIDLGVAFEQGKILPTPETVPFR
LSRDIVDGMGITGVEGVFRRCCEKTMEVMRSSQETLLTIVEVLLYDPLFDWTMNPLKALYLQQRPEDESDLHS
TPNADDQECKQSLSDTDQSFNKVAERVLMRLQEKLKGVEEGTVLSVGGQVNLLIQQAMDPKNLSRLFPGWK
AWV.

BARD1 GRCm39 – WT (CDS region obtained from m-RNA transcript NM_007525.3)

MPRRPPRVCSGNQPAPVPAMEPATDGLWAHSRAALARLEKLLRCSRCANILKEPVCLGGCEHIFCSGCISDCV
GSGCPVCYTPAWILDLKINRQLDSMIQLSSKLQNLLHDNKDSKDNTSRASLFGDAERKKNSIKMWFSPRSKKV
RYVVTKVSVQTQPQKAKDDKAQEASMYEFVSATPPVAVPKSAKTASRTSAKKHPKKSVAKINREENLRPETKS
RFDSKEELKEEKVVSCSQIPVMERPRVNGEIDLLASGSVVEPECSGSLTEVSLPLAEHIVSPDTVSKNEETPEKKV
CVKDLRSGGSNGNRKGCHRPTTSTSDSCGSNIPSTSRGIGEPALLAENVVLVDCSSLPSGQLQVDVTLRRKSNA
SDDPLSLSPGTPPPLLNNSTHRQMMSSPSTVKLSSGMPARKRNHRGETLLHIASIKGDIPSVEYLLQNGNDPN
VKDHAGWTPLHEACSHGHLKVVELLLQHNALVNTPGYQNDSPLHDAVKSGHIDIVKVLLSHGASRNAVNIFG
VRPVDYTDNENIRSLLLLPEENESFSTSQCSIVNTGQRKNGPLVFIGSGLSSQQQKMLSKLETVLKAKKCMEFD
STVTHVIVPDEEAQSTLKCMLGILSGCWILKFDWVKACLDSKVREQEEKYEVPGGPQRSRLNREQLLPKLFDG
CYFFLGGNFKHHPRDDLLKLIAAAGGKVLSRKPKPDSDVTQTINTVAYHAKPESDQRFCTQYIVYEDLFNCHPE
RVRQGKVWMAPSTWLISCIMAFELLPLDS.

855012 Sample Consensus for BARD1

MPRRPPRVCSGNQPAPVPAMEPATDGLWAHSRAALARLEKLLRCSRCANILKEPVCLGGCEHIFCSGCISDCV
GSGCPVCYTPAWILDLKINRQLDSMIQLSSKLQNLLHDNKDSKDNTSRASLFGDAERKKNSIKMWFSPRSKKV
RYVVTKVSVQTQPQKAKDDKAQEASMYEFVSATPPVAVPKSAKTASRTSAKKHPKKSVAKINREENLRPETKD
SRFDSKEELEGLPLQPTTSK.

BRCA2 GRCm39 – WT (CDS region obtained from m-RNA transcript NM_009765.3)

MPVEYKRRPTFWEIFKARCSTADLGPISLNWFEELSSEAPPYNSEPPEESEYKPHGYEPQLFKTPQRNPP
YHQFASTPIMFKERSQTLPLDQSPFRELGKVVASSKHKTHSKKKTKVDPVVDVASPPLKSCLSESPLTLR
CTQAVLQREKPVVSGSLFYTPKLKEGQTPKPISESLGVEVDPDMSWTSSLATPPTLSSTVLIARDEEARS
SVTPADSPATLKSCFSNHNESPQKNDRSVPSVIDSENKNQQEAFSQGLGKMLGDSSGKRNSFKDCLRKPI
PNILEDGETAVDTSEEDSFSLCFPKRRTRNLQKMRMGKTRKKIFSETRTDELSEEARRQTDDKNSFVFEM
ELRESDPLDPGVTSQKPFYSQNEEICNEAVQCSDSRWSQSNLSGLNETQTGKITLPHISSHSQNISEDFI
DMKKEGTGSITSEKSLPHISSLPEPEKMFSEETVVDKEHEGQHFESLEDSIAGKQMVSRTSQAACLSPSI
RKSIFKMREPLDETLGTVFSDSMTNSTFTEEHEASACGLGILTACSQREDSICPSSVDTGSWPTTLTDTS
ATVKNAGLISTLKNKKRKFIYSVSDDASLQGKKLQTHRQLELTNLSAQLEASAFEVPLTFTNVNSGIPDS
SDKKRCLPNDPEEPSLTNSFGTATSKEISYIHALISQDLNDKEAIVIEEKPQPYTAREADFLLCLPERTC
ENDQKSPKVSNGKEKVLVSACLPSAVQLSSISFESQENPLGDHNGTSTLKLTPSSKLPLSKADMVSREKM
CKMPEKLQCESCKVNIELSKNILEVNEICILSENSKTPGLLPPGENIIEVASSMKSQFNQNAKIVIQKDQ
KGSPFISEVAVNMNSEELFPDSGNNFAFQVTNKCNKPDLGSSVELQEEDLSHTQGPSLKNSPMAVDEDVD
DAHAAQVLITKDSDSLAVVHDYTEKSRNNIEQHQKGTEDKDFKSNSSLNMKSDGNSDCSDKWSEFLDPVL
NHNFGGSFRTASNKEIKLSEHNVKKSKMFFKDIEEQYPTRLACIDIVNTLPLANQKKLSEPHIFDLKSVT
TVSTQSHNQSSVSHEDTDTAPQMLSSKQDFHSNNLTTSQKAEITELSTILEESGSQFEFTQFRKPSHIAQ
NTSEVPGNQMVVLSTASKEWKDTDLHLPVDPSVGQTDHSKQFEGSAGVKQSFPHLLEDTCNKNTSCFLPN
INEMEFGGFCSALGTKLSVSNEALRKAMKLFSDIENSEEPSAKVGPRGFSSSAHHDSVASVFKIKKQNTE
KSFDEKSSKCQVTLQNNIEMTTCIFVGRNPEKYIKNTKHEDSYTSSQRNNLENSDGSMSSTSGPVYIHKG
DSDLPADQGSKCPESCTQYAREENTQIKENISDLTCLEIMKAEETCMKSSDKKQLPSDKMEQNIKEFNIS
FQTASGKNTRVSKESLNKSVNIFNRETDELTVISDSLNSKILHGINKDKMHTSCHKKAISIKKVFEDHFP
IVTVSQLPAQQHPEYEIESTKEPTLLSFHTASGKKVKIMQESLDKVKNLFDETQYVRKTASFSQGSKPLK
DSKKELTLAYEKIEVTASKCEEMQNFVSKETEMLPQQNYHMYRQTENLKTSNGTSSKVQENIENNVEKNP
RICCICQSSYPVTEDSALAYYTEDSRKTCVRESSLSKGRKWLREQGDKLGTRNTIKIECVKEHTEDFAGN
ASYEHSLVIIRTEIDTNHVSENQVSTLLSDPNVCHSYLSQSSFCHCDDMHNDSGYFLKNKIDSDVPPDMK
NAEGNTISPRVSATKERNLHPQTINEYCVQKLETNTSPHANKDVAIDPSLLDSRNCKVGSLVFITAHSQE
TERTKEIVTDNCYKIVEQNRQSKPDTCQTSCHKVLDDSKDFICPSSSGDVCINSRKDSFCPHNEQILQHN
QSMSGLKKAATPPVGLETWDTSKSIREPPQAAHPSRTYGIFSTASGKAIQVSDASLEKARQVFSEMDGDA
KQLSSMVSLEGNEKPHHSVKRENSVVHSTQGVLSLPKPLPGNVNSSVFSGFSTAGGKLVTVSESALHKVK
GMLEEFDLIRTEHTLQHSPIPEDVSKILPQPCAEIRTPEYPVNSKLQKTYNDKSSLPSNYKESGSSGNTQ
SIEVSLQLSQMERNQDTQLVLGTKVSHSKANLLGKEQTLPQNIKVKTDEMKTFSDVPVKTNVGEYYSKES
ENYFETEAVESAKAFMEDDELTDSEQTHAKCSLFTCPQNETLFNSRTRKRGGVTVDAVGQPPIKRSLLNE
FDRIIESKGKSLTPSKSTPDGTVKDRSLFTHHMSLEPVTCGPFCSSKERQGAQRPHLTSPAQELLSKGHP
WRHSALEKSPSSPIVSILPAHDVSATRTERTRHSGKSTKVFVPPFKMKSQFHGDEHFNSKNVNLEGKNQK
STDGDREDGNDSHVRQFNKDLMSSLQSARDLQDMRIKNKERRHLRLQPGSLYLTKSSTLPRISLQAAVGD
RAPSACSPKQLYIYGVSKECINVNSKNAEYFQFDIQDHFGKEDLCAGKGFQLADGGWLIPSNDGKAGKEE

FYRALCDTPGVDPKLISSIWVANHYRWIVWKLAAMEFAFPKEFANRCLNPERVLLQLKYRYDVEIDNSRR
SALKKILERDDTAAKTLVLCISDIISPSTKVSETSGGKTSGEDANKVDTIELTDGWYAVRAQLDPPLMAL
VKSGKLTVGQKIITQGAELVGSPDACAPLEAPDSLRLKISANSTRPARWHSRLGFFRDPRPFPLPLSSLF
SDGGNVGCVDIIVQRVYPLQWVEKTVSGLYIFRSEREEEKEALRFAEAQQKKLEALFTKVHTEFKDHEED
TTQRCVLSRTLTRQQVHALQDGAELYAAVQYASDPDHLEACFSEEQLRALNNYRQMLNDKKQARIQSEFR
KALESAEKEEGLSRDVTTVWKLRVTSYKKKEKSALLSIWRPSSDLSSLLTEGKRYRIYHLAVSKSKSKFE
RPSIQLTATKRTQYQQLPVSSETLLQVYQPRESLHFSRLSDPAFQPPCSEVDVVGVVVSVVKPIGLAPLV
YLSDECLNLLVVKFGIDLNEDIKPRVLIAASNLQCQPESTSGVPTLFAGHFSIFSASPKEAYFQEKVNNL
KHAIENIDTFYKEAEKKLIHVLEGDSPKWSTPNKDPTREPHAASTCCASDLLGSGGQFLRISPTGQQSYQ
SPLSHCTLKGKSMPLAHSAQMAAKSWSGENEIDDPKTCRKRRALDFLSRLPLPSPVSPICTFVSPAAQKA
FQPPRSCGTKYATPIKKEPSSPRRRTPFQKTSGVSLPDCDSVADEELALLSTQALTPDSVGGNEQAFPGD
STRNPQPAQRPDQQVGPRSRKESLRDCRGDSSEKLAVES.

855012 Sample Consensus for BRCA2

MPVEYKRRPTFWEIFKARCSTADLGPISLNWFEELSSEAPPYNSEPPEESEYKPHGYEPQLFKTPQRNPPYHQF
ASTPIMFKERSQTLPLDQSPFRELGKVVASSKHKTHSKKKTKVDPVVDVASPPLKSCLSESPLTLRCTQAVLQRE
KPVVSGSLFYTPKLKEGQTPKPISESLGVEVDPDMSWTSSLATPPTLSSTVLIARDEEARSSVTPADSPATLKSCF
SNHNESPQKNDRSVPSVIDSENKNQQEAFSQGLGKMLGDSSGKRNSFKDCLRKPIPNILEDGETAVDTSEEDS
FSLCFPKRRTRNLQKMRMGKTRKKIFSETRTDELSEEARRQTDDKNSFVFEMELRESDPLDPGVTSQKPFYSQ
NEEICNEAVQCSDSRWSQSNLSGLNETQTGKITLPHISSHSQNISEDFIDMKKEGTGSITSEKSLPHISSLPEPEK
MFSEETVVDKEHEGQHFESLEDSIAGKQMVSRTSQAACLSPSIRKSIFKMREPLDETLGTVFSDSMTNSTFTEE
HEASACGLGILTACSQREDSICPSSVDTGSWPTTLTDTSATVKNAGLISTLKNKKRKFIYSVSDDASLQGKKLQTH
RQLELTNLSAQLEASAFEVPLTFTNVNSGIPDSSDKKRCLPNDPEEPSLTNSFGTATSKEISYIHALISQDLNDKEA
IVIEEKPQPYTAREADFLLCLPERTCENDQKSPKVSNGKEKVLVSACLPSAVQLSSISFESQENPLGDHNGTSTLK
LTPSSKLPLSKADMVSREKMCKMPEKLQCESCKVNIELSKNILEVNEICILSENSKTPGLLPPGENIIEVASSMKS
QFNQNAKIVIQKDQKGSPFISEVAVNMNSEELFPDSGNNFAFQVTNKCNKPDLGSSVELQEEDLSHTQGPSL
KNSPMAVDEDVDDAHAAQVLITKDSDSLAVVHDYTEKSRNNIEQHQKGTEDKDFKSNSSLNMKSDGNSDCS
DKWSEFLDPVLNHNFGGSFRTASNKEIKLSEHNVKKSKMFFKDIEEQYPTRLACIDIVNTLPLANQKKLSEPHIF
DLKSVTTVSTQSHNQSSVSHEDTDTAPQMLSSKQDFHSNNLTTSQKAEITELSTILEESGSQFEFTQFRKPSHIA
QNTSEVPGNQMVVLSTASKEWKDTDLHLPVDPSVGQTDHSKQFEGSAGVKQSFPHLLEDTCNKNTSCFLPN
INEMEFGGFCSALGTKLSVSNEALRKAMKLFSDIENSEEPSAKVGPRGFSSSAHHDSVASVFKIKKQNTEKSFD
EKSSKCQVTLQNNIEMTTCIFVGRNPEKYIKNTKHEDSYTSSQRNNLENSDGSMSSTSGPVYIHKGDSDLPAD
QGSKCPESCTQYAREENTQIKENISDLTCLEIMKAEETCMKSSDKKQLPSDKMEQNIKEFNISFQTASGKNTRV
SKESLNKSVNIFNRETDELTVISDSLNSKILHGINKDKMHTSCHKKAISIKKVFEDHFPIVTVSQLPAQQHPEYEIE
STKEPTLLSFHTASGKKVKIMQESLDKVKNLFDETQYVRKTASFSQGSKPLKDSKKELTLAYEKIEVTASKCEEMQ
NFVSKETEMLPQQNYHMYRQTENLKTSNGTSSKVQENIENNVEKNPRICCICQSSYPVTEDSALAYYTEDSRK
TCVRESSLSKGRKWLREQGDKLGTRNTIKIECVKEHTEDFAGNASYEHSLVIIRTEIDTNHVSENQVSTLLSDPN

VCHSYLSQSSFCHCDDMHNDSGYFLKNKIDSDVPPDMKNAEGNTISPRVSATKERNLHPQTINEYCVQKLET
NTSPHANKDVAIDPSLLDSRNCKVGSLVFITAHSQETERTKEIVTDNCYKIVEQNRQSKPDTCQTSCHKVLDDS
KDFICPSSSGDVCINSRKDSFCPHNEQILQHNQSMSGLKKAATPPVGLETWDTSKSIREPPQAAHPSRTYGIFS
TASGKAIQVSDASLEKARQVFSEMDGDAKQLSSMVSLEGNEKPHHSVKRENSVVHSTQGVLSLPKPLPGNVN
SSVFSGFSTAGGKLVTVSESALHKVKGMLEEFDLIRTEHTLQHSPIPEDVSKILPQPCAEIRTPEYPVNSKLQKTY
NDKSSLPSNYKESGSSGNTQSIEVSLQLSQMERNQDTQLVLGTKVSHSKANLLGKEQTLPQNIKVKTDEMKTF
SDVPVKTNVGEYYSKESENYFETEAVESAKAFMEDDELTDSEQTHAKCSLFTCPQNETLFNSRTRKRGGVTVD
AVGQPPIKRSLLNEFDRIIESKGKSLTPSKSTPDGTVKDRSLFTHHMSLEPVTCGPFCSSKERQGAQRPHLTSPA
QELLSKGHPWRHSALEKSPSSPIVSILPAHDVSATRTERTRHSGKSTKVFVPPFKMKSQFHGDEHFNSKNVNLE
GKNQKSTDGDREDGNDSHVRQFNKDLMSSLQSARDLQDMRIKNKERRHLRLQPGSLYLTKSSTLPRISLQAA
VGDRAPSACSPKQLYIYGVSKECINVNSKNAEYFQFDIQDHFGKEDLCAGKGFQLADGGWLIPSNDGKAGKE
EFYRALCDTPGVDPKLISSIWVANHYRWIVWKLAAMEFAFPKEFANRCLNPERVLLQLKYRYDVEIDNSRRSA
LKKILERDDTAAKTLVLCISDIISPSTKVSETSGGKTSGEDANKVDTIELTDGWYAVRAQLDPPLMALVKSGKLTV
GQKIITQGAELVGSPDACAPLEAPDSLRLKISANSTRPARWHSRLGFFRDPRPFPLPLSSLFSDGGNVGCVDIIV
QRVYPLQWVEKTVSGLYIFRSEREEEKEALRFAEAQQKKLEALFTKVHTEFKDHEEDTTQRCVLSRTLTRQQVH
ALQDGAELYAAVQYASDPDHLEACFSEEQLRALNNYRQMLNDKKQARIQSEFRKALESAEKEEGLSRDVTTV
WKLRVTSYKKKEKSALLSIWRPSSDLSSLLTEGKRYRIYHLAVSKSKSKFERPSIQLTATKRTQYQQLPVSSETLLQ
VYQPRESLHFSRLSDPAFQPPCSEVDVVGVVVSVVKPIGLAPLVYLSDECLNLLVVKFGIDLNEDIKPRVLIAASN
LQCQPESTSGVPTLFACHFSIFSASPKEAYFQEKVNNLKHAIENIDTFYKEAEKKLIHVLEGDSPKWSTPNKDPT
REPHAASTCCASDLLGSGGQFLRISPTGQQSYQSPLSHCTLKGKSMPLAHSAQMAAKSWSGENEIDDPKTCR
KRRALDFLSRLPLPSPVSPICTFVSPAAQKAFQPPRSCGTKYATPIKKEPSSPRRRTPFQKTSGVSLPDCDSVADE
ELALLSTQALTPDSVGGNEQAFPGDSTRNPQPAQRPDQQVGPRSRKESLRDCRGDSSEKLAVES.

CDH1 GRCm39 – WT (CDS region obtained from m-RNA transcript NM_009864.3)

MGARCRSFSALLLLLQVSSWLCQELEPESCSPGFSSEVYTFPVPERHLERGHVLGRVRFEGCTGRPRTAF
FSEDSRFKVATDGTITVKRHLKLHKLETSFLVRARDSSHRELSTKVTLKSMGHHHHRHHHRDPASESNPE
LLMFPSVYPGLRRQKRDWVIPPISCPENEKGEFPKNLVQIKSNRDKETKVFYSITGQGADKPPVGVFIIE
RETGWLKVTQPLDREAIAKYILYSHAVSSNGEAVEDPMEIVITVTDQNDNRPEFTQPVFEGFVAEGAVPG
TSVMKVSATDADDDVNTYNAAIAYTIVSQDPELPHKNMFTVNRDTGVISVLTSGLDRESYPTYTLVVQAA
DLQGEGLSTTAKAVITVKDINDNAPVFNPSTYQGQVPENEVNARIATLKVTDDDAPNTPAWKAVYTVVND
PDQQFVVVTDPTTNDGILKTAKGLDFEAKQQYILHVRVENEEPFEGSLVPSTATVTVDVVDVNEAPIFMP
AERRVEVPEDFGVGQEITSYTAREPDTFMDQKITYRIWRDTANWLEINPETGAIFTRAEMDREDAEHVKN
STYVALIIATDDGSPIATGTGTLLLVLLDVNDNAPIPEPRNMQFCQRNPQPHIITILDPDLPPNTSPFTA
ELTHGASVNWTIEYNDAAQESLILQPRKDLEIGEYKIHLKLADNQNKDQVTTLDVHVCDCEGTVNNCMKA
GIVAAGLQVPAILGILGGILALLILILLLLLFLRRRTVVKEPLLPPDDDTRDNVYYYDEEGGGEEDQDFD
LSQLHRGLDARPEVTRNDVAPTLMSVPQYRPRPANPDEIGNFIDENLKAADSDPTAPPYDSLLVFDYEGS
GSEAASLSSLNSSESDQDQDYDYLNEWGNRFKKLADMYGGGEDD.

855012 Sample Consensus for CDH1

MGARCRSFSALLLLLQVSSWLCQELEPESCSPGFSSEVYTFPVPERHLERGHVLGRVRFEGCTGRPRTAFFSED
SRFKVATDGTITVKRHLKLHKLETSFLVRARDSSHRELSTKVTLKSMGHHHHRHHHRDPASESNPELLMFPSVY
PGLRRQKRDWVIPPISCPENEKGEFPKNLVQIKSNRDKETKVFYSITGQGADKPPVGVFIIERETGWLKVTQPL
DREAIAKYILYSHAVSSNGEAVEDPMEIVITVTDQNDNRPEFTQEVFEGSVAEGAVPGTSVMKVSATDADDDV
NTYNAAIAYTIVSQDPELPHKNMFTVNRDTGVISVLTSGLDRESYPTYTLVVQAADLQGEGLSTTAKAVITVKDI
NDNAPVFNPSTYQGQVPENEVNARIATLKVTDDDAPNTPAWKAVYTVVNDPDQQFVVVTDPTTNDGILKT
AKGLDFEAKQQYILHVRVENEEPFEGSLVPSTATVTVDVVDVNEAPIFMPAERRVEVPEDFGVGQEITSYTARE
PDTFMDQKITYRIWRDTANWLEINPETGAIFTRAEMDREDAEHVKNSTYVALIIATDDGSPIATGTGTLLLVLL
DVNDNAPIPEPRNMQFCQRNPQPHIITILDPDLPPNTSPFTAELTHGASVNWTIEYNDAAQESLILQPRKDLEI
GEYKIHLKLADNQNKDQVTTLDVHVCDCEGTVNNCMKAGIVAAGLQVPAILGILGGILALLILILLLLLFLRRRTV
VKEPLLPPDDDTRDNVYYYDEEGGGEEDQDFDLSQLHRGLDARPEVTRNDVAPTLMSVPQYRPRPANPDEI
GNFIDENLKAADSDPTAPPYDSLLVFDYEGSGSEAASLSSLNSSESDQDQDYDYLNEWGNRFKKLADMYGGG
EDD.

CHEK2 GRCm39 – WT (CDS region obtained from m-RNA transcript NM_016681.4)

MKSHHQSHSSTSSKAHDSASCSQSQGGFSQPQGTPSQLHELSQYQGSSSSSTGTVPSSSQSSHSSSGTLS
SLETVSTQELCSIPEDQEPEEPGPAPWARLWALQDGFSNLDCVNDNYWFGRDKSCEYCFDGPLLRRTDKY
RTYSKKHFRIFREMGPKNCYIVYIEDHSGNGTFVNTELIGKGKRCPLSNNSEIALSLCRNKVFVFFDLTV
DDQSVYPKELRDEYIMSKTLGSGACGEVKMAFERKTCQKVAIKIISKRRFALGSSREADTAPSVETEIEI
LKKLNHPCIIKIKDVFDAEDYYIVLELMEGGELFDRVVGNKRLKEATCKLYFYQMLVAVQYLHENGIIHR
DLKPENVLLSSQEEDCLIKITDFGQSKILGETSLMRTLCGTPTYLAPEVLVSNGTAGYSRAVDCWSLGVI
LFICLSGYPPFSEHKTQVSLKDQITSGKYNFIPEVWTDVSEEALDLVKKLLVVDPKARLTTEEALNHPWL
QDEYMKKKFQDLLVQEKNSVTLPVAPAQTSSQKRPLELEVEGMPSTKRLSVCGAVL.

855012 Sample Consensus for CHEK2

MKSHHQSHSSTSSKAHDSASCSQSQGGFSQPQGTPSQLHELSQYQGSSSSSTGTVPSSSQSSHSSSGTLSSLE
TVSTQELCSIPEDQEPEEPGPAPWARLWALQDGFSNLDCVNDNYWFGRDKSCEYCFDGPLLRRTDKYRTYSK
KHFRIFREMGPKNCYIVYIEDHSGNGTFVNTELIGKGKRCPLSNNSEIALSLCRNKVFVFFDLTVDDQSVYPKEL
RDEYIMSKTLGSGACGEVKMAFERKTCQKVAIKIISKRRFALGSSREADTAPSVETEIEILKKLNHPCIIKIKDVFD
AEDYYIVLELMEGGELFDRVVGNKRLKEATCKLYFYQMLVAVQYLHENGIIHRDLKPENVLLSSQEEDCLIKITDF
GQSKILGETSLMRTLCGTPTYLAPEVLVSNGTAGYSRAVDCWSLGVILFICLSGYPPFSEHKTQVSLKDQITSGKY
NFIPEVWTDVSEEALDLVKKLLVVDPKARLTTEEALNHPWLQDEYMKKKFQDLLVQEKNSVTLPVAPAQVF.

NF1 GRCm39 – WT (CDS region obtained from m-RNA transcript NM_010897.2)

MAAHRPVEWVQAVVSRFDEQLPIKTGQQNTHTKVSTEHNKECLINISKYKFSLVISGLTTILKNVNNMRI
FGEAAEKNLYLSQLIILDTLEKCLAGQPKDTMRLDETMLVKQLLPEICHFLHTCREGNQHAAELRNSASG
VLFSLSCNNFNAVFSRISTRLQELTVCSEDNVDVHDIELLQYINVDCAKLKRLLKETAFKFKALKKVAQL
AVINSLEKAFWNWVENYPDEFTKLYQIPQTDMAECAEKLFDLVDGFAESTKRKAAVWPLQIILLILCPEI
IQDISKDVVDESNINKKLFLDSLRKALAGHGGSRQLTESAAIACVKLCKASTYINWEDNSVIFLLVQSMV
VDLKNLLFNPSKPFSRGSQPADVDLMIDCLVSCFRISPHNNQHFKICLAQNSPSTFHYVLVNSLHRIITN
SALDWWPKIDAVYCHSVELRNMFGETLHKAVQGCGAHPAIRMAPSLTFKEKVTSLKFKEKPTDLETRSYK
CLLLSMVKLIHADPKLLLCNPRKQGPETQSSTAELITGLVQLVPQSHMPEVAQEAMEALLVLHQLDSIDL
WNPDAPVETFWEISSQMLFYICKKLTSHQMLSSTEILKWLREILICRNKFLLKNKQADRSSCHSLYLYGV
GCEMSATGNTTQMSVDHDEFLRACTPGASLRKGRGNSSMDSTAGCSGTPPICRQAQTKLEVALYMFLWNP
DTEAVLVAMSCFRHLCEEADIRCGVDEVSVHNFLPNYNTFMEFASVSNMMSTGRAALQKRVMALLRRIEH
PTAGNIEAWEDTHAKWEQATKLILNYPKAKMEDGQAAESLHKTIVKRRMSHVSGGGSIDLSDTDSLQEWI
NMTGFLCALGGVCLQQRSSSGLATYSPPMGAVSERKGSMISVMSSEGNIDSPVSRFMDRLLSLMVCNHEK
VGLQIRTNVKDLVGLELSPALYPMLFNKLKNTISKFFDSQGQVLLSDSNTQFVEQTIAIMKNLLDNHTEG
SSEHLGQASIETMMLNLVRYVRVLGNMVHAIQIKTKLCQLVEVMMARRDDLSFCQEMKFRNKMVEYLTDW
VMGTSNQAADDDIKCLTRDLDQASMEAVVSLLAGLPLQPEEGDGVELMEAKSQLFLKYFTLFMNLLNDCS
EVEDENAQTGGRKRGMSRRLASLRHCTVLAMSNLLNANVDSGLMHSIGLGYHKDLQTRATFMEVLTKILQ
QGTEFDTLAETVLADRFERLVELVTMMGDQGELPIAMALANVVPCSQWDELARVLVTLFDSRHLLYQLLW
NMFSKEVELADSMQTLFRGNSLASKIMTFCFKVYGATYLQKLLDPLLRVIITSSDWQHVSFEVDPTRLEP
SESLEENQRNLLQMTEKFFHAIISSSSEFPSQLRSVCHCLYQATCHSLLNKATVKERKENKKSVVSQRFP
QNSIGAVGSAMFLRFINPAIVSPYEAGILDKKPPPRIERGLKLMSKVLQSIANHVLFTKEEHMRPFNDFV
KSNFDLARRFFLDIASDCPTSDAVNHSLSFISDGNVLALHRLLWNNQEKIGQYLSSNRDHKAVGRRPFDK
MATLLAYLGPPEHKPVADTHWSSLNLTSSKFEEFMTRHQVHEKEEFKALKTLSIFYQAGTSKAGNPIFYY
VARRFKTGQINGDLLIYHVLLTLKPYYAKPYEIVVDLTHTGPSNRFKTDFLSKWFVVFPGFAYDNVSAVY
IYNCNSWVREYTKYHERLLTGLKGSKRLIFIDCPGKLAEHIEHEQQKLPAATLALEEDLKVFHNALKLAH
KDTKVSIKVGSTAVQVTSAERTKVLGQSVFLNDIYYASEIEEICLVDENQFTLTIANQGTPLTFMHQECE
AIVQSIIHIRTRWELSQPDSIPQHTKIRPKDVPGTLLNIALLNLGSSDPSLRSAAYNLLCALTCTFNLKI
EGQLLETSGLCIPANNTLFIVSISKTLAANEPHLTLEFLEECISGFSKSSIELKHLCLEYMTPWLSNLVR
FCKHNDDAKRQRVTAILDKLITMTINEKQMYPSIQAKIWGSLGQITDLLDVVLDSFIKTSATGGLGSIKA
EVMADTAVALASGNVKLVSSKVIGRMCKIIDKTCLSPTPTLEQHLMWDDIAILARYMLMLSFNNSLDVAA
HLPYLFHVVTFLVATGPLSLRASTHGLLINIIHSLCTCSQLHFSEETKQVLRLSLTEFSLPKFYLLFGIS
KVKSAAVIAFRSSYRDRSFSPGSYERETFALTSLETVTEALLEIMEACMRDIPTCKWLDQWTELAQRFAF
QYNPSLQPRALVVFGCISKRVSHGQIKQIIRILSKALESCLKGPDTYNSQVLIESTVIALTKLQPLLNKD
SPLHKALFWVAVAVLQLDEVNLYSAGTALLEQNLHTLDSLRIFNDKSPEEVFMAIRNPLEWHCKQMDHFV
GLNFNSNFNFALVGHLLKGYRHPSPAIVARTVRILHTLLTLVNKHRNCDKFEVNTQSVAYLAALLTVSEE
VRSRCSLKHRKSLLLTDISMENVPMDTYPIHHGDPSYRTLKETQPWSSPKGSEGYLAATYPAVGQTSPRA

RKSMSLDMGQPSQANTKKLLGTRKSFDHLISDTKAPKRQEMESGITTPPKMRRVAETDYEMETQRIPSSQ
QHPHLRKVSVSESNVLLDEEVLTDPKIQALLLTVLATLVKYTTDEFDQRILYEYLAEASVVFPKVFPVVH
NLLDSKINTLLSLCQDPNLLNPIHGIVQSVVYHEESPPQYQTSYLQSFGFNGLWRFAGPFSKQTQIPDYA
ELIVKFLDALIDTYLPGIDEETSEESLLTPTSPYPPALQSQLSITANLNLSNSMTSLATSQHSPGLDKEN
VELSPTAGHCNSGRTRHGSASQVQKQRSAGSFKRNSIKKIV.

855012 Sample Consensus for NF1

MAAHRPVEWVQAVVSRFDEQLPIKTGQQNTHTKVSTEHNKECLINISKYKFSLVISGLTTILKNVNNMRIFGEA
AEKNLYLSQLIILDTLEKCLAGQPKDTMRLDETMLVKQLLPEICHFLHTCREGNQHAAELRNSASGVLFSLSCN
NFNAVFSRISTRLQELTVCSEDNVDVHDIELLQYINVDCAKLKRLLKETAFKFKALKKVAQLAVINSLEKAFWNW
VENYPDEFTKLYQIPQTDMAECAEKLFDLVDGFAESTKRKAAVWPLQIILLILCPEIIQDISKDVVDESNINKKLFL
DSLRKALAGHGGSRQLTESAAIACVKLCKACTYINWEDNSVIFLLVQSMVVDLKNLLFNPSKPFSRGSQPADV
DLMIDCLVSCFRISPHNNQHFKICLAQNSPSTFHYVLVNSLHRIITNSALDWWPKIDAVYCHSVELRNMFGETL
HKAVQGCGAHPAIRMAPSLTFKEKVTSLKFKEKPTDLETRSYKCLLLSMVKLIHADPKLLLCNPRKQGPETQSST
AELITGLVQLVPQSHMPEVAQEAMEALLVLHQLDSIDLWNPDAPVETFWEISSQMLFYICKKLTSHQMLSSTEI
LKWLREILICRNKFLLKNKQADRSSCHSLYLYGVGCEMSATGNTTQMSVDHDEFLRACTPGASLRKGRGNSS
MDSTAGCSGTPPICRQAQTKLEVALYMFLWNPDTEAVLVAMSCFRHLCEEADIRCGVDEVSVHNFLPNYNTF
MEFASVSNMMSTGRAALQKRVMALLRRIEHPTAGNIEAWEDTHAKWEQATKLILNYPKAKMEDGQAAESL
HKTIVKRRMSHVSGGGSIDLSDTDSLQEWINMTGFLCALGGVCLQQRSSSGLATYSPPMGAVSERKGSMISV
MSSEGNIDSPVSRFMDRLLSLMVCNHEKVGLQIRTNVKDLVGLELSPALYPMLFNKLKNTISKFFDSQGQVLLS
DSNTQFVEQTIAIMKNLLDNHTEGSSEHLGQASIETMMLNLVRYVRVLGNMVHAIQIKTKLCQLVEVMMAR
RDDLSFCQEMKFRNKMVEYLTDWVMGTSNQAADDDIKCLTRDLDQASMEAVVSLLAGLPLQPEEGDGVEL
MEAKSQLFLKYFTLFMNLLNDCSEVEDENAQTGGRKRGMSRRLASLRHCTVLAMSNLLNANVDSGLMHSIG
LGYHKDLQTRATFMEVLTKILQQGTEFDTLAETVLADRFERLVELVTMMGDQGELPIAMALANVVPCSQWD
ELARVLVTLFDSRHLLYQLLWNMFSKEVELADSMQTLFRGNSLASKIMTFCFKVYGATYLQKLLDPLLRVIITSS
DWQHVSFEVDPTRLEPSESLEENQRNLLQMTEKFFHAIISSSSEFPSQLRSVCHCLYQATCHSLLNKATVKERKE
NKKSVVSQRFPQNSIGAVGSAMFLRFINPAIVSPYEAGILDKKPPPRIERGLKLMSKVLQSIANHVLFTKEEHM
RPFNDFVKSNFDLARRFFLDIASDCPTSDAVNHSLSFISDGNVLALHRLLWNNQEKIGQYLSSNRDHKAVGRR
PFDKMATLLAYLGPPEHKPVADTHWSSLNLTSSKFEEFMTRHQVHEKEEFKALKTLSIFYQAGTSKAGNPIFYY
VARRFKTGQINGDLLIYHVLLTLKPYYAKPYEIVVDLTHTGPSNRFKTDFLSKWFVVFPGFAYDNVSAVYIYNCN
SWVREYTKYHERLLTGLKGSKRLIFIDCPGKLAEHIEHEQQKLPAATLALEEDLKVFHNALKLAHKDTKVSIKVGS
TAVQVTSAERTKVLGQSVFLNDIYYASEIEEICLVDENQFTLTIANQGTPLTFMHQECEAIVQSIIHIRTRWELSQ
PDSIPQHTKIRPKDVPGTLLNIALLNLGSSDPSLRSAAYNLLCALTCTFNLKIEGQLLETSGLCIPANNTLFIVSISKT
LAANEPHLTLEFLEECISGFSKSSIELKHLCLEYMTPWLSNLVRFCKHNDDAKRQRVTAILDKLITMTINEKQMY
PSIQAKIWGSLGQITDLLDVVLDSFIKTSATGGLGSIKAEVMADTAVALASGNVKLVSSKVIGRMCKIIDKTCLSP
TPTLEQHLMWDDIAILARYMLMLSFNNSLDVAAHLPYLFHVVTFLVATGPLSLRASTHGLLINIIHSLCTCSQLH
FSEETKQVLRLSLTEFSLPKFYLLFGISKVKSAAVIAFRSSYRDRSFSPGSYERETFALTSLETVTEALLEIMEACMR

DIPTCKWLDQWTELAQRFAFQYNPSLQPRALVVFGCISKRVSHGQIKQIIRILSKALESCLKGPDTYNSQVLIEST
VIALTKLQPLLNKDSPLHKALFWVAVAVLQLDEVNLYSAGTALLEQNLHTLDSLRIFNDKSPEEVFMAIRNPLE
WHCKQMDHFVGLNFNSNFNFALVGHLLKGYRHPSPAIVARTVRILHTLLTLVNKHRNCDKFEVNTQSVAYLAA
LLTVSEEVRSRCSLKHRKSLLLTDISMENVPMDTYPIHHGDPSYRTLKETQPWSSPKGSEGYLAATYPAVGQTSP
RARKSMSLDMGQPSQANTKKLLGTRKSFDHLISDTKAPKRQEMESGITTPPKMRRVAETDYEMETQRIPSSQ
QHPHLRKVSVSESNVLLDEEVLTDPKIQALLLTVLATLVKYTTDEFDQRILYEYLAEASVVFPKVFPVVHNLLDSKI
NTLLSLCQDPNLLNPIHGIVQSVVYHEESPPQYQTSYLQSFGFNGLWRFAGPFSKQTQIPDYAELIVKFLDALIDT
YLPGIDEETSEESLLTPTSPYPPALQSQLSITANLNLSNSMTSLATSQHSPGLDKENVELSPTAGHCNSGRTRHG
SASQVQKQRSAGSFKRNSIKKIV.

PALB2 GRCm39 – WT (CDS region obtained from m-RNA transcript NM_001081238.2)

MEELSGKPLSYAEKEKLKEKLAFLKKEYSRTLARLQRAKRAEKAKNSKKAIEDGVPQPEASSQLSHSESI
NKGFPCDTLQSNHLDEETGENISQILDVEPQSFNCKQGKEVLHTPRAGDIQGQLLHSTSSPDGKKEQNTL
PGTTKTPWEKSSVSQEKEDYFDTNSLALLGKHRKGQESISRKNSRTPVSEKTHLLSLRSQIPDPPALVTG
IGEGILIPPSGKSERGIDTLVRGNTVSAEAAVPSCTASNSNHSQHLEHTPPKSGCKITTQGPASSTNLVA
QDQKMTIFTVNSVVYKAVRAHGQLPGSPNSCSVNDLTHSNLPANSTPNSKSLKSPSNTVDERNEPLQEDE
ILGPSKNFNLAAVSPPSTESQIHSCTMLEGLLFPAEYYVRTTRRMSDCQRKIALEAVIQSHLGVKKKELK
KKTKATKAVVLSSEDTDQSESGMLDTSTGQSSSGSLSQKLLSPAEVSSPPGPAGKATTPPPGRGHRGKRK
SARTSTLGHCQLLFPPCAALAVNRSKGKFTKHKCQNRGVVIHDFELPDEDFGLLKLEKLKSCSEKLIESP
DSKNCGERLPREGNHAALEELQRDSETEGLEEELTVPPGEAYRPGPTLRRQPGSKDLSSSIVLFTPADTA
APNDSGRPPPSLCSPAFPILGMTPALGSQAAGETLSTEAAQPCSTSQPPLLGDTNSLVNNSKQCNSSACS
PKPDTNLQASGRQGQPACDSDSGPQATPLPVESFTFRENQLCGNACLELHEHSTEQTETADRPACDNLNP
GNLQLVSELKNPSSSCSVDVSAMWWERAGAKEPCIVTACEDVVSLWKPLNSLQWEKVHTWHFTEVPVLQI
VPVPDVYNLICVALGSLEIREIRALLCSSGDDSEKQVLLKSGDIKAMLGLTKRRLVSSTGTFCNQQIQIM
TFADDGSSKDEQLLMPPDETVLTFAEVQGTQEALLGTTTVNSIVIWNLKTGQLLKKMHIDDSYQASVCHG
AYSEKGLLFVVVSQPCAKESQALGSPVFQLLVINPKTAQSVGVLLCSLPQGQAGRFLEGDVKDHVAAAVL
TSGTIAIWDLLLGHCTALLPPVSDQSWSLVKWSGTDSHLLAGQKDGNIFIYRYF.

855012 Sample Consensus for PALB2→ No Change

MEELSGKPLSYAEKEKLKEKLAFLKKEYSRTLARLQRAKRAEKAKNSKKAIEDGVPQPEASSQLSHSESINKGFP
CDTLQSNHLDEETGENISQILDVEPQSFNCKQGKEVLHTPRAGDIQGQLLHSTSSPDGKKEQNTLPGTTKTP
WEKSSVSQEKEDYFDTNSLALLGKHRKGQESISRKNSRTPVSEKTHLLSLRSQIPDPPALVTGIGEGILIPPSGKSE
RGIDTLVRGNTVSAEAAVPSCTASNSNHSQHLEHTPPKSGCKITTQGPASSTNLVAQDQKMTIFTVNSVVYKA
VRAHGQLPGSPNSCSVNDLTHSNLPANSTPNSKSLKSPSNTVDERNEPLQEDEILGPSKNFNLAAVSPPSTESQ
IHSCTMLEGLLFPAEYYVRTTRRMSDCQRKIALEAVIQSHLGVKKKELKKKTKATKAVVLSSEDTDQSESGMLD
TSTGQSSSGSLSQKLLSPAEVSSPPGPAGKATTPPPGRGHRGKRKSARTSTLGHCQLLFPPCAALAVNRSKGKF
TKHKCQNRGVVIHDFELPDEDFGLLKLEKLKSCSEKLIESPDSKNCGERLPREGNHAALEELQRDSETEGLEEEL
TVPPGEAYRPGPTLRRQPGSKDLSSSIVLFTPADTAAPNDSGRPPPSLCSPAFPILGMTPALGSQAAGETLSTEA
AQPCSTSQPPLLGDTNSLVNNSKQCNSSACSPKPDTNLQASGRQGQPACDSDSGPQATPLPVESFTFRENQL
CGNACLELHEHSTEQTETADRPACDNLNPGNLQLVSELKNPSSSCSVDVSAMWWERAGAKEPCIVTACEDV
VSLWKPLNSLQWEKVHTWHFTEVPVLQIVPVPDVYNLICVALGSLEIREIRALLCSSGDDSEKQVLLKSGDIKA
MLGLTKRRLVSSTGTFCNQQIQIMTFADDGSSKDEQLLMPPDETVLTFAEVQGTQEALLGTTTVNSIVIWNLK
TGQLLKKMHIDDSYQASVCHGAYSEKGLLFVVVSQPCAKESQALGSPVFQLLVINPKTAQSVGVLLCSLPQGQ
AGRFLEGDVKDHVAAAVLTSGTIAIWDLLLGHCTALLPPVSDQSWSLVKWSGTDSHLLAGQKDGNIFIYRYF.

PTEN GRCm39 – WT (CDS region obtained from m-RNA transcript NM_008960.2)

MTAIIKEIVSRNKRRYQEDGFDLDLTYIYPNIIAMGFPAERLEGVYRNNIDDVVRFLDSKHKNHYKIYNL
CAERHYDTAKFNCRVAQYPFEDHNPPQLELIKPFCEDLDQWLSEDDNHVAAIHCKAGKGRTGVMICAYLL
HRGKFLKAQEALDFYGEVRTRDKKGVTIPSQRRYVYYYSYLLKNHLDYRPVALLFHKMMFETIPMFSGGT
CNPQFVVCQLKVKIYSSNSGPTRREDKFMYFEFPQPLPVCGDIKVEFFHKQNKMLKKDKMFHFWVNTFFI
PGPEETSEKVENGSLCDQEIDSICSIERADNDKEYLVLTLTKNDLDKANKDKANRYFSPNFKVKLYFTKT
VEEPSNPEASSSTSVTPDVSDNEPDHYRYSDTTDSDPENEPFDEDQHSQITKV.

855012 Sample Consensus for PTEN → No Change

MTAIIKEIVSRNKRRYQEDGFDLDLTYIYPNIIAMGFPAERLEGVYRNNIDDVVRFLDSKHKNHYKIYNLCAERH
YDTAKFNCRVAQYPFEDHNPPQLELIKPFCEDLDQWLSEDDNHVAAIHCKAGKGRTGVMICAYLLHRGKFLK
AQEALDFYGEVRTRDKKGVTIPSQRRYVYYYSYLLKNHLDYRPVALLFHKMMFETIPMFSGGTCNPQFVVCQL
KVKIYSSNSGPTRREDKFMYFEFPQPLPVCGDIKVEFFHKQNKMLKKDKMFHFWVNTFFIPGPEETSEKVEN
GSLCDQEIDSICSIERADNDKEYLVLTLTKNDLDKANKDKANRYFSPNFKVKLYFTKTVEEPSNPEASSSTSVTPD
VSDNEPDHYRYSDTTDSDPENEPFDEDQHSQITKV.

RAD51C GRCm39 – WT (CDS region obtained from m-RNA transcript NM_053269.4)

MQRELVGYPLSPAVRGKLVAAGFQTAEDVLEVKPSELSKEVGISKEEALETLQILRRECLTNKPRCAGTSVANEK
CTALELLEQEHTQGFIITFCSALDNILGGGIPLMKTTEVCGVPGVGKTQLCMQLAVDVQIPECFGGVAGEAVFI
DTEGSFMVDRVVSLATACIQHLHLIAGTHTEEEHQKALKDFTLENILSHIYYFRCHDYTELLAQVYLLPDFLSDH
PKVQLVIIDGIAFPFRHDLEDLSLRTRLLNGLAQQMISLANNHRLAVILTNQMTTKIDKNQALLVPALGESWGH
AATIRLIFHWEQKQRFATLYKSPSQKESTIPFQITPQGFRDAVVTAASSQTESSLNFRKRSREPEEEC.

855012 Sample Consensus for RAD51C→ No Change

MQRELVGYPLSPAVRGKLVAAGFQTAEDVLEVKPSELSKEVGISKEEALETLQILRRECLTNKPRCAGTSVANEK
CTALELLEQEHTQGFIITFCSALDNILGGGIPLMKTTEVCGVPGVGKTQLCMQLAVDVQIPECFGGVAGEAVFI
DTEGSFMVDRVVSLATACIQHLHLIAGTHTEEEHQKALKDFTLENILSHIYYFRCHDYTELLAQVYLLPDFLSDH
PKVQLVIIDGIAFPFRHDLEDLSLRTRLLNGLAQQMISLANNHRLAVILTNQMTTKIDKNQALLVPALGESWGH
AATIRLIFHWEQKQRFATLYKSPSQKESTIPFQITPQGFRDAVVTAASSQTESSLNFRKRSREPEEEC.

RAD51D GRCm39 – WT (CDS region obtained from m-RNA transcript NM_011235.4)

MGMLRAGLCPGLTEETVQLLRGRKIKTVADLAAADLEEVAQKCGLSYKALVALRRVLLAQFSAFPLNGAD
LYEELKTSTAILSTGIGSLDKLLDAGLYTGEVTEIVGGPGSGKTQVCLCVAANVAHSLQQNVLYVDSNGG
MTASRLLQLLQARTQDEEKQASALQRIQVVRSFDIFRMLDMLQDLRGTIAQQEATSSGAVKVVIVDSVTA
VVAPLLGGQQREGLALMMQLARELKILARDLGVAVVVTNHLTRDWDGRRFKPALGRSWSFVPSTRILLDV
TEGAGTLGSSQRTVCLTKSPRQPTGLQEMIDIGTLGTEEQSPELPGKQT.

855012 Sample Consensus for RAD51D→ No Change

MGMLRAGLCPGLTEETVQLLRGRKIKTVADLAAADLEEVAQKCGLSYKALVALRRVLLAQFSAFPLNGADLYEE
LKTSTAILSTGIGSLDKLLDAGLYTGEVTEIVGGPGSGKTQVCLCVAANVAHSLQQNVLYVDSNGGMTASRLLQ
LLQARTQDEEKQASALQRIQVVRSFDIFRMLDMLQDLRGTIAQQEATSSGAVKVVIVDSVTAVVAPLLGGQQ
REGLALMMQLARELKILARDLGVAVVVTNHLTRDWDGRRFKPALGRSWSFVPSTRILLDVTEGAGTLGSSQR
TVCLTKSPRQPTGLQEMIDIGTLGTEEQSPELPGKQT.

STK11 GRCm39 – WT (CDS region obtained from m-RNA transcript NM_011492.5)

MDVADPEPLGLFSEGELMSVGMDTFIHRIDSTEVIYQPRRKRAKLIGKYLMGDLLGEGSYGKVKEVLDSETLCR
RAVKILKKKKLRRIPNGEANVKKEIQLLRRLRHRNVIQLVDVLYNEEKQKMYMVMEYCVCGMQEMLDSVPEK
RFPVCQAHGYFRQLIDGLEYLHSQGIVHKDIKPGNLLLTTNGTLKISDLGVAEALHPFAVDDTCRTSQGSPAFQ
PPEIANGLDTFSGFKVDIWSAGVTLYNITTGLYPFEGDNIYKLFENIGRGDFTIPCDCGPPLSDLLRGMLEYEPAK
RFSIRQIRQHSWFRKKHPLAEALVPIPPSPDTKDRWRSMTVVPYLEDLHGRAEEEEEEDLFDIEDGIIYTQDFTV
PGQVLEEEVGQNGQSHSLPKAVCVNGTEPQLSSKVKPEGRPGTANPARKVCSSNKIRRLSACKQQ.

855012 Sample Consensus for STK11 → No change

MDVADPEPLGLFSEGELMSVGMDTFIHRIDSTEVIYQPRRKRAKLIGKYLMGDLLGEGSYGKVKEVLDSETLCR
RAVKILKKKKLRRIPNGEANVKKEIQLLRRLRHRNVIQLVDVLYNEEKQKMYMVMEYCVCGMQEMLDSVPEK
RFPVCQAHGYFRQLIDGLEYLHSQGIVHKDIKPGNLLLTTNGTLKISDLGVAEALHPFAVDDTCRTSQGSPAFQ
PPEIANGLDTFSGFKVDIWSAGVTLYNITTGLYPFEGDNIYKLFENIGRGDFTIPCDCGPPLSDLLRGMLEYEPAK
RFSIRQIRQHSWFRKKHPLAEALVPIPPSPDTKDRWRSMTVVPYLEDLHGRAEEEEEEDLFDIEDGIIYTQDFTV
PGQVLEEEVGQNGQSHSLPKAVCVNGTEPQLSSKVKPEGRPGTANPARKVCSSNKIRRLSACKQQ.

TP53 GRCm39 – WT CDS region obtained from m-RNA transcript NM_011640.4)

MTAMEESQSDISLELPLSQETFSGLWKLLPPEDILPSPHCMDDLLLPQDVEEFFEGPSEALRVSGAPAAQDPVT ETPGPVAPAPATPWPLSSFVPSQKTYQGNYGFHLGFLQSGTAKSVMCTYSPPLNKLFCQLAKTCPVQLWVSAT PPAGSRVRAMAIYKKSQHMTEVVRRCPHHERCSDGDGLAPPQHLIRVEGNLYPEYLEDRQTFRHSVVVPYEP PEAGSEYTTIHYKYMCNSSCMGGMNRRPILTIITLEDSSGNLLGRDSFEVRVCACPGRDRRTEEENFRKKEVLC PELPPGSAKRALPTCTSASPPQKKKPLDGEYFTLKIRGRKRFEMFRELNEALELKDAHATEESGDSRAHSSYLKT KKGQSTSRHKKTMVKKVGPDSD.

855012 Sample Consensus for TP53

MTAMEESQSDISLELPLSQETFSGLWKL.

BRCA1_GRCm39 – WT – (Mus Musculus BRCA1 CDS Region obtained from NC_000077.6)

MDLSAVQIQEVQNVLHAMQKILECPICLELIKEPVSTKCDHIFCKFCMLKLLNQKKGPSQCPLCKNEITKRSLQ
GSTRFSQLAEELLRIMAAFELDTGMQLTNGFSFSKKRNNSCERLNEEASIIQSVGYRNRVRRLPQVEPGNATLK
DSLGVQLSNLGIVRSVKKNRQTQPRKKSVYIELDSDSSEETVTKPGDCSVRDQELLQTAPQEAGDEGKLHSAEE
AACEFSEGIRNIEHHQCSDDLNPTENHATERHPEKCQSISISNVCVEPCGTDAHASSLQPETSSLLLIEDRMNAE
KAEFCNKSKQPGIAVSQQSRWAASKGTCNDRQVPSTGEKVGPNADSLSDREKWTHPQSLCPENSGATTDVP
WITLNSSVQKVNEWFSRTGEMLTSDSASARRHESNAEAAVVLEVSNEVDGGFSSSRKTDLVTPDPHHTLMCK
SGRDFSKPVEDNISDKIFGKSYQRKGSRPHLNHVTEIIGTFITEPQITQEQPFTNKLKRKRSTSLQPEDFIKKADS
AGVQRTPDNINQGTDLMEPNEQAVSTTSNCQENKIAGSNLQKEKSAHPTESLRKEPASTAGAKSISNSVSDLE
VELNVHSSKAPKKNRLRRKSSIRCALPLEPISRNPSPPTCAELQIDSCGSSEETKKNHSNQQPAGHLREPQLIED
TEPAADAKKNEPNEHIRKRRASDAFPEEKLMNKAGLLTSCSSPRKSQGPVNPSPQRTGTEQLETRQMSDSAK
ELGDRVLGGEPSGKTTDRSEESTSVSLVSDTDYDTQNSVSVLDAHTVRYARTGSAQCMTQFVASENPKELVHG
SNNAGSGTEGLKPPLRHALNLSQEKVEMEDSELDTQYLQNTFQVSKRQSFALFSKPRSPQKDCAHSVPSKELS
PKVTAKGKQKERQGQEEFEISHVQAVAATVGLPVPCQEVSPIRSSIKTDNRKPLTEGRFERHTSSTEMAVGNE
NILQSTVHTVSLNNRGNACQEAGSGSIHEVCSTGDSFPGQLGRNRGPKVNTVPPLDSMQPGVCQQSVPVSD
KYLEIKKQEGEAVCADFSPCLFSDHLEQSMSGKVFQVCSETPDDLLDDVEIQGHTSFGEGDIMERSAVFNGSIL
RRESSRSPSPVTHASKSQSLHRASRKLESSEESDSTEDEDLPCFQHLLSRISNTPELTRCSSAVTQRMPEKAEGT
QAPWKGSSSDCNNEVIMIEASQEHQFSEDPRCSGSMFSSQHSAAQGSTANANSQDSNFIPPSKQRSHQCG
NEEAFLSDKELISDNEEMATCLEEDNDQEEDSIIPDSEASGYESETNLSEDCSQSDILTTQQRATMKYNLIKLQQ
EMAHLEAVLEQRGNQPSGHSPSLLADPCALEDLPDLEPNMSGAAILTSKNINENPVSQNLKSACDDKFQLQH
LEGPTSGDDESGMGRPSPFKSPLAGSRGSAHGCSRHLQKRNSPSQEELLQPAGSEASSEPHNSTGQSCLPRRE
LEGTPYLGSGISLFSSRDPESESPKEPAHIGTTPASTSALKIPQGQVAFRSAAAAGADKAVVGIVSKIKPELTSSEE
RADRDISMVVSGLTPKEVMTVQKFAEKYRLTLTDAITEETTHVIIKTDAEFVCERTLKYFLGIAGGKWIVSYSWV
VRSIQERRLLNVHEFEVKGDVVTGRNHQGPRRSRESREKLFKGLQVYCCEPFTNMPKDELERMLQLCGASVV
KELPSLTHDTGAHLVVIVQPSAWTEDSNCPDIGQLCKARLVMWDWVLDSLSSYRCRDLDAYLVQNITCDSSEP
QDSND.

855012 Sample Consensus for BRCA1- no change

MDLSAVQIQEVQNVLHAMQKILECPICLELIKEPVSTKCDHIFCKFCMLKLLNQKKGPSQCPLCKNEITKRSLQ
GSTRFSQLAEELLRIMAAFELDTGMQLTNGFSFSKKRNNSCERLNEEASIIQSVGYRNRVRRLPQVEPGNATLK
DSLGVQLSNLGIVRSVKKNRQTQPRKKSVYIELDSDSSEETVTKPGDCSVRDQELLQTAPQEAGDEGKLHSAEE
AACEFSEGIRNIEHHQCSDDLNPTENHATERHPEKCQSISISNVCVEPCGTDAHASSLQPETSSLLLIEDRMNAE
KAEFCNKSKQPGIAVSQQSRWAASKGTCNDRQVPSTGEKVGPNADSLSDREKWTHPQSLCPENSGATTDVP
WITLNSSVQKVNEWFSRTGEMLTSDSASARRHESNAEAAVVLEVSNEVDGGFSSSRKTDLVTPDPHHTLMCK
SGRDFSKPVEDNISDKIFGKSYQRKGSRPHLNHVTEIIGTFITEPQITQEQPFTNKLKRKRSTSLQPEDFIKKADS
AGVQRTPDNINQGTDLMEPNEQAVSTTSNCQENKIAGSNLQKEKSAHPTESLRKEPASTAGAKSISNSVSDLE

VELNVHSSKAPKKNRLRRKSSIRCALPLEPISRNPSPPTCAELQIDSCGSSEETKKNHSNQQPAGHLREPQLIED
TEPAADAKKNEPNEHIRKRRASDAFPEEKLMNKAGLLTSCSSPRKSQGPVNPSPQRTGTEQLETRQMSDSAK
ELGDRVLGGEPSGKTTDRSEESTSVSLVSDTDYDTQNSVSVLDAHTVRYARTGSAQCMTQFVASENPKELVHG
SNNAGSGTEGLKPPLRHALNLSQEKVEMEDSELDTQYLQNTFQVSKRQSFALFSKPRSPQKDCAHSVPSKELS
PKVTAKGKQKERQGQEEFEISHVQAVAATVGLPVPCQEVSPIRSSIKTDNRKPLTEGRFERHTSSTEMAVGNE
NILQSTVHTVSLNNRGNACQEAGSGSIHEVCSTGDSFPGQLGRNRGPKVNTVPPLDSMQPGVCQQSVPVSD
KYLEIKKQEGEAVCADFSPCLFSDHLEQSMSGKVFQVCSETPDDLLDDVEIQGHTSFGEGDIMERSAVFNGSIL
RRESSRSPSPVTHASKSQSLHRASRKLESSEESDSTEDEDLPCFQHLLSRISNTPELTRCSSAVTQRMPEKAEGT
QAPWKGSSSDCNNEVIMIEASQEHQFSEDPRCSGSMFSSQHSAAQGSTANANSQDSNFIPPSKQRSHQCG
NEEAFLSDKELISDNEEMATCLEEDNDQEEDSIIPDSEASGYESETNLSEDCSQSDILTTQQRATMKYNLIKLQQ
EMAHLEAVLEQRGNQPSGHSPSLLADPCALEDLPDLEPNMSGAAILTSKNINENPVSQNLKSACDDKFQLQH
LEGPTSGDDESGMGRPSPFKSPLAGSRGSAHGCSRHLQKRNSPSQEELLQPAGSEASSEPHNSTGQSCLPRRE
LEGTPYLGSGISLFSSRDPESESPKEPAHIGTTPASTSALKIPQGQVAFRSAAAAGADKAVVGIVSKIKPELTSSEE
RADRDISMVVSGLTPKEVMTVQKFAEKYRLTLTDAITEETTHVIIKTDAEFVCERTLKYFLGIAGGKWIVSYSWV
VRSIQERRLLNVHEFEVKGDVVTGRNHQGPRRSRESREKLFKGLQVYCCEPFTNMPKDELERMLQLCGASVV
KELPSLTHDTGAHLVVIVQPSAWTEDSNCPDIGQLCKARLVMWDWVLDSLSSYRCRDLDAYLVQNITCDSSEP
QDSND.

Figure S1: Multiple Sequence Alignment

BARD1

experimental mouse protein has muations that are shown in the rectangular box and eventually truncated

```
H_sapienBARD1.              MPDNRQPRNRQPRIRSGNEPRSAPAMEPDGRGAWAHSRAALDRLEKLLRCSRCTNILREP    60
M_musculusBARD1.            ------MPRRPPRVCSGNQPAPVPAMEPATDGLWAHSRAALARLEKLLRCSRCANILKEP    54
mutant_M_musculusBARD1.     ------MPRRPPRVCSGNQPAPVPAMEPATDGLWAHSRAALARLEKLLRCSRCANILKEP    54
                                    .* **: ***:*  .*****   * ******** ***********:***:**

H_sapienBARD1.              VCLGGCEHIFCSNCVSDCIGTGCPVCYTPAWIQDLKINRQLDSMIQLCSKLRNLLHDNEL    120
M_musculusBARD1.            VCLGGCEHIFCSGCISDCVGSGCPVCYTPAWILDLKINRQLDSMIQLSSKLQNLLHDNKD    114
mutant_M_musculusBARD1.     VCLGGCEHIFCSGCISDCVGSGCPVCYTPAWILDLKINRQLDSMIQLSSKLQNLLHDNKD    114
                            ************.*:***:*:*********** **************.***:******:

H_sapienBARD1.              SDLKEDKPRKSLFNDAGNKKNSIKMWFSPRSKKVRYVVSKASVQTQPAIKKDASAQ-QDS    179
M_musculusBARD1.            --SKDNTSRASLFGDAERKKNSIKMWFSPRSKKVRYVVTKVSVQTQPQKAKDDKAQEASM    172
mutant_M_musculusBARD1.     --SKDNTSRASLFGDAERKKNSIKMWFSPRSKKVRYVVTKVSVQTQPQKAKDDKAQEASM    172
                               *::. * ***.** .********************:*.******   ** .**  .

H_sapienBARD1.              YEFVSPSPPADVSERAKKASARSGKKQKKKTLAEINQKWNLEAEKEDGEFDSKEESKQKL    239
M_musculusBARD1.            YEFVSATPPVAVPKSAKTASRTSAKKHPKKSVAKINREENLRPETKDSRFDSKEELKEEK    232
mutant_M_musculusBARD1.     YEFVSATPPVAVPKSAKTASRTSAKKHPKKSVAKINREENLRPETKDSRFDSKEELEGLP    232
                            ***** :**. * : **.**  *.**: **::*:**:: **. *.:*..****** :

H_sapienBARD1.              VSFCSQPSVISSPQINGEIDLLASGSLTESECFGSLTEVSLPLAEQIESPDTKSRNEVVT    299
M_musculusBARD1.            VVSCSQIPVMERPRVNGEIDLLASGSVVEPECSGSLTEVSLPLAEHIVSPDTVSKNEETP    292
mutant_M_musculusBARD1.     LQPTTSK-----------------------------------------------------    239
                            :   :.
```

BRCA2

experimental mouse protein has muations that are shown in the rectangular box

```
human_BRCA2                       MPIGSKERPTFFEIFKTRCNKADLGPISLNWFEELSSEAPPYNSEPAEESEHKNNNYEPN   60
wildtype_mus_musculus_BRCA2       MPVEYKRRPTFWEIFKARCSTADLGPISLNWFEELSSEAPPYNSEPPEESEYKPHGYEPQ   60
mutant_mus                        MPVEYKRRPTFWEIFKARCSTADLGPISLNWFEELSSEAPPYNSEPPEESEYKPHGYEPQ   60
                                  **:  *.****:****:**..************************* ****:* :.***:

human_BRCA2                       LFKTPQRKPSYNQLASTPIIFKEQGLTLPLYQSPVKELDKFKLDLGRNVPNSRHKSLRTV   120
wildtype_mus_musculus_BRCA2       LFKTPQRNPPYHQFASTPIMFKERSQTLPLDQSPFRELGK-------VVASSKHKTHSKK   113
mutant_mus                        LFKTPQRNPPYHQFASTPIMFKERSQTLPLDQSPFRELGK-------VVASSKHKTHSKK   113
                                  *******:* *:*:*****:***:. **** ***.:**.*        * .*:**:  .

human_BRCA2                       KTKMDQADDVSCPLLNSCLSESPVVLQCTHVTPQRDKSVVCGSLFHTPKFVKGRQTPKHI   180
wildtype_mus_musculus_BRCA2       KTKVDPVVDVASPPLKSCLSESPLTLRCTQAVLQREKPVVSGSLFYTPKLKEG-QTPKPI   172
mutant_mus                        KTKVDPVVDVASPPLKSCLSESPLTLRCTQAVLQREKPVVSGSLFYTPKLKEG-QTPKPI   172
                                  ***:* . **:.* *:*******:.*:**:.. **:* **.****:***: :* **** *

human_BRCA2                       SESLGAEVDPDMSWSSSLATPPTLSSTVLIVRNEEASETVFPHDTTANVKSYFSNHDESL   240
wildtype_mus_musculus_BRCA2       SESLGVEVDPDMSWTSSLATPPTLSSTVLIARDEEARSSVTPADSPATLKSCFSNHNESP   232
mutant_mus                        SESLGVEVDPDMSWTSSLATPPTLSSTVLIARDEEARSSVTPADSPATLKSCFSNHNESP   232
                                  *****.********:***************.*:*** .:* * *: *.:** ****:**

human_BRCA2                       KKNDRFIASVTDSENTNQREAASHGFGKTSGNSF-KVNSCKDHIGKSMPNVLEDEVYETV   299
wildtype_mus_musculus_BRCA2       QKNDRSVPSVIDSENKNQQEAFSQGLGKMLGDSSGKRNSFKDCLRKPIPNILEDG--ETA   290
mutant_mus                        QKNDRSVPSVIDSENKNQQEAFSQGLGKMLGDSSGKRNSFKDCLRKPIPNILEDG--ETA   290
                                  :**** : ** ****.**:** *:*:**  *:*  *:*  * ** ** : * :**:***   **.
```

```
human_BRCA2                      VDTSEEDSFSLCFSKCRTKNLQKVRTSKTRKKIFHEANADECE-KSKNQVKEKYSFVSEV    358
wildtype_mus_musculus_BRCA2      VDTSEEDSFSLCFPKRRTRNLQKMRMGKTRKKIFSETRTDELSEEARRQTDDKNSFVFEM    350
mutant_mus                       VDTSEEDSFSLCFPKRRTRNLQKMRMGKTRKKIFSETRTDELSEEARRQTDDKNSFVFEM    350
                                 ************* * **:****:* .******* *:.:** . :::.*..:* *** *:

human_BRCA2                      EPNDTDPLDSNVAHQKPFESGSDKISKEVVPSLACEWSQLTLSGLNGAQMEKIPLLHISS    418
wildtype_mus_musculus_BRCA2      ELRESDPLDPGVTSQKPFYSQNEEICNEAVQCSDSRWSQSNLSGLNETQTGKITLPHISS    410
mutant_mus                       ELRESDPLDPGVTSQKPFYSQNEEICNEAVQCSDSRWSQSNLSGLNETQTGKITLPHISS    410
                                 * .::**** .*: **** * .::*.:*.* .  ..*** .***** :*  ** * ****

human_BRCA2                      CDQNISEKDLLDTENKRKKDFLTSENSLPRISSLPKSEKPLNEETVVNKRDEEQHLESHT    478
wildtype_mus_musculus_BRCA2      HSQNISEDFI-DM-KKEGTGSITSEKSLPHISSLPEPEKMFSEETVVDKEHEGQHFESLE    468
mutant_mus                       HSQNISEDFI-DM-KKEGTGSITSEKSLPHISSLPEPEKMFSEETVVDKEHEGQHFESLE    468
                                  .*****. : *  :*. .. :***:***:*****: ** :.*****:*..* **:**

human_BRCA2                      DCILAVKQAISGTSPVASSFQGIKKSIFRIRESPKETFNASFSGHMTDPNFKKETEASES    538
wildtype_mus_musculus_BRCA2      DSIAG-KQMVSRTSQAACLSPSIRKSIFKMREPLDETLGTVFSDSMTNSTFTEEHEASAC    527
mutant_mus                       DSIAG-KQMVSRTSQAACLSPSIRKSIFKMREPLDETLGTVFSDSMTNSTFTEEHEASAC    527
                                 *.* . ** :* ** .*.   .*:****::**  .**:.: **. **: .*.:* *** .

human_BRCA2                      GLEIHTVCSQKEDSLCPNLIDNGSWPATTTQNSVALKNAGLISTLKKKTNKFIYAIHDET    598
wildtype_mus_musculus_BRCA2      GLGILTACSQREDSICPSSVDTGSWPTTLTDTSATVKNAGLISTLKNKKRKFIYSVSDDA    587
mutant_mus                       GLGILTACSQREDSICPSSVDTGSWPTTLTDTSATVKNAGLISTLKNKKRKFIYSVSDDA    587
                                 ** * *.***:***:**. :*.****:* *:.*.::**********:*..****:: *::
```

```
human_BRCA2                      FYKGKKIPKDQKSELINCSAQFEANAFEAPLTFANADSGLLHSSVKRSCSQNDSEEPTLS    658
wildtype_mus_musculus_BRCA2      SLQGKKLQTHRQLELTNLSAQLEASAFEVPLTFTNVNSGIPDSSDKKRCLPNDPEEPSLT    647
mutant_mus                       SLQGKKLQTHRQLELTNLSAQLEASAFEVPLTFTNVNSGIPDSSDKKRCLPNDPEEPSLT    647
                                   :***: ..:: ** * ***:**.***.****:*.:**: .** *: *  ** ***:*:

human_BRCA2                      LTSSFGTILRKCSRNETCSNNTVISQDLDYKEAKCNKEKLQLFITPEADSLSCLQEGQCE    718
wildtype_mus_musculus_BRCA2      --NSFGTATS----KEISYIHALISQDLNDKEAIVIEEKPQPYTAREADFLLCLPERTCE    701
mutant_mus                       --NSFGTATS----KEISYIHALISQDLNDKEAIVIEEKPQPYTAREADFLLCLPERTCE    701
                                   .****       :* .  :::*****: ***   :** * : : *** * ** *  **

human_BRCA2                      NDPKSKKVSDIKEEVLAAACHPVQHSKVEYSDTDFQSQKSLLYDHENASTLILTPTSKDV    778
wildtype_mus_musculus_BRCA2      NDQKSPKVSNGKEKVLVSACLPSA---VQLSSISFESQENPLGDHNGTSTLKLTPSSKLP    758
mutant_mus                       NDQKSPKVSNGKEKVLVSACLPSA---VQLSSISFESQENPLGDHNGTSTLKLTPSSKLP    758
                                 ** ** ***: **:**.:** *     *: *. .*:**:. * **:.:*** ***:**

human_BRCA2                      LSNLVMISRGKESYKMSDKLKGNNYESDVELTKNIPMEKNQDVCALNENYKNVELLPPEK    838
wildtype_mus_musculus_BRCA2      LSKADMVSREK-MCKMPEKLQCESCKVNIELSKNIL--EVNEICILSENSKTPGLLPPGE    815
mutant_mus                       LSKADMVSREK-MCKMPEKLQCESCKVNIELSKNIL--EVNEICILSENSKTPGLLPPGE    815
                                 **:  *:** *   ** :**: :. : ::**:***   : :::* *.** *.  **** :

human_BRCA2                      YMRVASPSRKVQFNQNTNLRVIQKNQEETTSISKITVNPDSEELFSDNENNFVFQVANER    898
wildtype_mus_musculus_BRCA2      NIIEVASSMKSQFNQNAKI-VIQKDQKGSPFISEVAVNMNSEELFPDSGNNFAFQVTNKC    874
mutant_mus                       NIIEVASSMKSQFNQNAKI-VIQKDQKGSPFISEVAVNMNSEELFPDSGNNFAFQVTNKC    874
                                  :  .: * * *****::: ****:*: :  **:::** :***** *. ***.***:*:
```

```
human_BRCA2                      NNLALGNTKELHETDLTCVNEPIFKNSTMVLYGDTGDKQATQVSIKKDLV-----YVLAE   953
wildtype_mus_musculus_BRCA2      NKPDLGSSVELQEEDLSHTQGPSLKNSPMAVDEDVDDAHAAQVLITKDSDSLAVVHDYTE   934
mutant_mus                       NKPDLGSSVELQEEDLSHTQGPSLKNSPMAVDEDVDDAHAAQVLITKDSDSLAVVHDYTE   934
                                 *:  **.: **:* **: .: * :*** *.:  *..* :*:** *.**       :  :*

human_BRCA2                      ENKNSVKQHIKMTLGQDLKSDISLNIDKIPEKNNDYMNKWAGLLGPISNHSFGGSFRTAS   1013
wildtype_mus_musculus_BRCA2      KSRNNIEQHQKGTEDKDFKSNSSLNMK--SDGNSDCSDKWSEFLDPVLNHNFGGSFRTAS   992
mutant_mus                       KSRNNIEQHQKGTEDKDFKSNSSLNMK--SDGNSDCSDKWSEFLDPVLNHNFGGSFRTAS   992
                                 :.:*.::** * * .:*:**: ***:.   : *.*  :**: :*.*: **.*********

human_BRCA2                      NKEIKLSEHNIKKSKMFFKDIEEQYPTSLACVEIVNTLALDNQKKLSKPQSINTVSAHLQ   1073
wildtype_mus_musculus_BRCA2      NKEIKLSEHNVKKSKMFFKDIEEQYPTRLACIDIVNTLPLANQKKLSEPHIFDLKSVTTV   1052
mutant_mus                       NKEIKLSEHNVKKSKMFFKDIEEQYPTRLACIDIVNTLPLANQKKLSEPHIFDLKSVTTV   1052
                                 **********:**************** ***::***** * ******:*: ::  *.

human_BRCA2                      SSV----VVSDCKNSHITPQMLFSKQDFNSNHNLTPSQKAEITELSTILEESGSQFEFTQ   1129
wildtype_mus_musculus_BRCA2      STQSHNQSSVSHEDTDTAPQMLSSKQDFHSN-NLTTSQKAEITELSTILEESGSQFEFTQ   1111
mutant_mus                       STQSHNQSSVSHEDTDTAPQMLSSKQDFHSN-NLTTSQKAEITELSTILEESGSQFEFTQ   1111
                                 *:        . :::. :**** *****:** *** ************************

human_BRCA2                      FRKPSYILQKSTFEVPENQMTILKTTSEECRDADLHVIMNAPSIGQVDSSKQFEGTVEIK   1189
wildtype_mus_musculus_BRCA2      FRKPSHIAQ-NTSEVPGNQMVVLSTASKEWKDTDLHLPV-DPSVGQTDHSKQFEGSAGVK   1169
mutant_mus                       FRKPSHIAQ-NTSEVPGNQMVVLSTASKEWKDTDLHLPV-DPSVGQTDHSKQFEGSAGVK   1169
                                 *****:* * .* *** ***.:*.*:*:* :*:***: :  **:**.* ******:. :*
```

```
human_BRCA2                     RKFAGLLKNDCNKSASGYLTDENEVGFRGFYSAHGTKLNVSTEALQKAVKLFSDIENISE   1249
wildtype_mus_musculus_BRCA2     QSFPHLLEDTCNKNTSCFLPNINEMEFGGFCSALGTKLSVSNEALRKAMKLFSDIENS-E   1228
mutant_mus                      QSFPHLLEDTCNKNTSCFLPNINEMEFGGFCSALGTKLSVSNEALRKAMKLFSDIENS-E   1228
                                :.*  **:: ***.:* :* : **: * ** ** ****.**.***:**:********  *

human_BRCA2                     ETSAEVHPISLSSSKCHDSVVSMFKIENHN-DKTVSEKNNKCQLILQNNIEMTTGTFVEE   1308
wildtype_mus_musculus_BRCA2     EPSAKVGPRGFSSSAHHDSVASVFKIKKQNTEKSFDEKSSKCQVTLQNNIEMTTCIFVGR   1288
mutant_mus                      EPSAKVGPRGFSSSAHHDSVASVFKIKKQNTEKSFDEKSSKCQVTLQNNIEMTTCIFVGR   1288
                                * **:* * .:***  ****.*:***::*  :*:..**..***: *********  ** .

human_BRCA2                     ITENYKRNTENEDNKYTAASRNSHNLEFDGSDSSKNDTVCIHKDETDLLFTDQHNICLKL   1368
wildtype_mus_musculus_BRCA2     NPEKYIKNTKHEDSY-TSSQRNN-LENSDGSMSSTSGPVYIHKGDSDLP-ADQGSKCPES   1345
mutant_mus                      NPEKYIKNTKHEDSY-TSSQRNN-LENSDGSMSSTSGPVYIHKGDSDLP-ADQGSKCPES   1345
                                  *:* :**::**.  *::.**.   : *** **... * ***.::**  :** . * :

human_BRCA2                     SGQFMKEGNTQIKEDLSDLTFLEVAKAQEACHGNTSNKEQLTATKTEQNIKDFETSDTFF   1428
wildtype_mus_musculus_BRCA2     CTQYAREENTQIKENISDLTCLEIMKAEETCMKSS-DKKQLPSDKMEQNIKEFNI---SF   1401
mutant_mus                      CTQYAREENTQIKENISDLTCLEIMKAEETCMKSS-DKKQLPSDKMEQNIKEFNI---SF   1401
                                . *: :* ******::**** **: **:*:*  .: :*:** : * *****:*:     *

human_BRCA2                     QTASGKNISVAKESFNKIVNFFDQKPEELHNFS--LNSELHSDIRKNKMDILSYEETDIV   1486
wildtype_mus_musculus_BRCA2     QTASGKNTRVSKESLNKSVNIFNRETDELTVISDSLNSKILHGINKDKMHTSC-HKKAIS   1460
mutant_mus                      QTASGKNTRVSKESLNKSVNIFNRETDELTVISDSLNSKILHGINKDKMHTSC-HKKAIS   1460
                                *******  *:***:** **:*::: :**  :*  ***::  .*.*:**.  . .:. *
```

```
human_BRCA2                    KHKILKESVPVGTGNQLVTFQGQPERDEKIKEPTLLGFHTASGKKVKIAKESLDKVKNLF  1546
wildtype_mus_musculus_BRCA2    IKKVFEDHFPIVTVSQLPAQQHPEYEIESTKEPTLLSFHTASGKKVKIMQESLDKVKNLF  1520
mutant_mus                     IKKVFEDHFPIVTVSQLPAQQHPEYEIESTKEPTLLSFHTASGKKVKIMQESLDKVKNLF  1520
                                :*:::: .*: * .** : *    . *. ******.*********** :**********

human_BRCA2                    DEKEQGTSEITSFSHQWAKTLKYREACKDLELACETIEITAAPKCKEMQNSLNNDKNLVS  1606
wildtype_mus_musculus_BRCA2    DETQYVRK-TASFSQG---SKPLKDSKKELTLAYEKIEVT-ASKCEEMQNFVSKETEMLP  1575
mutant_mus                     DETQYVRK-TASFSQG---SKPLKDSKKELTLAYEKIEVT-ASKCEEMQNFVSKETEMLP  1575
                               **.:   .  :***:    :   ::: *:* ** *.**:* * **:**** :.::.:::

human_BRCA2                    IETVVPPKLLSDNLCRQTENLKTSKSIFLKVKVHENVEKETAKSPATCYTNQSPYSVIEN  1666
wildtype_mus_musculus_BRCA2    QQ--------NYHMYRQTENLKTSNGT--SSKVQENIENNVEKNPRICCICQSSYPVTED  1625
mutant_mus                     QQ--------NYHMYRQTENLKTSNGT--SSKVQENIENNVEKNPRICCICQSSYPVTED  1625
                                :        . :: *********:.    .**:**:*::. *.*  *   ** * * *:

human_BRCA2                    SALAFYTSCSRKTSVSQTSLLEAKKWLREGIFDG-Q-------PERINTADYVGNYLYEN  1718
wildtype_mus_musculus_BRCA2    SALAYYTEDSRKTCVRESSLSKGRKWLREQGDKLGTRNTIKIECVKEHTEDFAGNASYEH  1685
mutant_mus                     SALAYYTEDSRKTCVRESSLSKGRKWLREQGDKLGTRNTIKIECVKEHTEDFAGNASYEH  1685
                               ****:**. ****.* ::** :.:*****   .            : :* *:.**  **:

human_BRCA2                    NSNSTIAENDKNHLSEKQDTY-L-----SNSSMSNSYSYHSDEVYNDSGYLSKNKLDSGI  1772
wildtype_mus_musculus_BRCA2    SLVIIRTEIDTNHVSENQVSTLLSDPNVCHSYLSQSSFCHCDDMHNDSGYFLKNKIDSDV  1745
mutant_mus                     SLVIIRTEIDTNHVSENQVSTLLSDPNVCHSYLSQSSFCHCDDMHNDSGYFLKNKIDSDV  1745
                               .     :* *.**:**:* :  *     .:* :*:*   *.*:::*****: ***:**.:
```

```
human_BRCA2                    EPVLKNVEDQKNTSFSKVISNVKDANAYPQTVNEDICVEELVTSSSPCKNKNAAIKLSIS 1832
wildtype_mus_musculus_BRCA2    PPDMKNAEGNT---ISPRVSATKERNLHPQTIN-EYCVQKLETNTSPHANKDVAIDPSLL 1801
mutant_mus                     PPDMKNAEGNT---ISPRVSATKERNLHPQTIN-EYCVQKLETNTSPHANKDVAIDPSLL 1801
                                * :**.*.:.   :*  :* .*: * :***:* : **::* *.:**  **:.**. *:

human_BRCA2                    NSNNFEVGPPAFRIASGKIVCVSHETIKKVKDIFTDSFSKVIKENNENKSKICQTKIMAG 1892
wildtype_mus_musculus_BRCA2    DSRNCKVGSLVFITAHS-------QETERTKEIVTDNCYKIVEQNRQSKPDTCQT----S 1850
mutant_mus                     DSRNCKVGSLVFITAHS-------QETERTKEIVTDNCYKIVEQNRQSKPDTCQT----S 1850
                               :*.* :**  .*  * .       :  ::.*:*.**.  *::::*.:.* . ***    .

human_BRCA2                    CYEALDDSEDILHNSLDNDECSTHSHKVFADIQSEEILQHNQNMSGLEKVSKISPCDVSL 1952
wildtype_mus_musculus_BRCA2    CHKVLDDSKDFICPSSSGDVCINSRKDSFCP-HNEQILQHNQSMSGLKKAATPP---VGL 1906
mutant_mus                     CHKVLDDSKDFICPSSSGDVCINSRKDSFCP-HNEQILQHNQSMSGLKKAATPP---VGL 1906
                               *::.****:*::  * ..* * .  :. *.  :.*:******.****:*.:.     *.*

human_BRCA2                    ETSDICKCSIGKLHKSVSSANTCGIFSTASGKSVQVSDASLQNARQVFSEIEDSTKQVFS 2012
wildtype_mus_musculus_BRCA2    ETWDTSK-SIREPPQAAHPSRTYGIFSTASGKAIQVSDASLEKARQVFSEMDGDAKQLSS 1965
mutant_mus                     ETWDTSK-SIREPPQAAHPSRTYGIFSTASGKAIQVSDASLEKARQVFSEMDGDAKQLSS 1965
                               ** * .* ** :  ::.  :.* *********::*******::*******::..:**: *

human_BRCA2                    KVLFKSNEHSDQLTREENTAIRTPEHLISQKGFSYNVVNSSAFSGFSTASGKQVSILESS 2072
wildtype_mus_musculus_BRCA2    MVSLEGNEKPHHSVKRENSVVHSTQGVLSLPKPLPGNVNSSVFSGFSTAGGKLVTVSESA 2025
mutant_mus                     MVSLEGNEKPHHSVKRENSVVHSTQGVLSLPKPLPGNVNSSVFSGFSTAGGKLVTVSESA 2025
                                * ::.**: .: .:.**:.::: : ::*      . ****.*******.** *:: **:
```

```
human_BRCA2                     LHKVKGVLEEFDLIRTEHSLHYSPTSRQNVSKILPR--VDKRNPEHCVNSEMEKTCSKEF   2130
wildtype_mus_musculus_BRCA2     LHKVKGMLEEFDLIRTEHTLQHSPIPE-DVSKILPQPCAEIRTPEYPVNSKLQKTYNDKS   2084
mutant_mus                      LHKVKGMLEEFDLIRTEHTLQHSPIPE-DVSKILPQPCAEIRTPEYPVNSKLQKTYNDKS   2084
                                ******:***********:*::**  . :******:  .: *.**: ***:::** ..: 

human_BRCA2                     KLSNNLNVEGGSSENNHSIKVSPYLSQFQQD-KQQLVLGTKVSLVENIHVLGKEQASPKN   2189
wildtype_mus_musculus_BRCA2     SLPSNYK-ESGSSGNTQSIEVSLQLSQMERNQDTQLVLGTKVSH-SKANLLGKEQTLPQN   2142
mutant_mus                      SLPSNYK-ESGSSGNTQSIEVSLQLSQMERNQDTQLVLGTKVSH-SKANLLGKEQTLPQN   2142
                                .* .* : *.*** *.:**:**  ***:::: . *********  .: ::*****: *:*

human_BRCA2                     VKMEIGKTETFSDVPVKTNIEVCSTYSKDSENYFETEAVEIAKAFMEDDELTDSKLPSHA   2249
wildtype_mus_musculus_BRCA2     IKVKTDEMKTFSDVPVKTNVG--EYYSKESENYFETEAVESAKAFMEDDELTDSEQ-THA   2199
mutant_mus                      IKVKTDEMKTFSDVPVKTNVG--EYYSKESENYFETEAVESAKAFMEDDELTDSEQ-THA   2199
                                :*:: .: :**********:   . ***:*********** *************:  :**

human_BRCA2                     THSLFTCPENEEMVLSNSRIGKRRGEPLILVGEPSIKRNLLNEFDRIIENQEKSLKASKS   2309
wildtype_mus_musculus_BRCA2     KCSLFTCPQNETLF--NSRTRKRGGVTVDAVGQPPIKRSLLNEFDRIIESKGKSLTPSKS   2257
mutant_mus                      KCSLFTCPQNETLF--NSRTRKRGGVTVDAVGQPPIKRSLLNEFDRIIESKGKSLTPSKS   2257
                                . ******:** :.  ***  ** *  :  **:* ***.**********.: ***. ***

human_BRCA2                     TPDGTIKDRRLFMHHVSLEPITCVPFRTTKERQEIQNPNFTAPGQEFLSKSHLYEHLTLE   2369
wildtype_mus_musculus_BRCA2     TPDGTVKDRSLFTHHMSLEPVTCGPFCSSKERQGAQRPHLTSPAQELLSKGHPWRHSALE   2317
mutant_mus                      TPDGTVKDRSLFTHHMSLEPVTCGPFCSSKERQGAQRPHLTSPAQELLSKGHPWRHSALE   2317
                                *****:*** ** **:****:** ** ::****  *.*::*:*.**:***.* :.* :**
```

```
human_BRCA2                     KSSSNLAVSGHPFYQVSATRNEKMRHLITTGRPTKVFVPPFKTKSHFHRVEQCVR-NINL   2428
wildtype_mus_musculus_BRCA2     KSPSSPIVSILPAHDVSATRTERTRH---SGKSTKVFVPPFKMKSQFHGDEHFNSKNVNL   2374
mutant_mus                      KSPSSPIVSILPAHDVSATRTERTRH---SGKSTKVFVPPFKMKSQFHGDEHFNSKNVNL   2374
                                ** *.  **  * ::*****.*: **   :*: ********* **:**  *:    *:**

human_BRCA2                     EENRQKQNIDGHGSDDSKNKINDNEIHQFNKNNSNQAAAVTFTKCEEEPLDLITSLQNAR   2488
wildtype_mus_musculus_BRCA2     EGKNQK-STDGDR-----EDGNDSHVRQF-------------------NKDLMSSLQSAR   2409
mutant_mus                      EGKNQK-STDGDR-----EDGNDSHVRQF-------------------NKDLMSSLQSAR   2409
                                * :.** . **.      :. **..::**                     **::***.**

human_BRCA2                     DIQDMRIKKKQRQRVFPQPGSLYLAKTSTLPRISLKAAVGGQVPSACSHKQLYTYGVSKH   2548
wildtype_mus_musculus_BRCA2     DLQDMRIKNKERRHLRLQPGSLYLTKSSTLPRISLQAAVGDRAPSACSPKQLYIYGVSKE   2469
mutant_mus                      DLQDMRIKNKERRHLRLQPGSLYLTKSSTLPRISLQAAVGDRAPSACSPKQLYIYGVSKE   2469
                                *:******:*:*:::  *******:*:********:****.:.***** **** *****.

human_BRCA2                     CIKINSKNAESFQFHTEDYFGKESLWTGKGIQLADGGWLIPSNDGKAGKEEFYRALCDTP   2608
wildtype_mus_musculus_BRCA2     CINVNSKNAEYFQFDIQDHFGKEDLCAGKGFQLADGGWLIPSNDGKAGKEEFYRALCDTP   2529
mutant_mus                      CINVNSKNAEYFQFDIQDHFGKEDLCAGKGFQLADGGWLIPSNDGKAGKEEFYRALCDTP   2529
                                **::****** ***. :*:****.* :***:*****************************

human_BRCA2                     GVDPKLISRIWVYNHYRWIIWKLAAMECAFPKEFANRCLSPERVLLQLKYRYDTEIDRSR   2668
wildtype_mus_musculus_BRCA2     GVDPKLISSIWVANHYRWIVWKLAAMEFAFPKEFANRCLNPERVLLQLKYRYDVEIDNSR   2589
mutant_mus                      GVDPKLISSIWVANHYRWIVWKLAAMEFAFPKEFANRCLNPERVLLQLKYRYDVEIDNSR   2589
                                ******** *** ******:******* ***********.*************.***.**
```

```
human_BRCA2                    RSAIKKIMERDDTAAKTLVLCVSDIISLSANISETSSNKTSSADTQKVAIIELTDGWYAV  2728
wildtype_mus_musculus_BRCA2    RSALKKILERDDTAAKTLVLCISDIISPSTKVSETSGGKTSGEDANKVDTIELTDGWYAV  2649
mutant_mus                     RSALKKILERDDTAAKTLVLCISDIISPSTKVSETSGGKTSGEDANKVDTIELTDGWYAV  2649
                               ***:***:*************:***** *:::****..***. *::**  **********

human_BRCA2                    KAQLDPPLLAVLKNGRLTVGQKIILHGAELVGSPDACTPLEAPESLMLKISANSTRPARW  2788
wildtype_mus_musculus_BRCA2    RAQLDPPLMALVKSGKLTVGQKIITQGAELVGSPDACAPLEAPDSLRLKISANSTRPARW  2709
mutant_mus                     RAQLDPPLMALVKSGKLTVGQKIITQGAELVGSPDACAPLEAPDSLRLKISANSTRPARW  2709
                               :*******:*::*.*:******** :***********:*****:** *************

human_BRCA2                    YTKLGFFPDPRPFPLPLSSLFSDGGNVGCVDVIIQRAYPIQWMEKTSSGLYIFRNEREEE  2848
wildtype_mus_musculus_BRCA2    HSRLGFFRDPRPFPLPLSSLFSDGGNVGCVDIIVQRVYPLQWVEKTVSGLYIFRSEREEE  2769
mutant_mus                     HSRLGFFRDPRPFPLPLSSLFSDGGNVGCVDIIVQRVYPLQWVEKTVSGLYIFRSEREEE  2769
                               :::**** ***********************:*:**.**:**:*** *******.*****

human_BRCA2                    KEAAKYVEAQQKRLEALFTKIQEEFEEHEENTTKPYLPSRALTRQQVRALQDGAELYEAV  2908
wildtype_mus_musculus_BRCA2    KEALRFAEAQQKKLEALFTKVHTEFKDHEEDTTQRCVLSRTLTRQQVHALQDGAELYAAV  2829
mutant_mus                     KEALRFAEAQQKKLEALFTKVHTEFKDHEEDTTQRCVLSRTLTRQQVHALQDGAELYAAV  2829
                               *** ::.*****:*******:: **::***:**:  : **:******:********* **

human_BRCA2                    KNAADPAYLEGYFSEEQLRALNNHRQMLNDKKQAQIQLEIRKAMESAEQKEQGLSRDVTT  2968
wildtype_mus_musculus_BRCA2    QYASDPDHLEACFSEEQLRALNNYRQMLNDKKQARIQSEFRKALESAE-KEEGLSRDVTT  2888
mutant_mus                     QYASDPDHLEACFSEEQLRALNNYRQMLNDKKQARIQSEFRKALESAE-KEEGLSRDVTT  2888
                               : *:** :**. ***********:**********:** *:***:**** **:********
```

```
human_BRCA2                       VWKLRIVSYSKKEKDSVILSIWRPSSDLYSLLTEGKRYRIYHLATSKSKSKSERANIQLA   3028
wildtype_mus_musculus_BRCA2       VWKLRVTSYKKKEK-SALLSIWRPSSDLSSLLTEGKRYRIYHLAVSKSKSKFERPSIQLT   2947
mutant_mus                        VWKLRVTSYKKKEK-SALLSIWRPSSDLSSLLTEGKRYRIYHLAVSKSKSKFERPSIQLT   2947
                                  *****:.**.**** *.:********** ***************.****** ** .***:

human_BRCA2                       ATKKTQYQQLPVSDEILFQIYQPREPLHFSKFLDPDFQPSCSEVDLIGFVVSVVKKTGLA   3088
wildtype_mus_musculus_BRCA2       ATKRTQYQQLPVSSETLLQVYQPRESLHFSRLSDPAFQPPCSEVDVVGVVVSVVKPIGLA   3007
mutant_mus                        ATKRTQYQQLPVSSETLLQVYQPRESLHFSRLSDPAFQPPCSEVDVVGVVVSVVKPIGLA   3007
                                  ***:*********.* *:*:***** ****:: ** *** *****::*.******  ***

human_BRCA2                       PFVYLSDECYNLLAIKFWIDLNEDIIKPHMLIAASNLQWRPESKSGLLTLFAGDFSVFSA   3148
wildtype_mus_musculus_BRCA2       PLVYLSDECLNLLVVKFGIDLNEDI-KPRVLIAASNLQCQPESTSGVPTLFAGHFSIFSA   3066
mutant_mus                        PLVYLSDECLNLLVVKFGIDLNEDI-KPRVLIAASNLQCQPESTSGVPTLFACHFSIFSA   3066
                                  *:******* ***.:** ******* **::******** :***.**: **** .**:***

human_BRCA2                       SPKEGHFQETFNKMKNTVENIDILCNEAENKLMHILHANDPKWSTPTKDCTSGPYTA---   3205
wildtype_mus_musculus_BRCA2       SPKEAYFQEKVNNLKHAIENIDTFYKEAEKKLIHVLEGDSPKWSTPNKDPTREPHAASTC   3126
mutant_mus                        SPKEAYFQEKVNNLKHAIENIDTFYKEAEKKLIHVLEGDSPKWSTPNKDPTREPHAASTC   3126
                                  ****.:***..*::*::**** : :***:**:*:*..:.******.** *  *::*

human_BRCA2                       --QIIPGTGNKLLMSSPNCEIYYQSPLSLCMAKRKSVSTPVSAQMTSKSCKGEKEIDDQK   3263
wildtype_mus_musculus_BRCA2       CASDLLGSGGQFLRISPTGQQSYQSPLSHCTLKGKSMPLAHSAQMAAKSWSGENEIDDPK   3186
mutant_mus                        CASDLLGSGGQFLRISPTGQQSYQSPLSHCTLKGKSMPLAHSAQMAAKSWSGENEIDDPK   3186
                                    . : *:*.::*  **. :  ****** *  * **:    ****::** .**:**** *
```

```
human_BRCA2                      NCKKRRALDFLSRLPLPPPVSPICTFVSPAAQKAFQPPRSCGTKYETPIKKKELNSPQMT   3323
wildtype_mus_musculus_BRCA2      TCRKRRALDFLSRLPLPSPVSPICTFVSPAAQKAFQPPRSCGTKYATPIKKEPSSPRRRT   3246
mutant_mus                       TCRKRRALDFLSRLPLPSPVSPICTFVSPAAQKAFQPPRSCGTKYATPIKKEPSSPRRRT   3246
                                 .*:************** *************************** *****:  .  : *

human_BRCA2                      PFKKFNEISLLESNSIADEELALINTQALLSGSTGEKQFISVSESTRTAPTSSEDYLRLK   3383
wildtype_mus_musculus_BRCA2      PFQKTSGVSLPDCDSVADEELALLSTQALTPDSVGGNEQAFPGDSTRNPQPAQRPDQQVG   3306
mutant_mus                       PFQKTSGVSLPDCDSVADEELALLSTQALTPDSVGGNEQAFPGDSTRNPQPAQRPDQQVG   3306
                                 **:* . :** :.:*:*******:.****  .*.* ::    .:***.   :..   ::

human_BRCA2                      RRCTTSLIKEQESSQASTEECEKNKQDTITTKKYI    3418
wildtype_mus_musculus_BRCA2      PRS----------RKESLRDCRGDSSEKLAVES--    3329
mutant_mus                       PRS----------RKESLRDCRGDSSEKLAVES--    3329
                                  *.           : * .:*. :..:.::.:.
```

CDH1

experimental mouse protein has muations that are shown in the rectangular box

```
human_CDH1                    MGPWSRSLSALLLLLQVSSWLCQE--PEPCHPGFDAESYTFTVPRRHLERGRVLGRVNFE   58
wild_type_mus_musculus_CDH1   MGARCRSFSALLLLLQVSSWLCQELEPESCSPGFSSEVYTFPVPERHLERGHVLGRVRFE   60
mutant_mus_musculus_CDH1      MGARCRSFSALLLLLQVSSWLCQELEPESCSPGFSSEVYTFPVPERHLERGHVLGRVRFE   60
                              **  .**:****************  ** * ***.:* *** **.******:*****.**

human_CDH1                    DCTGRQRTAYFSLDTRFKVGTDGVITVKRPLRFHNPQIHFLVYAWDSTYRKFSTKVTLNT   118
wild_type_mus_musculus_CDH1   GCTGRPRTAFFSEDSRFKVATDGTITVKRHLKLHKLETSFLVRARDSSHRELSTKVTLKS   120
mutant_mus_musculus_CDH1      GCTGRPRTAFFSEDSRFKVATDGTITVKRHLKLHKLETSFLVRARDSSHRELSTKVTLKS   120
                              .**** ***:** *:****.***.***** *::*: :  *** * **::*::******::

human_CDH1                    VGHHHRPPPHQASVSGIQAELLTFPNSSPGLRRQKRDWVIPPISCPENEKGPFPKNLVQI   178
wild_type_mus_musculus_CDH1   MGHHHHRHHHRDPASESNPELLMFPSVYPGLRRQKRDWVIPPISCPENEKGEFPKNLVQI   180
mutant_mus_musculus_CDH1      MGHHHHRHHHRDPASESNPELLMFPSVYPGLRRQKRDWVIPPISCPENEKGEFPKNLVQI   180
                              :****:   *:  .*  : *** **.  *********************** ********

human_CDH1                    KSNKDKEGKVFYSITGQGADTPPVGVFIIERETGWLKVTEPLDRERIATYTLFSHAVSSN   238
wild_type_mus_musculus_CDH1   KSNRDKETKVFYSITGQGADKPPVGVFIIERETGWLKVTQPLDREAIAKYILYSHAVSSN   240
mutant_mus_musculus_CDH1      KSNRDKETKVFYSITGQGADKPPVGVFIIERETGWLKVTQPLDREAIAKYILYSHAVSSN   240
                              ***:*** ************.******************:***** **.* *:*******

human_CDH1                    GNAVEDPMEILITVTDQNDNKPEFTQEVFKGSVMEGALPGTSVMEVTATDADDDVNTYNA   298
wild_type_mus_musculus_CDH1   GEAVEDPMEIVITVTDQNDNRPEFTQPVFEGFVAEGAVPGTSVMKVSATDADDDVNTYNA   300
mutant_mus_musculus_CDH1      GEAVEDPMEIVITVTDQNDNRPEFTQEVFEGSVAEGAVPGTSVMKVSATDADDDVNTYNA   300
                              *:********:*********:***** **:* * ***:******:*:*************
```

```
human_CDH1                      AIAYTILSQDPELPDKNMFTINRNTGVISVVTTGLDRESFPTYTLVVQAADLQGEGLSTT  358
wild_type_mus_musculus_CDH1     AIAYTIVSQDPELPHKNMFTVNRDTGVISVLTSGLDRESYPTYTLVVQAADLQGEGLSTT  360
mutant_mus_musculus_CDH1        AIAYTIVSQDPELPHKNMFTVNRDTGVISVLTSGLDRESYPTYTLVVQAADLQGEGLSTT  360
                                ******:*******.*****:**:******:*:******:********************

human_CDH1                      ATAVITVTDTNDNPPIFNPTTYKGQVPENEANVVITTLKVTDADAPNTPAWEAVYTILND  418
wild_type_mus_musculus_CDH1     AKAVITVKDINDNAPVFNPSTYQGQVPENEVNARIATLKVTDDDAPNTPAWKAVYTVVND  420
mutant_mus_musculus_CDH1        AKAVITVKDINDNAPVFNPSTYQGQVPENEVNARIATLKVTDDDAPNTPAWKAVYTVVND  420
                                *.*****.* *** *:***:**:*******.*. *:****** ********:****::**

human_CDH1                      DGGQFVVTTNPVNNDGILKTAKGLDFEAKQQYILHVAVTNVVPFEVSLTTSTATVTVDVL  478
wild_type_mus_musculus_CDH1     PDQQFVVVTDPTTNDGILKTAKGLDFEAKQQYILHVRVENEEPFEGSLVPSTATVTVDVV  480
mutant_mus_musculus_CDH1        PDQQFVVVTDPTTNDGILKTAKGLDFEAKQQYILHVRVENEEPFEGSLVPSTATVTVDVV  480
                                 . ****.*:*..*********************** * *  *** **. *********:

human_CDH1                      DVNEAPIFVPPEKRVEVSEDFGVGQEITSYTAQEPDTFMEQKITYRIWRDTANWLEINPD  538
wild_type_mus_musculus_CDH1     DVNEAPIFMPAERRVEVPEDFGVGQEITSYTAREPDTFMDQKITYRIWRDTANWLEINPE  540
mutant_mus_musculus_CDH1        DVNEAPIFMPAERRVEVPEDFGVGQEITSYTAREPDTFMDQKITYRIWRDTANWLEINPE  540
                                ********:* *:**** **************:******:*******************:

human_CDH1                      TGAISTRAELDREDFEHVKNSTYTALIIATDNGSPVATGTGTLLLILSDVNDNAPIPEPR  598
wild_type_mus_musculus_CDH1     TGAIFTRAEMDREDAEHVKNSTYVALIIATDDGSPIATGTGTLLLVLLDVNDNAPIPEPR  600
mutant_mus_musculus_CDH1        TGAIFTRAEMDREDAEHVKNSTYVALIIATDDGSPIATGTGTLLLVLLDVNDNAPIPEPR  600
                                **** ****:**** ********.*******:***:*********:* ************
```

```
human_CDH1                      TIFFCERNPKPQVINIIDADLPPNTSPFTAELTHGASANWTIQYNDPTQESIILKPKMAL   658
wild_type_mus_musculus_CDH1     NMQFCQRNPQPHIITILDPDLPPNTSPFTAELTHGASVNWTIEYNDAAQESLILQPRKDL   660
mutant_mus_musculus_CDH1        NMQFCQRNPQPHIITILDPDLPPNTSPFTAELTHGASVNWTIEYNDAAQESLILQPRKDL   660
                                .: **:***:*::*.*:* ******************.****:*** :***:**:*:  *

human_CDH1                      EVGDYKINLKLMDNQNKDQVTTLEVSVCDCEGAAGVCRKAQPVEAGLQIPAILGILGGIL   718
wild_type_mus_musculus_CDH1     EIGEYKIHLKLADNQNKDQVTTLDVHVCDCEGTVNNCMKAGIVAAGLQVPAILGILGGIL   720
mutant_mus_musculus_CDH1        EIGEYKIHLKLADNQNKDQVTTLDVHVCDCEGTVNNCMKAGIVAAGLQVPAILGILGGIL   720
                                *:*:***:*** ***********:* ******:.. * **  * ****:***********

human_CDH1                      ALLILILLLLLFLRRRAVVKEPLLPPEDDTRDNVYYYDEEGGGEEDQDFDLSQLHRGLDA   778
wild_type_mus_musculus_CDH1     ALLILILLLLLFLRRRTVVKEPLLPPDDDTRDNVYYYDEEGGGEEDQDFDLSQLHRGLDA   780
mutant_mus_musculus_CDH1        ALLILILLLLLFLRRRTVVKEPLLPPDDDTRDNVYYYDEEGGGEEDQDFDLSQLHRGLDA   780
                                ****************:*********:*********************************

human_CDH1                      RPEVTRNDVAPTLMSVPRYLPRPANPDEIGNFIDENLKAADTDPTAPPYDSLLVFDYEGS   838
wild_type_mus_musculus_CDH1     RPEVTRNDVAPTLMSVPQYRPRPANPDEIGNFIDENLKAADSDPTAPPYDSLLVFDYEGS   840
mutant_mus_musculus_CDH1        RPEVTRNDVAPTLMSVPQYRPRPANPDEIGNFIDENLKAADSDPTAPPYDSLLVFDYEGS   840
                                *****************:* *********************:******************

human_CDH1                      GSEAASLSSLNSSESDKDQDYDYLNEWGNRFKKLADMYGGGEDD   882
wild_type_mus_musculus_CDH1     GSEAASLSSLNSSESDQDQDYDYLNEWGNRFKKLADMYGGGEDD   884
mutant_mus_musculus_CDH1        GSEAASLSSLNSSESDQDQDYDYLNEWGNRFKKLADMYGGGEDD   884
                                ****************:***************************
```

CHEK2

experimental mouse protein has muations that are shown in the rectangular box and eventually truncated

```
human_CHEK2                      SSHSSSGTLSSLETVSTQELYSIPEDQEPEDQEPEEPTPAPWARLWALQDGFANLETESG  111
wild_type_mus_musculus_CHEK2     SSHSSSGTLSSLETVSTQELCSIPEDQ-----EPEEPGPAPWARLWALQDGFSN------  109
mutant_mus_musculus_CHEK2        SSHSSSGTLSSLETVSTQELCSIPEDQ-----EPEEPGPAPWARLWALQDGFSN------  109
                                 ******************** ******     ***** **************:*

human_CHEK2                      HVTQSDLELLLSSDPPASASQSAGIRGVRHHPRPVCSLKCVNDNYWFGRDKSCEYCFDEP  171
wild_type_mus_musculus_CHEK2     -------------------------------------LDCVNDNYWFGRDKSCEYCFDGP  132
mutant_mus_musculus_CHEK2        -------------------------------------LDCVNDNYWFGRDKSCEYCFDGP  132
                                                                      *.******************* *

human_CHEK2                      LLKRTDKYRTYSKKHFRIFREVGPKNSYIAYIEDHSGNGTFVNTELVGKGKRRPLNNNSE  231
wild_type_mus_musculus_CHEK2     LLRRTDKYRTYSKKHFRIFREMGPKNCYIVYIEDHSGNGTFVNTELIGKGKRCPLSNNSE  192
mutant_mus_musculus_CHEK2        LLRRTDKYRTYSKKHFRIFREMGPKNCYIVYIEDHSGNGTFVNTELIGKGKRCPLSNNSE  192
                                 **:******************:****.**.****************:***** **.****

human_CHEK2                      IALSLSRNKVFVFFDLTVDDQSVYPKALRDEYIMSKTLGSGACGEVKLAFERKTCKKVAI  291
wild_type_mus_musculus_CHEK2     IALSLCRNKVFVFFDLTVDDQSVYPKELRDEYIMSKTLGSGACGEVKMAFERKTCQKVAI  252
mutant_mus_musculus_CHEK2        IALSLCRNKVFVFFDLTVDDQSVYPKELRDEYIMSKTLGSGACGEVKMAFERKTCQKVAI  252
                                 *****.******************** ********************:*******:****

human_CHEK2                      KIISKRKFAIGSAREADPALNVETEIEILKKLNHPCIIKIKNFFDAEDYYIVLELMEGGE  351
wild_type_mus_musculus_CHEK2     KIISKRRFALGSSREADTAPSVETEIEILKKLNHPCIIKIKDVFDAEDYYIVLELMEGGE  312
mutant_mus_musculus_CHEK2        KIISKRRFALGSSREADTAPSVETEIEILKKLNHPCIIKIKDVFDAEDYYIVLELMEGGE  312
                                 ******:**:**:**** * .********************:.*****************
```

```
human_CHEK2                         LFDKVVGNKRLKEATCKLYFYQMLLAVQYLHENGIIHRDLKPENVLLSSQEEDCLIKITD  411
wild_type_mus_musculus_CHEK2        LFDRVVGNKRLKEATCKLYFYQMLVAVQYLHENGIIHRDLKPENVLLSSQEEDCLIKITD  372
mutant_mus_musculus_CHEK2           LFDRVVGNKRLKEATCKLYFYQMLVAVQYLHENGIIHRDLKPENVLLSSQEEDCLIKITD  372
                                    ***:********************:***********************************

human_CHEK2                         FGHSKILGETSLMRTLCGTPTYLAPEVLVSVGTAGYNRAVDCWSLGVILFICLSGYPPFS  471
wild_type_mus_musculus_CHEK2        FGQSKILGETSLMRTLCGTPTYLAPEVLVSNGTAGYSRAVDCWSLGVILFICLSGYPPFS  432
mutant_mus_musculus_CHEK2           FGQSKILGETSLMRTLCGTPTYLAPEVLVSNGTAGYSRAVDCWSLGVILFICLSGYPPFS  432
                                    **:*************************** *****.***********************

human_CHEK2                         EHRTQVSLKDQITSGKYNFIPEVWAEVSEKALDLVKKLLVVDPKARFTTEEALRHPWLQD  531
wild_type_mus_musculus_CHEK2        EHKTQVSLKDQITSGKYNFIPEVWTDVSEEALDLVKKLLVVDPKARLTTEEALNHPWLQD  492
mutant_mus_musculus_CHEK2           EHKTQVSLKDQITSGKYNFIPEVWTDVSEEALDLVKKLLVVDPKARLTTEEALNHPWLQD  492
                                    **:*********************::***:****************:******.******

human_CHEK2                         EDMKRKFQDLLSEENESTALPQVLAQPSTSRKRPREGEAEGAETTKRPAVCAAVL       586
wild_type_mus_musculus_CHEK2        EYMKKKFQDLLVQEKNSVTLPVAPAQT-SSQKRPLELEVEGMPSTKRLSVCGAVL       546
mutant_mus_musculus_CHEK2           EYMKKKFQDLLVQEKNSVTLPVAPAQV-F--------------------------       520
                                    * **:****** :*::*.:** . **
```

NF1

experimental mouse protein has muations that are shown in the rectangular box

```
human_NF1_isoform_2         MAAHRPVEWVQAVVSRFDEQLPIKTGQQNTHTKVSTEHNKECLINISKYKFSLVISGLTT    60
wildtype_mus_musculus_NF1   MAAHRPVEWVQAVVSRFDEQLPIKTGQQNTHTKVSTEHNKECLINISKYKFSLVISGLTT    60
mutant_mus_musculus_NF1     MAAHRPVEWVQAVVSRFDEQLPIKTGQQNTHTKVSTEHNKECLINISKYKFSLVISGLTT    60
                            ************************************************************

human_NF1_isoform_2         ILKNVNNMRIFGEAAEKNLYLSQLIILDTLEKCLAGQPKDTMRLDETMLVKQLLPEICHF    120
wildtype_mus_musculus_NF1   ILKNVNNMRIFGEAAEKNLYLSQLIILDTLEKCLAGQPKDTMRLDETMLVKQLLPEICHF    120
mutant_mus_musculus_NF1     ILKNVNNMRIFGEAAEKNLYLSQLIILDTLEKCLAGQPKDTMRLDETMLVKQLLPEICHF    120
                            ************************************************************

human_NF1_isoform_2         LHTCREGNQHAAELRNSASGVLFSLSCNNFNAVFSRISTRLQELTVCSEDNVDVHDIELL    180
wildtype_mus_musculus_NF1   LHTCREGNQHAAELRNSASGVLFSLSCNNFNAVFSRISTRLQELTVCSEDNVDVHDIELL    180
mutant_mus_musculus_NF1     LHTCREGNQHAAELRNSASGVLFSLSCNNFNAVFSRISTRLQELTVCSEDNVDVHDIELL    180
                            ************************************************************

human_NF1_isoform_2         QYINVDCAKLKRLLKETAFKFKALKKVAQLAVINSLEKAFWNWVENYPDEFTKLYQIPQT    240
wildtype_mus_musculus_NF1   QYINVDCAKLKRLLKETAFKFKALKKVAQLAVINSLEKAFWNWVENYPDEFTKLYQIPQT    240
mutant_mus_musculus_NF1     QYINVDCAKLKRLLKETAFKFKALKKVAQLAVINSLEKAFWNWVENYPDEFTKLYQIPQT    240
                            ************************************************************

human_NF1_isoform_2         DMAECAEKLFDLVDGFAESTKRKAAVWPLQIILLILCPEIIQDISKDVVDENNMNKKLFL    300
wildtype_mus_musculus_NF1   DMAECAEKLFDLVDGFAESTKRKAAVWPLQIILLILCPEIIQDISKDVVDESNINKKLFL    300
mutant_mus_musculus_NF1     DMAECAEKLFDLVDGFAESTKRKAAVWPLQIILLILCPEIIQDISKDVVDESNINKKLFL    300
                            ***************************************************.*:******
```

```
human_NF1_isoform_2          DSLRKALAGHGGSRQLTESAAIACVKLCKASTYINWEDNSVIFLLVQSMVVDLKNLLFNP    360
wildtype_mus_musculus_NF1    DSLRKALAGHGGSRQLTESAAIACVKLCKASTYINWEDNSVIFLLVQSMVVDLKNLLFNP    360
mutant_mus_musculus_NF1      DSLRKALAGHGGSRQLTESAAIACVKLCKACTYINWEDNSVIFLLVQSMVVDLKNLLFNP    360
                             ******************************.*****************************

human_NF1_isoform_2          SKPFSRGSQPADVDLMIDCLVSCFRISPHNNQHFKICLAQNSPSTFHYVLVNSLHRIITN    420
wildtype_mus_musculus_NF1    SKPFSRGSQPADVDLMIDCLVSCFRISPHNNQHFKICLAQNSPSTFHYVLVNSLHRIITN    420
mutant_mus_musculus_NF1      SKPFSRGSQPADVDLMIDCLVSCFRISPHNNQHFKICLAQNSPSTFHYVLVNSLHRIITN    420
                             ************************************************************

human_NF1_isoform_2          SALDWWPKIDAVYCHSVELRNMFGETLHKAVQGCGAHPAIRMAPSLTFKEKVTSLKFKEK    480
wildtype_mus_musculus_NF1    SALDWWPKIDAVYCHSVELRNMFGETLHKAVQGCGAHPAIRMAPSLTFKEKVTSLKFKEK    480
mutant_mus_musculus_NF1      SALDWWPKIDAVYCHSVELRNMFGETLHKAVQGCGAHPAIRMAPSLTFKEKVTSLKFKEK    480
                             ************************************************************

human_NF1_isoform_2          PTDLETRSYKYLLLSMVKLIHADPKLLLCNPRKQGPETQGSTAELITGLVQLVPQSHMPE    540
wildtype_mus_musculus_NF1    PTDLETRSYKCLLLSMVKLIHADPKLLLCNPRKQGPETQSSTAELITGLVQLVPQSHMPE    540
mutant_mus_musculus_NF1      PTDLETRSYKCLLLSMVKLIHADPKLLLCNPRKQGPETQSSTAELITGLVQLVPQSHMPE    540
                             ********** ****************************.********************

human_NF1_isoform_2          IAQEAMEALLVLHQLDSIDLWNPDAPVETFWEISSQMLFYICKKLTSHQMLSSTEILKWL    600
wildtype_mus_musculus_NF1    VAQEAMEALLVLHQLDSIDLWNPDAPVETFWEISSQMLFYICKKLTSHQMLSSTEILKWL    600
mutant_mus_musculus_NF1      VAQEAMEALLVLHQLDSIDLWNPDAPVETFWEISSQMLFYICKKLTSHQMLSSTEILKWL    600
                             :***********************************************************
```

```
human_NF1_isoform_2           REILICRNKFLLKNKQADRSSCHFLLFYGVGCDIPSSGNTSQMSMDHEELLR--TPGASL    658
wildtype_mus_musculus_NF1     REILICRNKFLLKNKQADRSSCHSLYLYGVGCEMSATGNTTQMSVDHDEFLRACTPGASL    660
mutant_mus_musculus_NF1       REILICRNKFLLKNKQADRSSCHSLYLYGVGCEMSATGNTTQMSVDHDEFLRACTPGASL    660
                              *********************** * :*****:: ::***:***:**:*:**  ******

human_NF1_isoform_2           RKGKGNSSMDSAAGCSGTPPICRQAQTKLEVALYMFLWNPDTEAVLVAMSCFRHLCEEAD    718
wildtype_mus_musculus_NF1     RKGRGNSSMDSTAGCSGTPPICRQAQTKLEVALYMFLWNPDTEAVLVAMSCFRHLCEEAD    720
mutant_mus_musculus_NF1       RKGRGNSSMDSTAGCSGTPPICRQAQTKLEVALYMFLWNPDTEAVLVAMSCFRHLCEEAD    720
                              ***:*******:************************************************

human_NF1_isoform_2           IRCGVDEVSVHNLLPNYNTFMEFASVSNMMSTGRAALQKRVMALLRRIEHPTAGNTEAWE    778
wildtype_mus_musculus_NF1     IRCGVDEVSVHNFLPNYNTFMEFASVSNMMSTGRAALQKRVMALLRRIEHPTAGNIEAWE    780
mutant_mus_musculus_NF1       IRCGVDEVSVHNFLPNYNTFMEFASVSNMMSTGRAALQKRVMALLRRIEHPTAGNIEAWE    780
                              ************:****************************************** ****

human_NF1_isoform_2           DTHAKWEQATKLILNYPKAKMEDGQAAESLHKTIVKRRMSHVSGGGSIDLSDTDSLQEWI    838
wildtype_mus_musculus_NF1     DTHAKWEQATKLILNYPKAKMEDGQAAESLHKTIVKRRMSHVSGGGSIDLSDTDSLQEWI    840
mutant_mus_musculus_NF1       DTHAKWEQATKLILNYPKAKMEDGQAAESLHKTIVKRRMSHVSGGGSIDLSDTDSLQEWI    840
                              ************************************************************

human_NF1_isoform_2           NMTGFLCALGGVCLQQRSNSGLATYSPPMGPVSERKGSMISVMSSEGNADTPVSKFMDRL    898
wildtype_mus_musculus_NF1     NMTGFLCALGGVCLQQRSSSGLATYSPPMGAVSERKGSMISVMSSEGNIDSPVSRFMDRL    900
mutant_mus_musculus_NF1       NMTGFLCALGGVCLQQRSSSGLATYSPPMGAVSERKGSMISVMSSEGNIDSPVSRFMDRL    900
                              ******************.*********** ***************** *:***:*****
```

```
human_NF1_isoform_2          LSLMVCNHEKVGLQIRTNVKDLVGLELSPALYPMLFNKLKNTISKFFDSQGQVLLTDTNT     958
wildtype_mus_musculus_NF1    LSLMVCNHEKVGLQIRTNVKDLVGLELSPALYPMLFNKLKNTISKFFDSQGQVLLSDSNT     960
mutant_mus_musculus_NF1      LSLMVCNHEKVGLQIRTNVKDLVGLELSPALYPMLFNKLKNTISKFFDSQGQVLLSDSNT     960
                             *******************************************************:*:**

human_NF1_isoform_2          QFVEQTIAIMKNLLDNHTEGSSEHLGQASIETMMLNLVRYVRVLGNMVHAIQIKTKLCQL     1018
wildtype_mus_musculus_NF1    QFVEQTIAIMKNLLDNHTEGSSEHLGQASIETMMLNLVRYVRVLGNMVHAIQIKTKLCQL     1020
mutant_mus_musculus_NF1      QFVEQTIAIMKNLLDNHTEGSSEHLGQASIETMMLNLVRYVRVLGNMVHAIQIKTKLCQL     1020
                             ************************************************************

human_NF1_isoform_2          VEVMMARRDDLSFCQEMKFRNKMVEYLTDWVMGTSNQAADDDVKCLTRDLDQASMEAVVS     1078
wildtype_mus_musculus_NF1    VEVMMARRDDLSFCQEMKFRNKMVEYLTDWVMGTSNQAADDDIKCLTRDLDQASMEAVVS     1080
mutant_mus_musculus_NF1      VEVMMARRDDLSFCQEMKFRNKMVEYLTDWVMGTSNQAADDDIKCLTRDLDQASMEAVVS     1080
                             ******************************************:*****************

human_NF1_isoform_2          LLAGLPLQPEEGDGVELMEAKSQLFLKYFTLFMNLLNDCSEVEDESAQTGGRKRGMSRRL     1138
wildtype_mus_musculus_NF1    LLAGLPLQPEEGDGVELMEAKSQLFLKYFTLFMNLLNDCSEVEDENAQTGGRKRGMSRRL     1140
mutant_mus_musculus_NF1      LLAGLPLQPEEGDGVELMEAKSQLFLKYFTLFMNLLNDCSEVEDENAQTGGRKRGMSRRL     1140
                             *********************************************.**************

human_NF1_isoform_2          ASLRHCTVLAMSNLLNANVDSGLMHSIGLGYHKDLQTRATFMEVLTKILQQGTEFDTLAE     1198
wildtype_mus_musculus_NF1    ASLRHCTVLAMSNLLNANVDSGLMHSIGLGYHKDLQTRATFMEVLTKILQQGTEFDTLAE     1200
mutant_mus_musculus_NF1      ASLRHCTVLAMSNLLNANVDSGLMHSIGLGYHKDLQTRATFMEVLTKILQQGTEFDTLAE     1200
                             ************************************************************
```

```
human_NF1_isoform_2         TVLADRFERLVELVTMMGDQGELPIAMALANVVPCSQWDELARVLVTLFDSRHLLYQLLW    1258
wildtype_mus_musculus_NF1   TVLADRFERLVELVTMMGDQGELPIAMALANVVPCSQWDELARVLVTLFDSRHLLYQLLW    1260
mutant_mus_musculus_NF1     TVLADRFERLVELVTMMGDQGELPIAMALANVVPCSQWDELARVLVTLFDSRHLLYQLLW    1260
                            ************************************************************

human_NF1_isoform_2         NMFSKEVELADSMQTLFRGNSLASKIMTFCFKVYGATYLQKLLDPLLRIVITSSDWQHVS    1318
wildtype_mus_musculus_NF1   NMFSKEVELADSMQTLFRGNSLASKIMTFCFKVYGATYLQKLLDPLLRVIITSSDWQHVS    1320
mutant_mus_musculus_NF1     NMFSKEVELADSMQTLFRGNSLASKIMTFCFKVYGATYLQKLLDPLLRVIITSSDWQHVS    1320
                            ************************************************::**********

human_NF1_isoform_2         FEVDPTRLEPSESLEENQRNLLQMTEKFFHAIISSSSEFPPQLRSVCHCLYQ--------    1370
wildtype_mus_musculus_NF1   FEVDPTRLEPSESLEENQRNLLQMTEKFFHAIISSSSEFPSQLRSVCHCLYQATCHSLLN    1380
mutant_mus_musculus_NF1     FEVDPTRLEPSESLEENQRNLLQMTEKFFHAIISSSSEFPSQLRSVCHCLYQATCHSLLN    1380
                            **************************************** ***********

human_NF1_isoform_2         -------------VVSQRFPQNSIGAVGSAMFLRFINPAIVSPYEAGILDKKPPPRIERG    1417
wildtype_mus_musculus_NF1   KATVKERKENKKSVVSQRFPQNSIGAVGSAMFLRFINPAIVSPYEAGILDKKPPPRIERG    1440
mutant_mus_musculus_NF1     KATVKERKENKKSVVSQRFPQNSIGAVGSAMFLRFINPAIVSPYEAGILDKKPPPRIERG    1440
                                         ***********************************************

human_NF1_isoform_2         LKLMSKILQSIANHVLFTKEEHMRPFNDFVKSNFDAARRFFLDIASDCPTSDAVNHSLSF    1477
wildtype_mus_musculus_NF1   LKLMSKVLQSIANHVLFTKEEHMRPFNDFVKSNFDLARRFFLDIASDCPTSDAVNHSLSF    1500
mutant_mus_musculus_NF1     LKLMSKVLQSIANHVLFTKEEHMRPFNDFVKSNFDLARRFFLDIASDCPTSDAVNHSLSF    1500
                            ******:**************************** ************************
```

```
human_NF1_isoform_2         ISDGNVLALHRLLWNNQEKIGQYLSSNRDHKAVGRRPFDKMATLLAYLGPPEHKPVADTH    1537
wildtype_mus_musculus_NF1   ISDGNVLALHRLLWNNQEKIGQYLSSNRDHKAVGRRPFDKMATLLAYLGPPEHKPVADTH    1560
mutant_mus_musculus_NF1     ISDGNVLALHRLLWNNQEKIGQYLSSNRDHKAVGRRPFDKMATLLAYLGPPEHKPVADTH    1560
                            ************************************************************

human_NF1_isoform_2         WSSLNLTSSKFEEFMTRHQVHEKEEFKALKTLSIFYQAGTSKAGNPIFYYVARRFKTGQI    1597
wildtype_mus_musculus_NF1   WSSLNLTSSKFEEFMTRHQVHEKEEFKALKTLSIFYQAGTSKAGNPIFYYVARRFKTGQI    1620
mutant_mus_musculus_NF1     WSSLNLTSSKFEEFMTRHQVHEKEEFKALKTLSIFYQAGTSKAGNPIFYYVARRFKTGQI    1620
                            ************************************************************

human_NF1_isoform_2         NGDLLIYHVLLTLKPYYAKPYEIVVDLTHTGPSNRFKTDFLSKWFVVFPGFAYDNVSAVY    1657
wildtype_mus_musculus_NF1   NGDLLIYHVLLTLKPYYAKPYEIVVDLTHTGPSNRFKTDFLSKWFVVFPGFAYDNVSAVY    1680
mutant_mus_musculus_NF1     NGDLLIYHVLLTLKPYYAKPYEIVVDLTHTGPSNRFKTDFLSKWFVVFPGFAYDNVSAVY    1680
                            ************************************************************

human_NF1_isoform_2         IYNCNSWVREYTKYHERLLTGLKGSKRLVFIDCPGKLAEHIEHEQQKLPAATLALEEDLK    1717
wildtype_mus_musculus_NF1   IYNCNSWVREYTKYHERLLTGLKGSKRLIFIDCPGKLAEHIEHEQQKLPAATLALEEDLK    1740
mutant_mus_musculus_NF1     IYNCNSWVREYTKYHERLLTGLKGSKRLIFIDCPGKLAEHIEHEQQKLPAATLALEEDLK    1740
                            ****************************:*******************************

human_NF1_isoform_2         VFHNALKLAHKDTKVSIKVGSTAVQVTSAERTKVLGQSVFLNDIYYASEIEEICLVDENQ    1777
wildtype_mus_musculus_NF1   VFHNALKLAHKDTKVSIKVGSTAVQVTSAERTKVLGQSVFLNDIYYASEIEEICLVDENQ    1800
mutant_mus_musculus_NF1     VFHNALKLAHKDTKVSIKVGSTAVQVTSAERTKVLGQSVFLNDIYYASEIEEICLVDENQ    1800
                            ************************************************************
```

```
human_NF1_isoform_2          FTLTIANQGTPLTFMHQECEAIVQSIIHIRTRWELSQPDSIPQHTKIRPKDVPGTLLNIA    1837
wildtype_mus_musculus_NF1    FTLTIANQGTPLTFMHQECEAIVQSIIHIRTRWELSQPDSIPQHTKIRPKDVPGTLLNIA    1860
mutant_mus_musculus_NF1      FTLTIANQGTPLTFMHQECEAIVQSIIHIRTRWELSQPDSIPQHTKIRPKDVPGTLLNIA    1860
                             ************************************************************

human_NF1_isoform_2          LLNLGSSDPSLRSAAYNLLCALTCTFNLKIEGQLLETSGLCIPANNTLFIVSISKTLAAN    1897
wildtype_mus_musculus_NF1    LLNLGSSDPSLRSAAYNLLCALTCTFNLKIEGQLLETSGLCIPANNTLFIVSISKTLAAN    1920
mutant_mus_musculus_NF1      LLNLGSSDPSLRSAAYNLLCALTCTFNLKIEGQLLETSGLCIPANNTLFIVSISKTLAAN    1920
                             ************************************************************

human_NF1_isoform_2          EPHLTLEFLEECISGFSKSSIELKHLCLEYMTPWLSNLVRFCKHNDDAKRQRVTAILDKL    1957
wildtype_mus_musculus_NF1    EPHLTLEFLEECISGFSKSSIELKHLCLEYMTPWLSNLVRFCKHNDDAKRQRVTAILDKL    1980
mutant_mus_musculus_NF1      EPHLTLEFLEECISGFSKSSIELKHLCLEYMTPWLSNLVRFCKHNDDAKRQRVTAILDKL    1980
                             ************************************************************

human_NF1_isoform_2          ITMTINEKQMYPSIQAKIWGSLGQITDLLDVVLDSFIKTSATGGLGSIKAEVMADTAVAL    2017
wildtype_mus_musculus_NF1    ITMTINEKQMYPSIQAKIWGSLGQITDLLDVVLDSFIKTSATGGLGSIKAEVMADTAVAL    2040
mutant_mus_musculus_NF1      ITMTINEKQMYPSIQAKIWGSLGQITDLLDVVLDSFIKTSATGGLGSIKAEVMADTAVAL    2040
                             ************************************************************

human_NF1_isoform_2          ASGNVKLVSSKVIGRMCKIIDKTCLSPTPTLEQHLMWDDIAILARYMLMLSFNNSLDVAA    2077
wildtype_mus_musculus_NF1    ASGNVKLVSSKVIGRMCKIIDKTCLSPTPTLEQHLMWDDIAILARYMLMLSFNNSLDVAA    2100
mutant_mus_musculus_NF1      ASGNVKLVSSKVIGRMCKIIDKTCLSPTPTLEQHLMWDDIAILARYMLMLSFNNSLDVAA    2100
                             ************************************************************
```

```
human_NF1_isoform_2          HLPYLFHVVTFLVATGPLSLRASTHGLVINIIHSLCTCSQLHFSEETKQVLRLSLTEFSL    2137
wildtype_mus_musculus_NF1    HLPYLFHVVTFLVATGPLSLRASTHGLLINIIHSLCTCSQLHFSEETKQVLRLSLTEFSL    2160
mutant_mus_musculus_NF1      HLPYLFHVVTFLVATGPLSLRASTHGLLINIIHSLCTCSQLHFSEETKQVLRLSLTEFSL    2160
                             ***************************:********************************

human_NF1_isoform_2          PKFYLLFGISKVKSAAVIAFRSSYRDRSFSPGSYERETFALTSLETVTEALLEIMEACMR    2197
wildtype_mus_musculus_NF1    PKFYLLFGISKVKSAAVIAFRSSYRDRSFSPGSYERETFALTSLETVTEALLEIMEACMR    2220
mutant_mus_musculus_NF1      PKFYLLFGISKVKSAAVIAFRSSYRDRSFSPGSYERETFALTSLETVTEALLEIMEACMR    2220
                             ************************************************************

human_NF1_isoform_2          DIPTCKWLDQWTELAQRFAFQYNPSLQPRALVVFGCISKRVSHGQIKQIIRILSKALESC    2257
wildtype_mus_musculus_NF1    DIPTCKWLDQWTELAQRFAFQYNPSLQPRALVVFGCISKRVSHGQIKQIIRILSKALESC    2280
mutant_mus_musculus_NF1      DIPTCKWLDQWTELAQRFAFQYNPSLQPRALVVFGCISKRVSHGQIKQIIRILSKALESC    2280
                             ************************************************************

human_NF1_isoform_2          LKGPDTYNSQVLIEATVIALTKLQPLLNKDSPLHKALFWVAVAVLQLDEVNLYSAGTALL    2317
wildtype_mus_musculus_NF1    LKGPDTYNSQVLIESTVIALTKLQPLLNKDSPLHKALFWVAVAVLQLDEVNLYSAGTALL    2340
mutant_mus_musculus_NF1      LKGPDTYNSQVLIESTVIALTKLQPLLNKDSPLHKALFWVAVAVLQLDEVNLYSAGTALL    2340
                             **************:*********************************************

human_NF1_isoform_2          EQNLHTLDSLRIFNDKSPEEVFMAIRNPLEWHCKQMDHFVGLNFNSNFNFALVGHLLKGY    2377
wildtype_mus_musculus_NF1    EQNLHTLDSLRIFNDKSPEEVFMAIRNPLEWHCKQMDHFVGLNFNSNFNFALVGHLLKGY    2400
mutant_mus_musculus_NF1      EQNLHTLDSLRIFNDKSPEEVFMAIRNPLEWHCKQMDHFVGLNFNSNFNFALVGHLLKGY    2400
                             ************************************************************
```

```
human_NF1_isoform_2          RHPSPAIVARTVRILHTLLTLVNKHRNCDKFEVNTQSVAYLAALLTVSEEVRSRCSLKHR    2437
wildtype_mus_musculus_NF1    RHPSPAIVARTVRILHTLLTLVNKHRNCDKFEVNTQSVAYLAALLTVSEEVRSRCSLKHR    2460
mutant_mus_musculus_NF1      RHPSPAIVARTVRILHTLLTLVNKHRNCDKFEVNTQSVAYLAALLTVSEEVRSRCSLKHR    2460
                             ************************************************************

human_NF1_isoform_2          KSLLLTDISMENVPMDTYPIHHGDPSYRTLKETQPWSSPKGSEGYLAATYPTVGQTSPRA    2497
wildtype_mus_musculus_NF1    KSLLLTDISMENVPMDTYPIHHGDPSYRTLKETQPWSSPKGSEGYLAATYPAVGQTSPRA    2520
mutant_mus_musculus_NF1      KSLLLTDISMENVPMDTYPIHHGDPSYRTLKETQPWSSPKGSEGYLAATYPAVGQTSPRA    2520
                             ***************************************************:********

human_NF1_isoform_2          RKSMSLDMGQPSQANTKKLLGTRKSFDHLISDTKAPKRQEMESGITTPPKMRRVAETDYE    2557
wildtype_mus_musculus_NF1    RKSMSLDMGQPSQANTKKLLGTRKSFDHLISDTKAPKRQEMESGITTPPKMRRVAETDYE    2580
mutant_mus_musculus_NF1      RKSMSLDMGQPSQANTKKLLGTRKSFDHLISDTKAPKRQEMESGITTPPKMRRVAETDYE    2580
                             ************************************************************

human_NF1_isoform_2          METQRISSSQQHPHLRKVSVSESNVLLDEEVLTDPKIQALLLTVLATLVKYTTDEFDQRI    2617
wildtype_mus_musculus_NF1    METQRIPSSQQHPHLRKVSVSESNVLLDEEVLTDPKIQALLLTVLATLVKYTTDEFDQRI    2640
mutant_mus_musculus_NF1      METQRIPSSQQHPHLRKVSVSESNVLLDEEVLTDPKIQALLLTVLATLVKYTTDEFDQRI    2640
                             ****** *****************************************************

human_NF1_isoform_2          LYEYLAEASVVFPKVFPVVHNLLDSKINTLLSLCQDPNLLNPIHGIVQSVVYHEESPPQY    2677
wildtype_mus_musculus_NF1    LYEYLAEASVVFPKVFPVVHNLLDSKINTLLSLCQDPNLLNPIHGIVQSVVYHEESPPQY    2700
mutant_mus_musculus_NF1      LYEYLAEASVVFPKVFPVVHNLLDSKINTLLSLCQDPNLLNPIHGIVQSVVYHEESPPQY    2700
                             ************************************************************
```

```
human_NF1_isoform_2          QTSYLQSFGFNGLWRFAGPFSKQTQIPDYAELIVKFLDALIDTYLPGIDEETSEESLLTP     2737
wildtype_mus_musculus_NF1    QTSYLQSFGFNGLWRFAGPFSKQTQIPDYAELIVKFLDALIDTYLPGIDEETSEESLLTP     2760
mutant_mus_musculus_NF1      QTSYLQSFGFNGLWRFAGPFSKQTQIPDYAELIVKFLDALIDTYLPGIDEETSEESLLTP     2760
                             ************************************************************

human_NF1_isoform_2          TSPYPPALQSQLSITANLNLSNSMTSLATSQHSPGIDKENVELSPTTGHCNSGRTRHGSA     2797
wildtype_mus_musculus_NF1    TSPYPPALQSQLSITANLNLSNSMTSLATSQHSPGLDKENVELSPTAGHCNSGRTRHGSA     2820
mutant_mus_musculus_NF1      TSPYPPALQSQLSITANLNLSNSMTSLATSQHSPGLDKENVELSPTAGHCNSGRTRHGSA     2820
                             ***********************************:**********:*************

human_NF1_isoform_2          SQVQKQRSAGSFKRNSIKKIV    2818
wildtype_mus_musculus_NF1    SQVQKQRSAGSFKRNSIKKIV    2841
mutant_mus_musculus_NF1      SQVQKQRSAGSFKRNSIKKIV    2841
                             *********************
```

TP53

experimental mouse protein is severely truncated

```
human_TP53_isoform_a           ---MEEPQSDPSVEPPLSQETFSDLWKLLPENNVLSPLPSQAMDDLMLSPDDIEQWFTED    57
wildtype_mus_musculus_TP53     MTAMEESQSDISLELPLSQETFSGLWKLLPPEDILPSP--HCMDD-LLLPQDVEEFFE--    55
mutant_mus_musculus_TP53       MTAMEESQSDISLELPLSQETFSGLWKL--------------------------------    28
                                  *** *** *:* ********.****

human_TP53_isoform_a           PGPDEAPRMPEAAPPVAPAPAAPTPAAPAPAPSWPLSSSVPSQKTYQGSYGFRLGFLHSG    117
wildtype_mus_musculus_TP53     -GPSEALRVSGAPAAQDPVTETPGPVAPAPATPWPLSSFVPSQKTYQGNYGFHLGFLQSG    114
mutant_mus_musculus_TP53       ------------------------------------------------------------    28


human_TP53_isoform_a           TAKSVTCTYSPALNKMFCQLAKTCPVQLWVDSTPPPGTRVRAMAIYKQSQHMTEVVRRCP    177
wildtype_mus_musculus_TP53     TAKSVMCTYSPPLNKLFCQLAKTCPVQLWVSATPPAGSRVRAMAIYKKSQHMTEVVRRCP    174
mutant_mus_musculus_TP53       ------------------------------------------------------------    28


human_TP53_isoform_a           HHERCSDSDGLAPPQHLIRVEGNLRVEYLDDRNTFRHSVVVPYEPPEVGSDCTTIHYNYM    237
wildtype_mus_musculus_TP53     HHERCSDGDGLAPPQHLIRVEGNLYPEYLEDRQTFRHSVVVPYEPPEAGSEYTTIHYKYM    234
mutant_mus_musculus_TP53       ------------------------------------------------------------    28
```

```
human_TP53_isoform_a         CNSSCMGGMNRRPILTIITLEDSSGNLLGRNSFEVRVCACPGRDRRTEEENLRKKGEPHH    297
wildtype_mus_musculus_TP53   CNSSCMGGMNRRPILTIITLEDSSGNLLGRDSFEVRVCACPGRDRRTEEENFRKKEVLCP    294
mutant_mus_musculus_TP53     ------------------------------------------------------------    28

human_TP53_isoform_a         ELPPGSTKRALPNNTSSSPQPKKKPLDGEYFTLQIRGRERFEMFRELNEALELKDAQAGK    357
wildtype_mus_musculus_TP53   ELPPGSAKRALPTCTSASPPQKKKPLDGEYFTLKIRGRKRFEMFRELNEALELKDAHATE    354
mutant_mus_musculus_TP53     ------------------------------------------------------------    28

human_TP53_isoform_a         EPGGSRAHSSHLKSKKGQSTSRHKKLMFKTEGPDSD    393
wildtype_mus_musculus_TP53   ESGDSRAHSSYLKTKKGQSTSRHKKTMVKKVGPDSD    390
mutant_mus_musculus_TP53     ------------------------------------    28
```

Figure S1: DNA Alignment

Read Alignment (BWA) Results

Input 1: Reference Genome Sequences

GCA_000001635.9_GRCm39_genomic

Sequences	Minimum Length	Maximum Length	Average Length	Total Length
61	1,976	195,154,279	44,724,958	2,728,222,451

Input 2: Sequencing Data

A total of 2 libraries have been processed.

Sample Name	Files	Sequencing	Format
855012_R1	855012_R1.fastq.gz	Single-End	FASTQ
855012_R2	855012_R2.fastq.gz	Single-End	FASTQ

Results Overview

Globals

Sample	Total Alignments	Mapped	Supplementary	Unmapped	Duplicated Reads (estimated)	Duplication Rate
855012_R1	65,720,912	65,664,736 / 99.915%	13,786,193 / 20.977%	56,176 / 0.085%	30,317,309 / 46.13%	23.84
855012_R2	65,129,286	64,972,430 / 99.759%	13,194,567 / 20.259%	156,856 / 0.241%	29,643,127 / 45.514%	23.48

ACTG Content

Sample	A's	C's	T's	G's	N's	GC (%)
855012_R1	3,785,036,436 / 27.837%	2,803,481,848 / 20.618%	3,726,890,402 / 27.409%	3,281,708,630 / 24.135%	24,301,815 / 0.179%	44.75
855012_R2	3,753,534,125 / 27.869%	2,782,591,037 / 20.66%	3,705,079,529 / 27.509%	3,227,399,199 / 23.962%	4,782,640 / 0.036%	44.62

Coverage

Sample	Mean	Standard Deviation
855012_R1	5X	1,457.231X
855012_R2	4.945X	1,374.676X

Mappping Quality

Sample	Mean Mapping Quality
855012_R1	47.79
855012_R2	47.782

Mismatches and Indels

Sample	General Error Rate	Mismatches	Insertions	Mapped Reads with Insertion (%)	Deletions	Mapped Reds with Deletion (%)	Homopolymer Indels (%)
855012_R1	0.012	151,050,476	3,079,222	4.2	4,617,519	6.15	57.59
855012_R2	0.012	150,442,449	2,975,641	4.11	4,479,573	6.06	56.53

Analysis Parameters

Parameter	Value
Minimum Seed Length	19
Band Width	100
Z-dropoff	100
Trigger Re-seeding	1.5
Seed Occurrence	20
Skip Seeds	500
Drop Chains	0.5
Discard Chains	0
Mate Rescue Rounds	50
Skip Mate Rescue	false
Skip Pairing	false
Matching Score	1
Mismatch Penalty	4
Gap Open Penalty (DEL)	6
Gap Open Penalty (INS)	6
Gap Extension Penalty (DEL)	1
Gap Extension Penalty (INS)	1
5'-end Clipping Penalty	5
3'-end Clipping Penalty	5
Unpaired Read Penalty	17
Minimum Score	30
Split Alignments as Primary	false
MapQ of Supp. Alignments	false
Output All Alignments	false
Soft Clipping for Supp.	false
Shorter Split Hits as Secondary	false
Sort BAM File	By Read Name
Add Read Group Information	false

References

- OmicsBox - Bioinformatics made easy. BioBam Bioinformatics. March 3, 2019. www.biobam.com/omicsbox.
- Li H. and Durbin R. (2009). Fast and accurate short read alignment with Burrows-Wheeler transform. *Bioinformatics (Oxford, England), 25*(14), 1754-60.
- Li H., Handsaker B., Wysoker A., Fennell T., Ruan J., Homer N., Marth G., Abecasis G. and Durbin R. (2009). The Sequence Alignment/Map format and SAMtools. *Bioinformatics (Oxford, England), 25*(16), 2078-9.
- Okonechnikov K., Conesa A. and Garcia-Alcalde F. (2016). Qualimap 2: advanced multi-sample quality control for high-throughput sequencing data. *Bioinformatics (Oxford, England), 32*(2), 292-4.

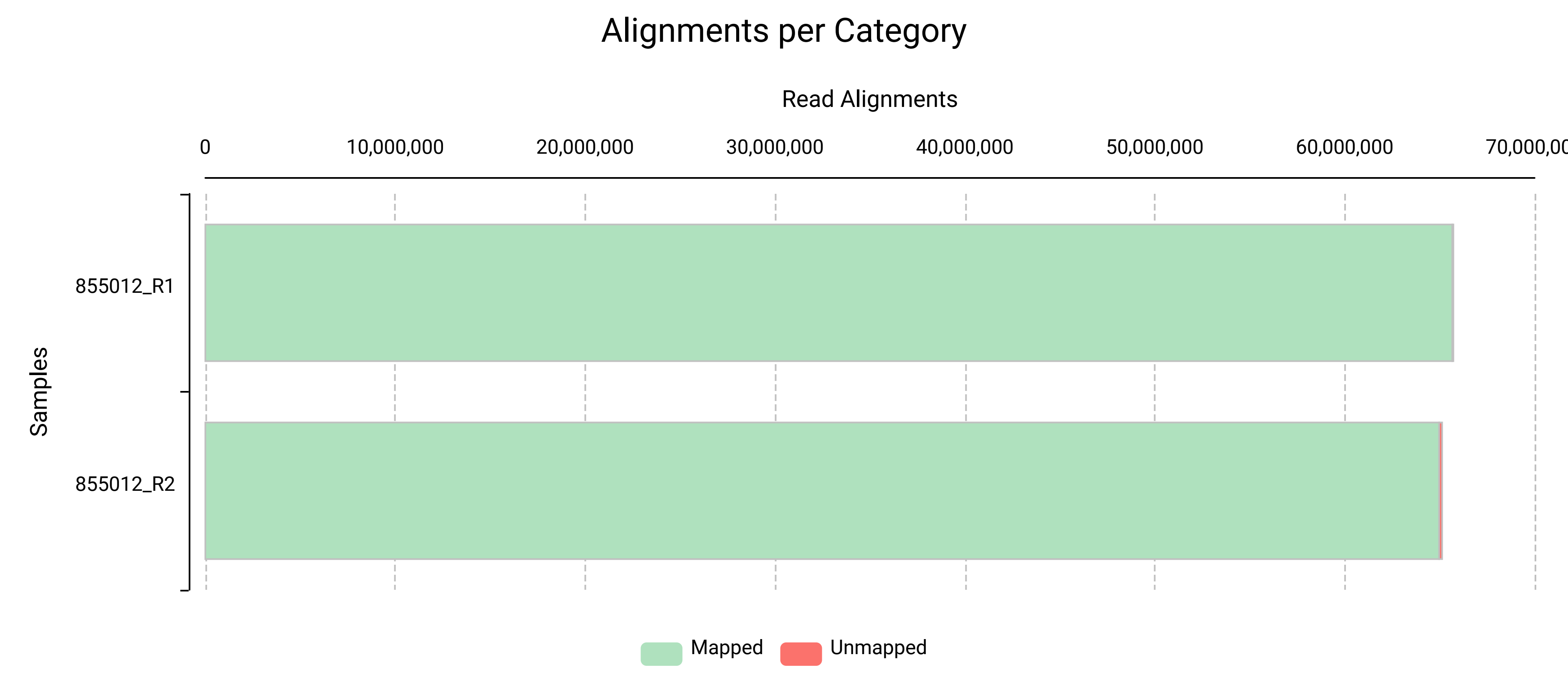

Alignments per Category
Read Alignments
0
10,000,000
20,000,000
30,000,000
40,000,000
50,000,000
60,000,000
Samples
855012_R1
855012_R2
Mapped
Unmapped

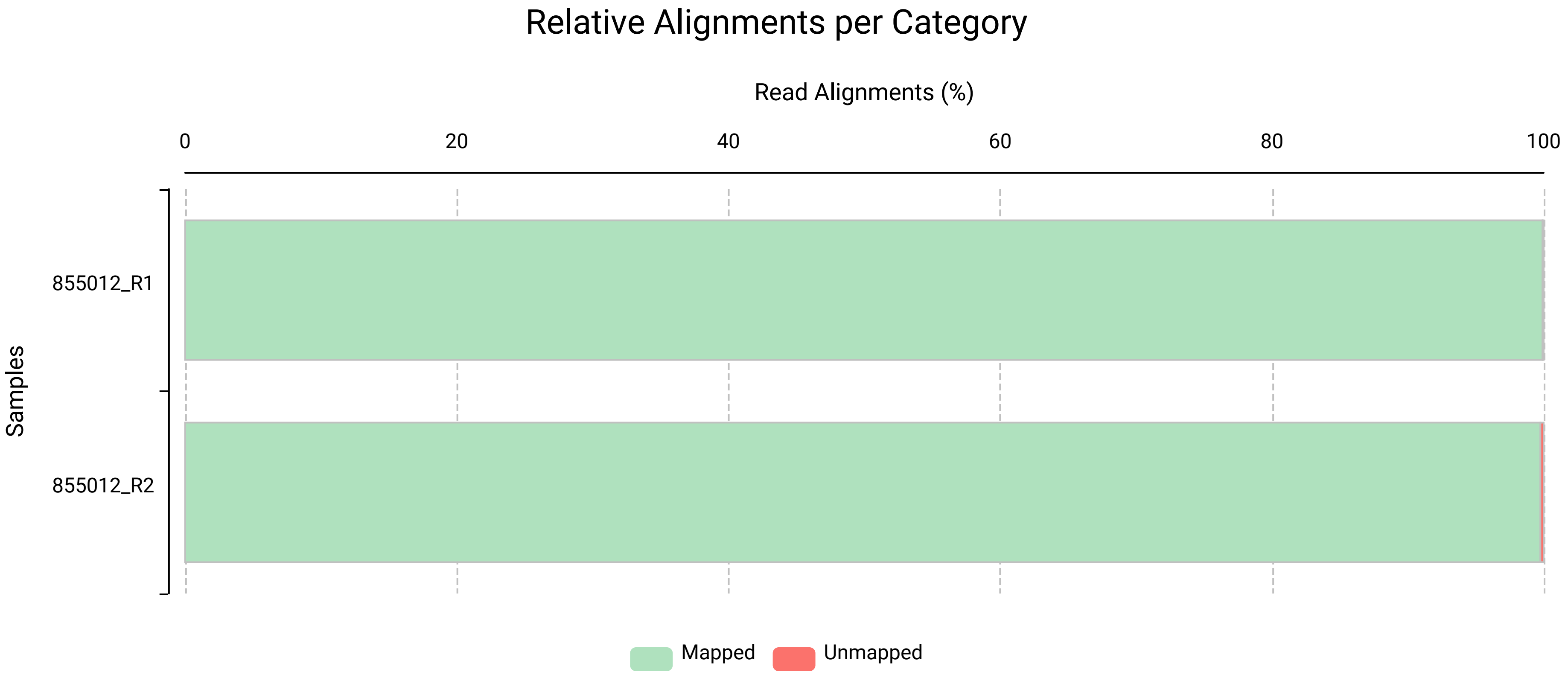
Relative Alignments per Category
Read Alignments (%)
0
20
40
60
80
100
Samples
855012_R1
855012_R2
Mapped
Unmapped

Figure S1: Sequencing Report

Illumina NextSeq Sequencing Run QC Report

Run ID: 241030_VL00482_40_AACJNGWM5

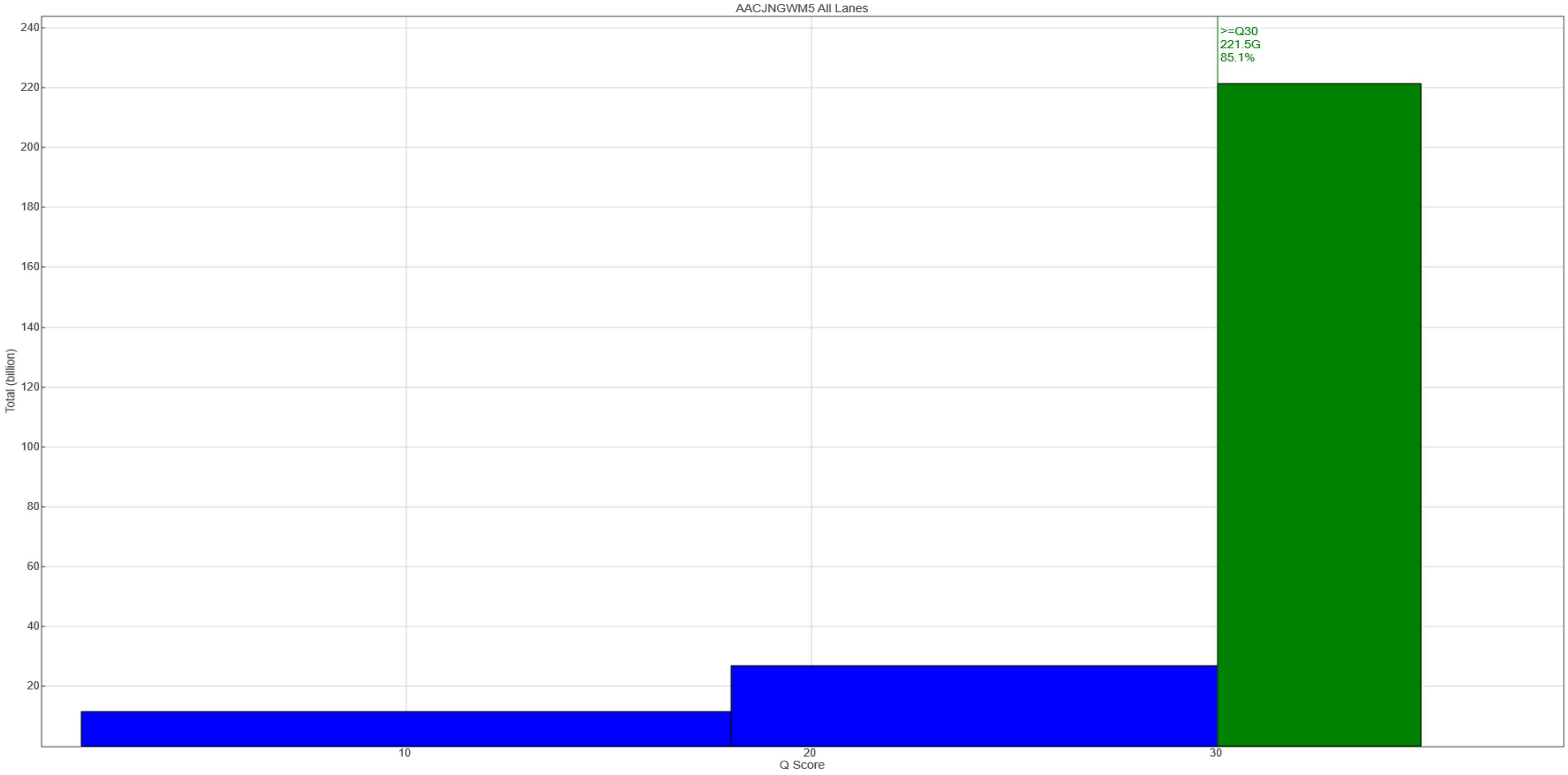

Q Score Distribution.

Q scores are used to measure base calling accuracy, one of the most common metrics for assessing sequencing data quality. Q score is a Phred-like quality score, defined as logarithmically related to the base calling error probability: $Q = -10 \log_{10}(P)$.

A base is assigned a Q score of 30 (Q30) when its base call accuracy is 99.9%, and the probability of an incorrect base call is 1 in 1000. On the NextSeq 1000 platform, a base is assigned a simplified Q score of 34 (high-quality), 26 (medium), 12 (marginal), or 2 (no-call). <u>A NextSeq sequencing run is considered high quality overall with ≥ 80% bases higher than Q30 at 2 x 300 bp</u>.

Illumina NextSeq Sequencing Run QC Report
Run ID: 241030_VL00482_40_AACJNGWM5

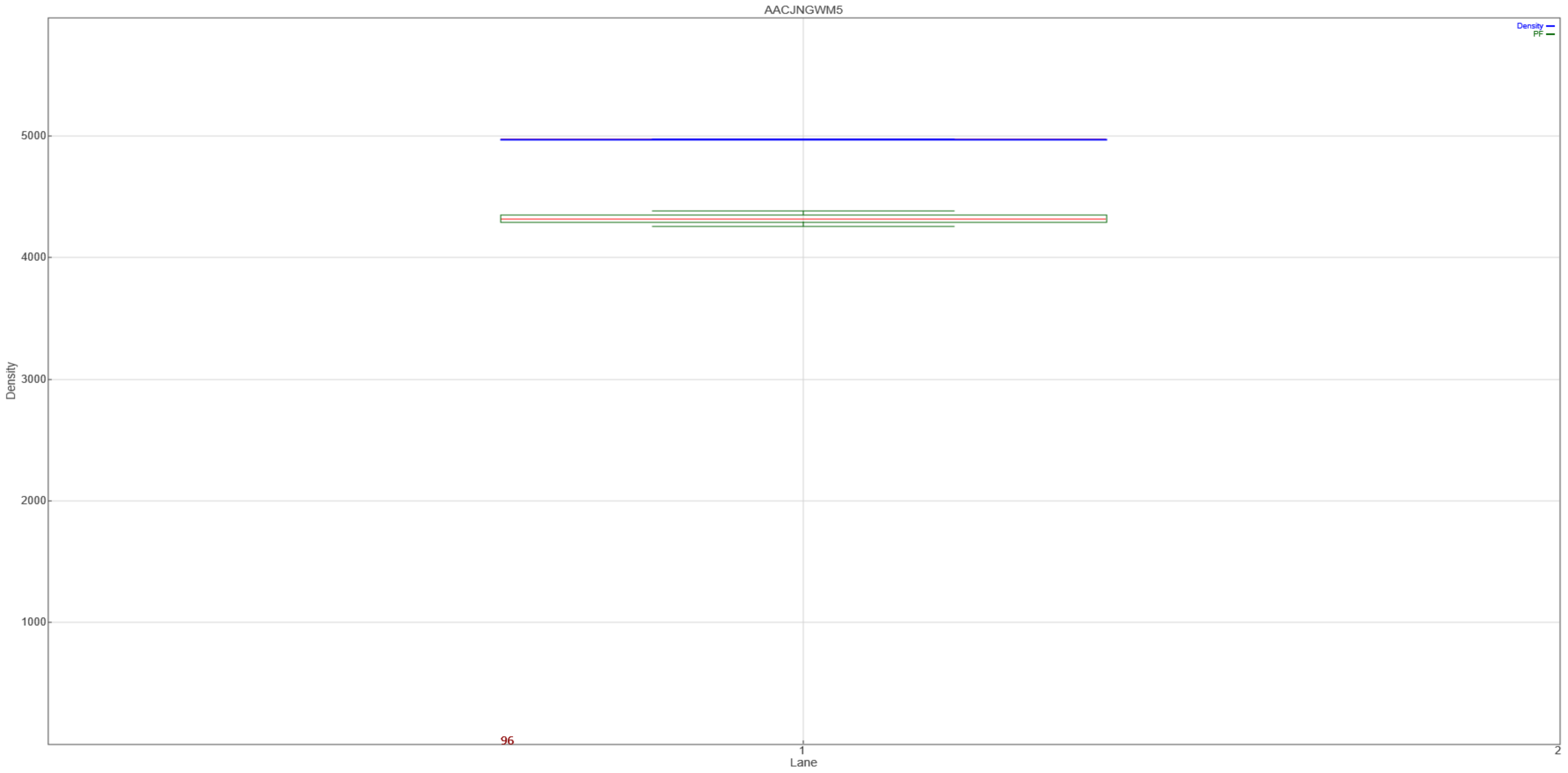

Cluster Density.

Cluster density is the density of clonal clusters produced and amplified from nucleic acid libraries over the sequencing flow cell, influencing run quality and total data output. The density box plots compare the raw cluster density (in blue) to the % cluster density passing chastity filter (in green). With optimal cluster density and loading concentration, the raw and passing-filter (PF) cluster density box plots appear close to one another. On the NextSeq 1000 platform, the patterned flow cell structure may cause % cluster PF to appear lower, but the quality of the yielded sample reads is unaffected.

Illumina NextSeq Sequencing Run QC Report
Run ID: 241030_VL00482_40_AACJNGWM5

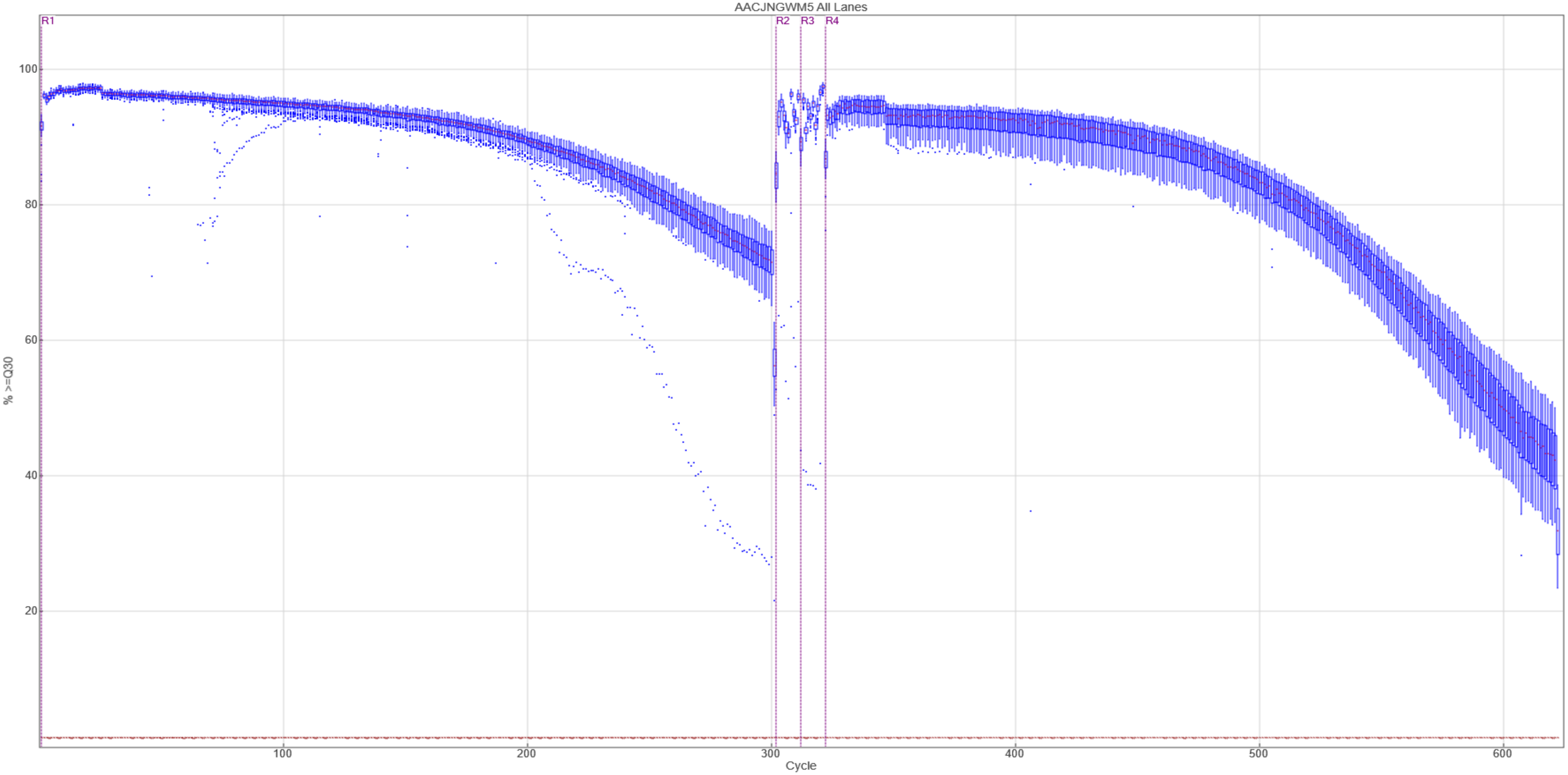

% Bases ≥ Q30 by Cycle.

% of bases with Q score ≥ 30 in each sequencing cycle. Typically, the % Bases ≥ Q30 is lower in later sequencing cycles, and overall lower in R2 cycles. This percentage resets at the beginning of R2 cycles. The short read cycles in between R1 and R2 reads denote index reads for sample multiplexing.

Q score of bases in all cycles make up the overall Q Score Distribution.

Illumina NextSeq Sequencing Run QC Report
Run ID: 241030_VL00482_40_AACJNGWM5

Run Summary

Level	Yield Total (G)	Projected Total Yield (G)	Aligned (%)	Error Rate (%)	Intensity Cycle 1	% >= Q30
Read 1	126.07	126.07	4.49	0.32	92	89.96
Read 2 (I)	3.78	3.78	0.00	NaN	162	91.86
Read 3 (I)	3.78	3.78	0.00	NaN	144	92.98
Read 4	124.91	124.91	4.39	0.42	65	80.01
Non-Indexed Total	250.98	250.98	4.44	0.37	78	85.01
Total	258.54	258.54	4.44	0.37	116	85.23

Read 1

Lane	Tiles	Density (K/mm2)	Cluster PF (%)	Legacy Phasing/Prephasing Rate	Phasing slope/offset	Prephasing slope/offset	Cluster Count Raw (M)	Cluster Count PF (M)	% >= Q30	Yield (G)	Cycles Err Rated	Aligned (%)	Error Rate (%)	Error Rate 35 cycle (%)	Error Rate 75 cycle (%)	Error Rate 100 cycle (%)	Intensity Cycle 1
1	96	4974 ± 0	86.91 ± 0.60	0.124 / 0.043	0.075 / 0.600	0.046 / 0.234	484.31	420.93	89.96	126.07	300	4.49 ± 0.08	0.32 ± 0.15	0.05 ± 0.01	0.12 ± 0.37	0.13 ± 0.26	92 ± 6

Read 2 (I)

Lane	Tiles	Density (K/mm2)	Cluster PF (%)	Legacy Phasing/Prephasing Rate	Phasing slope/offset	Prephasing slope/offset	Cluster Count Raw (M)	Cluster Count PF (M)	% >= Q30	Yield (G)	Cycles Err Rated	Aligned (%)	Error Rate (%)	Error Rate 35 cycle (%)	Error Rate 75 cycle (%)	Error Rate 100 cycle (%)	Intensity Cycle 1
1	96	4974 ± 0	86.91 ± 0.60	NaN / NaN	NaN / NaN	NaN / NaN	484.31	420.93	91.86	3.78	0	NaN ± NaN	NaN ± NaN	NaN ± NaN	NaN ± NaN	NaN ± NaN	162 ± 11

Read 3 (I)

Lane	Tiles	Density (K/mm2)	Cluster PF (%)	Legacy Phasing/Prephasing Rate	Phasing slope/offset	Prephasing slope/offset	Cluster Count Raw (M)	Cluster Count PF (M)	% >= Q30	Yield (G)	Cycles Err Rated	Aligned (%)	Error Rate (%)	Error Rate 35 cycle (%)	Error Rate 75 cycle (%)	Error Rate 100 cycle (%)	Intensity Cycle 1
1	96	4974 ± 0	86.91 ± 0.60	NaN / NaN	NaN / NaN	NaN / NaN	484.31	420.93	92.98	3.78	0	NaN ± NaN	NaN ± NaN	NaN ± NaN	NaN ± NaN	NaN ± NaN	144 ± 13

Read 4

Lane	Tiles	Density (K/mm2)	Cluster PF (%)	Legacy Phasing/Prephasing Rate	Phasing slope/offset	Prephasing slope/offset	Cluster Count Raw (M)	Cluster Count PF (M)	% >= Q30	Yield (G)	Cycles Err Rated	Aligned (%)	Error Rate (%)	Error Rate 35 cycle (%)	Error Rate 75 cycle (%)	Error Rate 100 cycle (%)	Intensity Cycle 1
1	96	4974 ± 0	86.91 ± 0.60	0.096 / 0.020	0.085 / 0.149	0.041 / 1.003	484.31	420.93	80.01	124.91	0 - 300	4.39 ± 0.46	0.42 ± 0.12	0.14 ± 0.04	0.19 ± 0.05	0.22 ± 0.07	65 ± 7

QC Summary.
Data quality metrics and statistics summarized per lane and per read, provided as means and standard deviations over the tiles used in the lane. Yield Total denotes the total number of bases sequenced, with associated cluster and Q30 metrics reported. Aligned denotes percentage of the run library that aligned to the PhiX genome, which corresponds to amount of PhiX spiked in as quality control sample for the sequencing run.

Figure S1: Variant Annotation

Variant Annotation Report

Input Data

VCF File: bcftools.vcf.gz

Reference Genome: GCA_000001635.9_GRCm39_genomic.fna

Annotation File: genomic.gff

Results

General Information

Statistic	Count
Lines of input read	7044834
Novel / existing variants	0
Overlapped genes	1
Overlapped regulatory features	0
Overlapped transcripts	24
Variants filtered out	0
Variants processed	7044834

Variant Classes

Type of variant	Count (% total)
SNP	5664972 (77.98%)
Substitution	0 (0.00%)
Insertion	597735 (8.23%)
Deletion	672669 (9.26%)
Indel	329448 (4.53%)
Other	0 (0.00%)
**Total**	7264824 (100.00%)

Effect of the Variants

Genetic Consequences

Name	Count (% severe)
Downstream gene variant	16 (0.00%)
Intergenic variant	7264821 (96.97%)
Non coding transcript exon variant	1 (100.00%)
Upstream gene variant	16 (12.50%)
**Total**	7264854 (96.00%)

Genetic Consequences refer to the outcome of the variant at DNA and RNA level.

Because of that, all variants have consequences at that level and they can have more than one consequence.

Coding Consequences

Name	Count (% total)
**Total**	0 (100.00%)

Coding Consequences refer to the effect of the variant at protein level.

Thus, only variants that fall inside conding regions will have a consequence.

Population Genetics

Ts/Tv Ratio

Change	Fraction
AC	0.090
AG	0.324
AT	0.103
CG	0.070
CT	0.323
GT	0.090
Overall Ts/Tv ratio	1.830

Heterozygosis in Samples

Sample	Observed Homozygotes	Expected Homozygotes	Number of Sites	Inbreeding Coefficient (F)
855012_R1	126802	431016.8	1172566	-0.41024
855012_R2	139217	431016.8	1169167	-0.39531

References

- McLaren W., Gil L., Hunt SE., Riat HS., Ritchie GR., Thormann A., Flicek P. and Cunningham F. (2016). The Ensembl Variant Effect Predictor. *Genome biology, 17*(1), 122.
- OmicsBox - Bioinformatics made easy. BioBam Bioinformatics. March 3, 2019. www.biobam.com/omicsbox.

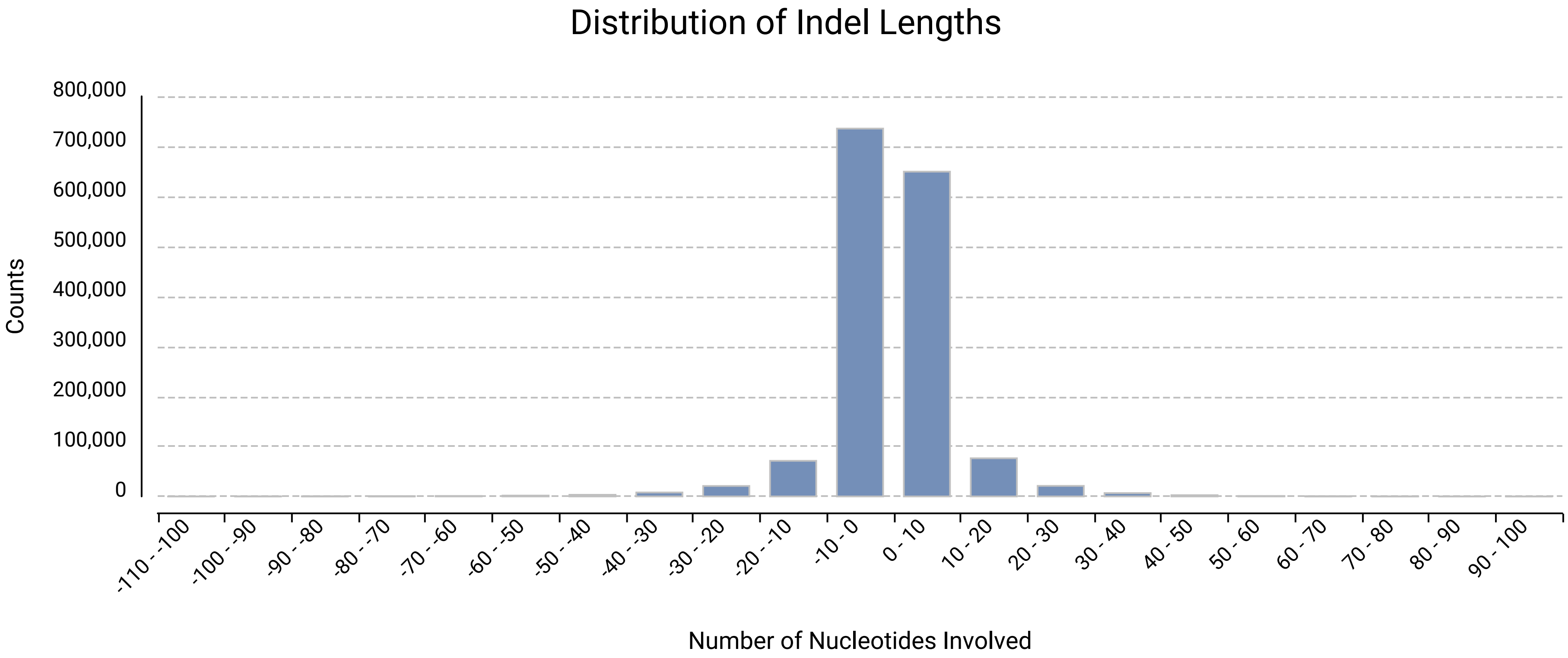

Distribution of Indel Lengths
800,000
700,000
600,000
500,000
400,000
300,000
200,000
100,000
0
Counts
-110 - -100
-100 - -90
-90 - -80
-80 - -70
-70 - -60
-60 - -50
-50 - -40
-40 - -30
-30 - -20
-20 - -10
-10 - 0
0 - 10
10 - 20
20 - 30
30 - 40
40 - 50
50 - 60
60 - 70
70 - 80
80 - 90
90 - 100
Number of Nucleotides Involved

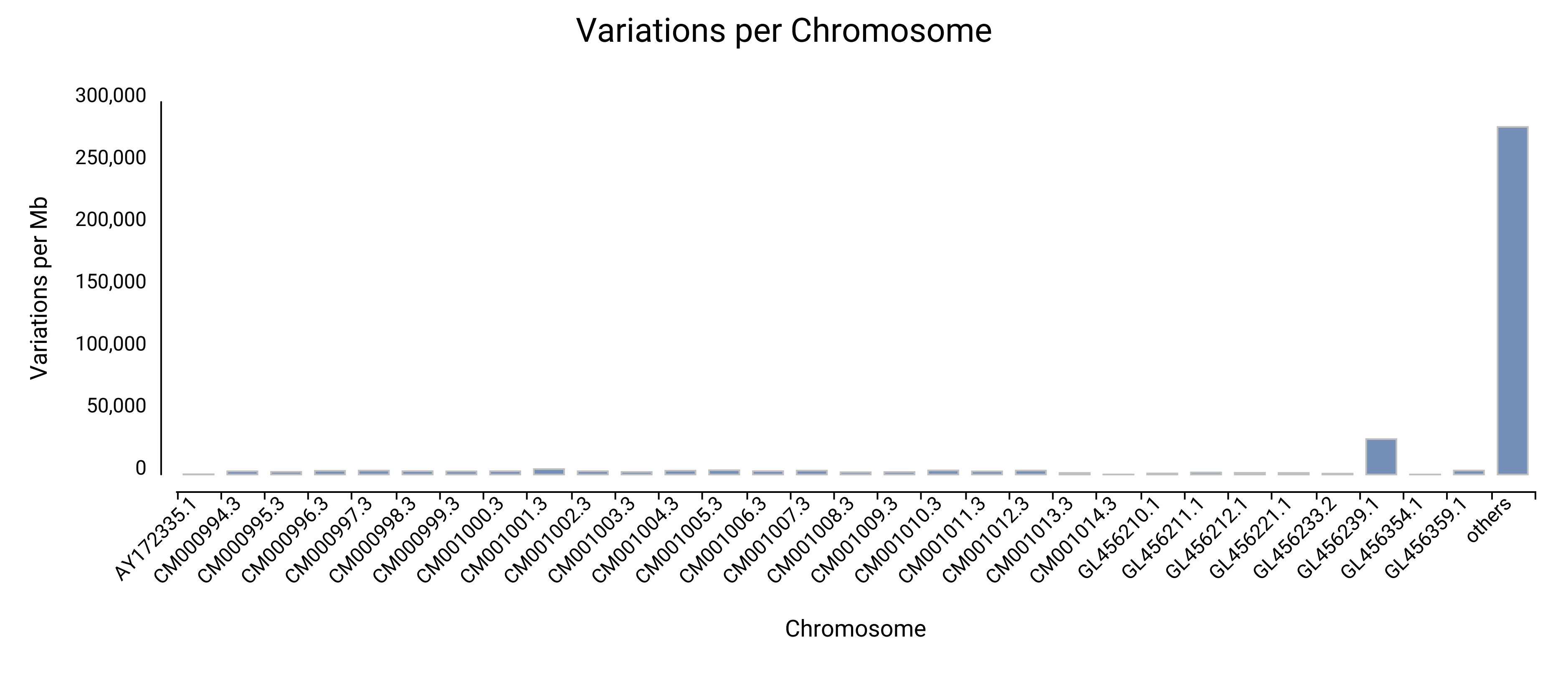
Variations per Chromosome
Variations per Mb
300,000
250,000
200,000
150,000
100,000
50,000
0
AY172335.1
CM000994.3
CM000995.3
CM000996.3
CM000997.3
CM000998.3
CM000999.3
CM001000.3
CM001001.3
CM001002.3
CM001003.3
CM001004.3
CM001005.3
CM001006.3
CM001007.3
CM001008.3
CM001009.3
CM001010.3
CM001011.3
CM001012.3
CM001013.3
CM001014.3
GL456210.1
GL456211.1
GL456212.1
GL456221.1
GL456233.2
GL456239.1
GL456354.1
GL456359.1
others
Chromosome

Figure S1: Variant Calling Report

Variant Calling Report (BCFtools)

Input Data

Reference: GCA_000001635.9_GRCm39_genomic.fna

Number of BAM Files Used: 2

Results

VCF File Saved as: D:\855012 BRCA1 4T1 Research\Analysis\Variant Calling\bcftools.vcf.gz

Type of variant	Frequency
SNP	5627248
INDEL	1417586

Number of alleles in a variant	Frequency
2	6849701
3	170276
4	24857

Statistics	Count (% total)
Number of Variants	7044834
Number of Genotypes	14089668
Number of Heterozygotes	2329553 (16.53%)
Missing Data	500531 (3.55%)

Analysis Parameters

Parameter	Value
Remove Duplicates	true
Adjust Mapping Quality	0
Max. Depth	250
BAQ options	No BAQ
Min. Mapping Quality	0
Min. Base Quality	13
Ignore @RG Tags	false
Extension Error Probability	20
Minimum Fraction of Gapped Reads	0.002
Tandem Quality	500
Skip Indel Calling	false
Gapped Reads for Indel	1
Phred Open Sequencing Error	40
Keep Alternate Alleles	true
Use Groups	false

References

- OmicsBox - Bioinformatics made easy. BioBam Bioinformatics. March 3, 2019. www.biobam.com/omicsbox.
- Danecek P et al. (2021). Twelve years of SAMtools and BCFtools. *GigaScience, 10*(2).

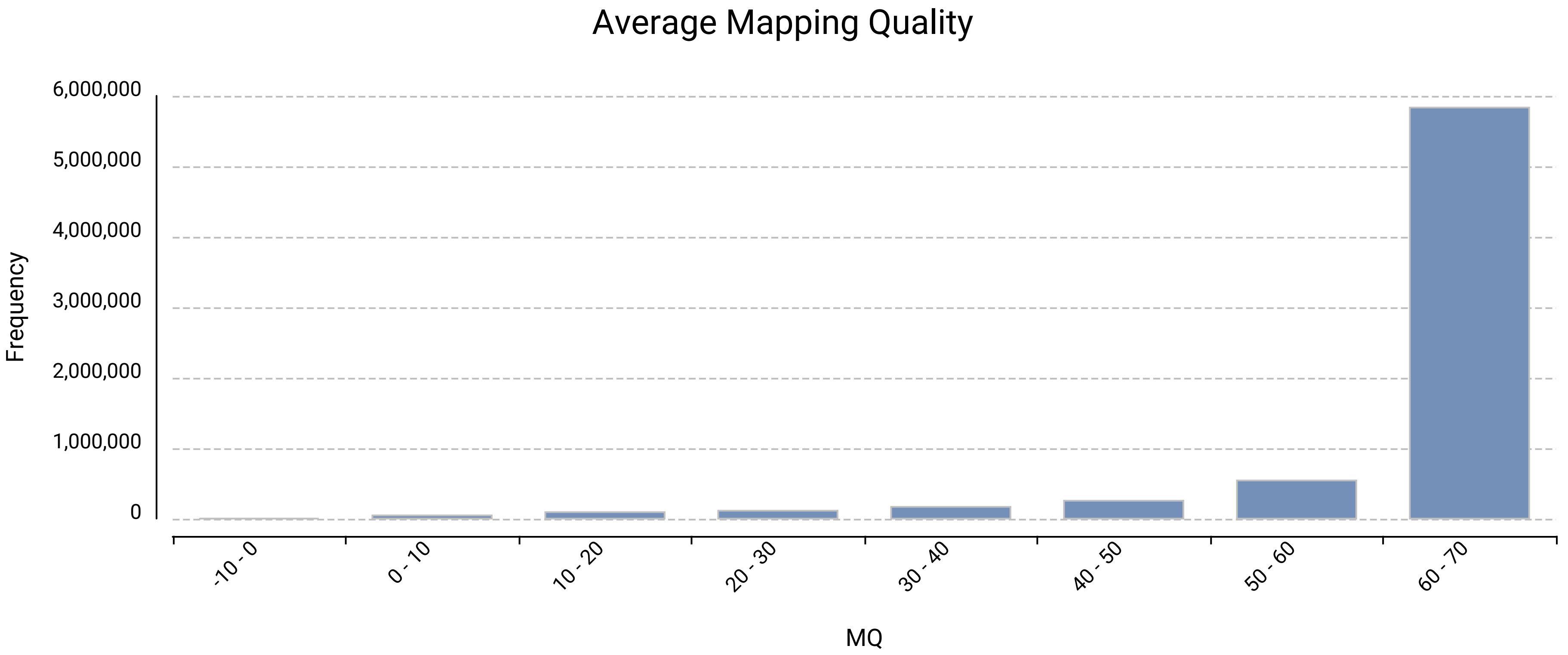
Average Mapping Quality
6,000,000
5,000,000
4,000,000
3,000,000
2,000,000
1,000,000
0
Frequency
-10 - 0
0 - 10
10 - 20
20 - 30
30 - 40
40 - 50
50 - 60
60 - 70
MQ

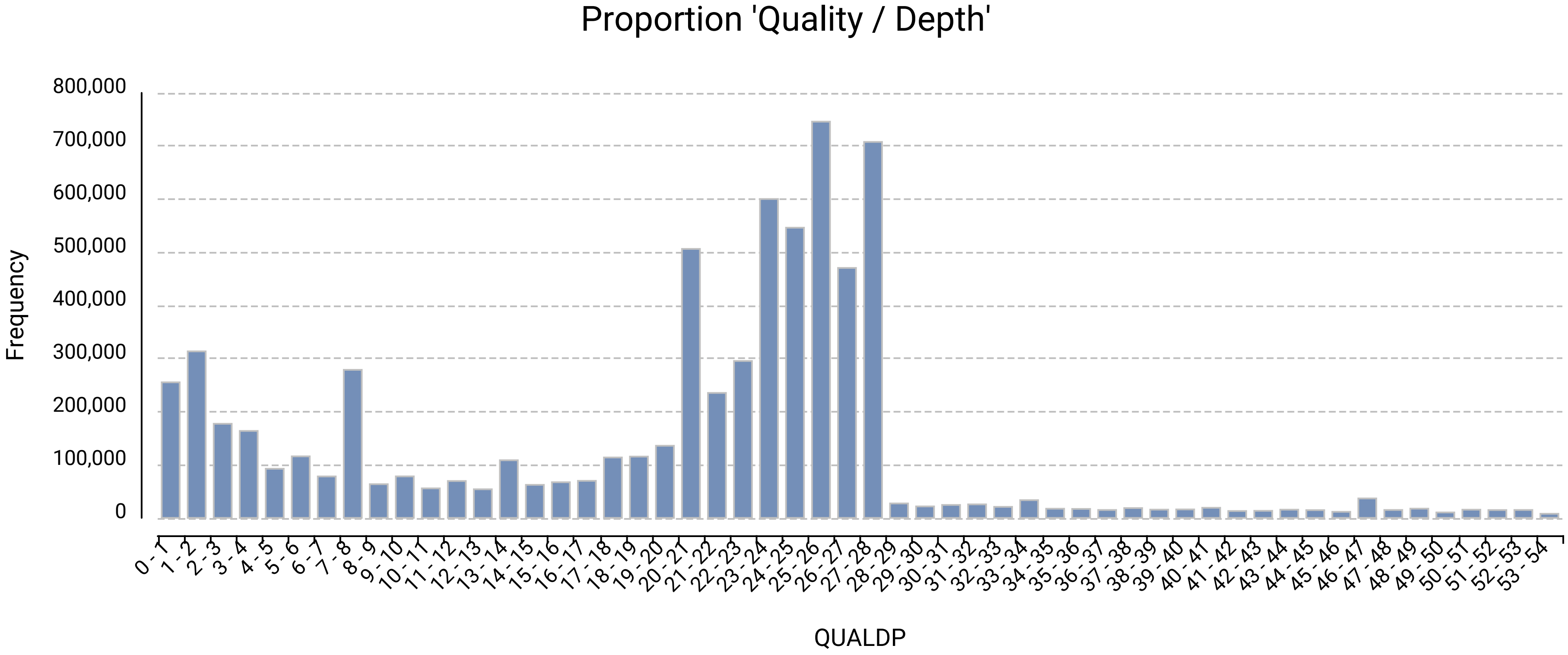

Proportion 'Quality / Depth'
Frequency
800,000
700,000
600,000
500,000
400,000
300,000
200,000
100,000
0
0 - 1
1 - 2
2 - 3
3 - 4
4 - 5
5 - 6
6 - 7
7 - 8
8 - 9
9 - 10
10 - 11
11 - 12
12 - 13
13 - 14
14 - 15
15 - 16
16 - 17
17 - 18
18 - 19
19 - 20
20 - 21
21 - 22
22 - 23
23 - 24
24 - 25
25 - 26
26 - 27
27 - 28
28 - 29
29 - 30
30 - 31
31 - 32
32 - 33
33 - 34
34 - 35
35 - 36
36 - 37
37 - 38
38 - 39
39 - 40
40 - 41
41 - 42
42 - 43
43 - 44
44 - 45
45 - 46
46 - 47
47 - 48
48 - 49
49 - 50
50 - 51
51 - 52
52 - 53
53 - 54
QUALDP

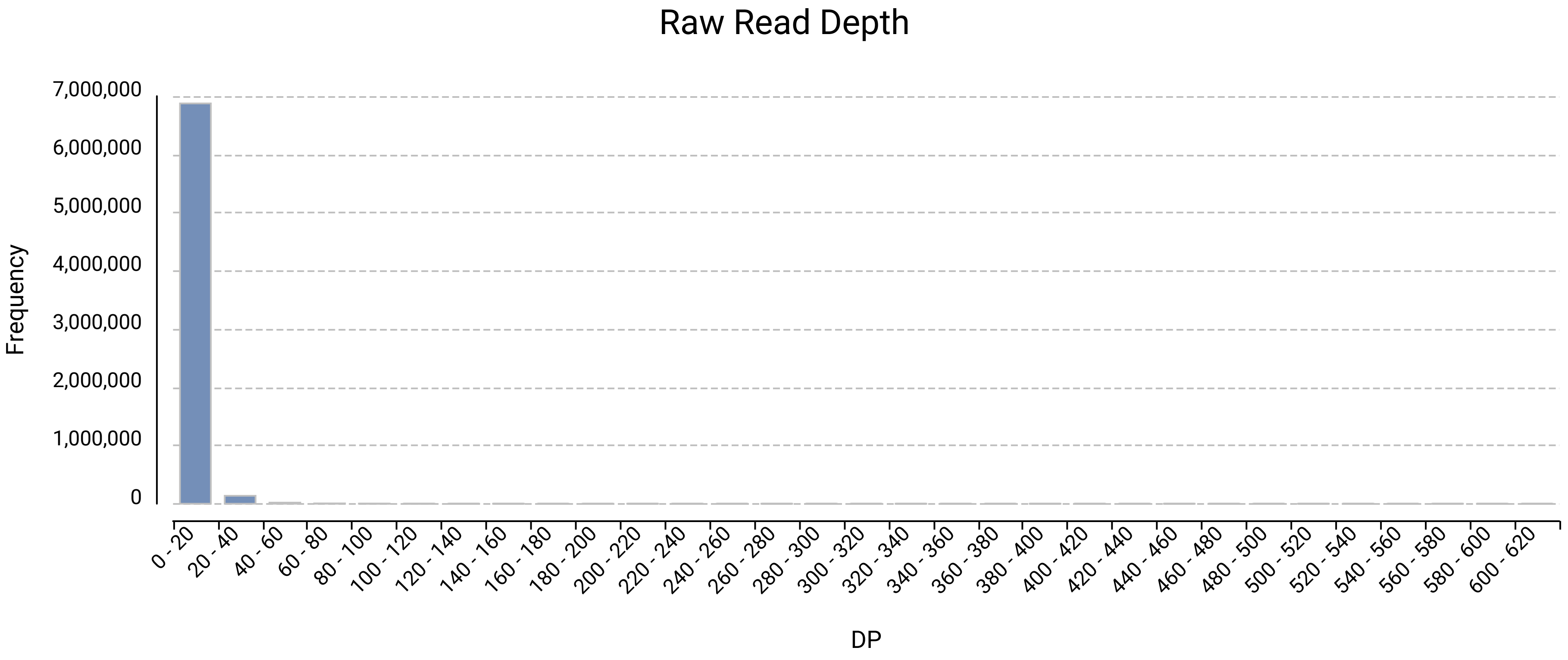

Raw Read Depth
Frequency
7,000,000
6,000,000
5,000,000
4,000,000
3,000,000
2,000,000
1,000,000
0
0 - 20
20 - 40
40 - 60
60 - 80
80 - 100
100 - 120
120 - 140
140 - 160
160 - 180
180 - 200
200 - 220
220 - 240
240 - 260
260 - 280
280 - 300
300 - 320
320 - 340
340 - 360
360 - 380
380 - 400
400 - 420
420 - 440
440 - 460
460 - 480
480 - 500
500 - 520
520 - 540
540 - 560
560 - 580
580 - 600
600 - 620
DP
