# Supplementary material for: Berry-derived gold nanoparticles induce integrated ROS-mediated apoptosis, immune modulation, and transcriptomic remodeling in 4T1 triple-negative cancer cells
Source: Cell Death Discov. 2026 Apr 10;12:225. doi: 10.1038/s41420-026-03023-z (PMC13184259; doi:10.1038/s41420-026-03023-z)
Supplement: Supplementary file 2 — Figure S2 [file 41420_2026_3023_MOESM2_ESM.pdf]

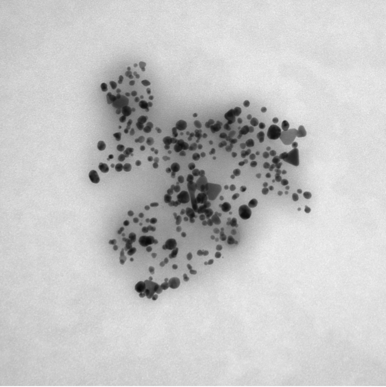

blackberry_03.tif
blackberry
Cal: 2.76pix/nm
13:15 05/23/25
TEM Mode: Imaging
Microscopist: JRT

100 nm
HV=80.0kV
Direct Mag: 170000x
CMIF OSU

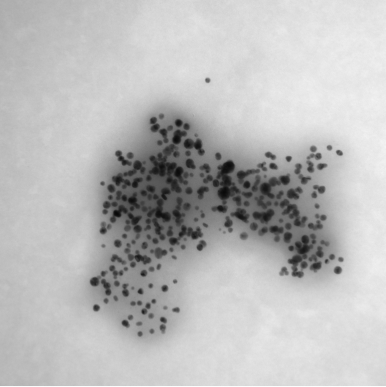

blackberry_04.tif
blackberry
Cal: 2.76pix/nm
13:18 05/23/25
TEM Mode: Imaging
Microscopist: JRT

100 nm
HV=80.0kV
Direct Mag: 170000x
CMIF OSU

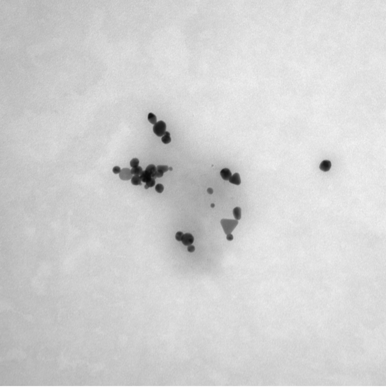

blackberry_05.tif
blackberry
Cal: 2.76pix/nm
13:19 05/23/25
TEM Mode: Imaging
Microscopist: JRT

100 nm
HV=80.0kV
Direct Mag: 170000x
CMIF OSU

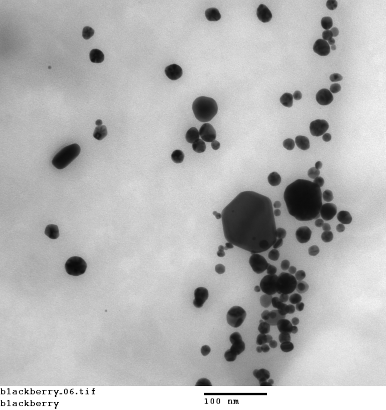

blackberry_06.tif
blackberry
Cal: 2.76pix/nm
13:23 05/23/25
TEM Mode: Imaging
Microscopist: JRT

HV=80.0kV
Direct Mag: 170000x
CMIF OSU

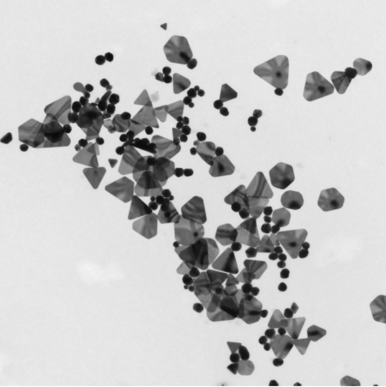

blueberry_01.tif
blueberry
Cal: 1.116pix/nm
9:23 05/23/25
TEM Mode: Imaging
Microscopist: JRT

100 nm
HV=80.0kV
Direct Mag: 68000x
CMIF OSU

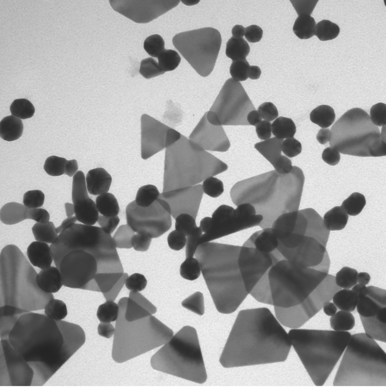

blueberry_02.tif
blueberry
Cal: 2.76pix/nm
9:27 05/23/25
TEM Mode: Imaging
Microscopist: JRT

100 nm
HV=80.0kV
Direct Mag: 170000x
CMIF OSU

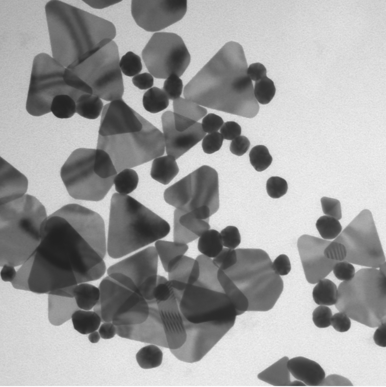

blueberry_03.tif
blueberry
Cal: 2.76pix/nm
9:26 05/23/25
TEM Mode: Imaging
Microscopist: JRT

100 nm
HV=80.0kV
Direct Mag: 170000x
CMIF OSU

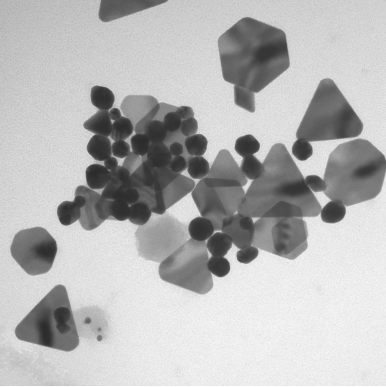

blueberry_04.tif
blueberry
Cal: 2.76pix/nm
9:28 05/23/25
TEM Mode: Imaging
Microscopist: JRT

100 nm
HV=80.0kV
Direct Mag: 170000x
CMIF OSU

UPLC-QQQ/MS Extracted Ion Chromatogram for all the 15 identified analytes

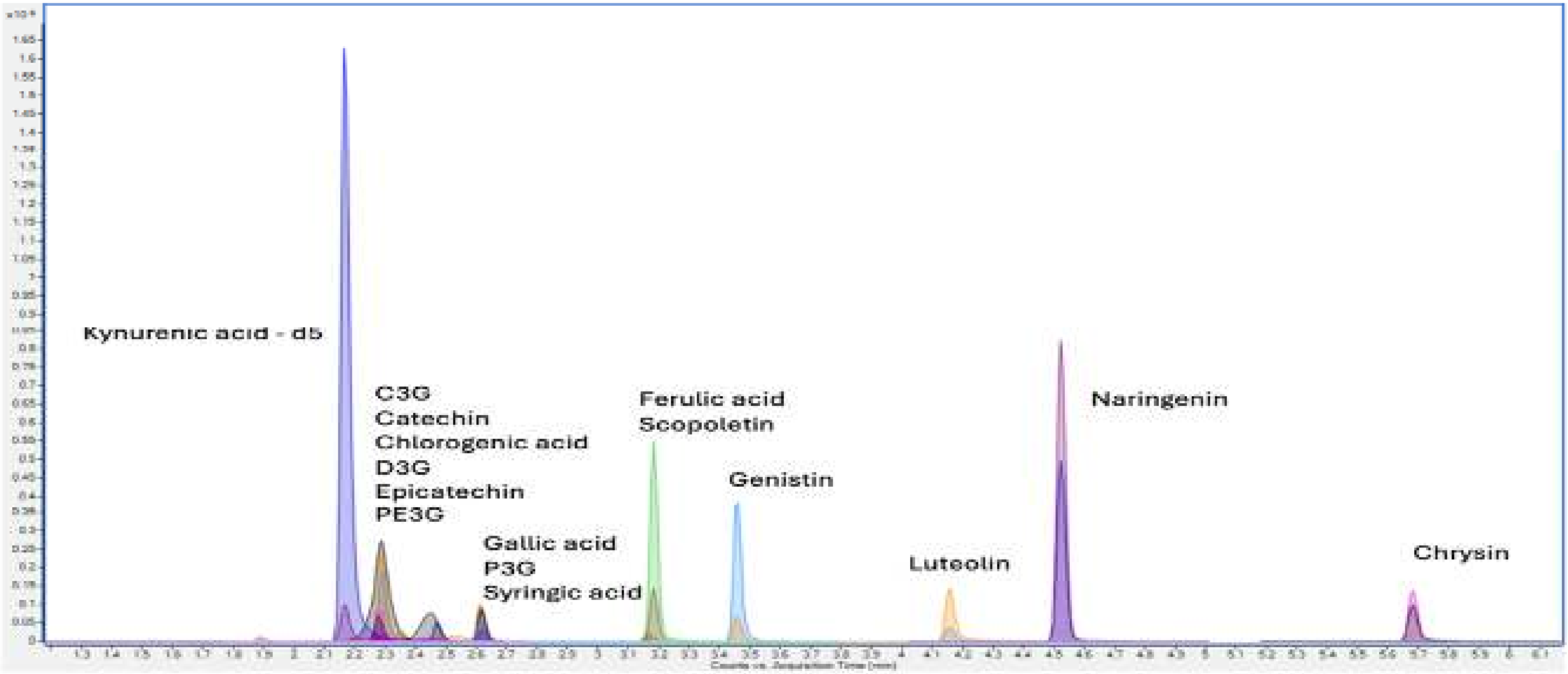

Fig. S2: MRM transitions and optimized collision energy for targeted 15 compounds.

Compound	precursor ion in m/z	product ions in m/z (Collision energy, V)
**Catechin**	289.1	245.0 (15), 109.0 (28)
**Gallic acid**	171	72.8 (16), 57.0 (20)
**PE3G**	433.1	271.0 (20), 93.0 (80)
**Ferulic acid**	195.1	177.9 (12), 134.0 (16)
**Scopoletin**	191	176.0 (16), 104.0 (28)
**Genistin**	433.1	268.0 (38), 43.0 (65)
**Luteolin**	285	133.0 (42), 132.0 (62)
**Chrysin**	253.1	143.0 (16), 62.9 (36)
**Naringenin**	271.1	150.9 (20), 119.0 (32)
**Syringic acid**	197.1	182.0 (12), 123.0 (24)
**P3G**	463.1	301.0 (20), 201.0 (76)
**Chlorogenic acid**	355.1	163.0 (12), 89.0 (68)
**Epicatechin**	289.1	245.0 (15), 109.0 (28)
**D3G**	465.1	303.0 (20), 229.0 (64)
**C3G**	449.1	287.0 (20), 137.0 (68)
**Kynurenic acid - d5**	195.1	149.1 (48), 121.1 (37)

Limit of detection for all the analytes

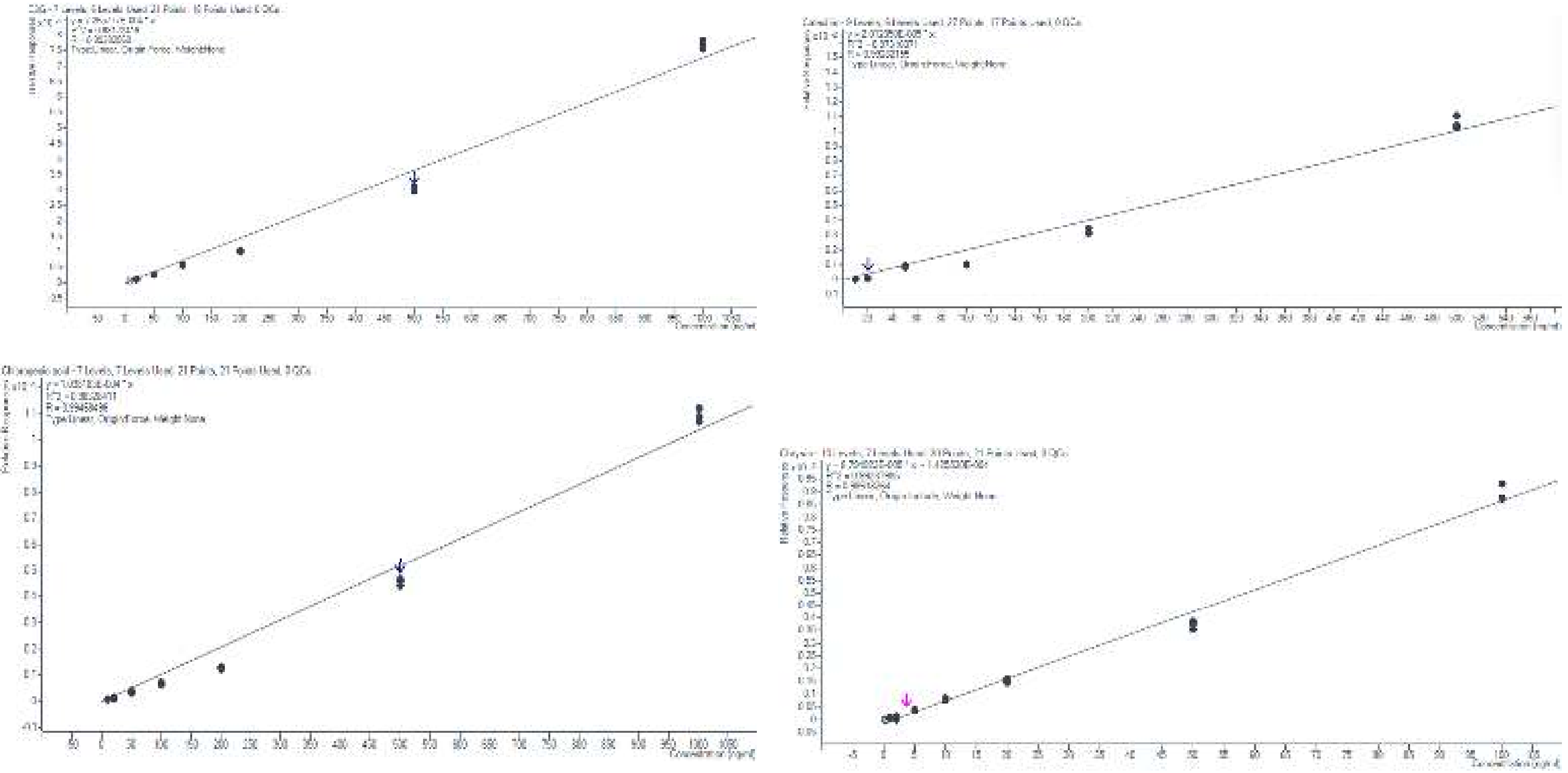

Limit of detection for all the analytes

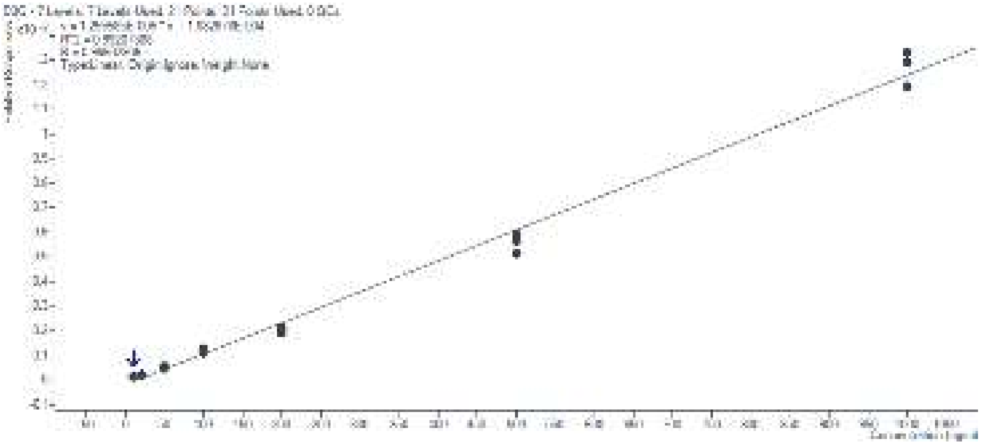

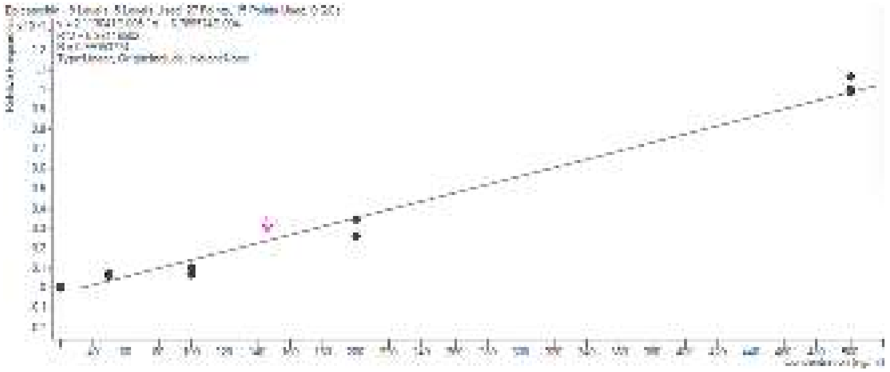

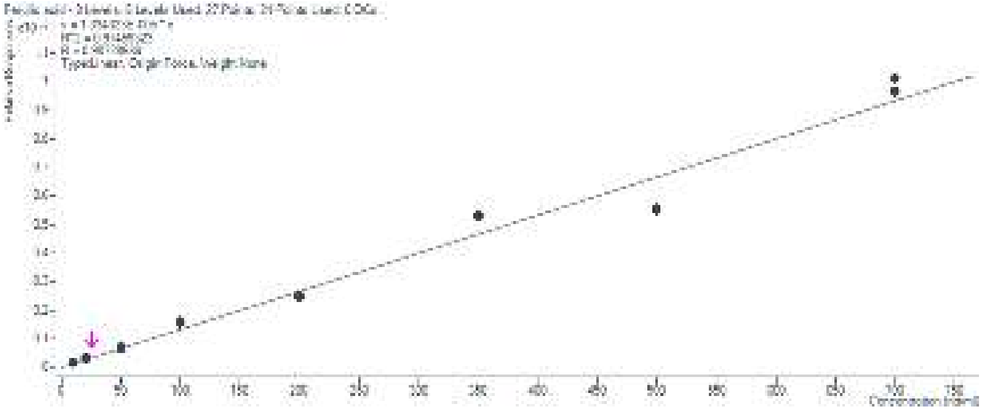

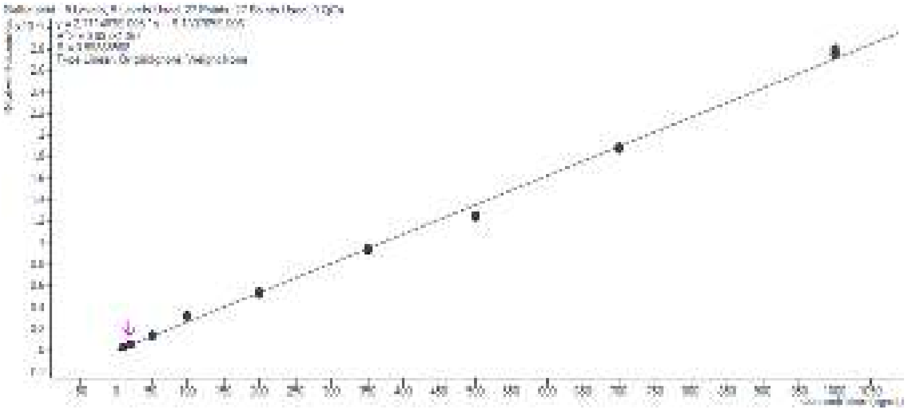

Limit of detection for all the analytes

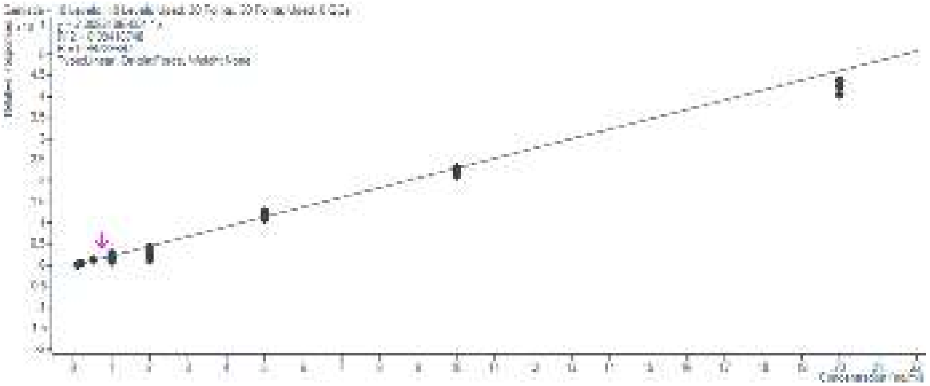

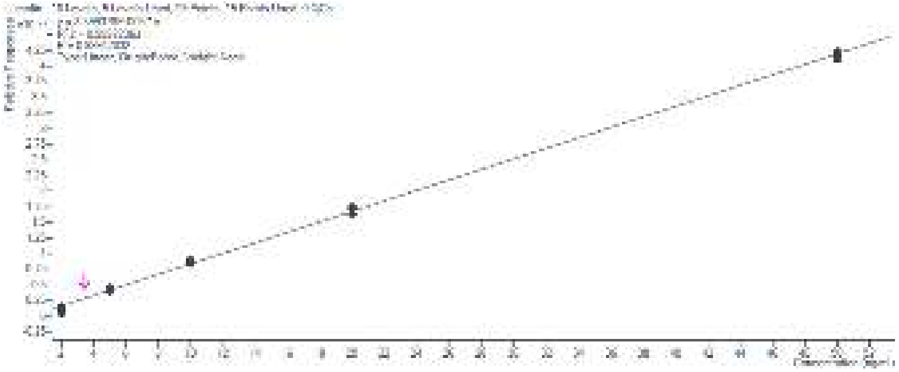

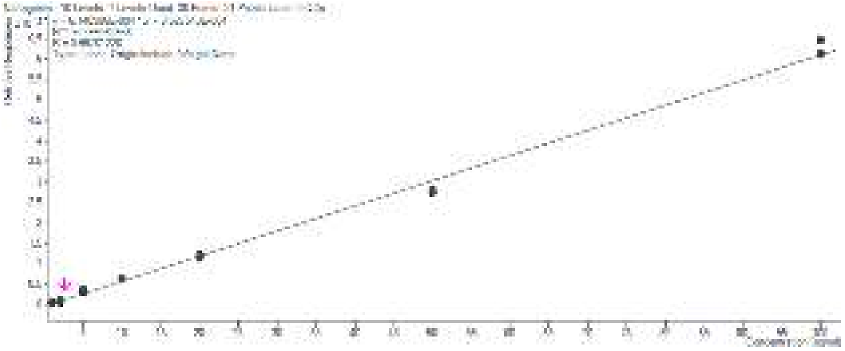

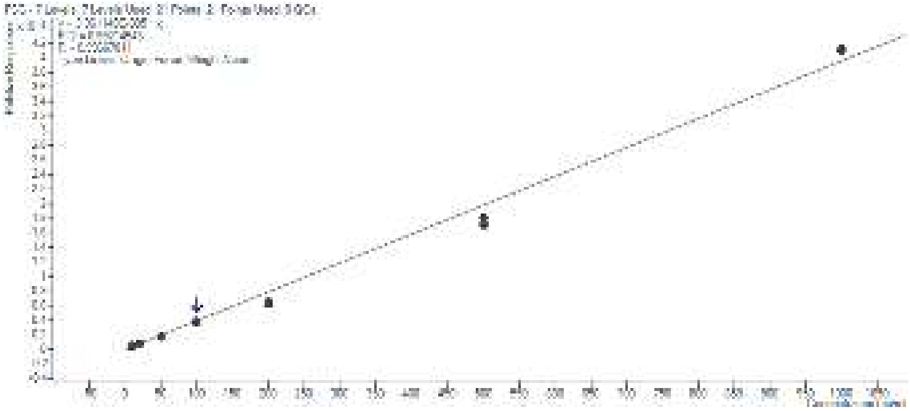

Limit of detection for all the analytes

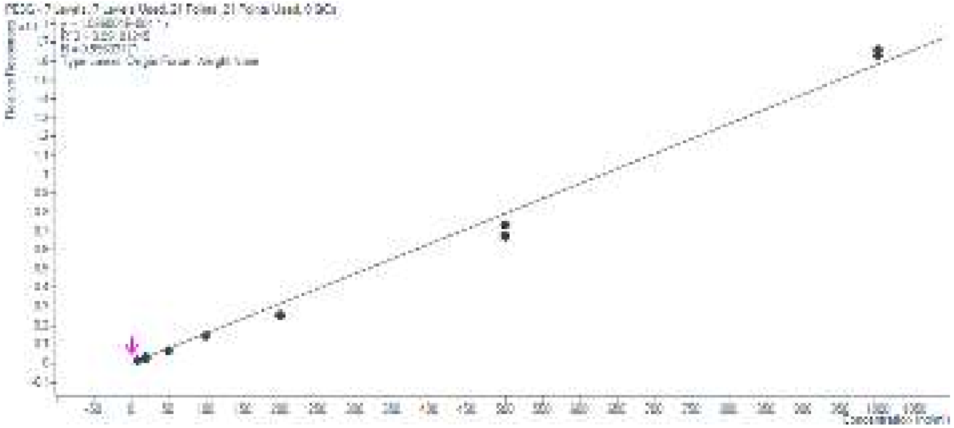

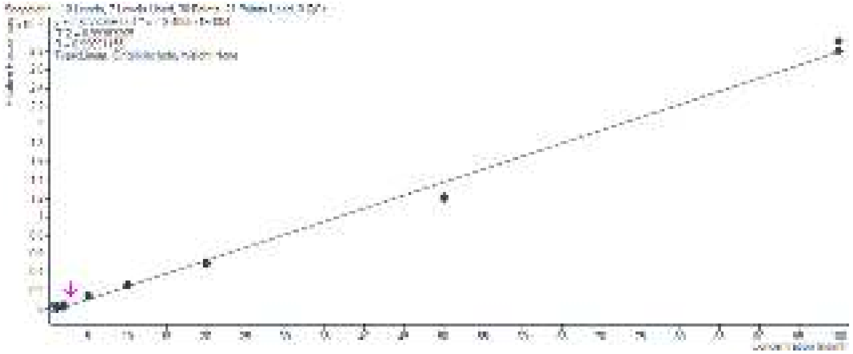

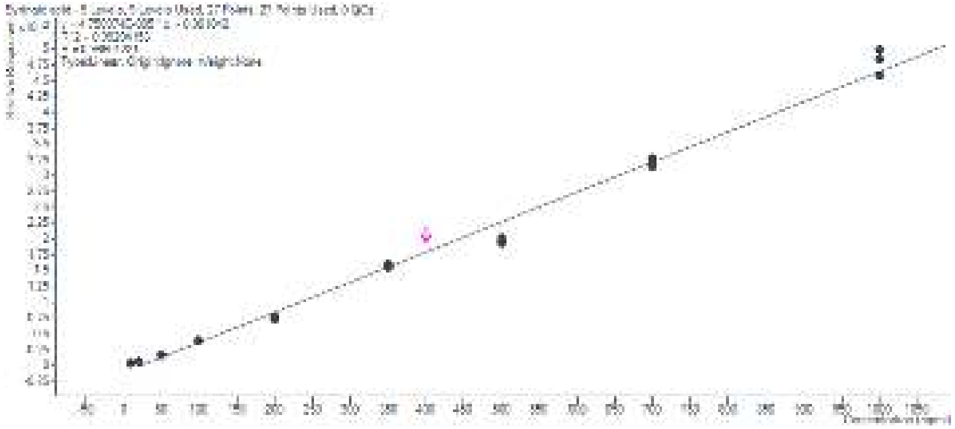

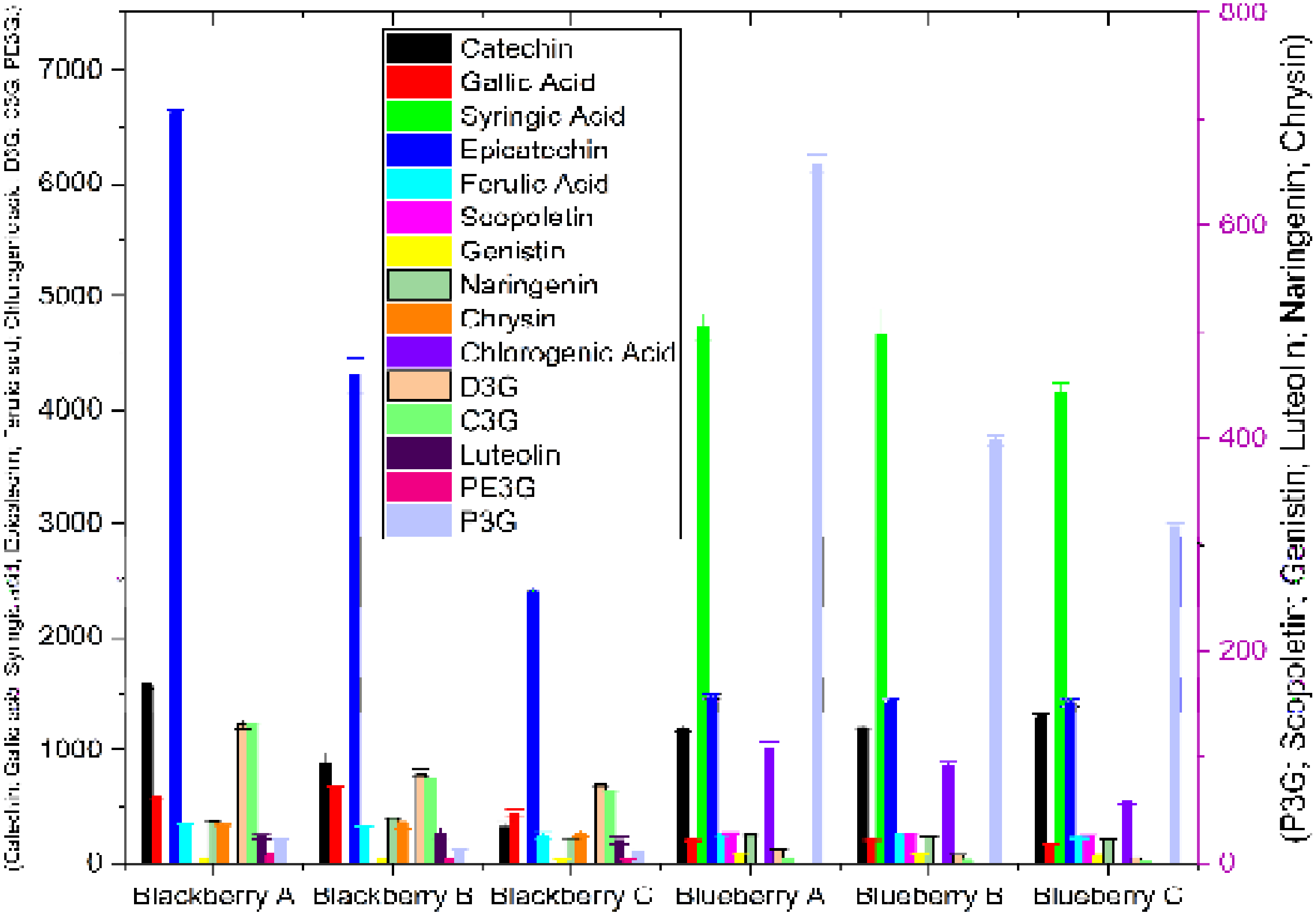

7000
6000
5000
4000
3000
2000
1000
0
800
600
400
200
0
Catechin
Gallic Acid
Syringic Acid
Epicatechin
Ferulic Acid
Scopoletin
Genistin
Naringenin
Chrysin
Chlorogenic Acid
D3G
C3G
Luteolin
PE3G
P3G
Blackberry A
Blackberry B
Blackberry C
Blueberry A
Blueberry B
Blueberry C
(P3G; Scopoletin; Genistin; Luteolin; Naringenin; Chrysin)

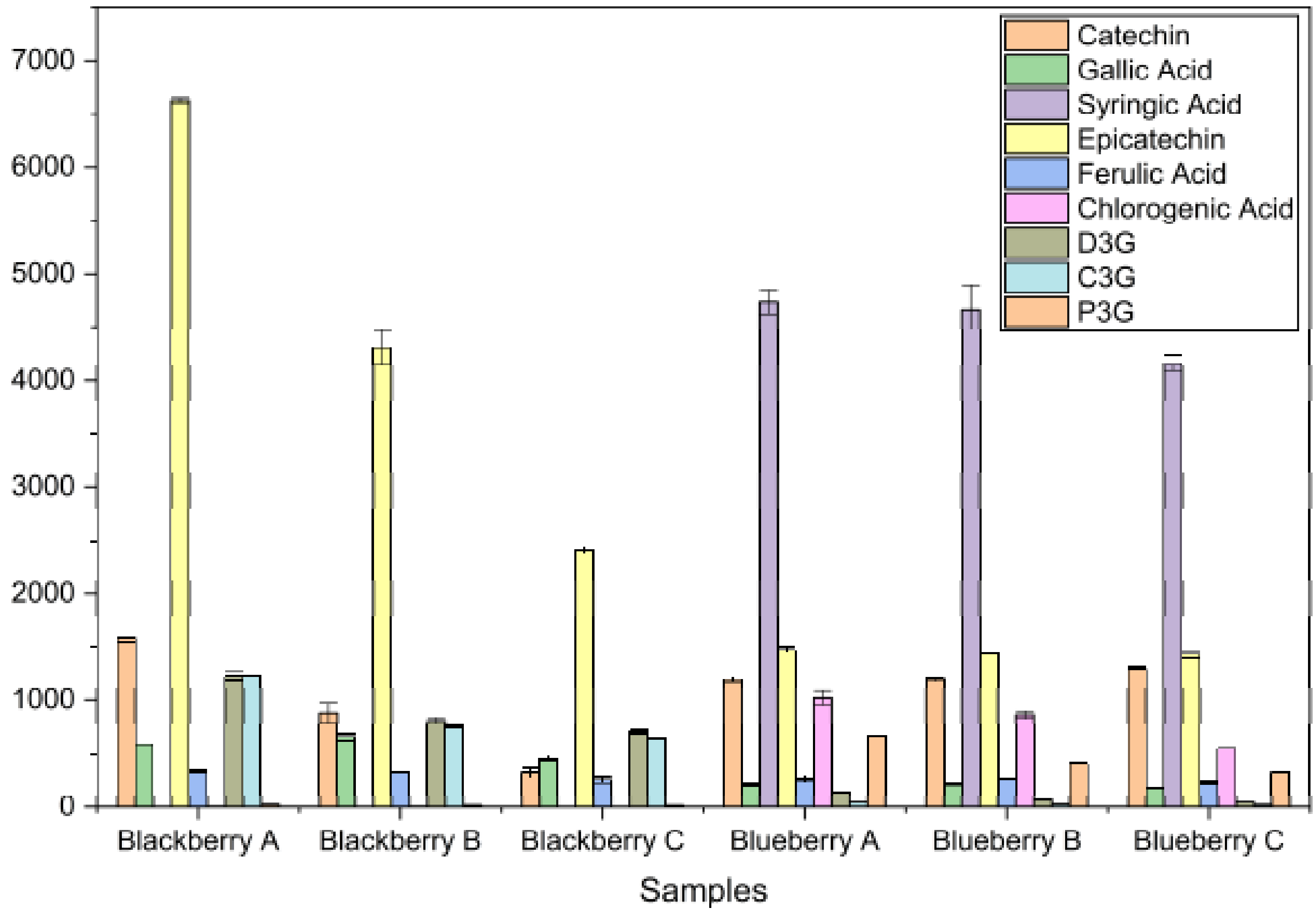

Catechin
Gallic Acid
Syringic Acid
Epicatechin
Ferulic Acid
Chlorogenic Acid
D3G
C3G
P3G
0
1000
2000
3000
4000
5000
6000
7000
Blackberry A
Blackberry B
Blackberry C
Blueberry A
Blueberry B
Blueberry C
Samples

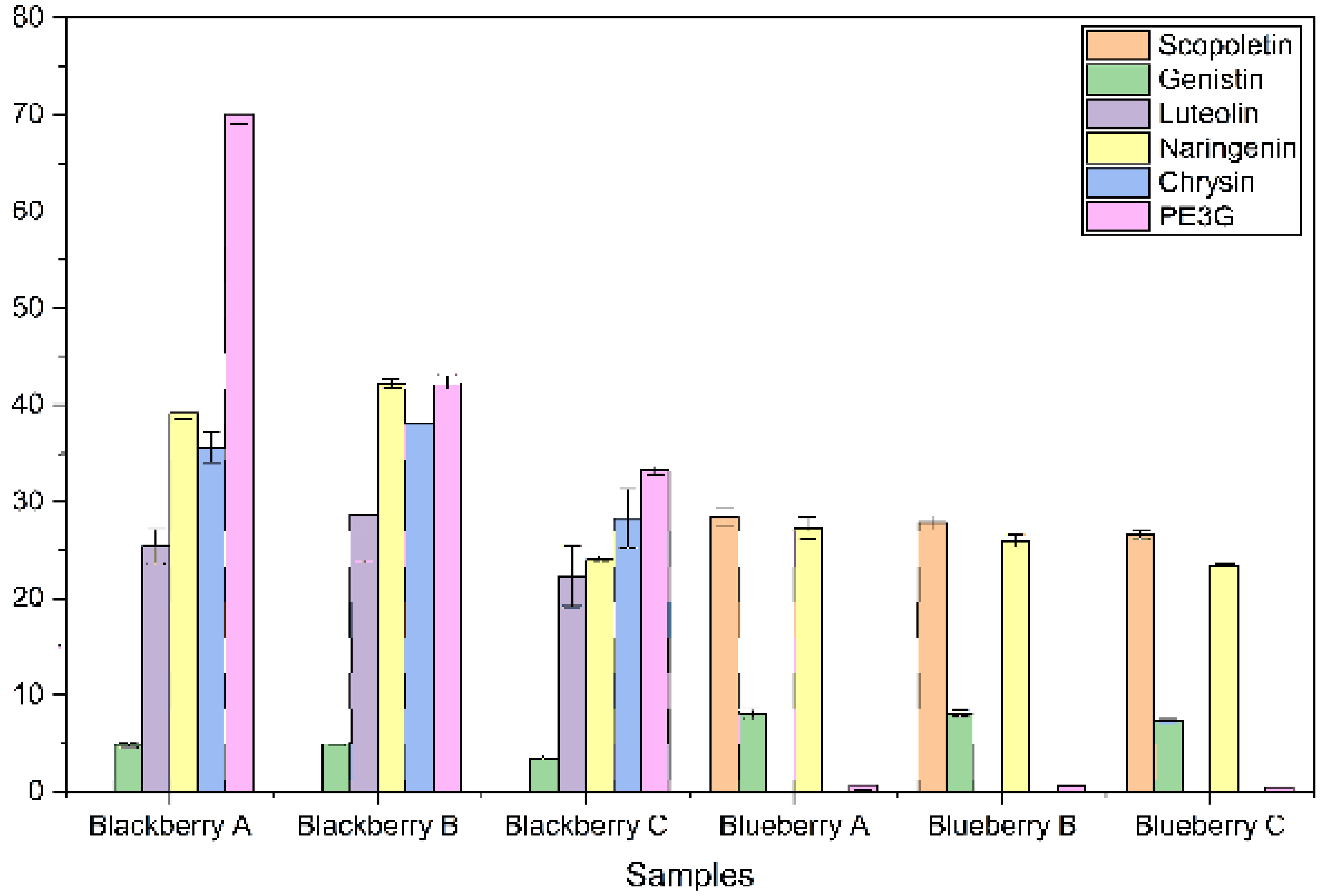

0
10
20
30
40
50
60
70
80
Scopoletin
Genistin
Luteolin
Naringenin
Chrysin
PE3G
Blackberry A
Blackberry B
Blackberry C
Blueberry A
Blueberry B
Blueberry C
Samples

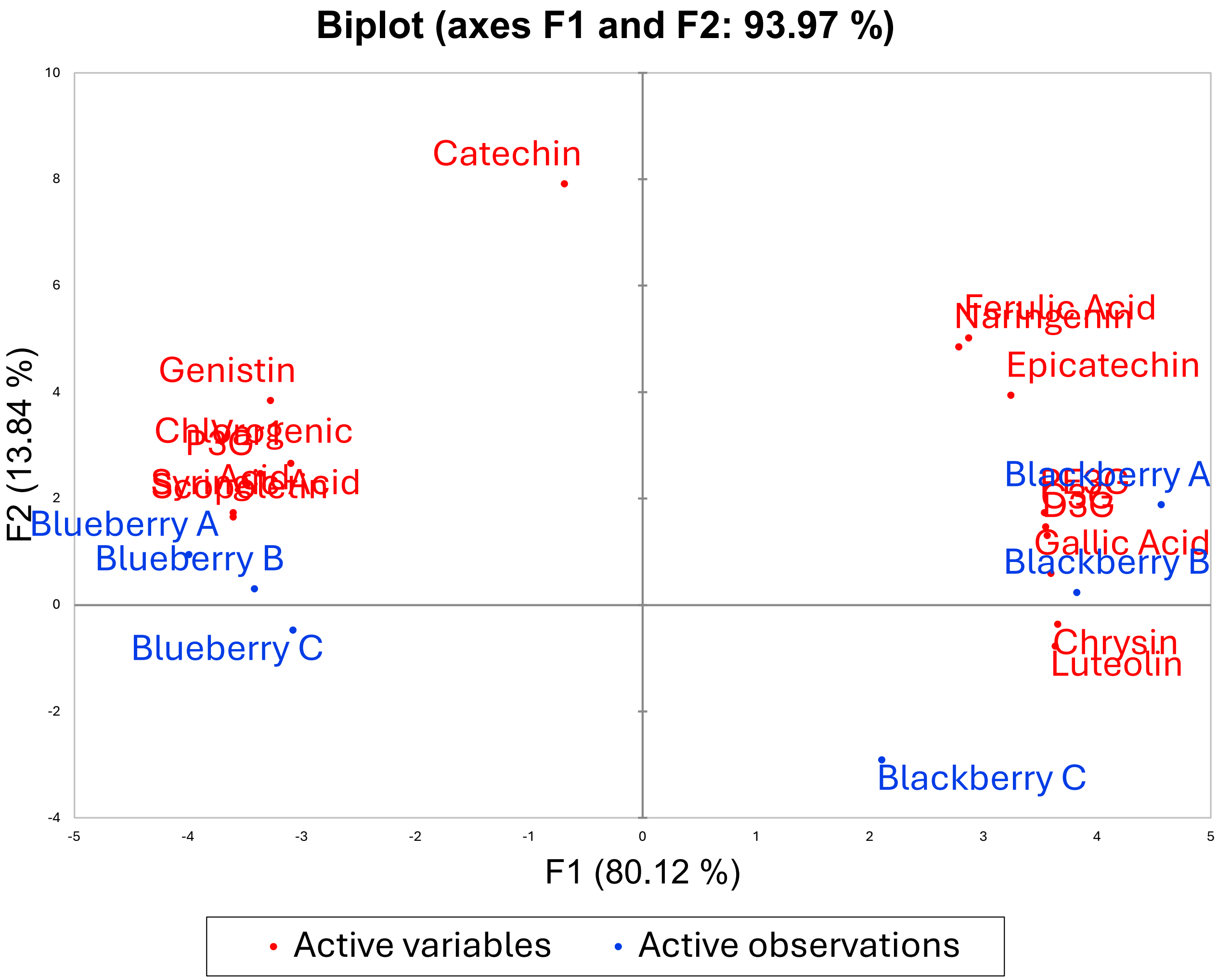

Biplot (axes F1 and F2: 93.97 %)
Catechin
Genistin
Ferulic Acid
Naringenin
Epicatechin
Blueberry A
Blueberry B
Blueberry C
Blackberry A
Blackberry B
Blackberry C
Gallic Acid
Chrysin
Luteolin
F1 (80.12 %)
F2 (13.84 %)
Active variables
Active observations

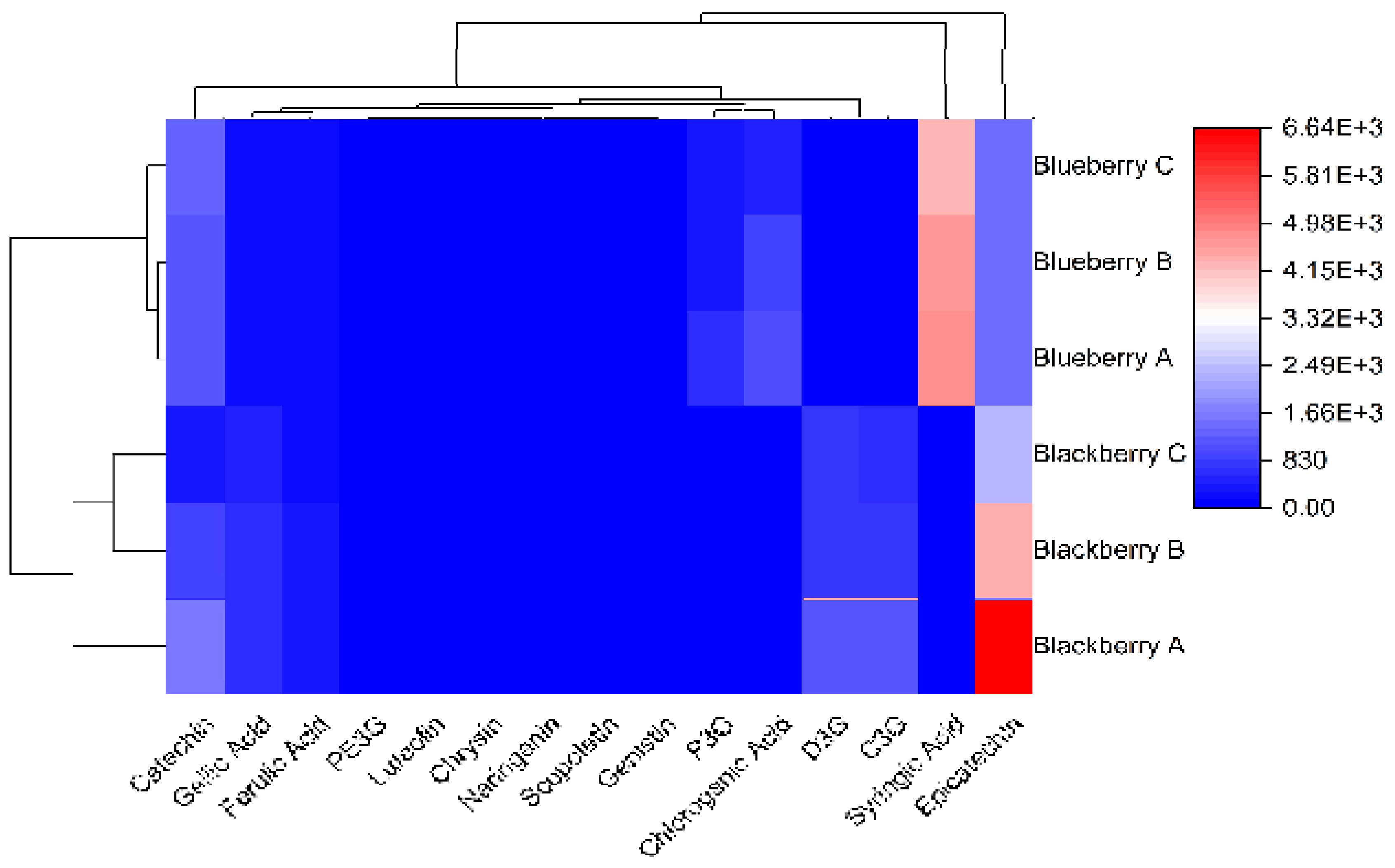

Blueberry C
Blueberry B
Blueberry A
Blackberry C
Blackberry B
Blackberry A
6.64E+3
5.81E+3
4.98E+3
4.15E+3
3.32E+3
2.49E+3
1.66E+3
830
0.00
Catechin
Gallic Acid
Ferulic Acid
PE3G
Luteolin
Chrysin
Naringenin
Scopoletin
Genistin
P3G
Chlorogenic Acid
D3G
C3G
Syringic Acid
Epicatechin

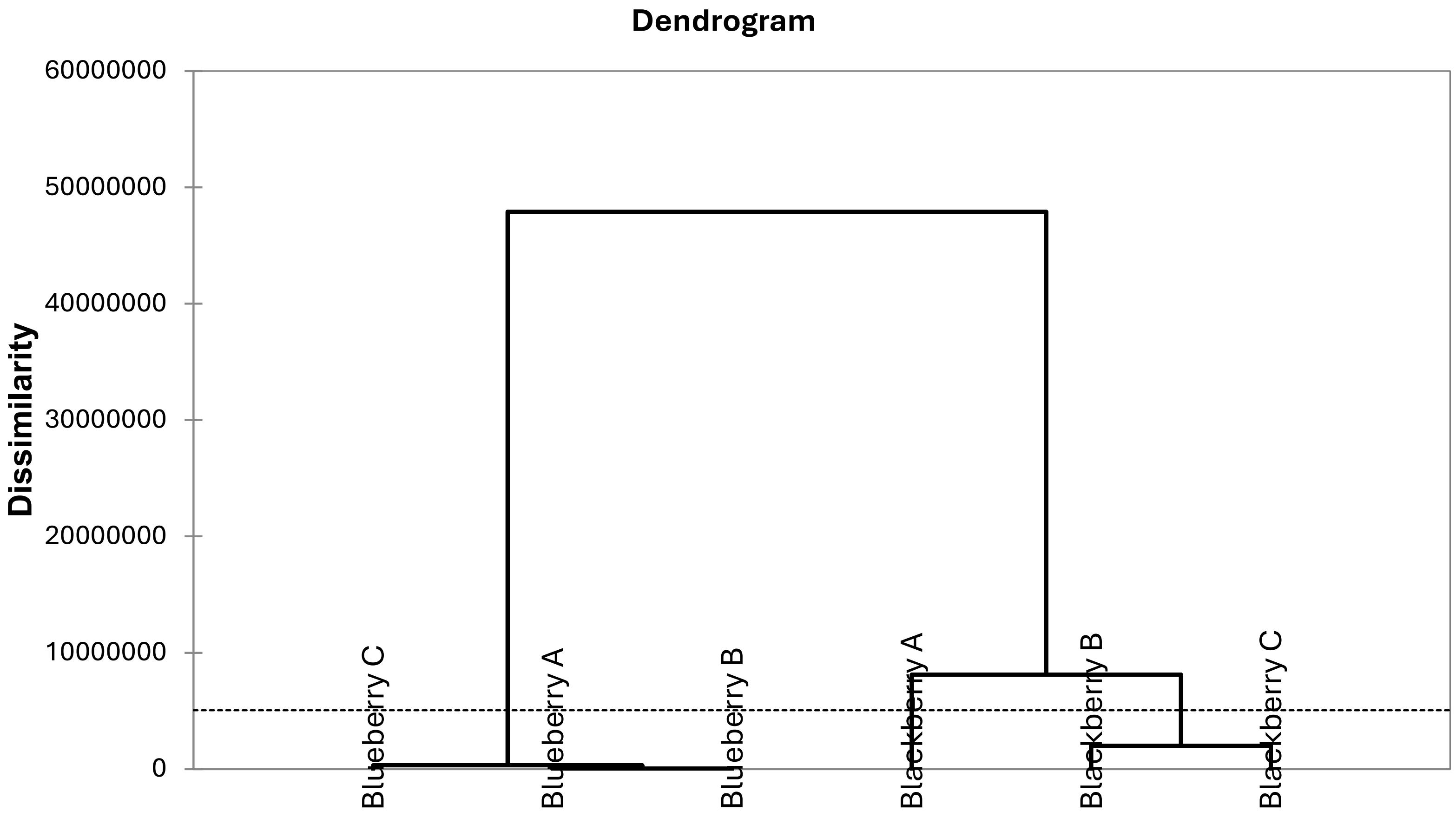

Dendrogram
Dissimilarity
60000000
50000000
40000000
30000000
20000000
10000000
0
Blueberry C
Blueberry A
Blueberry B
Blackberry A
Blackberry B
Blackberry C

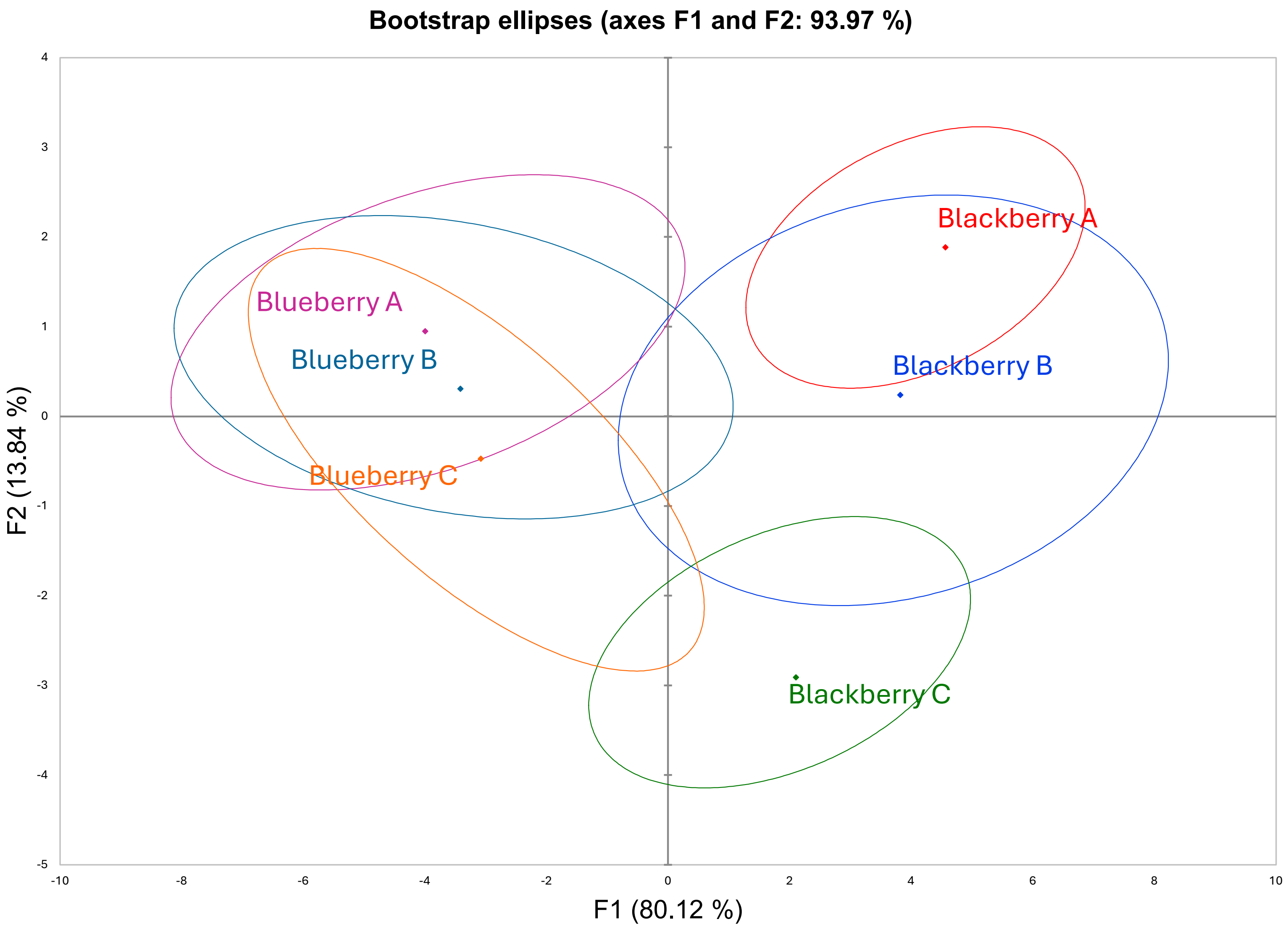

Bootstrap ellipses (axes F1 and F2: 93.97 %)
F1 (80.12 %)
F2 (13.84 %)
Blueberry A
Blueberry B
Blueberry C
Blackberry A
Blackberry B
Blackberry C
-10
-8
-6
-4
-2
0
2
4
6
8
10
4
3
2
1
0
-1
-2
-3
-4
-5

UPLC-QQQ/MS Extracted Ion Chromatogram for all the 15 identified analytes

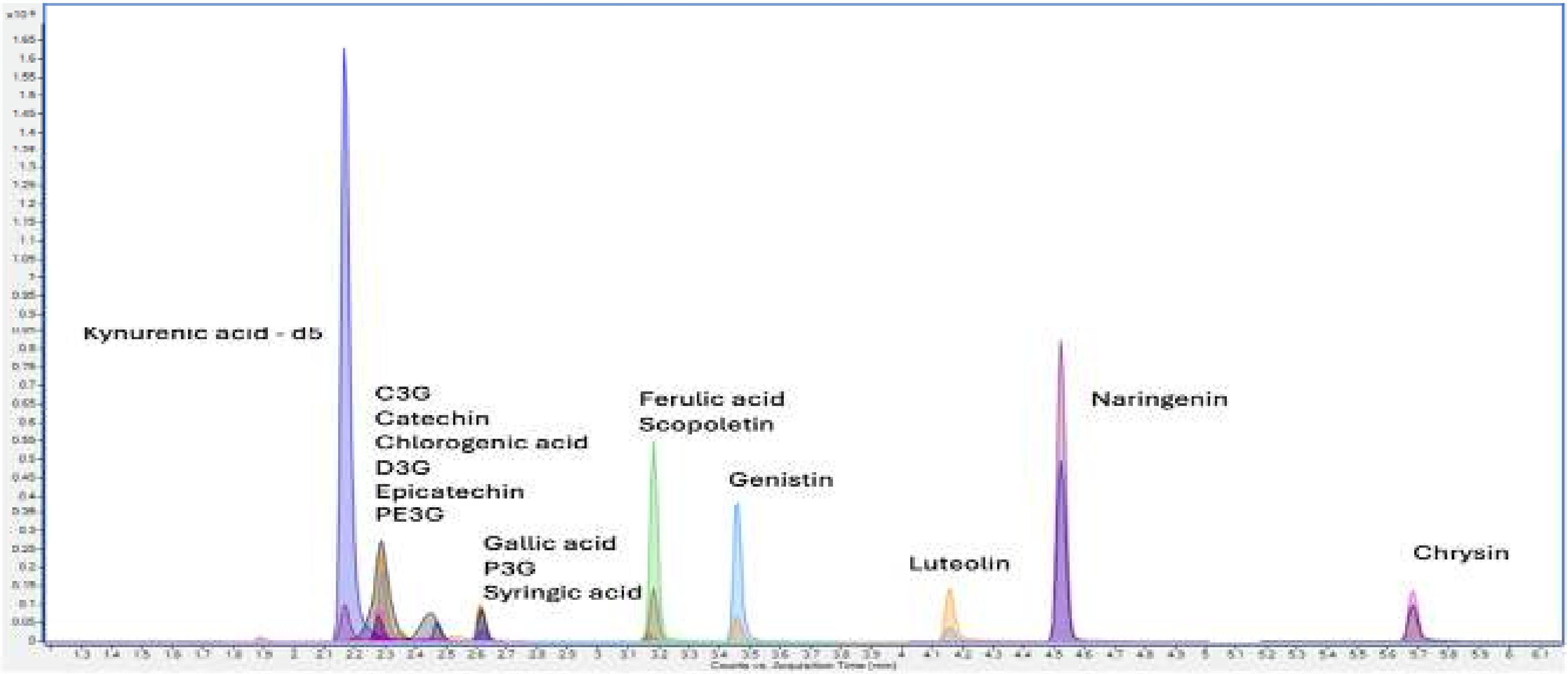

Fig. S2: MRM transitions and optimized collision energy for targeted 15 compounds.

Compound	precursor ion in m/z	product ions in m/z (Collision energy, V)
Catechin	289.1	245.0 (15), 109.0 (28)
Gallic acid	171	72.8 (16), 57.0 (20)
PE3G	433.1	271.0 (20), 93.0 (80)
Ferulic acid	195.1	177.9 (12), 134.0 (16)
Scopoletin	191	176.0 (16), 104.0 (28)
Genistin	433.1	268.0 (38), 43.0 (65)
Luteolin	285	133.0 (42), 132.0 (62)
Chrysin	253.1	143.0 (16), 62.9 (36)
Naringenin	271.1	150.9 (20), 119.0 (32)
Syringic acid	197.1	182.0 (12), 123.0 (24)
P3G	463.1	301.0 (20), 201.0 (76)
Chlorogenic acid	355.1	163.0 (12), 89.0 (68)
Epicatechin	289.1	245.0 (15), 109.0 (28)
D3G	465.1	303.0 (20), 229.0 (64)
C3G	449.1	287.0 (20), 137.0 (68)
Kynurenic acid - d5	195.1	149.1 (48), 121.1 (37)

Limit of detection for all the analytes

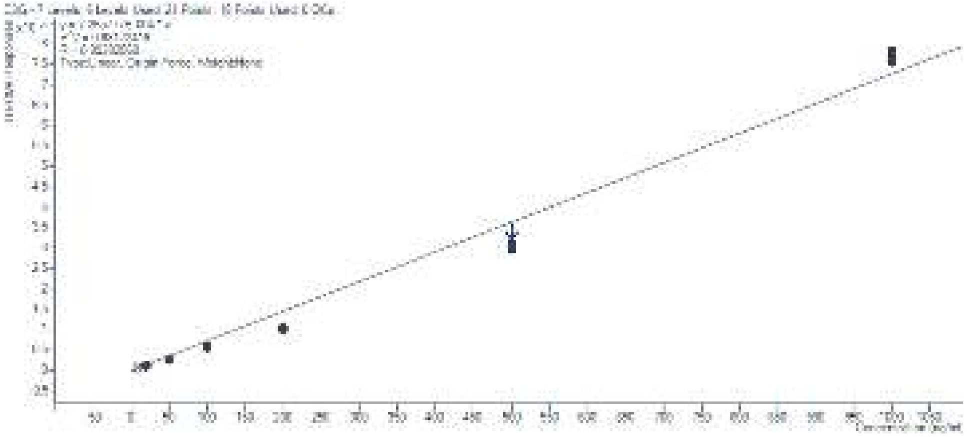

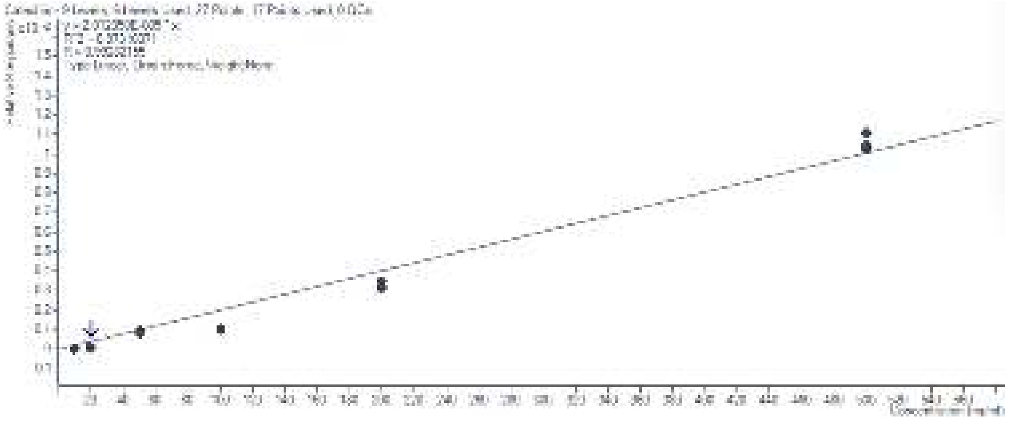

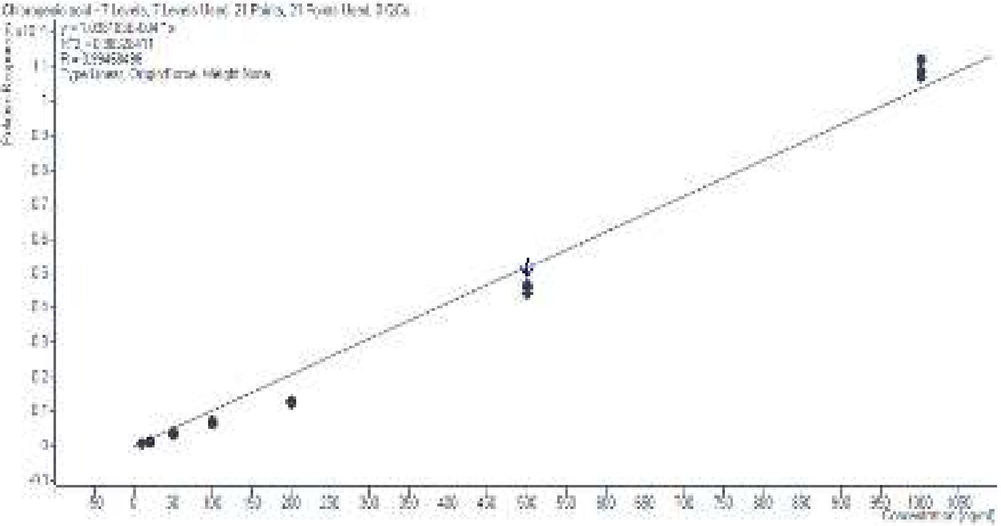

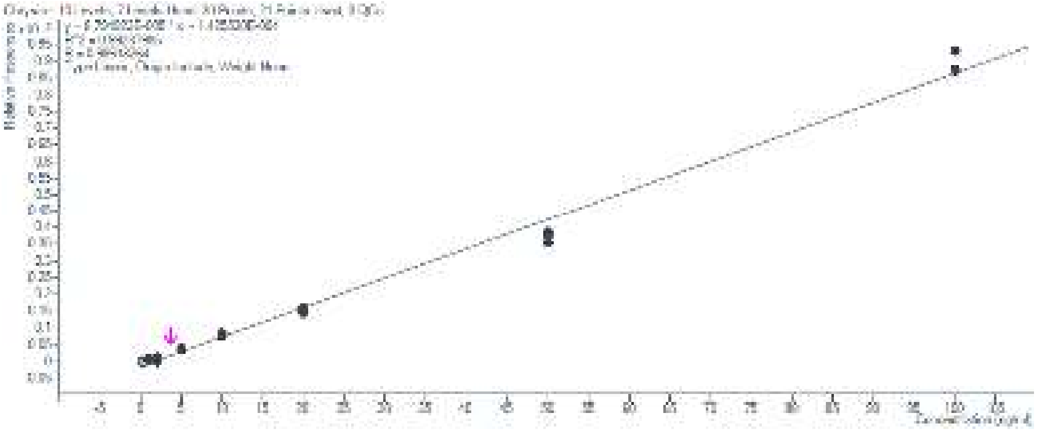

Limit of detection for all the analytes

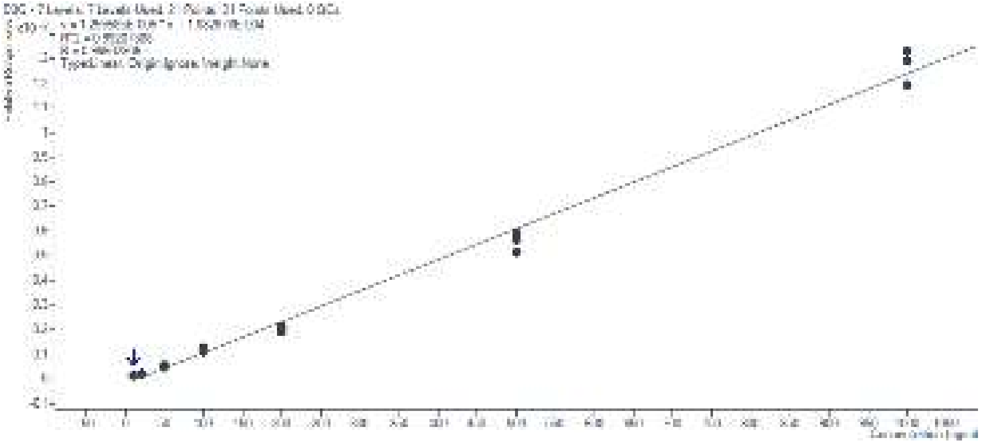

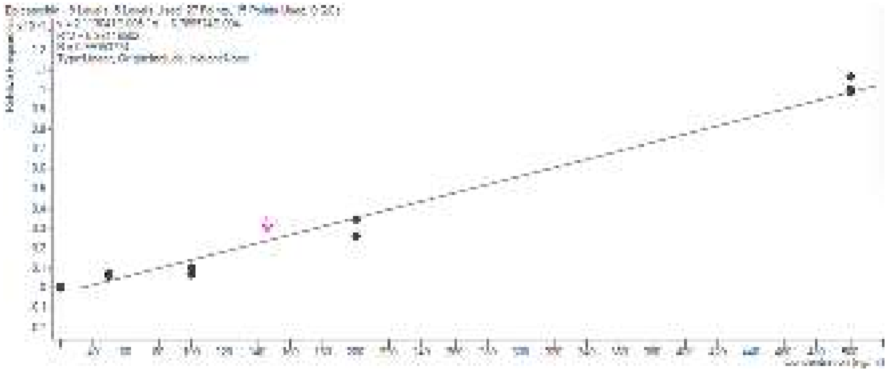

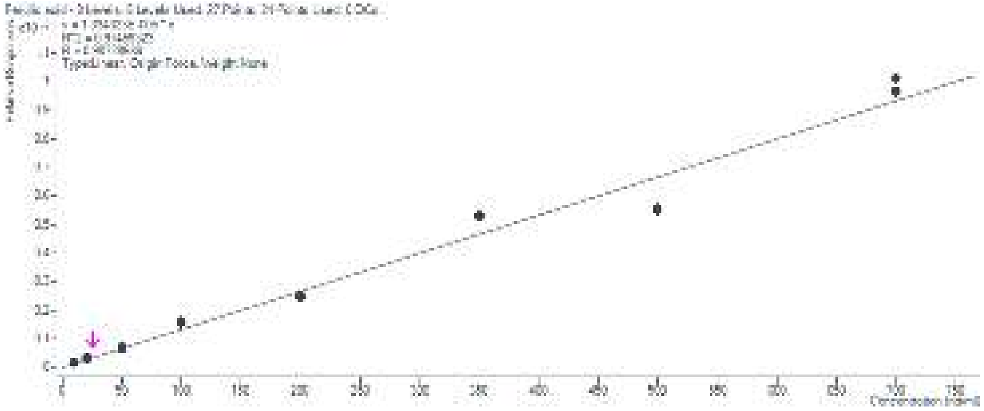

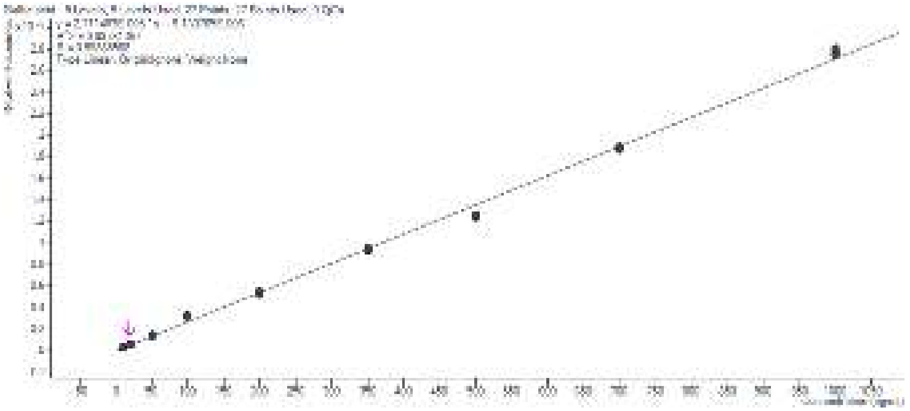

Limit of detection for all the analytes

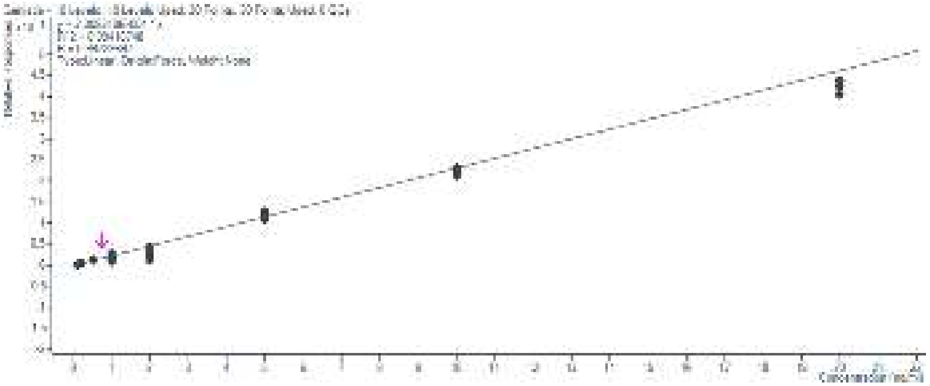

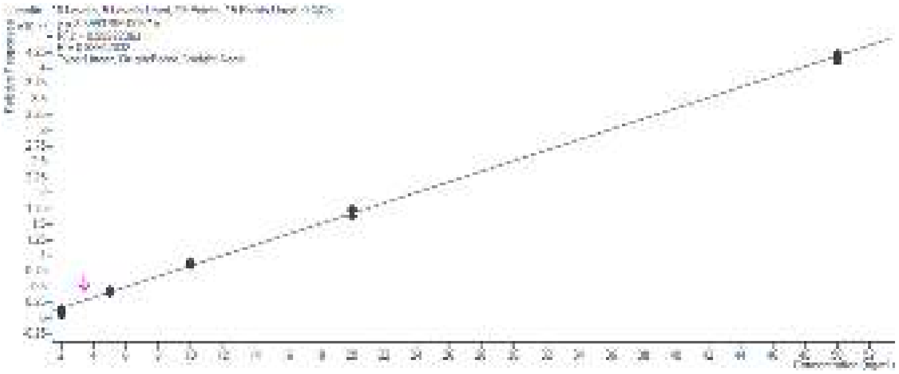

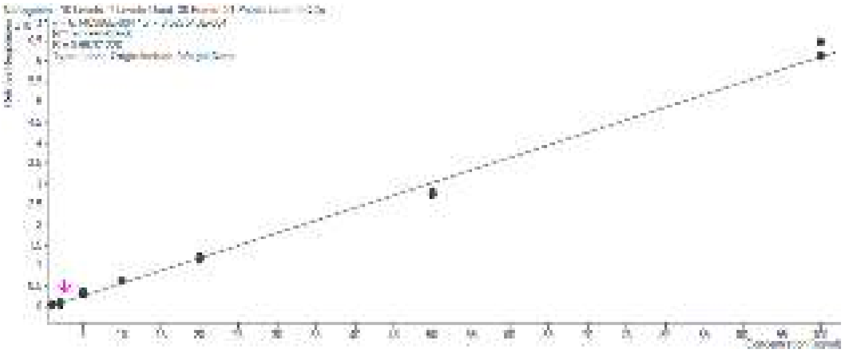

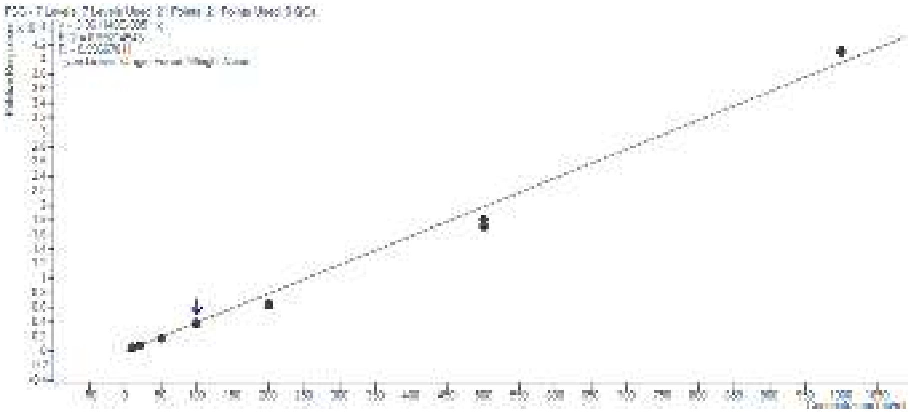

Limit of detection for all the analytes

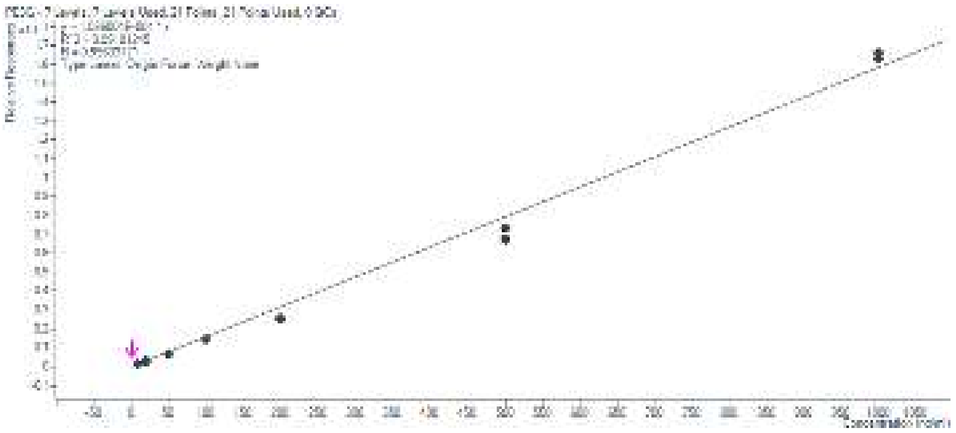

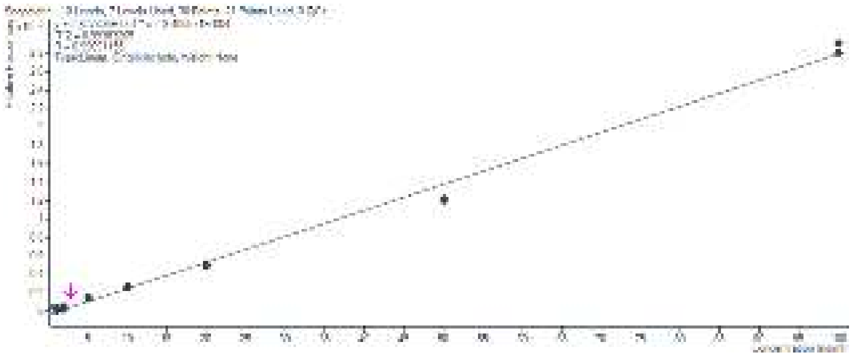

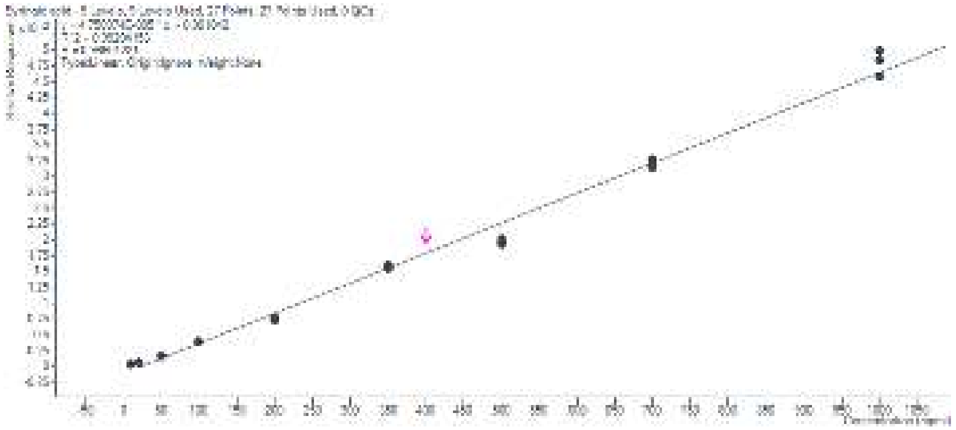

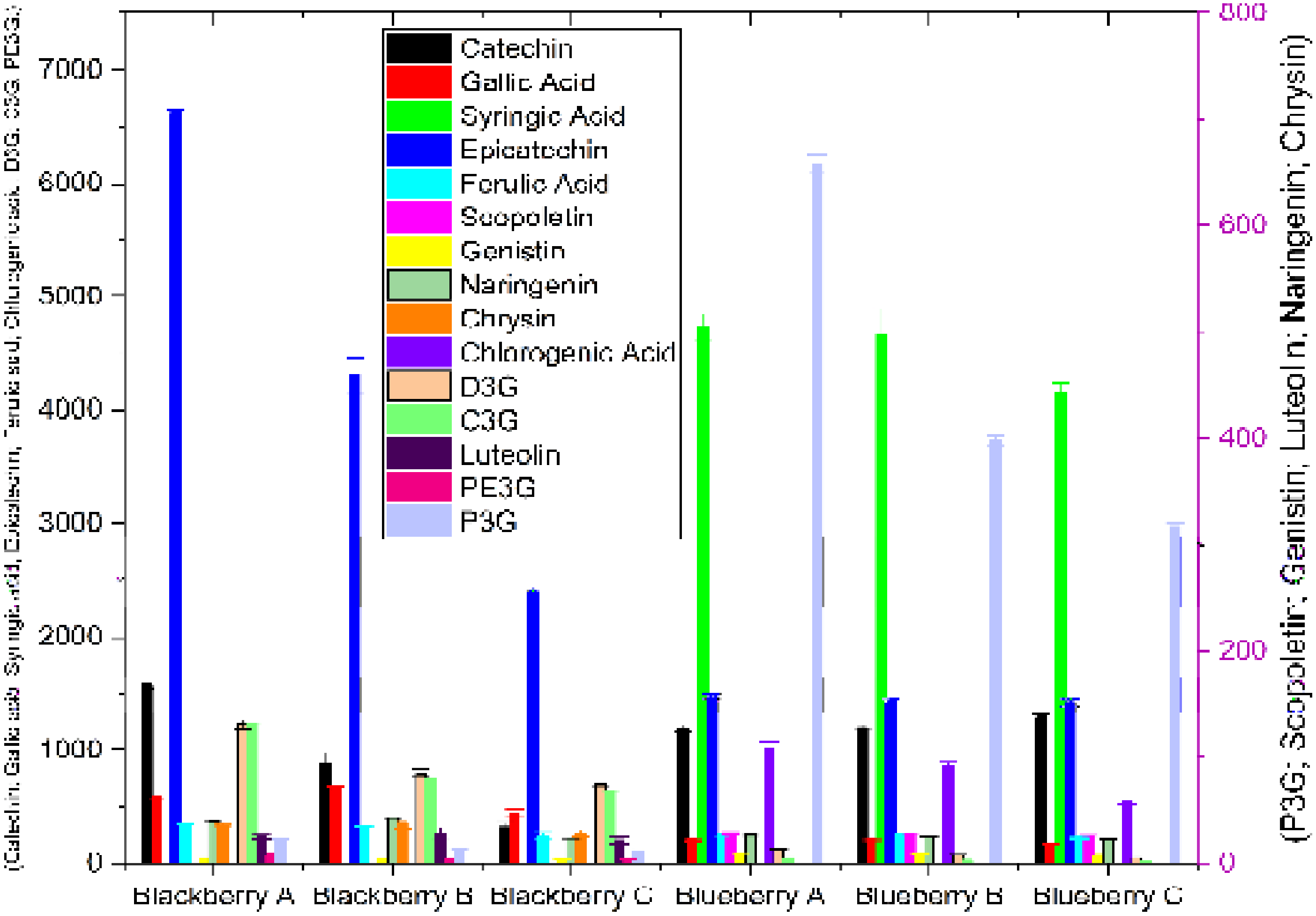

Catechin
Gallic Acid
Syringic Acid
Epicatechin
Ferulic Acid
Scopoletin
Genistin
Naringenin
Chrysin
Chlorogenic Acid
D3G
C3G
Luteolin
PE3G
P3G
0
1000
2000
3000
4000
5000
6000
7000
0
200
400
600
800
Blackberry A
Blackberry B
Blackberry C
Blueberry A
Blueberry B
Blueberry C
(P3G; Scopoletin; Genistin; Luteolin; Naringenin; Chrysin)

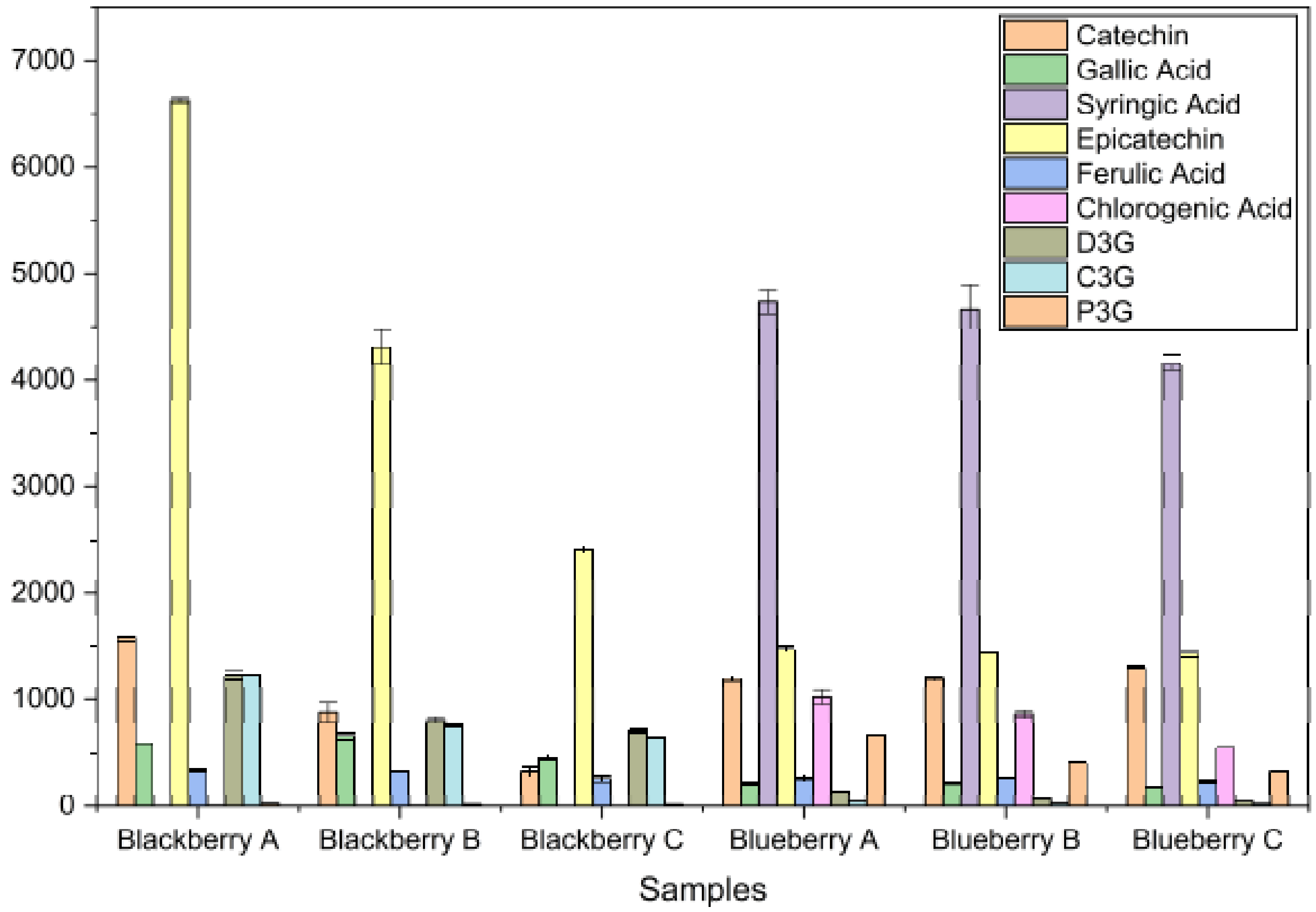

7000
6000
5000
4000
3000
2000
1000
0
Catechin
Gallic Acid
Syringic Acid
Epicatechin
Ferulic Acid
Chlorogenic Acid
D3G
C3G
P3G
Blackberry A
Blackberry B
Blackberry C
Blueberry A
Blueberry B
Blueberry C
Samples

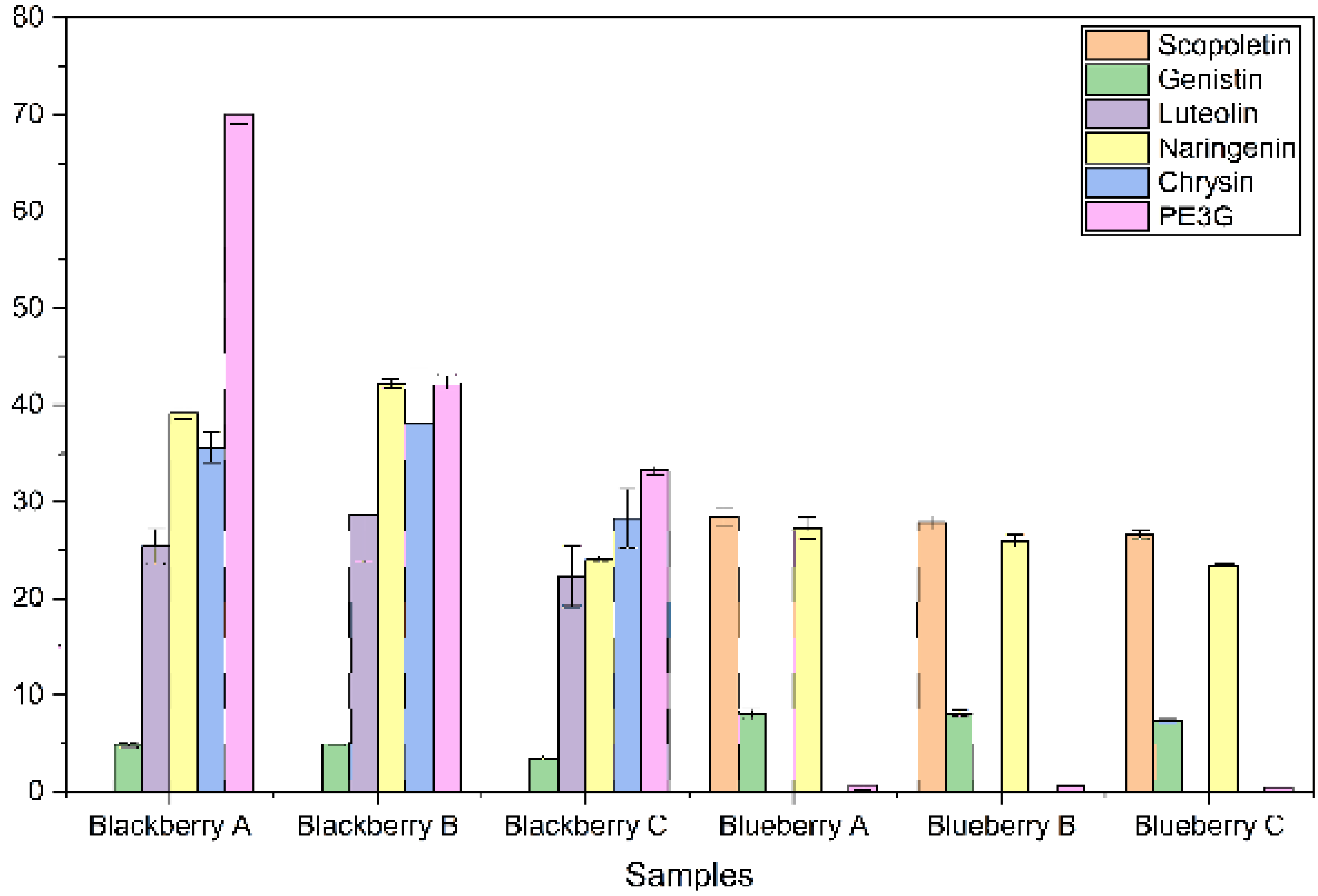

0
10
20
30
40
50
60
70
80
Scopoletin
Genistin
Luteolin
Naringenin
Chrysin
PE3G
Blackberry A
Blackberry B
Blackberry C
Blueberry A
Blueberry B
Blueberry C
Samples

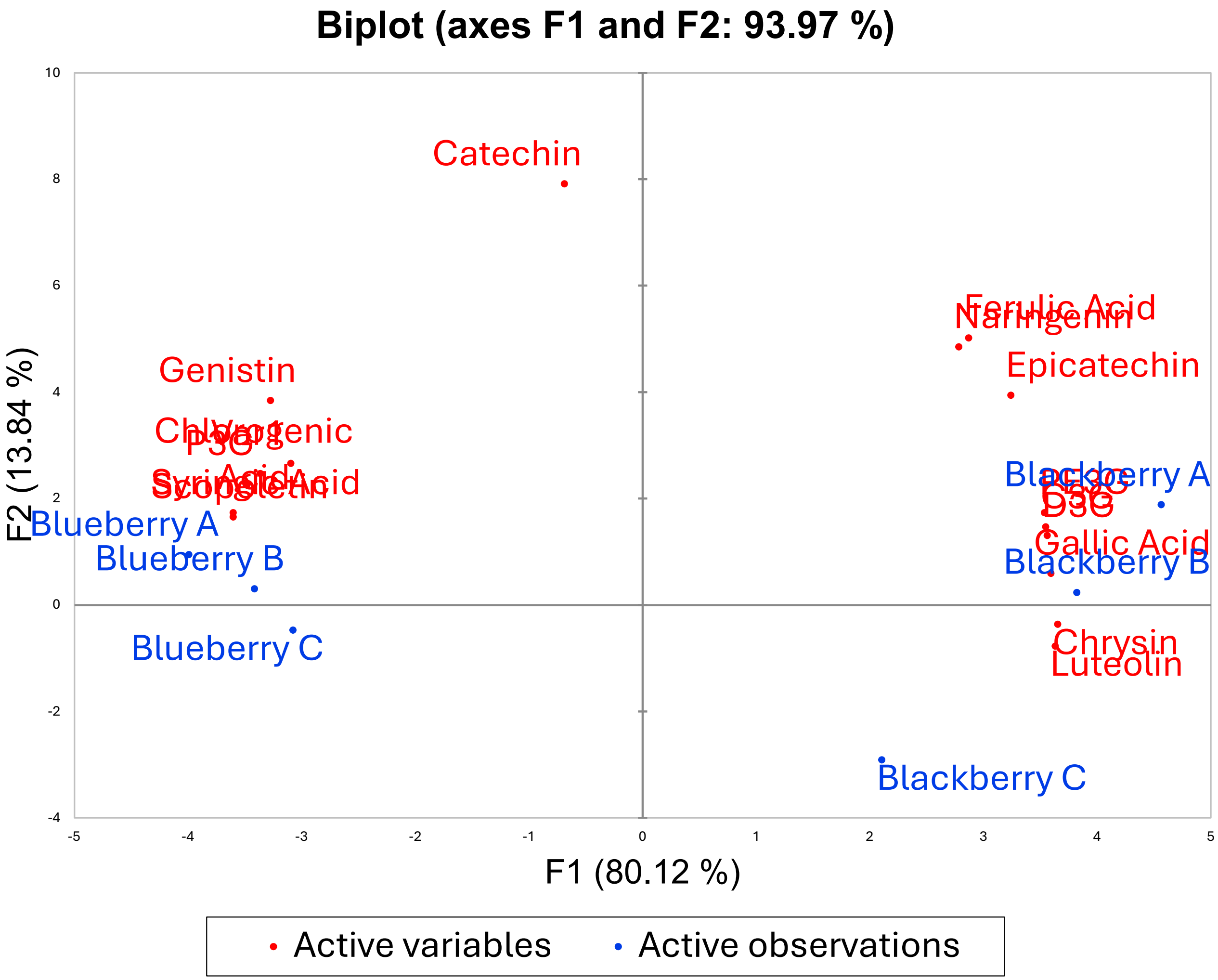
Biplot (axes F1 and F2: 93.97 %)
F1 (80.12 %)
F2 (13.84 %)
Catechin
Genistin
Ferulic Acid
Naringenin
Epicatechin
Blackberry A
D3G
Gallic Acid
Blackberry B
Chrysin
Luteolin
Blackberry C
Blueberry A
Blueberry B
Blueberry C
Active variables
Active observations

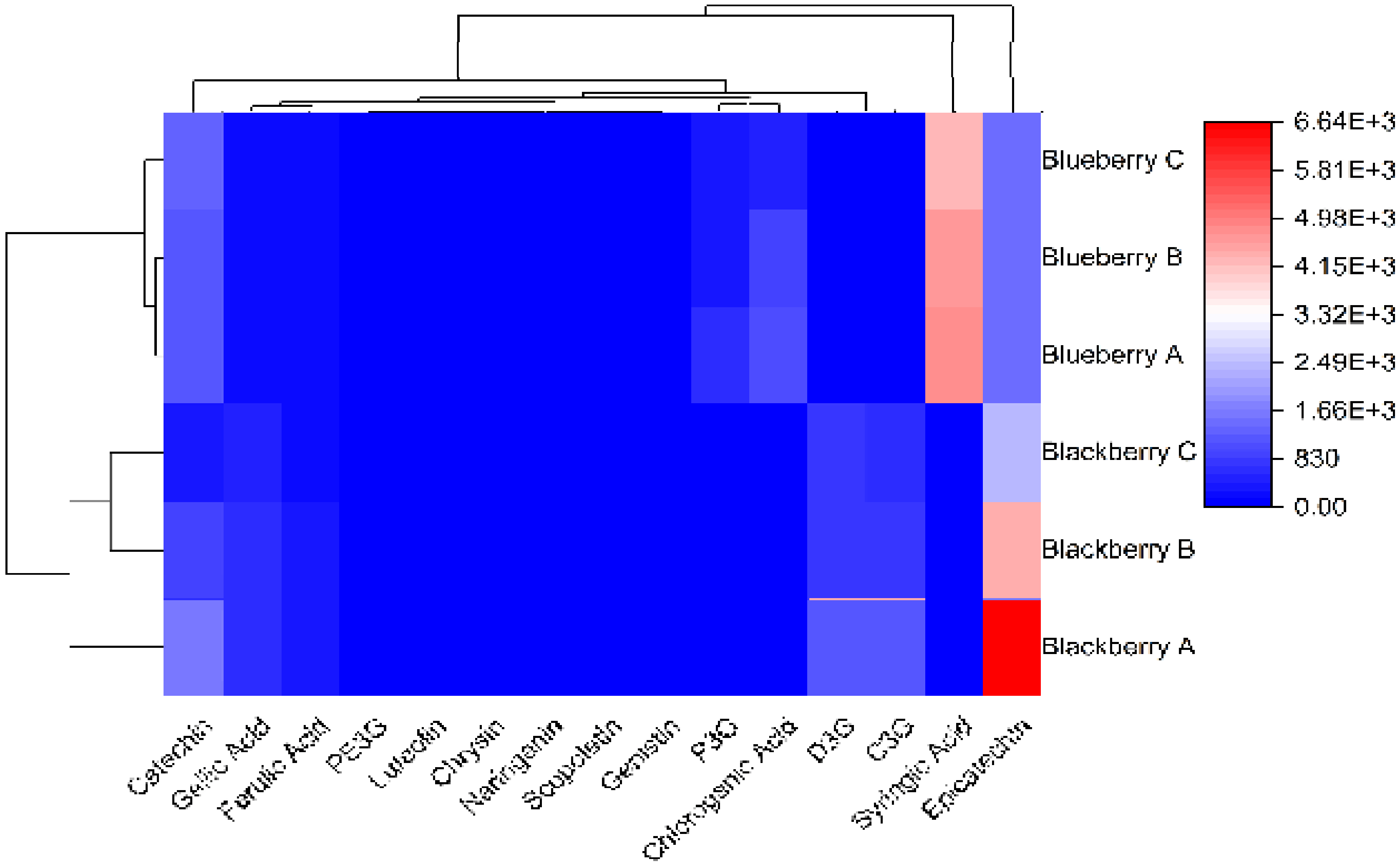
Blueberry C
Blueberry B
Blueberry A
Blackberry C
Blackberry B
Blackberry A
6.64E+3
5.81E+3
4.98E+3
4.15E+3
3.32E+3
2.49E+3
1.66E+3
830
0.00
Catechin
Gallic Acid
Ferulic Acid
PE3G
Luteolin
Chrysin
Naringenin
Scopoletin
Genistin
P3G
Chlorogenic Acid
D3G
C3G
Syringic Acid
Epicatechin

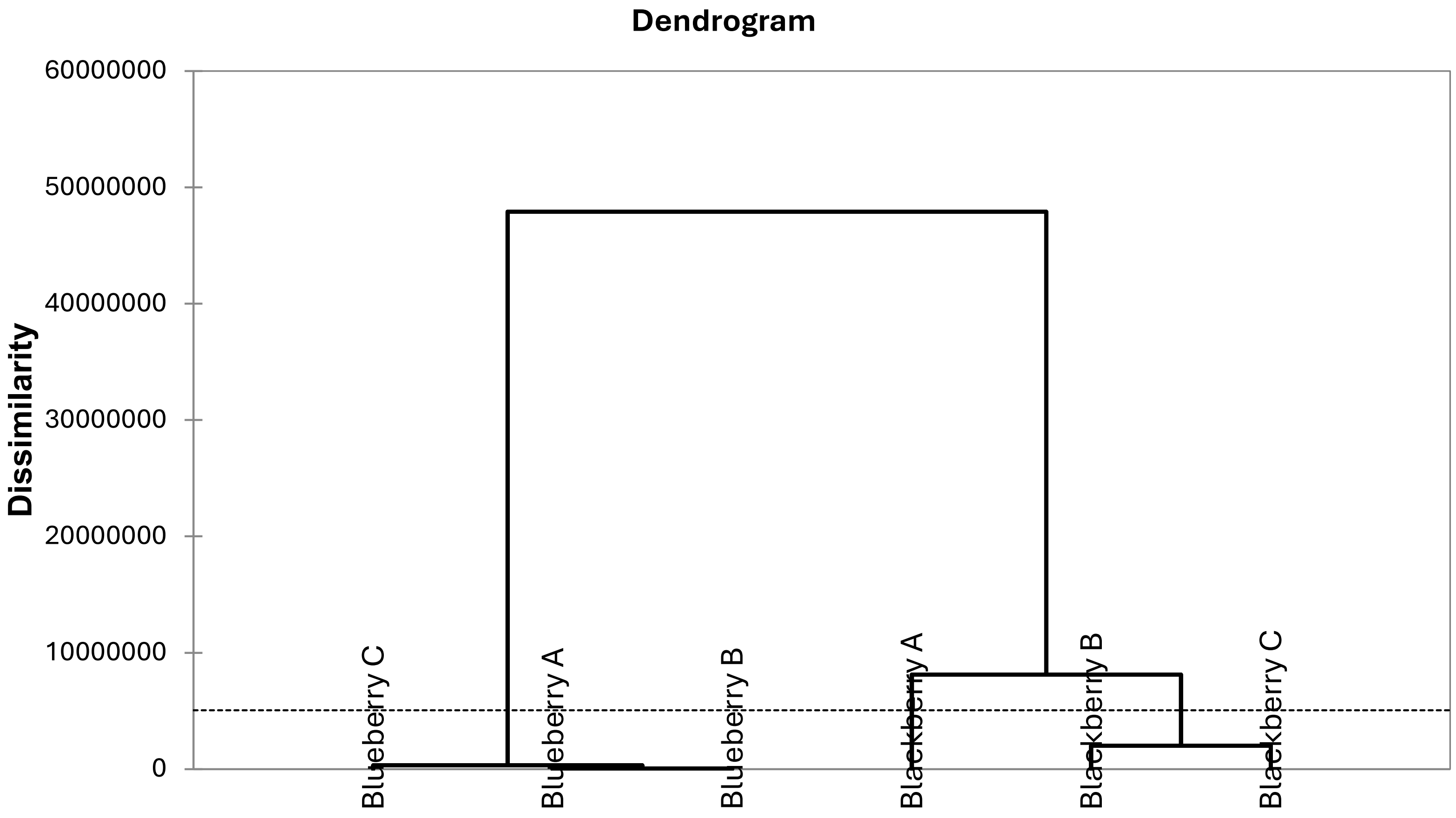

Dendrogram
Dissimilarity
60000000
50000000
40000000
30000000
20000000
10000000
0
Blueberry C
Blueberry A
Blueberry B
Blackberry A
Blackberry B
Blackberry C

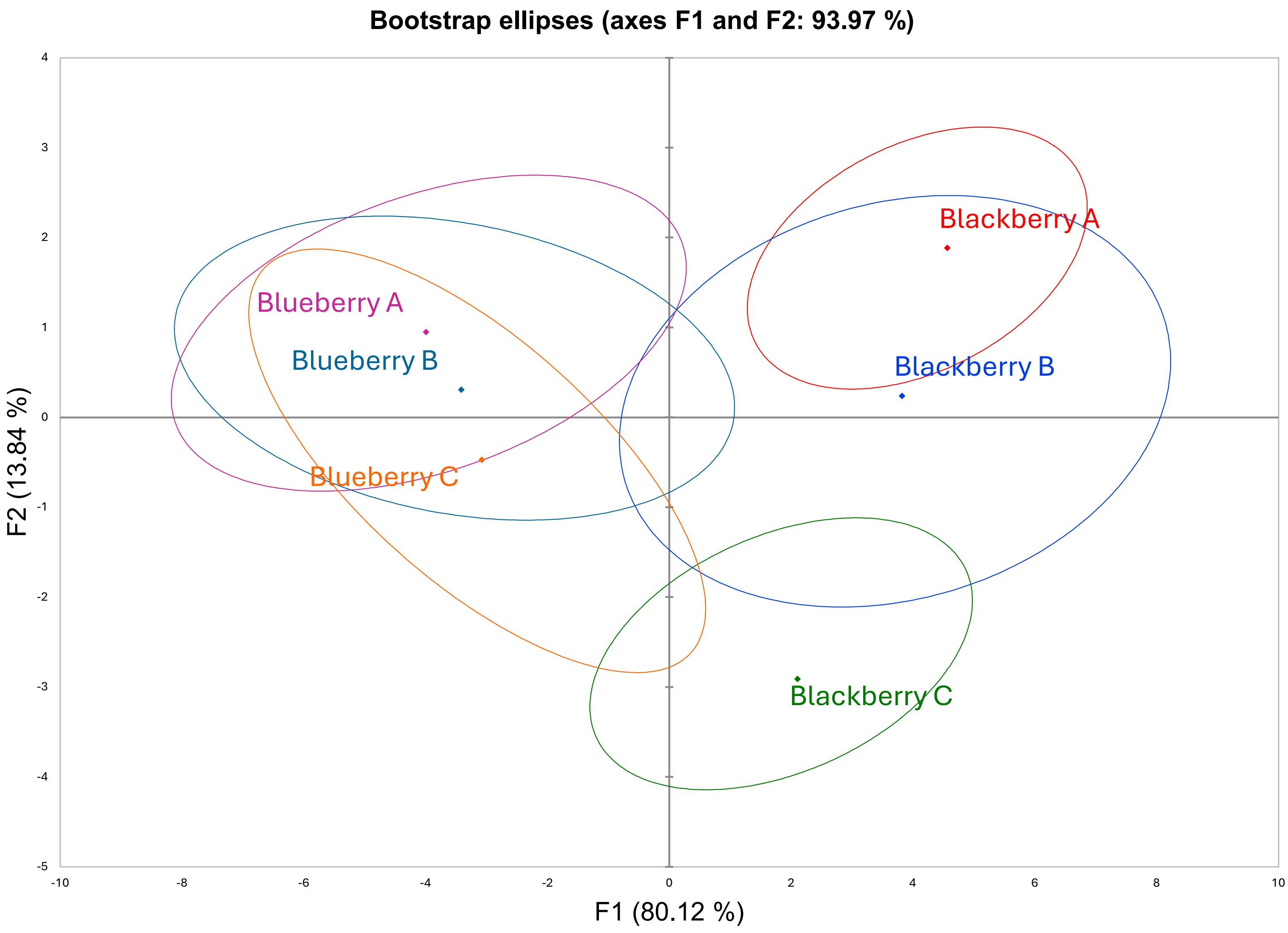
Bootstrap ellipses (axes F1 and F2: 93.97 %)
Blueberry A
Blueberry B
Blueberry C
Blackberry A
Blackberry B
Blackberry C
F1 (80.12 %)
F2 (13.84 %)
-10
-8
-6
-4
-2
0
2
4
6
8
10
4
3
2
1
0
-1
-2
-3
-4
-5
