# Supplementary material for: Berry-derived gold nanoparticles induce integrated ROS-mediated apoptosis, immune modulation, and transcriptomic remodeling in 4T1 triple-negative cancer cells
Source: Cell Death Discov. 2026 Apr 10;12:225. doi: 10.1038/s41420-026-03023-z (PMC13184259; doi:10.1038/s41420-026-03023-z)
Supplement: Supplementary file 3 — Figure S3 [file 41420_2026_3023_MOESM3_ESM.pdf]

Assay Class:	Eukaryote Total RNA Nano	Created:	8/21/2025 7:07:26 AM
Data Path:	C:\...Eukaryote Total RNA Nano_DE72903137_2025-08-21_07-07-19.xad	Modified:	8/21/2025 7:17:33 AM

Electrophoresis File Run Summary

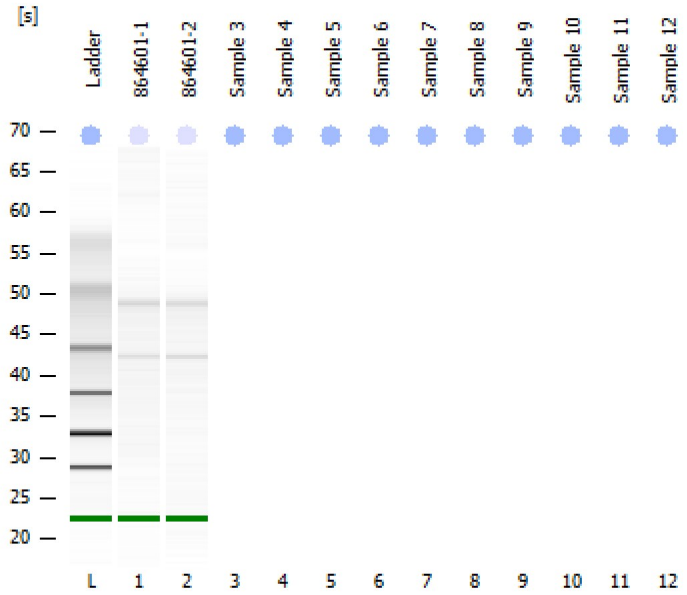

Instrument Information:

Instrument Name:	DE72903137	Firmware:	C.01.069
Serial#:	DE72903137	Type:	G2939A

Assay Information:

Assay Origin Path:	C:\Program Files (x86)\Agilent\2100 bioanalyzer\2100 expert\SecuredArea\Method\Templates\RNA\Eukaryote Total RNA Nano Series II. approved.xsy
Assay Class:	Eukaryote Total RNA Nano
Version:	2.6
Assay Comments:	Total RNA Analysis ng sensitivity (Eukaryote)

© Copyright 2003 - 2009 Agilent Technologies, Inc.

Chip Information:

Chip Lot #:

Reagent Kit Lot #:

Chip Comments:

864601-1

RIN: 6.10

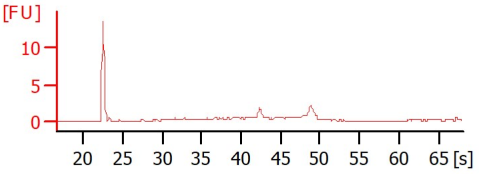

864601-2

RIN: 7.30

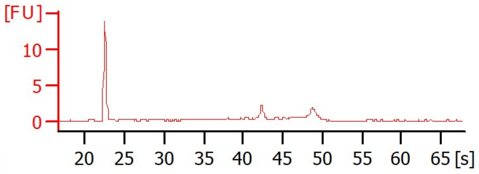

© Copyright 2003 - 2021 Agilent, Inc.

Assay Class: Eukaryote Total RNA Nano
Data Path: C:\...Eukaryote Total RNA Nano_DE72903137_2025-08-21_07-07-19.xad
Created: 8/21/2025 7:07:26 AM
Modified: 8/21/2025 7:17:33 AM

Electrophoresis File Run Summary (Chip Summary)

Sample Name	Sample Comment	Status	Result Label	Result Color	Approved
864601-1		✓	RIN: 6.10		Not Reviewed
864601-2		✓	RIN: 7.30		Not Reviewed
Sample 3					Not Reviewed
Sample 4					Not Reviewed
Sample 5					Not Reviewed
Sample 6					Not Reviewed
Sample 7					Not Reviewed
Sample 8					Not Reviewed
Sample 9					Not Reviewed
Sample 10					Not Reviewed
Sample 11					Not Reviewed
Sample 12					Not Reviewed
Ladder		✓	All Other Samples		Not Reviewed

Chip Lot # **Reagent Kit Lot #**

Chip Comments :

© Copyright 2003 - 2021 Agilent, Inc.

Assay Class: Eukaryote Total RNA Nano
Data Path: C:\...Eukaryote Total RNA Nano_DE72903137_2025-08-21_07-07-19.xad
Created: 8/21/2025 7:07:26 AM
Modified: 8/21/2025 7:17:33 AM

Electrophoresis Assay Details

General Analysis Settings

Number of Available Sample and Ladder Wells (Max.) : 13
Minimum Visible Range [s] : 17
Maximum Visible Range [s] : 70
Start Analysis Time Range [s] : 19
End Analysis Time Range [s] : 69
Ladder Concentration [ng/µl] : 150
Lower Marker Concentration [ng/µl] : 0
Upper Marker Concentration [ng/µl] : 0
Used Lower Marker for Quantitation
Standard Curve Fit is Logarithmic
Show Data Aligned to Lower Marker

Integrator Settings

Integration Start Time [s] : 19
Integration End Time [s] : 69
Slope Threshold : 0.6
Height Threshold [FU] : 0.5
Area Threshold : 0.2
Width Threshold [s] : 0.5
Baseline Plateau [s] : 6

Filter Settings

Filter Width [s] : 0.5
Polynomial Order : 4

Ladder

Ladder Peak	Size
1	25
2	200
3	500
4	1000
5	2000
6	4000

© Copyright 2003 - 2021 Agilent, Inc.

Assay Class:	Eukaryote Total RNA Nano	Created:	8/21/2025 7:07:26 AM
Data Path:	C:\...Eukaryote Total RNA Nano_DE72903137_2025-08-21_07-07-19.xad	Modified:	8/21/2025 7:17:33 AM

Electropherogram Summary

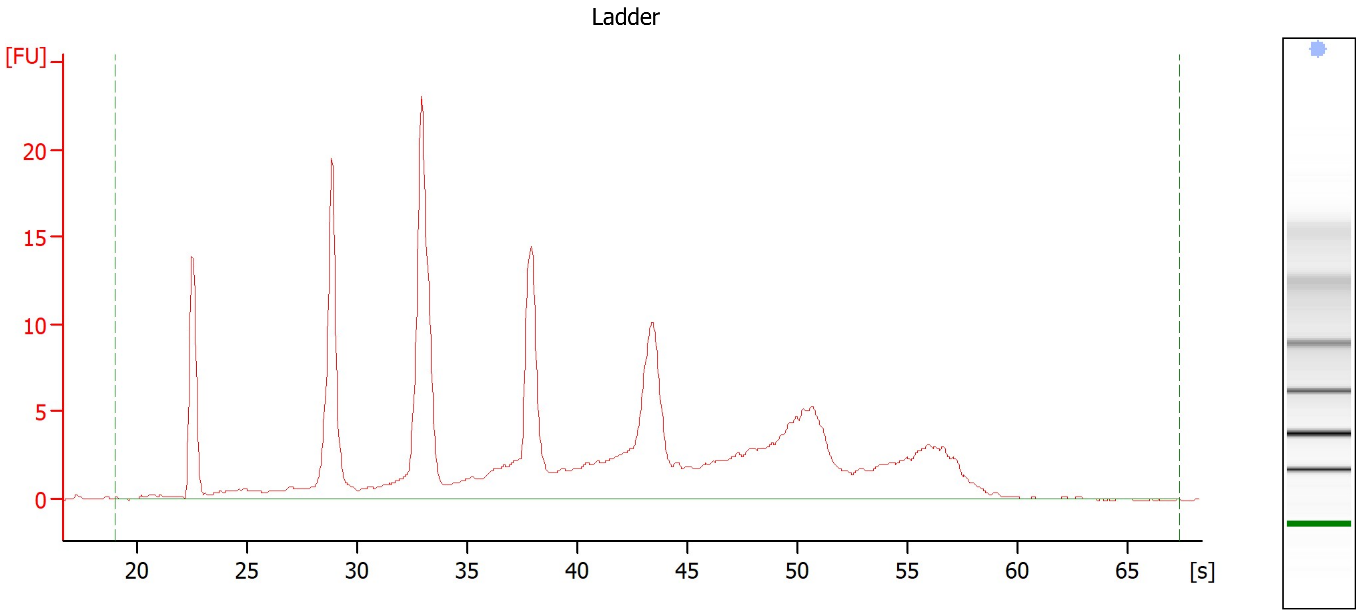

Overall Results for Ladder

RNA Area:	242.2	Result Flagging Color:	
RNA Concentration:	150 ng/µl	Result Flagging Label:	All Other Samples

 © Copyright 2003 - 2021 Agilent, Inc.

Assay Class:	Eukaryote Total RNA Nano	Created:	8/21/2025 7:07:26 AM
Data Path:	C:\...Eukaryote Total RNA Nano_DE72903137_2025-08-21_07-07-19.xad	Modified:	8/21/2025 7:17:33 AM

Electropherogram Summary Continued ...

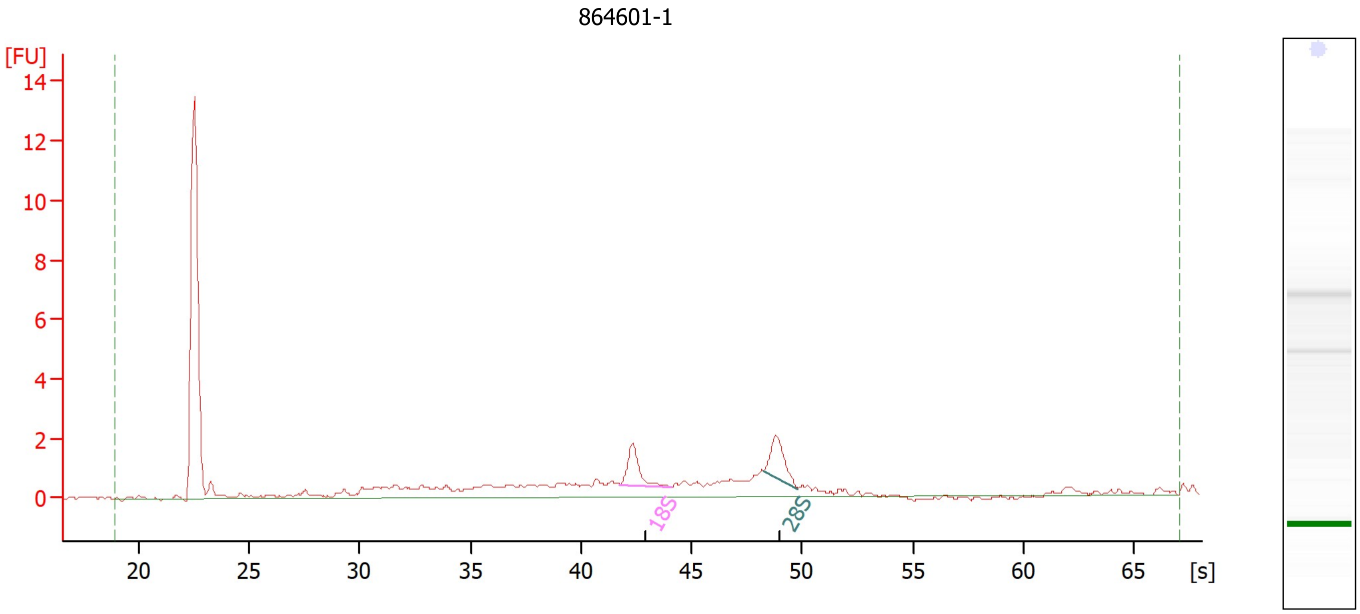

Overall Results for sample 1 : 864601-1

RNA Area:	29.5	RNA Integrity Number (RIN):	6.1 (B.02.11)
RNA Concentration:	18 ng/µl	Result Flagging Color:	
rRNA Ratio [28s / 18s]:	1.0	Result Flagging Label:	RIN: 6.10

Fragment table for sample 1 : 864601-1

Name	Start Time [s]	End Time [s]	Area	% of total Area
18S	41.74	44.13	1.9	6.4
28S	48.26	49.81	2.0	6.7

 © Copyright 2003 - 2021 Agilent, Inc.

Assay Class: Eukaryote Total RNA Nano
Data Path: C:\...Eukaryote Total RNA Nano_DE72903137_2025-08-21_07-07-19.xad

Created: 8/21/2025 7:07:26 AM
Modified: 8/21/2025 7:17:33 AM

Electropherogram Summary Continued ...

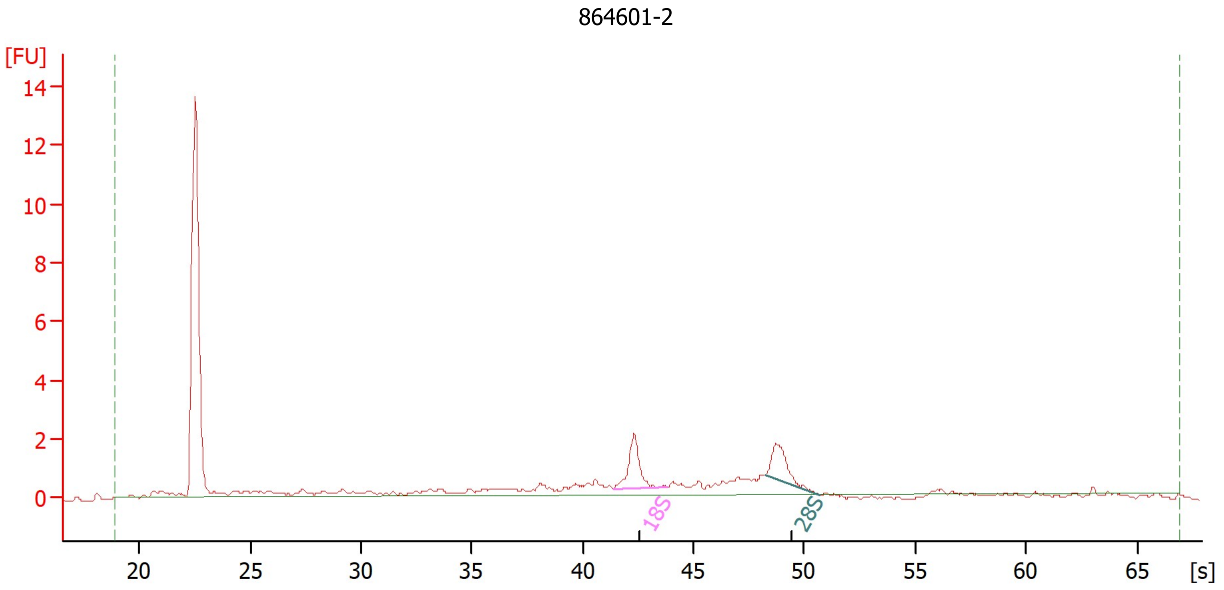

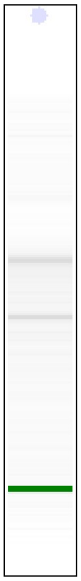

Overall Results for sample 2 : 864601-2

RNA Area:	23.8	RNA Integrity Number (RIN):	7.3 (B.02.11)
RNA Concentration:	15 ng/µl	Result Flagging Color:	
rRNA Ratio [28s / 18s]:	0.8	Result Flagging Label:	RIN: 7.30

Fragment table for sample 2 : 864601-2

Name	Start Time [s]	End Time [s]	Area	% of total Area
18S	41.36	43.84	2.6	10.8
28S	48.25	50.67	1.9	8.1

© Copyright 2003 - 2021 Agilent, Inc.

Assay Class:	Eukaryote Total RNA Nano	Created:	8/21/2025 7:07:26 AM
Data Path:	C:\...Eukaryote Total RNA Nano_DE72903137_2025-08-21_07-07-19.xad	Modified:	8/21/2025 7:17:33 AM

Gel Image

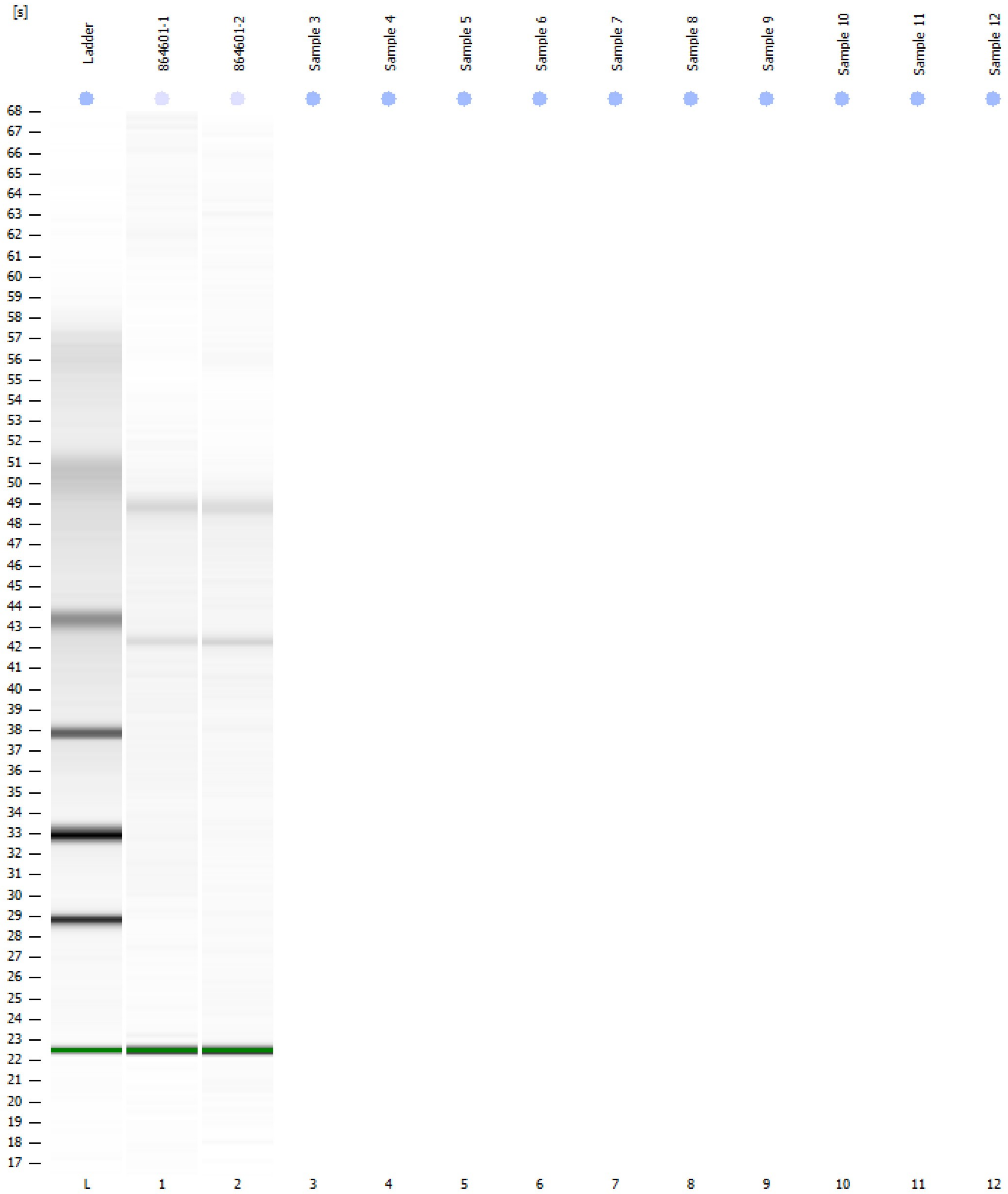

 © Copyright 2003 - 2021 Agilent, Inc.

Assay Class:	Eukaryote Total RNA Nano	Created:	8/21/2025 7:07:26 AM
Data Path:	C:\...Eukaryote Total RNA Nano_DE72903137_2025-08-21_07-07-19.xad	Modified:	8/21/2025 7:17:33 AM

Curves

Standard Curve

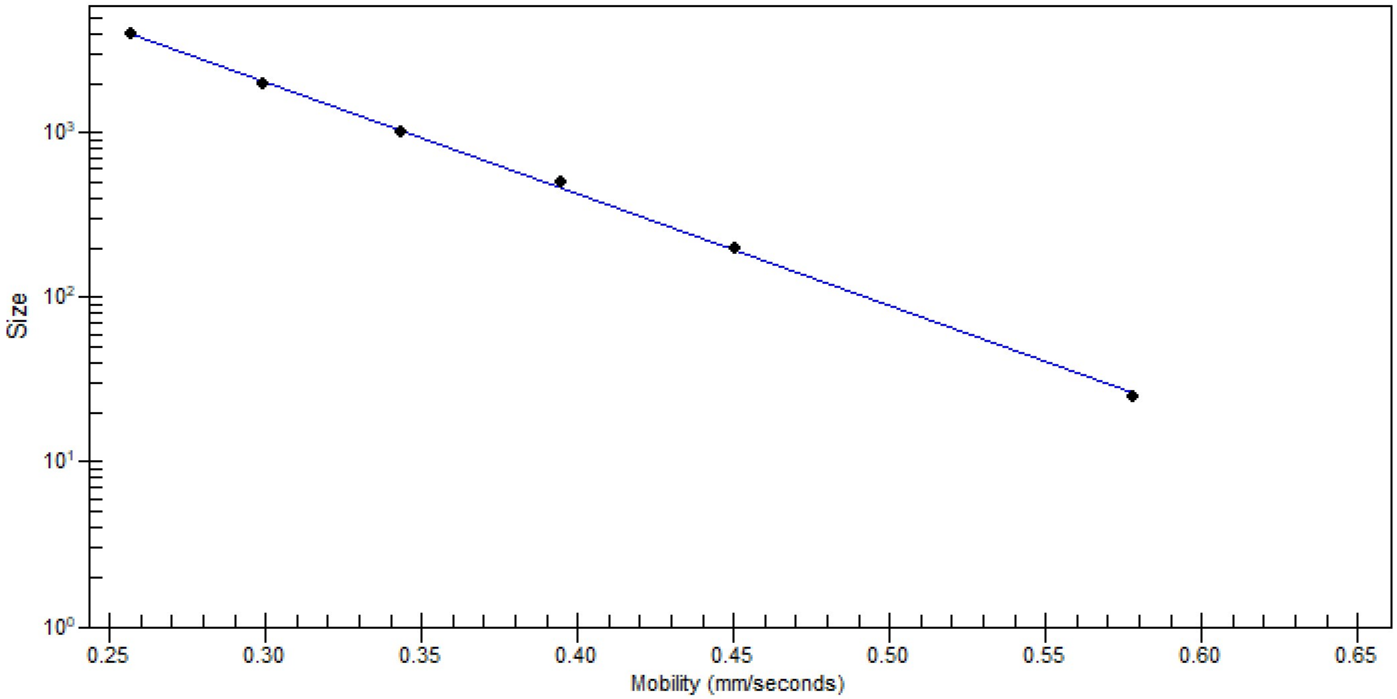

© Copyright 2003 - 2021 Agilent, Inc.

Assay Class: Eukaryote Total RNA Nano
Data Path: C:\...Eukaryote Total RNA Nano_DE72903137_2025-08-21_07-07-19.xad
Created: 8/21/2025 7:07:26 AM
Modified: 8/21/2025 7:17:33 AM

Run Logbook

Description	Number	Source	Category	Sub Category	Time	Time Zone	User	Host
Run ended on port 1 (Number of wells acquired: 3)		Instrument	Run		8/21/2025 7:17:33 AM	(GMT --05:00) Central Standard Time	Jonathan Dixit	BIOANALYZER2
Run started on port 1 (File: C:\Program Files (x86)\Agilent\2100 bioanalyzer\2100 expert\Secured Area\Data\RNA\EukaryoteTotal RNA Nano\Eukaryote Total RNA Nano Series II_approved\2100 expert_Eukaryote Total RNA Nano_DE72903137_2025-08-21_07-07-19.xad)		Instrument	Run		8/21/2025 7:07:32 AM	(GMT --05:00) Central Standard Time	Jonathan Dixit	BIOANALYZER2
Product Number : G2939A		Instrument	Run		8/21/2025 7:07:32 AM	(GMT --05:00) Central Standard Time	Jonathan Dixit	BIOANALYZER2
Name :		Instrument	Run		8/21/2025 7:07:32 AM	(GMT --05:00) Central Standard Time	Jonathan Dixit	BIOANALYZER2
Vendor : Agilent Technologies		Instrument	Run		8/21/2025 7:07:32 AM	(GMT --05:00) Central Standard Time	Jonathan Dixit	BIOANALYZER2
Serial# : DE72903137		Instrument	Run		8/21/2025 7:07:32 AM	(GMT --05:00) Central Standard Time	Jonathan Dixit	BIOANALYZER2
Firmware : C.01.069		Instrument	Run		8/21/2025 7:07:32 AM	(GMT --05:00) Central Standard Time	Jonathan Dixit	BIOANALYZER2
Cartridge : Electrode		Instrument	Run		8/21/2025 7:07:32 AM	(GMT --05:00) Central Standard Time	Jonathan Dixit	BIOANALYZER2

 © Copyright 2003 - 2021 Agilent, Inc.

Assay Class: Eukaryote Total RNA Nano
Data Path: C:\...Eukaryote Total RNA Nano_DE72903137_2025-08-21_07-07-19.xad

Created: 8/21/2025 7:07:26 AM
Modified: 8/21/2025 7:17:33 AM

Signature Logbook

Time	Time Zone	User Name	Role	Meaning	Comment	Version
8/21/2025 7:07:26 AM	(GMT --05:00) Central Standard Time	Jonathan Dixit	Advanced Operator	Started Chip Run		1

© Copyright 2003 - 2021 Agilent, Inc.

Assay Class: Eukaryote Total RNA Nano
Data Path: C:\...Eukaryote Total RNA Nano_DE72903137_2025-08-21_07-07-19.xad

Created: 8/21/2025 7:07:26 AM
Modified: 8/21/2025 7:17:33 AM

Audit Trail

Desc.	User Name	Version	Meaning	Sample Name	Sample Number
Started Chip Run	Jonathan Dixit	1	Started Chip Run		

© Copyright 2003 - 2021 Agilent, Inc.

OSUCCC Genomics Shared Resource: Next Generation Sequencing Report

PI/User: Fagbohun, Oladapo
eRamp: 740023
Report date: 08/11/2025

The mRNA libraries were generated using the flowing reagents from Qiagen:

- QIAseq FastSelect RNA Lib HMR Kit(96) -cat. no. 334235 Using both primers N6-T RT and ODT-RT.
- QIA UX96 Index kit UDI-B (96) 331825
- Library input amount: 100ng total RNA (quantified using Qubit Fluorometer).
- Fragmentation: 3 minutes
- PCR: 20x
- Libraries were sequenced with Novaseq X Plus 10B.
- If this study results in a publication, please acknowledge the GSR and its partially supporting grant P30-CA016058 from National Cancer Institute. Please do not hesitate to contact us for more details you may need in the future.

WorkID	ID on cap	Sample name	LibQubit ng/ul	Size bp	LibNo	i7	Seq_name	PF Clusers
1	C	4T1 CELLS CONTROL	15.9	501	13430	iMUDI162	T13430_FagbohunO_C_V1Q_1	45456083
2	BLU	4T1 CELLS WITH BLUEBERRY	12	487	13431	iMUDI163	T13431_FagbohunO_BLU_V1Q_1	44851127
3	BLA	4T1 CELLS WITH BLACKBERRY	5.87	479	13432	iMUDI164	T13432_FagbohunO_BLA_V1Q_1	37614465
4	BLU-A	4T1 CELLS WITH BLUEBERRY N	12.7	488	13433	iMUDI165	T13433_FagbohunO_BLU-A_V1Q_1	50691706
6	CFO		1.46	516	13434	iMUDI166	T13434_FagbohunO_CFO_V1Q_1	41204555
8	FGD		11.2	488	13435	iMUDI167	T13435_FagbohunO_FGD_V1Q_1	45265645
10	RAW RNA	RAW CELLS	13.4	496	13436	iMUDI168	T13436_FagbohunO_RAW RNA_V1Q_1	39875755
11	RU2C	RAW CELLS WITH LPS	13.3	471	13437	iMUDI169	T13437_FagbohunO_RU2C_V1Q_1	37532186

Libraries traces

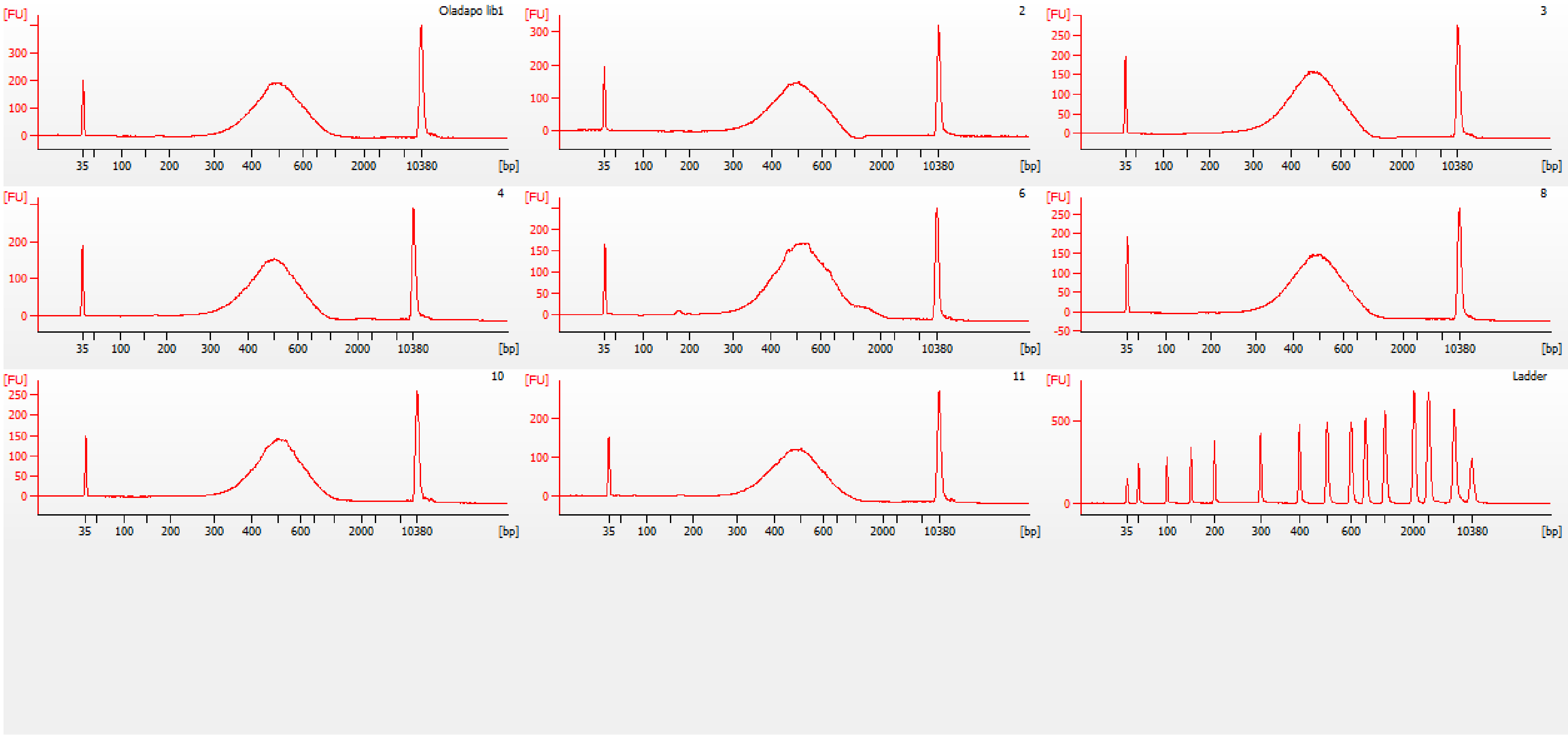
Oladapo lib1
2
3
4
6
8
10
11
Ladder
[FU]
[bp]
35 100 200 300 400 600 2000 10380

Gene-level Quantification

Name: Counts

Input 1: Annotation File

40,402 genomic features of type 'gene' and grouped by the 'ID' attribute have been retrieved from 42 reference sequences present in the annotation file 'genomic.gff'.

Reference sequence	Genomic features	Length (bp)
NC_000069.7	1,977	159,644,302
NC_000068.8	3,094	181,642,880
NC_000067.7	2,366	194,879,448
NC_000087.8	300	90,855,176
NT_187058.1	2	163,041
NC_000086.8	1,544	169,111,787
NT_187057.1	5	200,398
NT_187056.1	2	173,826
NC_000085.7	1,153	61,221,141
NT_187055.1	1	1,475
NT_166462.1	1	28,015
NT_166443.1	1	21,592
NT_165789.3	5	469,175
NT_166456.1	1	14,096
NT_166438.1	4	189,992
NC_000084.7	1,079	90,613,788
NC_000083.7	1,726	95,187,353
NC_000082.7	1,278	97,885,218
NC_000081.7	1,516	103,929,578
NC_000080.7	1,817	124,915,098
NT_166282.1	7	153,219
NT_187064.1	1	20,242
NT_166281.1	8	229,381
NC_000078.7	1,666	119,984,981
NT_166280.1	8	167,692
NC_000079.7	1,836	120,779,797
NC_000076.7	1,914	130,428,771
NT_166307.1	2	172,761
NC_000077.7	2,697	121,847,592
NC_000074.7	2,054	130,013,050
NC_000075.7	2,120	124,204,755
NT_166450.1	1	47,073
NT_162750.1	10	175,458
NT_187060.1	1	60,820
NC_005089.1	37	15,422
NW_023337853.1	1	27,820
NC_000072.7	2,274	149,487,661
NC_000073.7	3,226	144,877,796
NW_023337852.1	1	7,732
NC_000070.7	2,361	156,691,794
NT_187059.1	13	924,675
NC_000071.7	2,292	151,623,773
**Total**	**40,402**	**2,723,089,644**

Input 2: Alignment Files

A total of 5 alignment files have been processed.

Sample	Filename	Program	Sequencing	Format
4T1-BLA-1_S1_L001_R_merged	D:\4T1 Analysis 2\4T1-BLA-1_S1_L001_R_merged.bam	STAR (version='2.7.8a')	single-end	bam
4T1BLAA-1_S1_L001_R_merged	D:\4T1 Analysis 2\4T1BLAA-1_S1_L001_R_merged.bam	STAR (version='2.7.8a')	single-end	bam
4T1BLU-1_S1_L001_R_merged	D:\4T1 Analysis 2\4T1BLU-1_S1_L001_R_merged.bam	STAR (version='2.7.8a')	single-end	bam
4T1BLUA-1_S1_L001_R_merged	D:\4T1 Analysis 2\4T1BLUA-1_S1_L001_R_merged.bam	STAR (version='2.7.8a')	single-end	bam
4T1C-1_S1_L001_R_merged	D:\4T1 Analysis 2\4T1C-1_S1_L001_R_merged.bam	STAR (version='2.7.8a')	single-end	bam

Results Overview

Input Reads		Aligned Reads					
**Sample**	**Total Records**	**Feature**	**No Feature**	**Ambiguous**	**Alignment not Unique**	**Low Alignment Quality**	**Not align[illegible]**
4T1-BLA-1_S1_L001_R_merged	81,359,434	29,821,435 / 36.65%	31,851,621 / 39.15%	380,341 / 0.47%	19,306,037 / 23.73%	0	[illegible]
4T1BLAA-1_S1_L001_R_merged	138,809,960	48,953,728 / 35.27%	51,379,972 / 37.01%	624,035 / 0.45%	37,852,225 / 27.27%	0	[illegible]
4T1BLU-1_S1_L001_R_merged	98,925,266	35,416,426 / 35.80%	38,554,758 / 38.97%	449,703 / 0.45%	24,504,379 / 24.77%	0	[illegible]
4T1BLUA-1_S1_L001_R_merged	108,055,131	38,349,395 / 35.49%	40,984,445 / 37.93%	518,120 / 0.48%	28,203,171 / 26.10%	0	[illegible]
4T1C-1_S1_L001_R_merged	99,704,322	34,464,214 / 34.57%	37,244,921 / 37.36%	415,484 / 0.42%	27,579,703 / 27.66%	0	[illegible]

Warnings:

- 10,306 features (25.51%) for which no aligned reads were detected in any of the samples. Features with low counts provide little evidence for differential expression so they should be filtered out to improve further analysis.

Analysis Parameters

Parameter	Value
Feature File	genomic.gff
Quantification Level	gene
Group by	ID
Strand Specificity	Strand Specific Forward
Overlap Mode	Union
Lowest Mapping Quality	10

Read Alignment (STAR) Results

Input 1: Reference Genome Sequences

GCF_000001635.27_GRCm39_genomic

Sequences	Minimum Length	Maximum Length	Average Length	Total Length
61	1,976	195,154,279	44,724,958	2,728,222,451

Input 2: FASTQ Files

A total of 15 libraries have been processed.

Sample Name	Files	Sequencing	Format
BLA-1_S7_L001_R_Merged	BLA-1_S7_L001_R_Merged.fastq	Single-End	FASTQ
BLA-2_S8_L001_R_Merged	BLA-2_S8_L001_R_Merged.fastq	Single-End	FASTQ
BLA-3_S9_L001_R_Merged	BLA-3_S9_L001_R_Merged.fastq	Single-End	FASTQ
BLA-A-1_S13_L001_R_Merged	BLA-A-1_S13_L001_R_Merged.fastq	Single-End	FASTQ
BLA-A-2_S14_L001_R_Merged	BLA-A-2_S14_L001_R_Merged.fastq	Single-End	FASTQ
BLA-A-3_S15_L001_R1_001_Merged	BLA-A-3_S15_L001_R1_001_Merged.fastq	Single-End	FASTQ
BLU-1_S4_L001_R_Merged	BLU-1_S4_L001_R_Merged.fastq	Single-End	FASTQ
BLU-2_S5_L001_R_Merged	BLU-2_S5_L001_R_Merged.fastq	Single-End	FASTQ
BLU-3_S6_L001_R_Merged	BLU-3_S6_L001_R_Merged.fastq	Single-End	FASTQ
BLU-A-1S10_L001_R_Merged	BLU-A-1S10_L001_R_Merged.fastq	Single-End	FASTQ
BLU-A-2_S11_L001_R_Merged	BLU-A-2_S11_L001_R_Merged.fastq	Single-End	FASTQ
BLU-A-3_S12_L001_R_Merged	BLU-A-3_S12_L001_R_Merged.fastq	Single-End	FASTQ
CONTROL-1_S1_L001_R_Merged	CONTROL-1_S1_L001_R_Merged.fastq	Single-End	FASTQ
CONTROL-2_S2_L001_R_Merged	CONTROL-2_S2_L001_R_Merged.fastq	Single-End	FASTQ
CONTROL-3_S3_L001_R_Merged	CONTROL-3_S3_L001_R_Merged.fastq	Single-End	FASTQ

Results Overview

Unique Reads

Name	Total Reads	Uniquely Mapped Reads	Average Mapped Length	Number of Splices			
				Total	Annot.	GT/AG	GC/AG
BLA-1_S7_L001_R_Merged	68,000,000	56,091,192 / 82.487%	142.05	14,924,363	0	14,542,976	159,955
BLA-2_S8_L001_R_Merged	68,000,000	56,091,542 / 82.488%	142.05	14,924,074	0	14,542,807	159,764
BLA-3_S9_L001_R_Merged	68,000,000	56,090,027 / 82.485%	142.05	14,922,484	0	14,541,378	159,873
BLA-A-1_S13_L001_R_Merged	68,000,000	52,828,074 / 77.688%	143.86	16,349,021	0	16,063,669	178,402
BLA-A-2_S14_L001_R_Merged	68,000,000	52,824,174 / 77.683%	143.86	16,359,138	0	16,074,291	178,514
BLA-A-3_S15_L001_R1_001_Merged	68,000,000	52,825,649 / 77.685%	143.87	16,350,659	0	16,065,112	178,701
BLU-1_S4_L001_R_Merged	68,000,000	56,419,052 / 82.969%	142.2	15,667,724	0	15,309,810	131,780
BLU-2_S5_L001_R_Merged	68,000,000	56,415,686 / 82.964%	142.2	15,665,616	0	15,307,854	131,821
BLU-3_S6_L001_R_Merged	68,000,000	56,418,587 / 82.969%	142.2	15,667,546	0	15,309,647	131,779
BLU-A-1S10_L001_R_Merged	68,000,000	53,559,265 / 78.764%	142.12	16,650,316	0	16,167,752	152,458
BLU-A-2_S11_L001_R_Merged	68,000,000	53,560,561 / 78.766%	142.11	16,649,768	0	16,167,016	152,418
BLU-A-3_S12_L001_R_Merged	68,000,000	53,555,304 / 78.758%	142.11	16,646,162	0	16,162,544	152,576
CONTROL-1_S1_L001_R_Merged	68,000,000	53,949,544 / 79.338%	141.95	18,442,977	0	18,071,984	152,463
CONTROL-2_S2_L001_R_Merged	68,000,000	53,946,634 / 79.333%	141.95	18,446,833	0	18,075,494	152,446
CONTROL-3_S3_L001_R_Merged	68,000,000	53,944,676 / 79.33%	141.95	18,442,655	0	18,071,205	152,329

Name	Mismatch Rate per Base (%)	Deletions		Insertions	
		Rate per Base (%)	Average Length	Rate per Base (%)	Average Length
BLA-1_S7_L001_R_Merged	0.48	0.04	3.1	0.03	2.03
BLA-2_S8_L001_R_Merged	0.48	0.04	3.1	0.03	2.03
BLA-3_S9_L001_R_Merged	0.48	0.04	3.1	0.03	2.03
BLA-A-1_S13_L001_R_Merged	0.38	0.03	2.42	0.02	2.23
BLA-A-2_S14_L001_R_Merged	0.38	0.03	2.42	0.02	2.23
BLA-A-3_S15_L001_R1_001_Merged	0.38	0.03	2.42	0.02	2.22
BLU-1_S4_L001_R_Merged	0.44	0.04	2.89	0.02	1.95
BLU-2_S5_L001_R_Merged	0.44	0.04	2.89	0.02	1.95
BLU-3_S6_L001_R_Merged	0.44	0.04	2.88	0.02	1.95
BLU-A-1S10_L001_R_Merged	0.45	0.04	2.95	0.02	1.93
BLU-A-2_S11_L001_R_Merged	0.45	0.04	2.95	0.02	1.94
BLU-A-3_S12_L001_R_Merged	0.45	0.04	2.95	0.02	1.94
CONTROL-1_S1_L001_R_Merged	0.45	0.04	2.97	0.02	1.92
CONTROL-2_S2_L001_R_Merged	0.45	0.04	2.97	0.02	1.92
CONTROL-3_S3_L001_R_Merged	0.45	0.04	2.97	0.02	1.92

Multi-mapping Reads

Name	Reads Mapped to Multiple Loci	Reads Mapped to too Many Loci
BLA-1_S7_L001_R_Merged	5,100,856 / 7.501%	279,825 / 0.412%
BLA-2_S8_L001_R_Merged	5,100,367 / 7.501%	279,939 / 0.412%
BLA-3_S9_L001_R_Merged	5,100,671 / 7.501%	279,923 / 0.412%
BLA-A-1_S13_L001_R_Merged	6,550,609 / 9.633%	141,577 / 0.208%
BLA-A-2_S14_L001_R_Merged	6,548,856 / 9.631%	141,256 / 0.208%
BLA-A-3_S15_L001_R1_001_Merged	6,543,817 / 9.623%	141,301 / 0.208%
BLU-1_S4_L001_R_Merged	5,486,288 / 8.068%	287,170 / 0.422%
BLU-2_S5_L001_R_Merged	5,487,442 / 8.07%	286,935 / 0.422%
BLU-3_S6_L001_R_Merged	5,486,518 / 8.068%	286,864 / 0.422%
BLU-A-1S10_L001_R_Merged	5,829,821 / 8.573%	248,726 / 0.366%
BLU-A-2_S11_L001_R_Merged	5,828,514 / 8.571%	249,110 / 0.366%
BLU-A-3_S12_L001_R_Merged	5,832,237 / 8.577%	249,316 / 0.367%
CONTROL-1_S1_L001_R_Merged	6,281,283 / 9.237%	291,298 / 0.428%
CONTROL-2_S2_L001_R_Merged	6,284,898 / 9.242%	291,107 / 0.428%
CONTROL-3_S3_L001_R_Merged	6,284,741 / 9.242%	291,173 / 0.428%

Chimeric Reads

Name	Chimeric reads
BLA-1_S7_L001_R_Merged	0 / 0%
BLA-2_S8_L001_R_Merged	0 / 0%
BLA-3_S9_L001_R_Merged	0 / 0%
BLA-A-1_S13_L001_R_Merged	0 / 0%
BLA-A-2_S14_L001_R_Merged	0 / 0%
BLA-A-3_S15_L001_R1_001_Merged	0 / 0%
BLU-1_S4_L001_R_Merged	0 / 0%
BLU-2_S5_L001_R_Merged	0 / 0%
BLU-3_S6_L001_R_Merged	0 / 0%
BLU-A-1S10_L001_R_Merged	0 / 0%
BLU-A-2_S11_L001_R_Merged	0 / 0%
BLU-A-3_S12_L001_R_Merged	0 / 0%
CONTROL-1_S1_L001_R_Merged	0 / 0%
CONTROL-2_S2_L001_R_Merged	0 / 0%
CONTROL-3_S3_L001_R_Merged	0 / 0%

Unmapped Reads

Name	Reads Unmapped: Too many mismatches	Reads Unmapped: Too short	Reads Unmapped: Other
BLA-1_S7_L001_R_Merged	0 / 0%	6,160,800 / 9.06%	367,200 / 0.54%
BLA-2_S8_L001_R_Merged	0 / 0%	6,160,800 / 9.06%	367,200 / 0.54%
BLA-3_S9_L001_R_Merged	0 / 0%	6,160,800 / 9.06%	367,200 / 0.54%
BLA-A-1_S13_L001_R_Merged	0 / 0%	8,221,200 / 12.09%	258,400 / 0.38%
BLA-A-2_S14_L001_R_Merged	0 / 0%	8,228,000 / 12.1%	258,400 / 0.38%
BLA-A-3_S15_L001_R1_001_Merged	0 / 0%	8,228,000 / 12.1%	258,400 / 0.38%
BLU-1_S4_L001_R_Merged	0 / 0%	5,433,200 / 7.99%	374,000 / 0.55%
BLU-2_S5_L001_R_Merged	0 / 0%	5,440,000 / 8%	374,000 / 0.55%
BLU-3_S6_L001_R_Merged	0 / 0%	5,433,200 / 7.99%	374,000 / 0.55%
BLU-A-1S10_L001_R_Merged	0 / 0%	8,024,000 / 11.8%	340,000 / 0.5%
BLU-A-2_S11_L001_R_Merged	0 / 0%	8,024,000 / 11.8%	340,000 / 0.5%
BLU-A-3_S12_L001_R_Merged	0 / 0%	8,024,000 / 11.8%	340,000 / 0.5%
CONTROL-1_S1_L001_R_Merged	0 / 0%	7,126,400 / 10.48%	346,800 / 0.51%
CONTROL-2_S2_L001_R_Merged	0 / 0%	7,126,400 / 10.48%	346,800 / 0.51%
CONTROL-3_S3_L001_R_Merged	0 / 0%	7,133,200 / 10.49%	346,800 / 0.51%

Analysis Parameters

Parameter	Value
Provide Annotations	true
Annotation File	D:\Complete Reference\ncbi_dataset\data\GCF_000001635.27\genomic.gtf
Exon Feature	transcript
Overhang	100
2-pass Mapping	false
Sort by Coordinate	true
Min. Intron Length	20
Max. Intron Length	1000000
Max. # of Multiple Alignments	20
Max. # of Mismatches	999
Include Chimeric Alignments	false
Add Read Group Information	false
Save Splice Junctions	true
Save Unmapped Reads	false

References

- OmicsBox - Bioinformatics made easy. BioBam Bioinformatics. March 3, 2019. www.biobam.com/omicsbox.
- Dobin A., Davis CA., Schlesinger F., Drenkow J., Zaleski C., Jha S., Batut P., Chaisson M. and Gingeras TR. (2013). STAR: ultrafast universal RNA-seq aligner. *Bioinformatics (Oxford, England), 29*(1), 15-21.

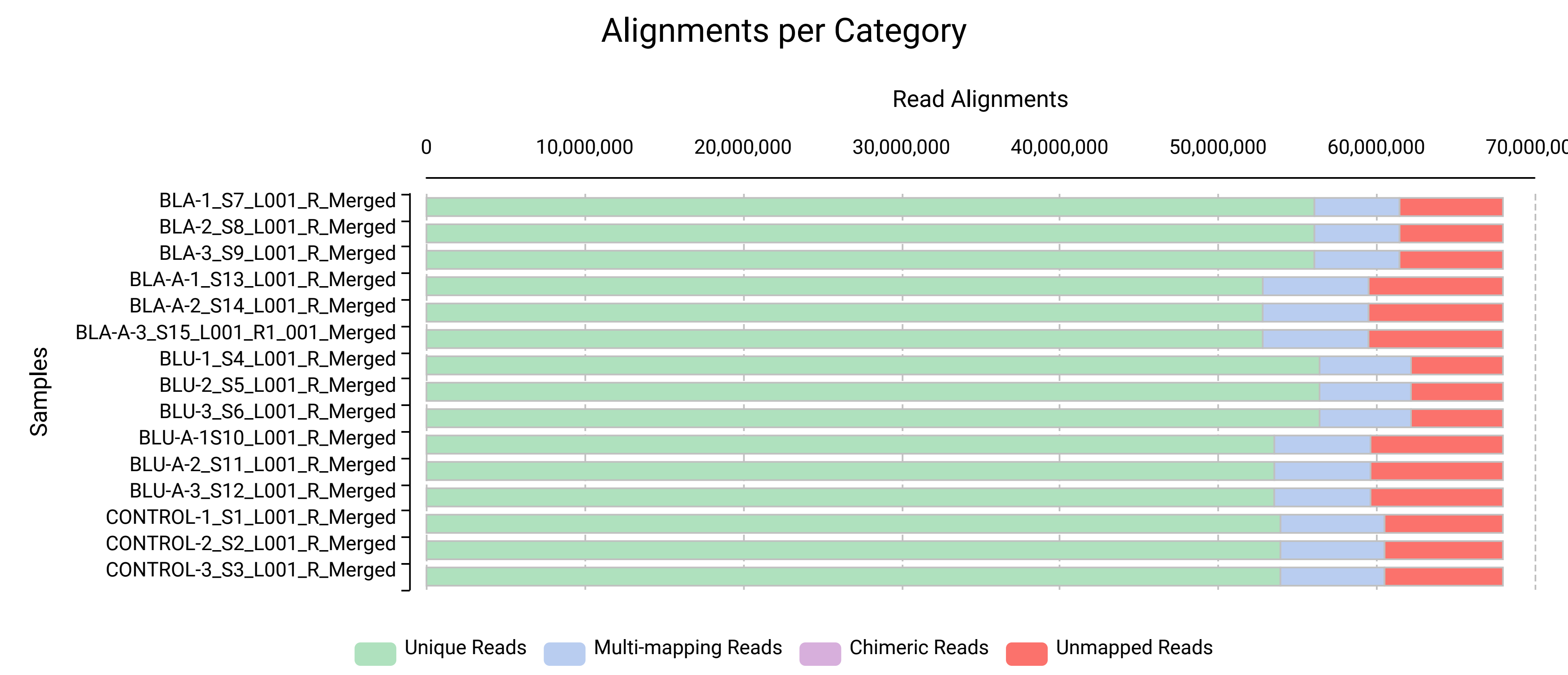

Alignments per Category
Read Alignments
0
10,000,000
20,000,000
30,000,000
40,000,000
50,000,000
60,000,000
70,000,00
Samples
BLA-1_S7_L001_R_Merged
BLA-2_S8_L001_R_Merged
BLA-3_S9_L001_R_Merged
BLA-A-1_S13_L001_R_Merged
BLA-A-2_S14_L001_R_Merged
BLA-A-3_S15_L001_R1_001_Merged
BLU-1_S4_L001_R_Merged
BLU-2_S5_L001_R_Merged
BLU-3_S6_L001_R_Merged
BLU-A-1S10_L001_R_Merged
BLU-A-2_S11_L001_R_Merged
BLU-A-3_S12_L001_R_Merged
CONTROL-1_S1_L001_R_Merged
CONTROL-2_S2_L001_R_Merged
CONTROL-3_S3_L001_R_Merged
Unique Reads
Multi-mapping Reads
Chimeric Reads
Unmapped Reads

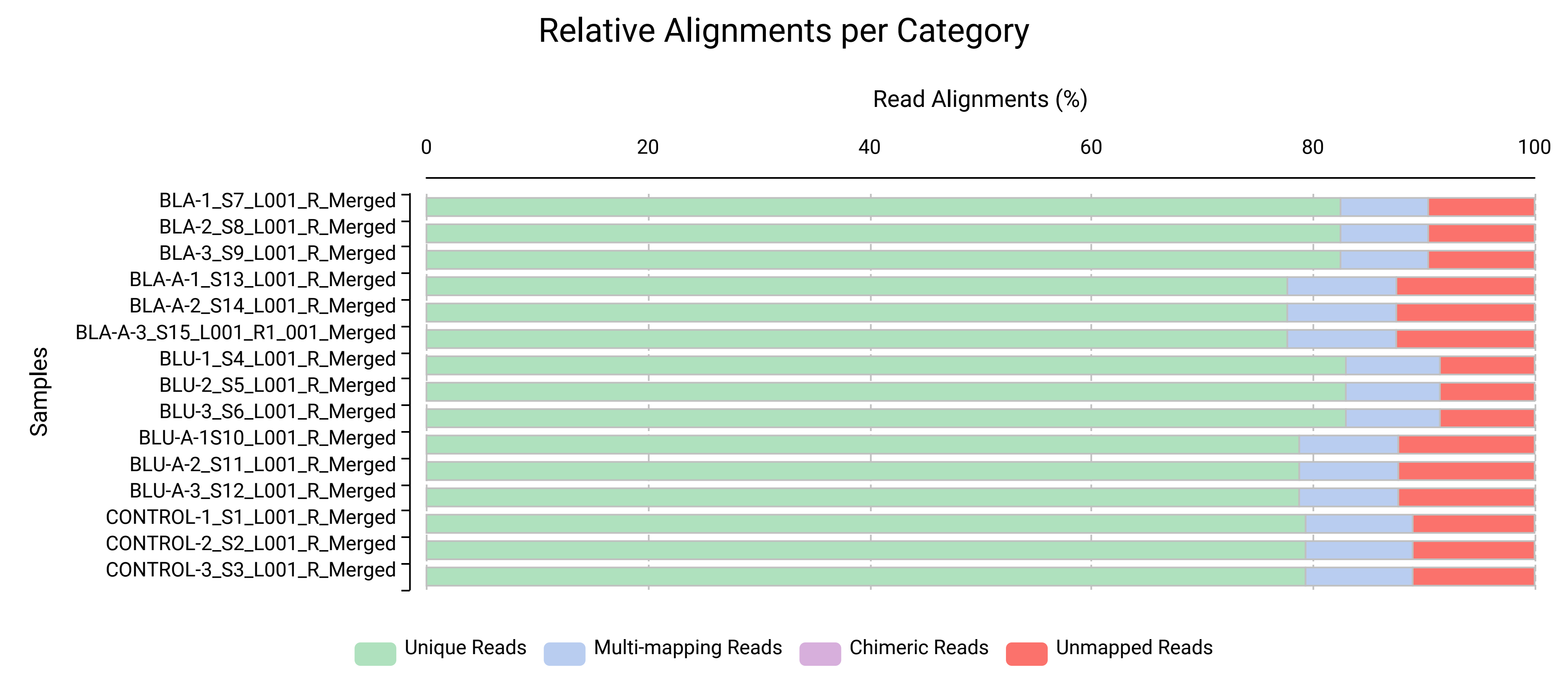
Relative Alignments per Category
Read Alignments (%)
0
20
40
60
80
100
Samples
BLA-1_S7_L001_R_Merged
BLA-2_S8_L001_R_Merged
BLA-3_S9_L001_R_Merged
BLA-A-1_S13_L001_R_Merged
BLA-A-2_S14_L001_R_Merged
BLA-A-3_S15_L001_R1_001_Merged
BLU-1_S4_L001_R_Merged
BLU-2_S5_L001_R_Merged
BLU-3_S6_L001_R_Merged
BLU-A-1S10_L001_R_Merged
BLU-A-2_S11_L001_R_Merged
BLU-A-3_S12_L001_R_Merged
CONTROL-1_S1_L001_R_Merged
CONTROL-2_S2_L001_R_Merged
CONTROL-3_S3_L001_R_Merged
Unique Reads
Multi-mapping Reads
Chimeric Reads
Unmapped Reads

OBJECTIVE

Bioinformatics Analysis of 4TI dataset derived from RNA-seq of mouse cell lines.

TEST ARTICLES AND TEST SYSTEM

1. Published reference genome of *Mus musculus* genome assembly GRCm39 on Gencode with accession # GCA_000001635.9 as reference sequence.

Data Analysis Software

1. DRAGEN RNA Pipeline version 4.4.4002
2. DRAGEN Differential Expression Pipeline version 4.3.7

TESTING PROCEDURE

1. Sequencing reads were aligned to the published *Mus musculus* reference genome. Mapping rates and coverage statistics of the samples were reported.

2. Gene expression levels were quantified and compared between test and control samples. Differentially expressed genes were reported.

RESULTS

Bioinformatics

68 million reads were generated for each sample with adapter bases and minimum length <32 bp removed ({ACGT_ID}_S{1-15}_R1_001.fastq.gz and {ACGT_ID}_S{1-15}_R2_001.fastq.gz). The sequenced reads of the samples were mapped to the published *Mus Musculus* reference genome obtained from Gencode ({ACGT_ID}.bam and {ACGT_ID}.bam.bai). Mapping rates above 92% were observed for the samples (Table 2). Variants against the reference were called by enabling variant calling option ({ACGT_ID}.variant.vcf.gz).

Gene fusion detection was performed using the DRAGEN RNA splice-aware aligner. Detected fusion events are summarized in {ACGT_ID}.fusion_candidates.features.csv. Final pass-filtered calls are provided in {ACGT_ID}.fusion_candidates.final, which lists each passing fusion along with the names of supporting reads—split reads, soft-clipped reads, and discordant read pairs—and their passing scores. The chimeric alignments are used to find potential breakpoints and read evidence is accumulated for the resulting fusion event candidates. Information about chimeric alignments and high confidence splice junctions were provided in the deliverables ({ACGT_ID}.Chimeric.out.junction and {ACGT_ID}.SJ.out.tab).

Transcript quantification in table 4 summarizes expression at the transcript level sorted by transcripts per million (TPM) normalized for both transcript length and sequencing depth and estimated read count per transcript before normalization ({ACGT_ID}.quant.sf).

Quantification results at gene level were obtained by summing together all transcripts that share same gene ID in the annotation file. Table 5 lists the top 10 gene IDs, sorted by Transcripts per Million (TPM), along with estimated number of reads and length of the spliced transcript, where the length is represented as expression-weighted means of the individual transcripts in the gene ({ACGT_ID}.quant.genes.sf).

Differential gene expression analysis was performed statistically based on quantification of transcript expression levels, and each of the samples 864691-2 through 864691-5 were compared with control sample 864691-1. In each sample pair comparison, approximately 2X more genes were observed to be up

regulated than down regulated in the test samples compared to the control sample expect for 864691-5 Vs 864691-1 comparison. Most significant up and down regulated genes in each comparison were reported in Table 7, ordered by adjusted p value. For each sample, the top three genes were evaluated by comparing their expression across all other samples (Figure 4). Complete differential gene expression analysis files were provided in the Deliverables (“Differential Expression Analysis” folder).

DELIVERABLES

SUBSET READS

864691-1A_S1_L001_R1_001.fastq.gz
864691-1A_S1_L001_R2_001.fastq.gz

864691-1B_S2_L001_R1_001.fastq.gz
864691-1B_S2_L001_R2_001.fastq.gz

864691-1C_S3_L001_R1_001.fastq.gz
864691-1C_S3_L001_R2_001.fastq.gz

864691-2A_S4_L001_R1_001.fastq.gz
864691-2A_S4_L001_R2_001.fastq.gz

864691-2B_S5_L001_R1_001.fastq.gz
864691-2B_S5_L001_R2_001.fastq.gz

864691-2C_S6_L001_R1_001.fastq.gz
864691-2C_S6_L001_R2_001.fastq.gz

864691-3A_S7_L001_R1_001.fastq.gz
864691-3A_S7_L001_R2_001.fastq.gz

864691-3B_S8_L001_R1_001.fastq.gz
864691-3B_S8_L001_R2_001.fastq.gz

864691-3C_S9_L001_R1_001.fastq.gz
864691-3C_S9_L001_R2_001.fastq.gz

864691-4A_S10_L001_R1_001.fastq.gz
864691-4A_S10_L001_R2_001.fastq.gz

864691-4B_S11_L001_R1_001.fastq.gz
864691-4B_S11_L001_R2_001.fastq.gz

864691-4C_S12_L001_R1_001.fastq.gz
864691-4C_S12_L001_R2_001.fastq.gz

864691-5A_S13_L001_R1_001.fastq.gz
864691-5A_S13_L001_R2_001.fastq.gz

864691-5B_S14_L001_R1_001.fastq.gz
864691-5B_S14_L001_R2_001.fastq.gz
864691-5C_S15_L001_R1_001.fastq.gz
864691-5C_S15_L001_R2_001.fastq.gz

REFERENCE SEQUENCE and ANNOTATION FILE (Folder)

ALIGNMENT TO REFERENCE

864691-1A.bam
864691-1A.bam.bai

864691-1B.bam
864691-1B.bam.bai

864691-1C.bam
864691-1C.bam.bai

864691-2A.bam
864691-2A.bam.bai

864691-2B.bam
864691-2B.bam.bai

864691-2C.bam
864691-2C.bam.bai

864691-3A.bam
864691-3A.bam.bai

864691-3B.bam
864691-3B.bam.bai

864691-3C.bam
864691-3C.bam.bai

864691-4A.bam
864691-4A.bam.bai

864691-4B.bam
864691-4B.bam.bai
864691-4C.bam
864691-4C.bam.bai

864691-5A.bam
864691-5A.bam.bai

864691-5B.bam
864691-5B.bam.bai

864691-5C.bam
864691-5C.bam.bai

VARIANT CALLING

864691-1A.variant.vcf.gz
864691-1B.variant.vcf.gz
864691-1C.variant.vcf.gz

864691-2A.variant.vcf.gz
864691-2B.variant.vcf.gz
864691-2C.variant.vcf.gz

864691-3A.variant.vcf.gz
864691-3B.variant.vcf.gz
864691-3C.variant.vcf.gz

864691-4A.variant.vcf.gz
864691-4B.variant.vcf.gz
864691-4C.variant.vcf.gz

864691-5A.variant.vcf.gz
864691-5B.variant.vcf.gz
864691-5C.variant.vcf.gz

SPLICE JUNCTION

864691-1A.SJ.out.tab
864691-1B.SJ.out.tab
864691-1C.SJ.out.tab

864691-2A.SJ.out.tab
864691-2B.SJ.out.tab
864691-2C.SJ.out.tab

864691-3A.SJ.out.tab
864691-3B.SJ.out.tab
864691-3C.SJ.out.tab

864691-4A.SJ.out.tab
864691-4B.SJ.out.tab
864691-4C.SJ.out.tab

864691-5A.SJ.out.tab
864691-5B.SJ.out.tab
864691-5C.SJ.out.tab

CHIMERIC ALIGNMENTS

864691-1A.Chimeric.out.junction
864691-1B.Chimeric.out.junction
864691-1C.Chimeric.out.junction

864691-2A.Chimeric.out.junction
864691-2B.Chimeric.out.junction
864691-2C.Chimeric.out.junction

864691-3A.Chimeric.out.junction
864691-3B.Chimeric.out.junction
864691-3C.Chimeric.out.junction

864691-4A.Chimeric.out.junction
864691-4B.Chimeric.out.junction
864691-4C.Chimeric.out.junction

864691-5A.Chimeric.out.junction
864691-5B.Chimeric.out.junction
864691-5C.Chimeric.out.junction

RNA GENE FUSION DETECTION

864691-1A.fusion_candidates.features.csv
864691-1A.fusion_candidates.final

864691-1B.fusion_candidates.features.csv
864691-1B.fusion_candidates.final

864691-1C.fusion_candidates.features.csv
864691-1C.fusion_candidates.final

864691-2A.fusion_candidates.features.csv
864691-2A.fusion_candidates.final

864691-2B.fusion_candidates.features.csv
864691-2B.fusion_candidates.final

864691-2C.fusion_candidates.features.csv
864691-2C.fusion_candidates.final

864691-3A.fusion_candidates.features.csv
864691-3A.fusion_candidates.final

864691-3B.fusion_candidates.features.csv
864691-3B.fusion_candidates.final

864691-3C.fusion_candidates.features.csv
864691-3C.fusion_candidates.final

864691-4A.fusion_candidates.features.csv
864691-4A.fusion_candidates.final

864691-4B.fusion_candidates.features.csv
864691-4B.fusion_candidates.final

864691-4C.fusion_candidates.features.csv
864691-4C.fusion_candidates.final

864691-5A.fusion_candidates.features.csv
864691-5A.fusion_candidates.final

864691-5B.fusion_candidates.features.csv
864691-5B.fusion_candidates.final

864691-5C.fusion_candidates.features.csv
864691-5C.fusion_candidates.final

TRANSCRIPT QUANTIFICATION RESULTS

864691-1A.quant.sf
864691-1B.quant.sf
864691-1C.quant.sf

864691-2A.quant.sf
864691-2B.quant.sf
864691-2C.quant.sf

864691-3A.quant.sf
864691-3B.quant.sf
864691-3C.quant.sf

864691-4A.quant.sf
864691-4B.quant.sf
864691-4C.quant.sf

864691-5A.quant.sf
864691-5B.quant.sf
864691-5C.quant.sf

GENE QUANTIFICATION RESULTS

864691-1A.quant.genes.sf
864691-1B.quant.genes.sf
864691-1C.quant.genes.sf

864691-2A.quant.genes.sf
864691-2B.quant.genes.sf
864691-2C.quant.genes.sf

864691-3A.quant.genes.sf
864691-3B.quant.genes.sf
864691-3C.quant.genes.sf

864691-4A.quant.genes.sf
864691-4B.quant.genes.sf
864691-4C.quant.genes.sf

864691-5A.quant.genes.sf
864691-5B.quant.genes.sf
864691-5C.quant.genes.sf

DIFFERENTIAL EXPRESSION ANALYSIS

Differential Expression Analysis

TABLES AND FIGURES

Table 1: ACGT ID Designation

Sample ID	ACGT ID
4T1 CELLS CONTROL	864691-1
4T1 CELLS WITH BLUEBERRY	864691-2

4T1 CELLS WITH BLACKBERRY	864691-3
4T1 CELLS WITH BLUEBERRY GOLD NANOPARTICLES	864691-4
4T1 CELLS WITH BLACKBERRYGOLD NANOPARTICLES	864691-5

Table 2: Bioinformatics Summary of Analyzed Samples

ACGT ID	Raw Reads	Subset Reads	Mapping Rate (%)	Number of Genes with >=10X coverage
864691-1A	90,912,166	68,000,000	93.80%	10,117
864691-1B		68,000,000	93.80%	10,110
864691-1C		68,000,000	93.80%	10,120
864691-2A	89,702,254	68,000,000	95.53%	10,066
864691-2B		68,000,000	95.53%	10,048
864691-2C		68,000,000	95.53%	10,048
864691-3A	75,228,930	68,000,000	95.00%	10,087
864691-3B		68,000,000	95.00%	10,097
864691-3C		68,000,000	95.00%	10,090
864691-4A	101,383,412	68,000,000	92.40%	10,234
864691-4B		68,000,000	92.40%	10,227
864691-4C		68,000,000	92.40%	10,223
864691-5A	129,957,416	68,000,000	97.28%	9,936
864691-5B		68,000,000	97.28%	9,929
864691-5C		68,000,000	97.28%	9,940

Table 3: Variant Summary of Analyzed Samples

ACGT ID	Number of Variants	Number of SNPs	Ratio of Transitions to Tranversions (Ti/Tv)	Ratio of Homozygous to Heterozygous variants
864691-1A	109,135	94,211	2.44	0.08

864691-1B	108,749	93,966	2.43	0.08
864691-1C	108,712	93,895	2.43	0.08
864691-2A	129,901	111,739	2.38	0.07
864691-2B	129,817	111,785	2.38	0.07
864691-2C	129,586	111,444	2.38	0.07
864691-3A	103,213	89,492	2.43	0.08
864691-3B	103,077	89,376	2.43	0.08
864691-3C	103,315	89,570	2.43	0.08
864691-4A	145,651	125,087	2.38	0.07
864691-4B	145,753	125,259	2.39	0.07
864691-4C	145,817	125,307	2.38	0.07
864691-5A	297,111	243,722	2.30	0.03
864691-5B	296,979	243,490	2.29	0.03
864691-5C	297,534	243,995	2.29	0.03

Table 4: Transcript Expression Quantification (Top 10)

864691-1A

Name	Length	EffectiveLength	TPM	NumReads
ENSMUST00020183253.1	331	89.2	31693.59	47565.22
ENSMUST00000175032.4	300	75.1	18396.11	23224.92
ENSMUST00000143570.2	505	238.6	17572.34	70514.3
ENSMUST00000336252.1	259	60.2	16528.5	16749
ENSMUST00000292750.1	302	75.9	11799.51	15062.5
ENSMUST00000084013.1	297	73.9	10000.78	12425
ENSMUST00000082407.1	204	46.7	9318.698	7313
ENSMUST00000042235.15	1794	1602.2	8979.554	241980

ENSMUST00000082402.1	1545	1254.7	8857.866	186937
ENSMUST00000240377.1	1849	1560.9	8745.807	229607

864691-1B

Name	Length	EffectiveLength	TPM	NumReads
ENSMUST00020183253.1	331	89.3	31597.26	47467.86
ENSMUST00000175032.4	300	75.1	18114.43	22864.98
ENSMUST00000143570.2	505	239	17645	70925.69
ENSMUST00000336252.1	259	60.2	16432.73	16639
ENSMUST00000292750.1	302	75.9	12068.81	15403.33
ENSMUST00000084013.1	297	73.9	10091.29	12533.48
ENSMUST00000082407.1	204	46.6	9383.579	7352
ENSMUST00000042235.15	1794	1603.7	8957.586	241584.9
ENSMUST00000082402.1	1545	1254.7	8868.631	187137.7
ENSMUST00000240377.1	1849	1561.7	8727.093	229196

864691-1C

Name	Length	EffectiveLength	TPM	NumReads
ENSMUST00020183253.1	331	89.3	31694.4	47609.72
ENSMUST00000175032.4	300	75	18801.9	23727.04
ENSMUST00000143570.2	505	238.5	17644.18	70828.42
ENSMUST00000336252.1	259	60.3	16323.73	16563
ENSMUST00000292750.1	302	75.8	11509.78	14690.52
ENSMUST00000084013.1	297	73.8	10024.66	12449.66
ENSMUST00000082407.1	204	46.7	9398.144	7389
ENSMUST00000042235.15	1794	1601.9	8964.023	241647.8
ENSMUST00000082402.1	1545	1254.6	8857.818	187018.3
ENSMUST00000240377.1	1849	1561.1	8732.57	229406

864691-2A

Name	Length	EffectiveLength	TPM	NumReads
ENSMUST00020183253.1	331	91.6	27762.08	33951.38
ENSMUST00000143570.2	505	241.9	18192.88	58741.63
ENSMUST00000336252.1	259	60.7	16463.13	13333
ENSMUST00000175032.4	300	76.4	11630.18	11858.75
ENSMUST00000084013.1	297	75.1	10020.83	10043.57
ENSMUST00000240377.1	1849	1520.4	9431.554	191440
ENSMUST00000042235.15	1794	1602.8	9171.319	196243.8
ENSMUST00000082407.1	204	46	9096.423	5591
ENSMUST00000082402.1	1545	1305.3	8882.988	154793.3
ENSMUST00000292750.1	302	77.2	6417.337	6618.15

864691-2B

Name	Length	EffectiveLength	TPM	NumReads
ENSMUST00020183253.1	331	91.5	27904.44	34086.86
ENSMUST00000143570.2	505	241.9	18085.16	58425.7
ENSMUST00000336252.1	259	60.4	16405.29	13231
ENSMUST00000175032.4	300	76.2	11707.06	11907.99
ENSMUST00000084013.1	297	74.8	10110.01	10106.75
ENSMUST00000240377.1	1849	1521.1	9427.734	191539
ENSMUST00000082407.1	204	45.7	9240.949	5635
ENSMUST00000042235.15	1794	1603.4	9188.523	196781.8
ENSMUST00000082402.1	1545	1305	8890.766	154975
ENSMUST00000292750.1	302	77	6275.271	6456.11

864691-2C

Name	Length	EffectiveLength	TPM	NumReads
ENSMUST00020183253.1	331	91.4	27783.07	33916.96
ENSMUST00000143570.2	505	241.9	18064.41	58357.35
ENSMUST00000336252.1	259	60.3	16467.7	13253
ENSMUST00000175032.4	300	76.1	11547.29	11741.76
ENSMUST00000084013.1	297	74.8	10123.6	10117.17
ENSMUST00000240377.1	1849	1521	9412.818	191211
ENSMUST00000082407.1	204	45.7	9313.287	5682
ENSMUST00000042235.15	1794	1603.4	9210.212	197234.2
ENSMUST00000082402.1	1545	1305.1	8878.829	154763.1
ENSMUST00000292750.1	302	77	6387.229	6568.93

864691-3A

Name	Length	EffectiveLength	TPM	NumReads
ENSMUST00020183253.1	331	93.8	33854.4	40234.53
ENSMUST00000336252.1	259	60.6	23443.82	18002
ENSMUST00000292750.1	302	78.3	21150.29	21002.07
ENSMUST00000143570.2	505	249	21052.59	66444.53
ENSMUST00000240377.1	1849	1537.4	15274.52	297647
ENSMUST00000042235.15	1794	1610.6	8499.724	173517.4
ENSMUST00000082402.1	1545	1308.2	8016.877	132930.9
ENSMUST00000175032.4	300	77.4	7107.105	6969.19
ENSMUST00000000756.6	780	449	5900.238	33582.28
ENSMUST00000084013.1	297	75.9	5790.157	5574.02

864691-3B

Name	Length	EffectiveLength	TPM	NumReads
ENSMUST00020183253.1	331	93.7	33902.19	40264.22
ENSMUST00000336252.1	259	60.6	23406.04	17970
ENSMUST00000292750.1	302	78.3	21060.01	20903.18
ENSMUST00000143570.2	505	248.9	21008.4	66274.91
ENSMUST00000240377.1	1849	1537.1	15289.48	297879
ENSMUST00000042235.15	1794	1610.6	8504.443	173614.6
ENSMUST00000082402.1	1545	1308.1	8040.905	133326.9
ENSMUST00000175032.4	300	77.3	7165.066	7023.91
ENSMUST00000000756.6	780	448.8	5918.003	33669.32
ENSMUST00000084013.1	297	75.9	5703.873	5490.43

864691-3C

Name	Length	EffectiveLength	TPM	NumReads
ENSMUST00020183253.1	331	93.6	33938.04	40292.17
ENSMUST00000336252.1	259	60.5	23337.63	17913
ENSMUST00000292750.1	302	78.2	21059.98	20891.75
ENSMUST00000143570.2	505	248.9	21044.6	66407.34
ENSMUST00000240377.1	1849	1537.2	15270.34	297594
ENSMUST00000042235.15	1794	1610.7	8494.328	173454.8
ENSMUST00000082402.1	1545	1308.1	8027.778	133128.2
ENSMUST00000175032.4	300	77.3	7236.525	7090.4
ENSMUST00000000756.6	780	448.9	5903.225	33596.94
ENSMUST00000084013.1	297	75.9	5800.024	5578.88

864691-4A

Name	Length	EffectiveLength	TPM	NumReads
ENSMUST00020183253.1	331	94.3	35700.59	52560.69
ENSMUST00000292750.1	302	80.1	28980.45	36271.6
ENSMUST00000175032.4	300	79.3	27347.05	33861.68
ENSMUST00000143570.2	505	246.3	20079.51	77248.27
ENSMUST00000084013.1	297	78	12569.57	15320.3
ENSMUST00000082407.1	204	49	10186.04	7795
ENSMUST00000082402.1	1545	1254.2	10119.26	198224.8
ENSMUST00000336252.1	259	63.8	10092.28	10052
ENSMUST00000042235.15	1794	1610.9	8718.11	219354.5
ENSMUST00000081342.7	482	208.5	7666.246	24961.61

864691-4B

Name	Length	EffectiveLength	TPM	NumReads

ENSMUST00020183253.1	331	94.3	35706.98	52578.75
ENSMUST00000292750.1	302	80	28836.6	36040.62
ENSMUST00000175032.4	300	79.2	27579.42	34095.39
ENSMUST00000143570.2	505	246.1	20015.73	76913.25
ENSMUST00000084013.1	297	78	12377.62	15066.88
ENSMUST00000082402.1	1545	1253.7	10119.17	198104.1
ENSMUST00000336252.1	259	63.7	10047.02	9997
ENSMUST00000082407.1	204	49	10046.04	7692
ENSMUST00000042235.15	1794	1609.9	8723.933	219306.8
ENSMUST00000081342.7	482	208.4	7643.466	24877.12

864691-4C

Name	Length	EffectiveLength	TPM	NumReads
ENSMUST00020183253.1	331	94.3	35665.64	52550.71
ENSMUST00000292750.1	302	80.1	30138.69	37706.78
ENSMUST00000175032.4	300	79.2	26355.64	32615.47
ENSMUST00000143570.2	505	246.4	20097.5	77341.34
ENSMUST00000084013.1	297	78	12541.53	15277.92
ENSMUST00000082402.1	1545	1254	10122.82	198276.4
ENSMUST00000336252.1	259	63.8	10084.3	10047
ENSMUST00000082407.1	204	49	10053.13	7697
ENSMUST00000042235.15	1794	1611.5	8696.862	218906.2
ENSMUST00000081342.7	482	208.4	7595.325	24726.77

864691-5A

Name	Length	EffectiveLength	TPM	NumReads
ENSMUST00020183253.1	331	120.6	141083	339955.2
ENSMUST00000174924.4	300	95.8	57410.63	109872.9
ENSMUST00000175032.4	300	95.8	48647.36	93101.66
ENSMUST00000082407.1	204	30.8	31664.07	19454
ENSMUST00000175096.3	319	111.1	22253.7	49359
ENSMUST00000157463.2	271	75	15604.61	23374.79
ENSMUST00000082402.1	1545	1261.2	14576.61	367191.6
ENSMUST00000082408.1	681	427.2	12804.1	109252.4
ENSMUST00000082392.1	957	692	9411.26	130068
ENSMUST00000082405.1	684	435.5	9334.608	81185.99

864691-5B

Name	Length	EffectiveLength	TPM	NumReads
ENSMUST00020183253.1	331	120.7	141527.1	340752.7
ENSMUST00000174924.4	300	95.9	57053.44	109198.8

ENSMUST00000175032.4	300	95.9	48987.06	93759.99
ENSMUST00000082407.1	204	30.8	31445.3	19340
ENSMUST00000175096.3	319	111.2	22233.97	49333
ENSMUST00000157463.2	271	75.2	15468.98	23199.52
ENSMUST00000082402.1	1545	1260.9	14581.18	366861.3
ENSMUST00000082408.1	681	427.4	12796.48	109138.3
ENSMUST00000082392.1	957	692.1	9429.087	130218
ENSMUST00000082405.1	684	435.5	9365.673	81388.28

864691-5C

Name	Length	EffectiveLength	TPM	NumReads
ENSMUST00020183253.1	331	120.7	140995.2	339878.1
ENSMUST00000174924.4	300	95.9	56573.78	108372
ENSMUST00000175032.4	300	95.9	49120.44	94094.45
ENSMUST00000082407.1	204	30.8	31801.82	19544.71
ENSMUST00000175096.3	319	111.3	22182.73	49309
ENSMUST00000157463.2	271	75.1	15531.99	23314.58
ENSMUST00000082402.1	1545	1261	14538.15	366283.7
ENSMUST00000082408.1	681	427.2	12771.77	109003.8
ENSMUST00000082392.1	957	691.8	9412.918	130110
ENSMUST00000082405.1	684	435.4	9363.403	81454.78

Table 5: Gene Expression Quantification (Top 10)

864691-1A

Name	Length	EffectiveLength	TPM	NumReads
ENSMUSG00002076161.1	331	89.23	31693.59	47565.22
ENSMUSG00000037742.15	941	699.41	26692.03	313999.7
ENSMUSG00000118866.1	300	75.06	18396.11	23224.92
ENSMUSG00000136525.1	259	60.25	16528.5	16749
ENSMUSG00000129312.1	302	75.9	11799.51	15062.5
ENSMUSG00000065947.1	297	73.87	10000.78	12425
ENSMUSG00000064356.1	204	46.66	9318.698	7313
ENSMUSG00000064351.1	1545	1254.73	8857.866	186937
ENSMUSG00000119584.1	1849	1560.88	8745.807	229607
ENSMUSG00000094777.3	482	202.05	8412.023	28588.04

864691-1B

Name	Length	EffectiveLength	TPM	NumReads
ENSMUSG00002076161.1	331	89.33	31597.26	47467.86
ENSMUSG00000037742.15	939	698.24	26742.27	314015.9

ENSMUSG00000118866.1	300	75.06	18114.43	22864.98
ENSMUSG00000136525.1	259	60.21	16432.73	16639
ENSMUSG00000129312.1	302	75.89	12068.81	15403.33
ENSMUSG00000065947.1	297	73.85	10091.29	12533.48
ENSMUSG00000064356.1	204	46.59	9383.579	7352
ENSMUSG00000064351.1	1545	1254.75	8868.631	187137.7
ENSMUSG00000119584.1	1849	1561.67	8727.093	229196
ENSMUSG00000094777.3	482	202.17	8414.709	28608.86

864691-1C

Name	Length	EffectiveLength	TPM	NumReads
ENSMUSG00002076161.1	331	89.26	31694.4	47609.72
ENSMUSG00000037742.15	939	697.53	26751.83	314020.2
ENSMUSG00000118866.1	300	74.99	18801.9	23727.04
ENSMUSG00000136525.1	259	60.29	16323.73	16563
ENSMUSG00000129312.1	302	75.85	11509.78	14690.52
ENSMUSG00000065947.1	297	73.8	10024.66	12449.66
ENSMUSG00000064356.1	204	46.72	9398.144	7389
ENSMUSG00000064351.1	1545	1254.63	8857.818	187018.3
ENSMUSG00000119584.1	1849	1561.06	8732.57	229406
ENSMUSG00000094777.3	482	202.1	8431.025	28673.87

864691-2A

Name	Length	EffectiveLength	TPM	NumReads
ENSMUSG00002076161.1	331	91.6	27762.08	33951.38
ENSMUSG00000037742.15	937	697.65	27510.08	256228.5
ENSMUSG00000136525.1	259	60.66	16463.13	13333
ENSMUSG00000118866.1	300	76.38	11630.18	11858.75
ENSMUSG00000065947.1	297	75.07	10020.83	10043.57
ENSMUSG00000119584.1	1849	1520.39	9431.554	191440
ENSMUSG00000064356.1	204	46.04	9096.423	5591
ENSMUSG00000064351.1	1545	1305.26	8882.988	154793.3
ENSMUSG00000057322.13	370	116.05	7080.148	10969
ENSMUSG00000022283.16	1753	1365.58	6422.524	117090.1

864691-2B

Name	Length	EffectiveLength	TPM	NumReads
ENSMUSG00002076161.1	331	91.46	27904.44	34086.86
ENSMUSG00000037742.15	939	700.24	27417.42	256433.2
ENSMUSG00000136525.1	259	60.38	16405.29	13231
ENSMUSG00000118866.1	300	76.15	11707.06	11907.99

ENSMUSG00000065947.1	297	74.84	10110.01	10106.75
ENSMUSG00000119584.1	1849	1521.08	9427.734	191539
ENSMUSG00000064356.1	204	45.65	9240.949	5635
ENSMUSG00000064351.1	1545	1305.04	8890.766	154975
ENSMUSG00000057322.13	370	115.88	7071.893	10946
ENSMUSG00000022283.16	1755	1367.33	6402.854	116935.5

864691-2C

Name	Length	EffectiveLength	TPM	NumReads
ENSMUSG00002076161.1	331	91.4	27783.07	33916.96
ENSMUSG00000037742.15	940	701.3	27423.41	256861.8
ENSMUSG00000136525.1	259	60.26	16467.7	13253
ENSMUSG00000118866.1	300	76.13	11547.29	11741.76
ENSMUSG00000065947.1	297	74.83	10123.6	10117.17
ENSMUSG00000119584.1	1849	1520.97	9412.818	191211
ENSMUSG00000064356.1	204	45.68	9313.287	5682
ENSMUSG00000064351.1	1545	1305.09	8878.829	154763.1
ENSMUSG00000057322.13	370	115.92	7022.646	10873
ENSMUSG00000022283.16	1749	1361.6	6424.521	116832.1

864691-3A

Name	Length	EffectiveLength	TPM	NumReads
ENSMUSG00002076161.1	331	93.76	33854.4	40234.53
ENSMUSG00000037742.15	876	640.6	29696.61	241129.8
ENSMUSG00000136525.1	259	60.58	23443.82	18002
ENSMUSG00000129312.1	302	78.34	21150.29	21002.07
ENSMUSG00000119584.1	1849	1537.37	15274.52	297647
ENSMUSG00000064351.1	1545	1308.17	8016.877	132930.9
ENSMUSG00000022283.16	1511	1129.12	7894.401	112983.3
ENSMUSG00000118866.1	300	77.36	7107.105	6969.19
ENSMUSG00000038274.14	597	294.6	6895.513	25749.04
ENSMUSG00000057322.13	369	118.38	6540.899	9814.97

864691-3B

Name	Length	EffectiveLength	TPM	NumReads
ENSMUSG00002076161.1	331	93.7	33902.19	40264.22
ENSMUSG00000037742.15	876	641.25	29654.48	241034.2
ENSMUSG00000136525.1	259	60.57	23406.04	17970
ENSMUSG00000129312.1	302	78.31	21060.01	20903.18
ENSMUSG00000119584.1	1849	1537.05	15289.48	297879
ENSMUSG00000064351.1	1545	1308.14	8040.905	133326.9

ENSMUSG00000022283.16	1515	1133.63	7840.946	112666.9
ENSMUSG00000118866.1	300	77.34	7165.066	7023.91
ENSMUSG00000038274.14	597	294.43	6893.805	25727.24
ENSMUSG00000057322.13	369	118.25	6493.216	9731.97

864691-3C

Name	Length	EffectiveLength	TPM	NumReads
ENSMUSG00002076161.1	331	93.65	33938.04	40292.17
ENSMUSG00000037742.15	876	640.49	29683.76	241032.9
ENSMUSG00000136525.1	259	60.54	23337.63	17913
ENSMUSG00000129312.1	302	78.25	21059.98	20891.75
ENSMUSG00000119584.1	1849	1537.19	15270.34	297594
ENSMUSG00000064351.1	1545	1308.06	8027.778	133128.2
ENSMUSG00000022283.16	1514	1132.56	7850.487	112721.5
ENSMUSG00000118866.1	300	77.28	7236.525	7090.4
ENSMUSG00000038274.14	598	294.59	6869.641	25656.43
ENSMUSG00000057322.13	369	118.16	6509.433	9750.97

864691-4A

Name	Length	EffectiveLength	TPM	NumReads
ENSMUSG00002076161.1	331	94.26	35700.59	52560.69
ENSMUSG00000129312.1	302	80.13	28980.45	36271.6
ENSMUSG00000037742.15	895	659.33	28933.08	297956.4
ENSMUSG00000118866.1	300	79.28	27347.05	33861.68
ENSMUSG00000065947.1	297	78.04	12569.57	15320.3
ENSMUSG00000064356.1	204	49	10186.04	7795
ENSMUSG00000064351.1	1545	1254.16	10119.26	198224.8
ENSMUSG00000136525.1	259	63.77	10092.28	10052
ENSMUSG00000094777.3	482	208.47	7666.246	24961.61
ENSMUSG00000057322.13	370	119.6	6980.899	13040.99

864691-4B

Name	Length	EffectiveLength	TPM	NumReads
ENSMUSG00002076161.1	331	94.3	35706.98	52578.75
ENSMUSG00000037742.15	896	659.96	28875.45	297571.5
ENSMUSG00000129312.1	302	80.04	28836.6	36040.62
ENSMUSG00000118866.1	300	79.17	27579.42	34095.39
ENSMUSG00000065947.1	297	77.96	12377.62	15066.88
ENSMUSG00000064351.1	1545	1253.74	10119.17	198104.1
ENSMUSG00000136525.1	259	63.72	10047.02	9997
ENSMUSG00000064356.1	204	49.03	10046.04	7692

ENSMUSG00000094777.3	482	208.43	7643.466	24877.12
ENSMUSG00000057322.13	370	119.68	6931.218	12952.99

864691-4C

Name	Length	EffectiveLength	TPM	NumReads
ENSMUSG00002076161.1	331	94.33	35665.64	52550.71
ENSMUSG00000129312.1	302	80.1	30138.69	37706.78
ENSMUSG00000037742.15	894	658.61	28931.69	297617.7
ENSMUSG00000118866.1	300	79.23	26355.64	32615.47
ENSMUSG00000065947.1	297	77.99	12541.53	15277.92
ENSMUSG00000064351.1	1545	1254.04	10122.82	198276.4
ENSMUSG00000136525.1	259	63.79	10084.3	10047
ENSMUSG00000064356.1	204	49.02	10053.13	7697
ENSMUSG00000094777.3	482	208.43	7595.325	24726.77
ENSMUSG00000057322.13	370	119.75	7004.452	13100.99

864691-5A

Name	Length	EffectiveLength	TPM	NumReads
ENSMUSG00002076161.1	331	120.65	141083	339955.2
ENSMUSG00000118841.1	300	95.82	57410.63	109872.9
ENSMUSG00000118866.1	300	95.82	48647.36	93101.66
ENSMUSG00000064356.1	204	30.76	31664.07	19454
ENSMUSG00000092837.3	319	111.05	22253.7	49359
ENSMUSG00000088088.2	271	75	15604.61	23374.79
ENSMUSG00000064351.1	1545	1261.25	14576.61	367191.6
ENSMUSG00000064357.1	681	427.21	12804.1	109252.4
ENSMUSG00000064341.1	957	691.97	9411.26	130068
ENSMUSG00000064354.1	684	435.46	9334.608	81185.99

864691-5B

Name	Length	EffectiveLength	TPM	NumReads
ENSMUSG00002076161.1	331	120.66	141527.1	340752.7
ENSMUSG00000118841.1	300	95.92	57053.44	109198.8
ENSMUSG00000118866.1	300	95.92	48987.06	93759.99
ENSMUSG00000064356.1	204	30.82	31445.3	19340
ENSMUSG00000092837.3	319	111.2	22233.97	49333
ENSMUSG00000088088.2	271	75.16	15468.98	23199.52
ENSMUSG00000064351.1	1545	1260.9	14581.18	366861.3
ENSMUSG00000064357.1	681	427.42	12796.48	109138.3
ENSMUSG00000064341.1	957	692.11	9429.087	130218
ENSMUSG00000064354.1	684	435.51	9365.673	81388.28

864691-5C

Name	Length	EffectiveLength	TPM	NumReads
ENSMUSG00002076161.1	331	120.65	140995.2	339878.1
ENSMUSG00000118841.1	300	95.88	56573.78	108372
ENSMUSG00000118866.1	300	95.88	49120.44	94094.45
ENSMUSG00000064356.1	204	30.76	31801.82	19544.71
ENSMUSG00000092837.3	319	111.26	22182.73	49309
ENSMUSG00000088088.2	271	75.13	15531.99	23314.58
ENSMUSG00000064351.1	1545	1261.03	14538.15	366283.7
ENSMUSG00000064357.1	681	427.18	12771.77	109003.8
ENSMUSG00000064341.1	957	691.84	9412.918	130110
ENSMUSG00000064354.1	684	435.41	9363.403	81454.78

Table 6: Differential Expression Metrics

Sample Pair Comparison	Total genes in annotation	Genes expressed and tested	Differentially expressed genes	Total transcripts in annotation	Transcripts expressed and tested	Differentially expressed transcripts
**864691-2** Vs 864691-1	78,258	15,716	10,306	278,299	37,029	27,720
**864691-3** Vs 864691-1		15,891	11,675		37,386	30,201
**864691-4** Vs 864691-1		15,611	9,462		36,606	25,086
**864691-5** Vs 864691-1		14,826	11,948		38,195	30,080

Sample Pair Comparison	Differential Regulation Gene Count			
	Total Change	Up Regulated	Down Regulated	No Change
**864691-2** Vs 864691-1	15,715	2,091	1,003	12,621
**864691-3** Vs 864691-1	15,891	2,669	1,348	11,874
**864691-4** Vs 864691-1	15,611	1,628	846	13,137
**864691-5** Vs 864691-1	14,826	2,522	2,717	9,587

Table 7: Differential Gene Expression – Pairwise Sample Comparison

864691-2 Vs 864691-1

Most Significant UP Regulated Genes

Gene ID	Gene Name	Gene Description	log2 FoldChange	P Value adjusted
ENSMUSG00000093485.2	Gm20708	predicted gene 20708	10.34611	2.92E-17
ENSMUSG00000119761.1	Snord3b2	small nucleolar RNA, C/D box 3B2	9.144879	1.79E-13
ENSMUSG00000093954.9	Gm16867	predicted gene, 16867	9.04874	3.49E-13
ENSMUSG00000097795.2	Gm7678	predicted gene 7678	8.949355	6.90E-13
ENSMUSG00000112693.3	Gm5512	predicted gene 5512	8.750059	2.68E-12
ENSMUSG00000121597.1	ENSMUSG00000121597	novel KRAB containing domain zinc finger protein	8.688751	4.30E-12
ENSMUSG00000104161.2	Gm20089	predicted gene, 20089	8.646081	6.49E-12
ENSMUSG00000092278.2	Gm8752	predicted pseudogene 8752	8.494992	1.48E-11
ENSMUSG00000120217.2	Gm57197	predicted gene, 57197	8.446328	2.05E-11
ENSMUSG00000086199.9	Bcas3os1	BCAS3 microtubule associated cell migration factor, opposite strand 1	8.405841	2.74E-11

Most Significant DOWN Regulated Genes

Gene ID	Gene Name	Gene Description	log2 FoldChange	P Value adjusted
ENSMUSG00000117694.2	Snhg4	small nucleolar RNA host gene 4	-11.051	1.15E-19
ENSMUSG00000100679.3	Gm28778	predicted gene 28778	-10.0997	1.73E-16
ENSMUSG00000116016.2	Gm49496	predicted gene, 49496	-9.84963	1.13E-15

ENSMUSG00000030680.7	Pagr1a	PAXIP1 associated glutamate rich protein 1A	-9.58783	6.61E-13
ENSMUSG00000083405.2	Gm15725	predicted gene 15725	-9.41041	2.80E-14
ENSMUSG00000116875.2	Morf4l1-ps1	mortality factor 4 like 1, pseudogene 1	-8.66869	4.25E-12
ENSMUSG00000049734.10	Trex1	three prime repair exonuclease 1	-8.62112	5.55E-12
ENSMUSG00000138882.1	ENSMUSG00000138882	predicted gene, 63396	-8.60301	6.63E-12
ENSMUSG00000092232.2	Gm20521	predicted gene 20521	-8.57202	7.71E-12
ENSMUSG00000091277.3	Gm1818	predicted gene 1818	-8.3202	3.97E-11

864691-3 Vs 864691-1

Most Significant UP Regulated Genes

Gene ID	Gene Name	Gene Description	log2 FoldChange	P Value adjusted
ENSMUSG00000099041.2	Gm28035	predicted gene, 28035	10.3767	3.07E-15
ENSMUSG00000082082.2	Gm13230	predicted gene 13230	10.0401	1.94E-16
ENSMUSG00000098573.2	Gm27232	predicted gene 27232	9.879896	5.69E-16
ENSMUSG00000115232.2	Gm49378	predicted gene, 49378	9.485621	1.03E-14
ENSMUSG00000116069.2	Gm49510	predicted gene, 49510	9.431032	1.53E-14
ENSMUSG00000094475.9	Gm11007	predicted gene 11007	8.797075	1.57E-12
ENSMUSG00000134054.1	ENSMUSG00000134054	predicted gene, 41285	8.776292	1.56E-12
ENSMUSG00000085376.2	Gm14508	predicted gene 14508	8.73484	2.08E-12
ENSMUSG00000048458.9	Inka2	inka box actin regulator 2	8.649101	3.77E-12
ENSMUSG00000123148.1	ENSMUSG00000123148	predicted gene, 74587	8.641762	3.97E-12

Most Significant DOWN Regulated Genes

Gene ID	Gene Name	Gene Description	log2 FoldChange	P Value adjusted
ENSMUSG00000107478.2	Gm45234	predicted gene 45234	-11.9033	5.57E-23
ENSMUSG00000100679.3	Gm28778	predicted gene 28778	-10.0604	1.33E-16
ENSMUSG00000144222.1	Gm57848	predicted gene, 57848	-9.68665	2.32E-15
ENSMUSG00000030680.7	Pagr1a	PAXIP1 associated glutamate rich protein 1A	-9.54827	5.19E-13

ENSMUSG00000037845.15	Fdxacb1	ferredoxin-fold anticodon binding domain containing 1	-9.10756	1.32E-13
ENSMUSG00000120920.1	Gm56812	predicted gene, 56812	-8.89046	5.90E-13
ENSMUSG00000111692.2	Gm49373	predicted gene, 49373	-8.88566	6.59E-13
ENSMUSG00000089739.3	Gm20431	predicted gene 20431	-8.83035	1.93E-12
ENSMUSG00000085742.4	Gm13536	predicted gene 13536	-8.67539	2.56E-12
ENSMUSG00000117809.2	Asdurf	Asnsd1 upstream reading frame	-8.64101	3.29E-12

864691-4 Vs 864691-1

Most Significant UP Regulated Genes

Gene ID	Gene Name	Gene Description	log2 FoldChange	P Value adjusted
ENSMUSG00000115232.2	Gm49378	predicted gene, 49378	10.02697	5.06E-16
ENSMUSG00000093485.2	Gm20708	predicted gene 20708	9.514781	1.82E-14
ENSMUSG00000099041.2	Gm28035	predicted gene, 28035	9.307289	1.01E-13
ENSMUSG00000131993.1	ENSMUSG00000131993	predicted gene, 75078	8.854418	1.73E-12
ENSMUSG00000136522.1	ENSMUSG00000136522	predicted gene, 38868	8.759692	3.17E-12
ENSMUSG00000119427.1	Gm24830	predicted gene, 24830	8.739936	3.60E-12
ENSMUSG00000119774.1	Gm57479	predicted gene, 57479	8.720066	4.15E-12
ENSMUSG00000065087.3	Snord22	small nucleolar RNA, C/D box 22	8.622762	7.69E-12
ENSMUSG00000097998.2	Gm27019	predicted gene, 27019	8.422934	2.78E-11
ENSMUSG00000102222.2	Pcdhga10	protocadherin gamma subfamily A, 10	8.381167	3.64E-11

Most Significant DOWN Regulated Genes

Gene ID	Gene Name	Gene Description	log2 FoldChange	P Value adjusted
ENSMUSG00000072621.15	Slfn10-ps	schlafen 10	-10.4186	2.49E-17
ENSMUSG00000089739.3	Gm20431	predicted gene 20431	-8.89345	3.07E-12
ENSMUSG00000117809.2	Asdurf	Asnsd1 upstream reading frame	-8.70364	4.15E-12

ENSMUSG00000138882.1	ENSMUSG00000138882	predicted gene, 63396	-8.62633	7.41E-12
ENSMUSG00000092232.2	Gm20521	predicted gene 20521	-8.59544	8.40E-12
ENSMUSG00000121418.1	ENSMUSG00000121418	predicted gene, 58357	-8.20314	9.95E-11
ENSMUSG00000072647.7	Adam1a	a disintegrin and metallopeptidase domain 1a	-8.05658	2.51E-10
ENSMUSG00000083500.2	Gm15470	predicted gene 15470	-8.03826	2.86E-10
ENSMUSG00000127218.1	ENSMUSG00000127218	predicted gene, 72463	-7.69793	2.33E-09
ENSMUSG00000125995.1	ENSMUSG00000125995	predicted gene, 74153	-7.66253	2.86E-09

864691-5 Vs 864691-1

Most Significant UP Regulated Genes

Gene ID	Gene Name	Gene Description	log2 FoldChange	P Value adjusted
ENSMUSG00000088088.2	Rmrp	RNA component of mitochondrial RNAase P	17.20062	1.38E-47
ENSMUSG00000093769.5	H3c14	H3 clustered histone 14	14.48934	3.91E-34
ENSMUSG00000091957.6	Rps2-ps10	ribosomal protein S2, pseudogene 10	14.35674	1.65E-33
ENSMUSG00000115232.2	Gm49378	predicted gene, 49378	12.31297	6.09E-25
ENSMUSG00000119262.1	Snord3b1	small nucleolar RNA, C/D box 3B1	12.06481	3.31E-23
ENSMUSG00000119851.1	Snord3b4	small nucleolar RNA, C/D box 3B4	11.73456	9.97E-23
ENSMUSG00000119761.1	Snord3b2	small nucleolar RNA, C/D box 3B2	11.54514	6.00E-22
ENSMUSG00000119648.1	Snord3b3	small nucleolar RNA, C/D box 3B3	10.75194	3.45E-17
ENSMUSG00000140475.1	Gm62098	predicted gene, 62098	10.14122	3.84E-17
ENSMUSG00000120367.2	Gm57294	predicted gene, 57294	10.08515	5.90E-17

Most Significant DOWN Regulated Genes

Gene ID	Gene Name	Gene Description	log2 FoldChange	P Value adjusted
ENSMUSG00000086583.6	Gm15500	predicted pseudogene 15500	-13.5179	7.22E-30
ENSMUSG00000124476.1	Gm59808	predicted gene, 59808	-12.8051	6.51E-27
ENSMUSG00000130597.1	Gm59785	predicted gene, 59785	-12.0058	2.51E-61

ENSMUSG00000107478.2	Gm45234	predicted gene, 45234	-11.83	4.81E-23
ENSMUSG00000121570.1	Gm58608	predicted gene, 58608	-11.5566	4.30E-22
ENSMUSG00000129906.1	ENSMUSG00000129906	predicted gene, 63993	-11.5464	4.57E-22
ENSMUSG00000108815.2	Gm49388	predicted gene, 49388	-11.5183	5.85E-22
ENSMUSG00000100153.4	Ppp1ccb	protein phosphatase 1 catalytic subunit gamma B	-11.2765	4.39E-21
ENSMUSG00000080542.3	Rnu4atac	RNA, U4atac small nuclear (U12-dependent splicing)	-10.9497	6.29E-20
ENSMUSG00000114117.2	Gm48795	predicted gene, 48795	-10.9386	6.86E-20

Figure 1: Differentially expressed genes Heatmap

864691-2 Vs 864691-1

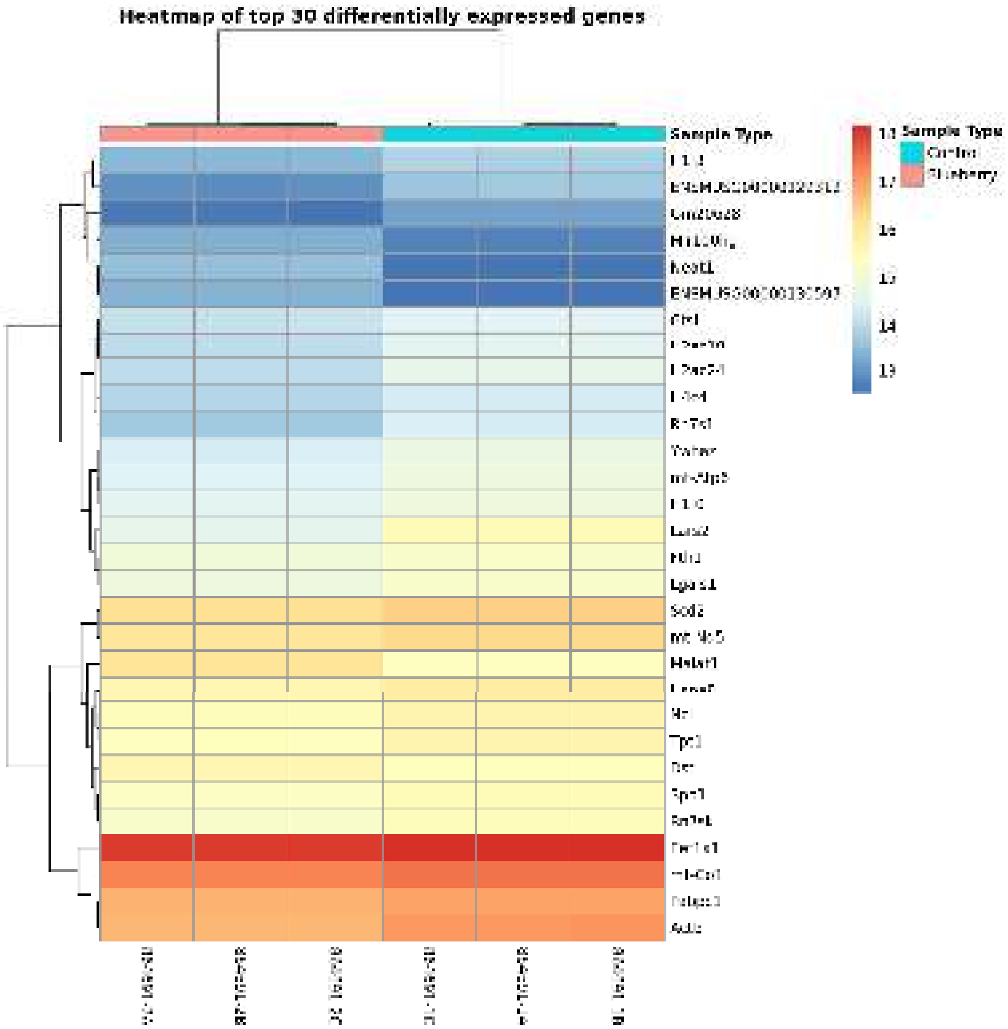

864691-3 Vs 864691-1

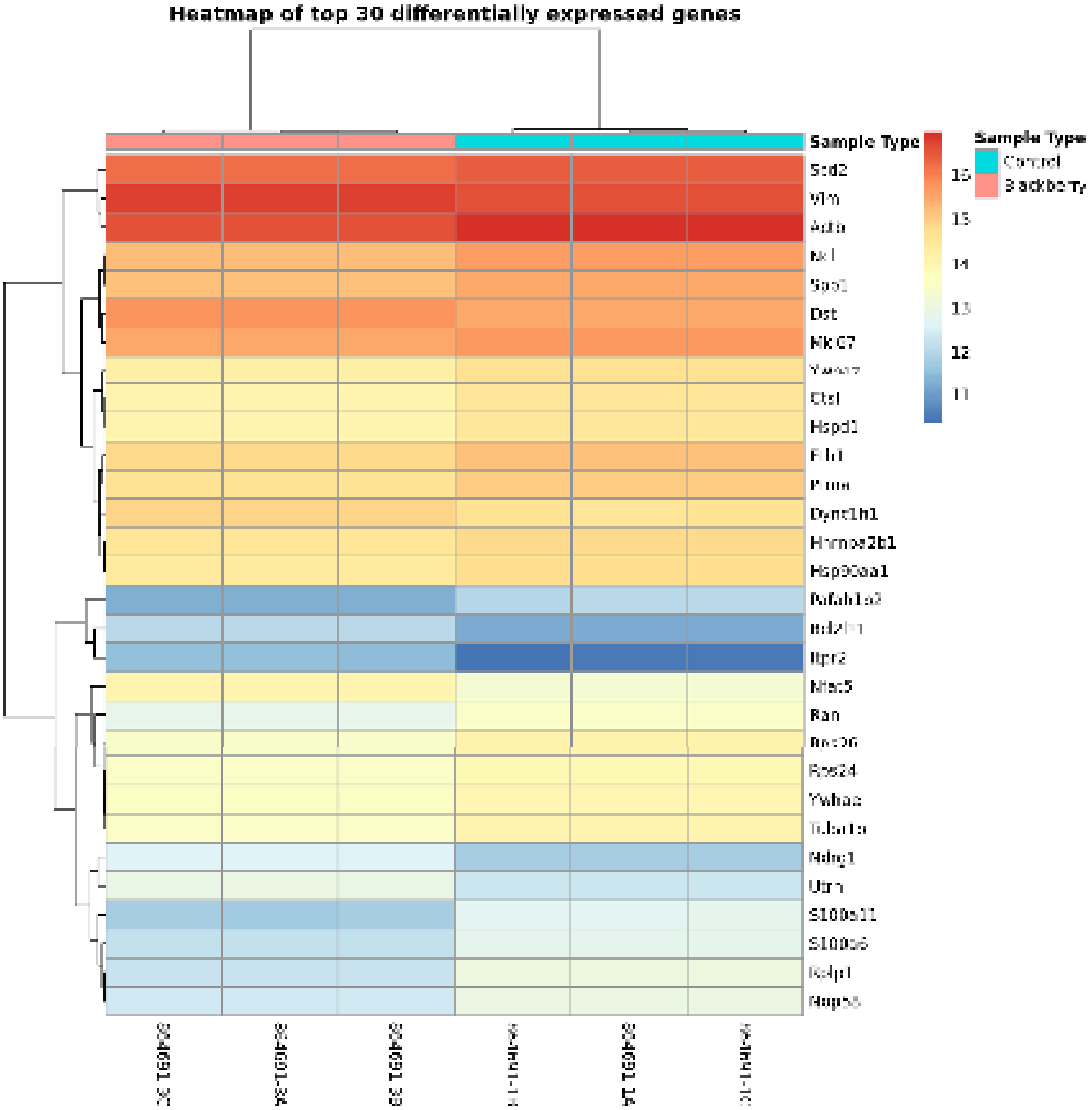

864691-4 Vs 864691-1

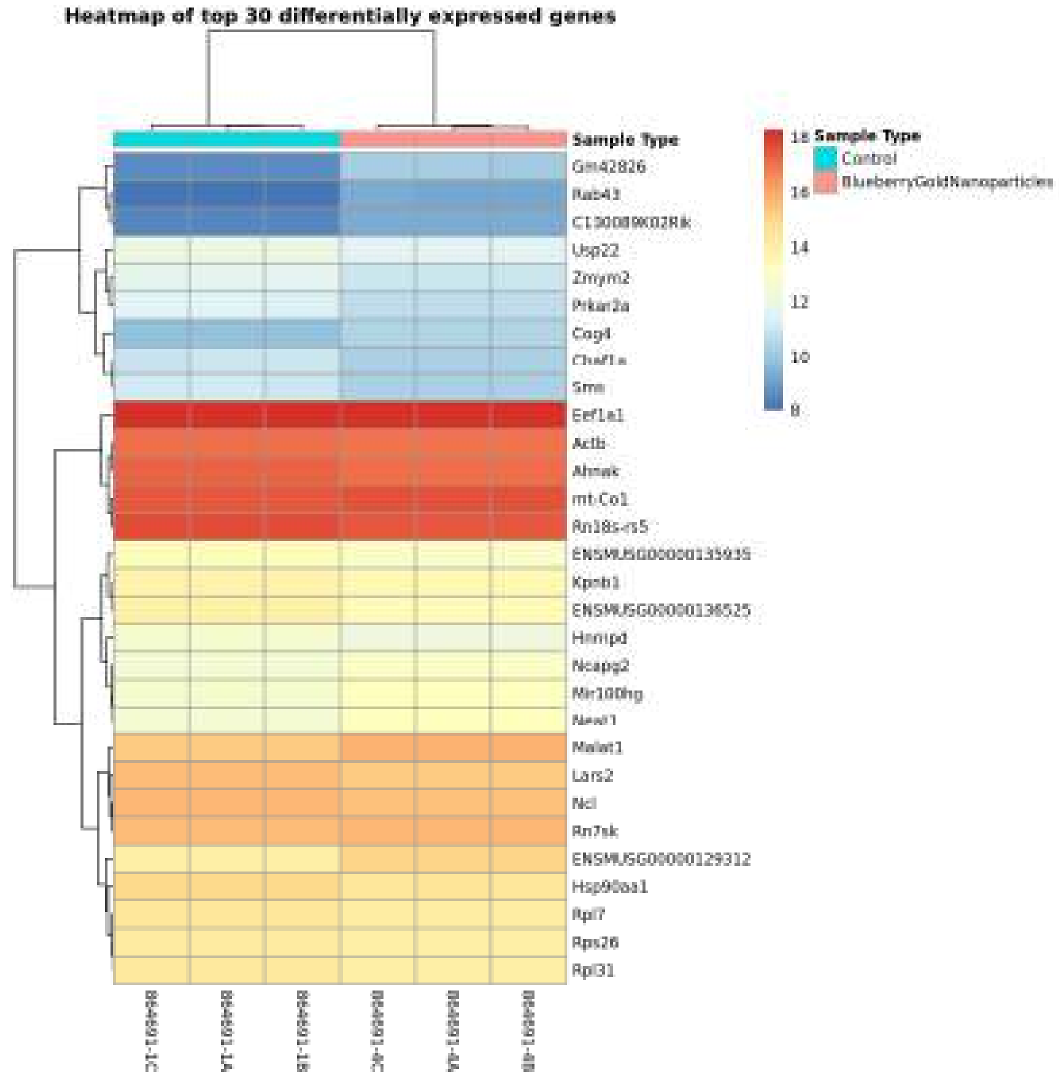

864691-5 Vs 864691-1

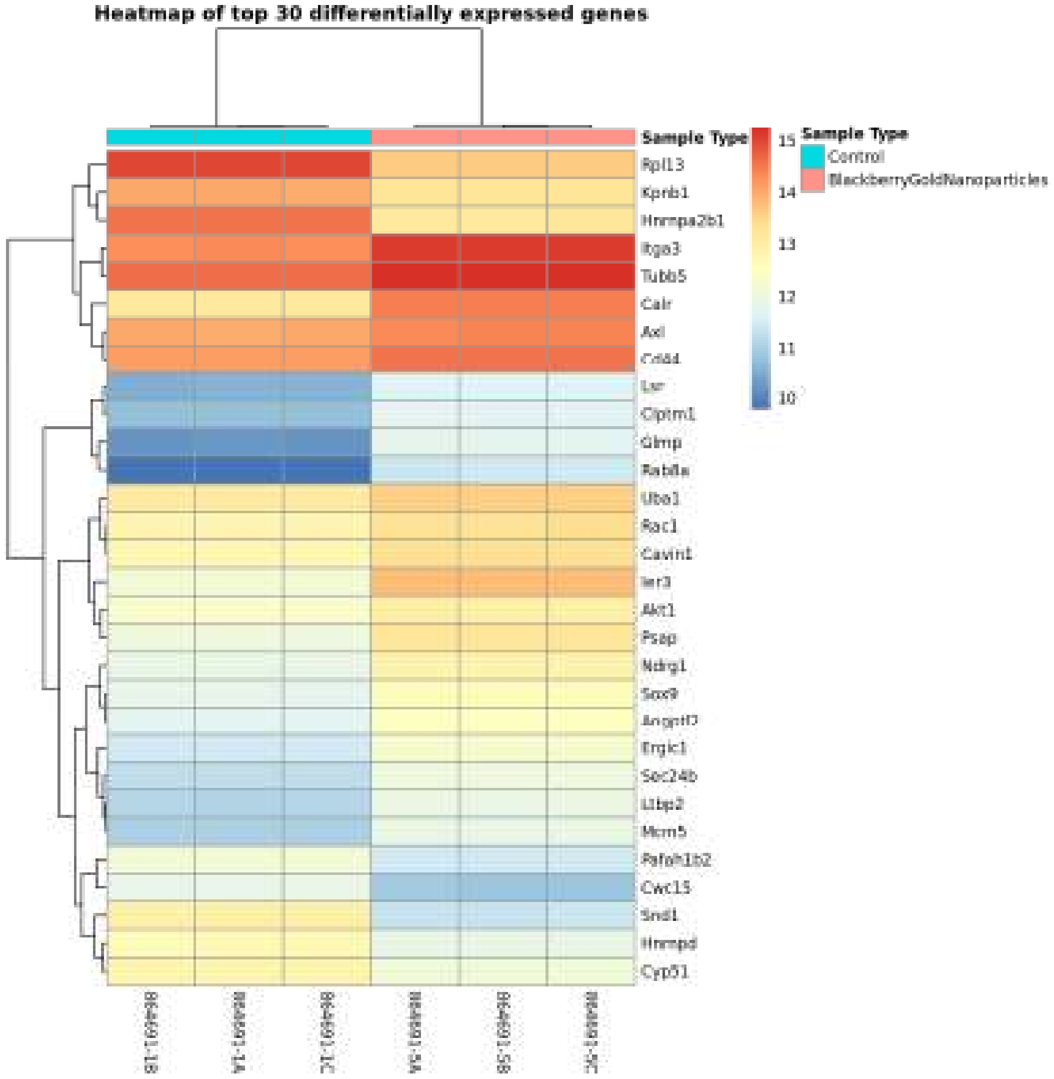

Figure 2: MA plot

864691-2 Vs 864691-1

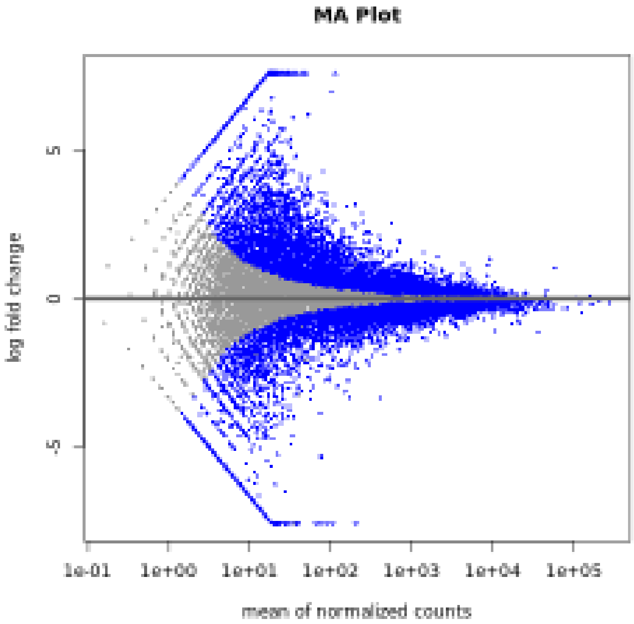

864691-3 Vs 864691-1

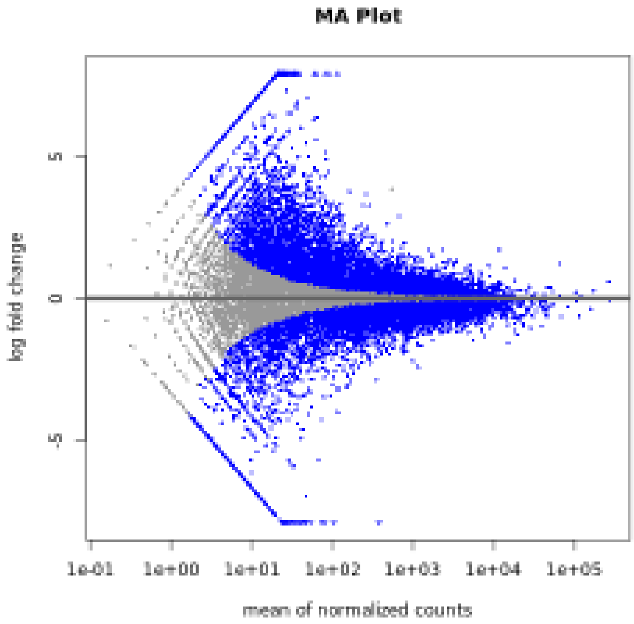

864691-4 Vs 864691-1

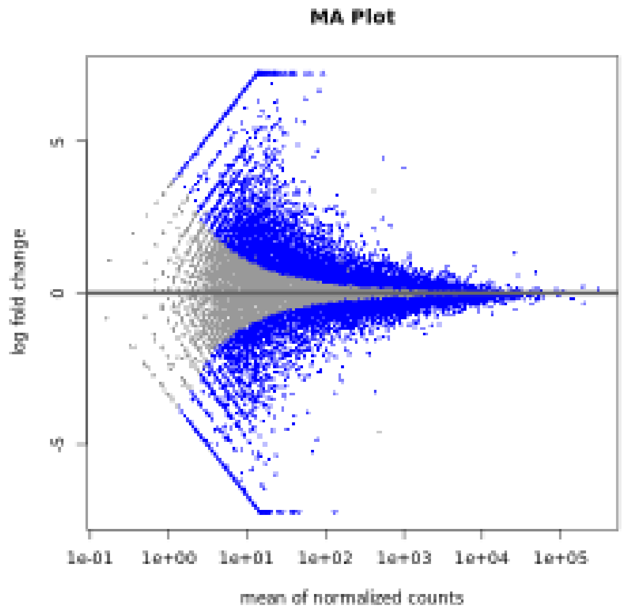

864691-5 Vs 864691-1

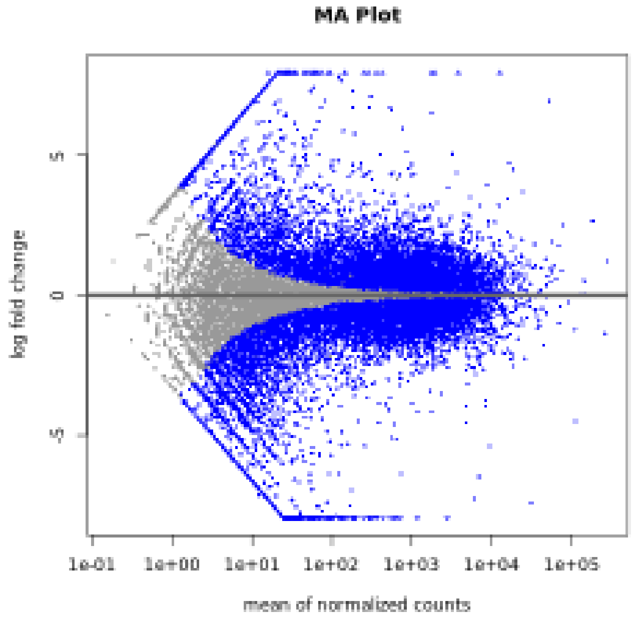

Figure 3: PCA plot

864691-2 Vs 864691-1

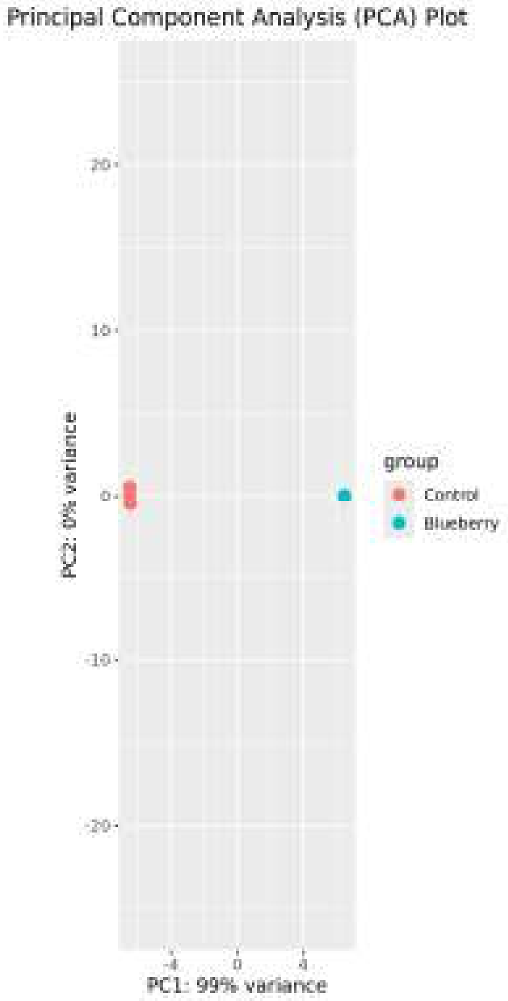

864691-3 Vs 864691-1

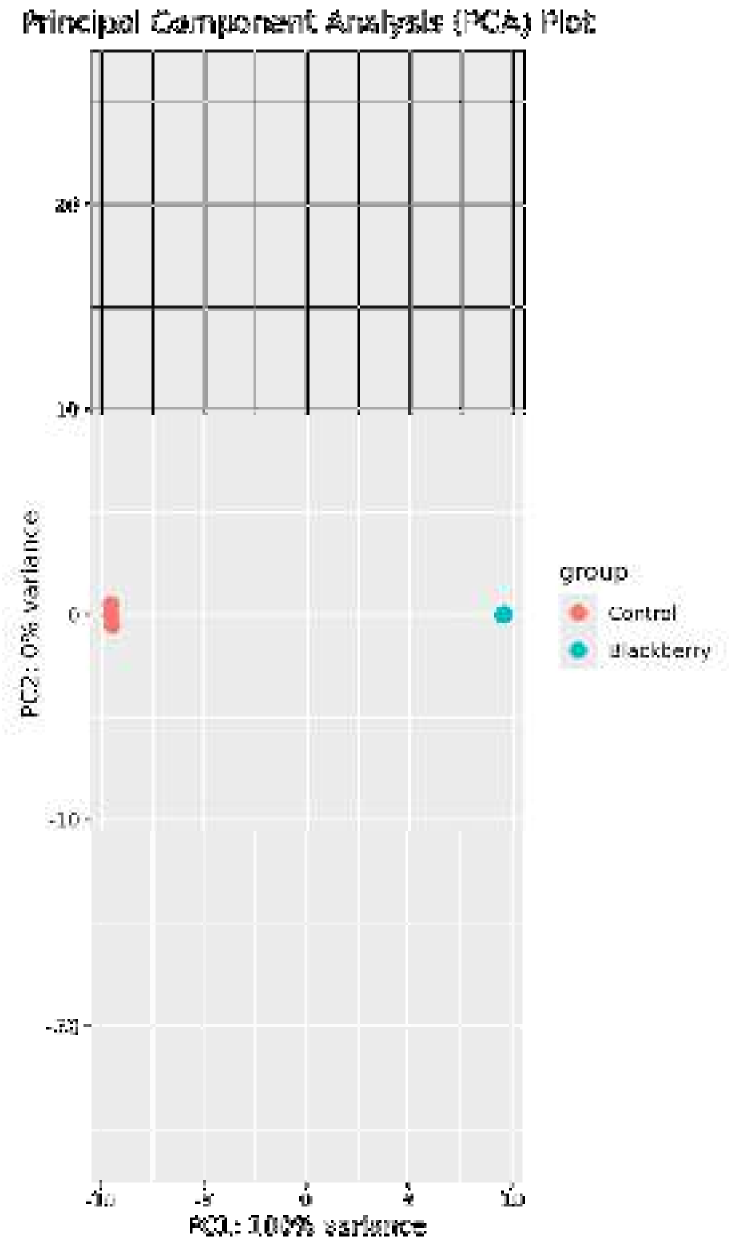

864691-4 Vs 864691-1

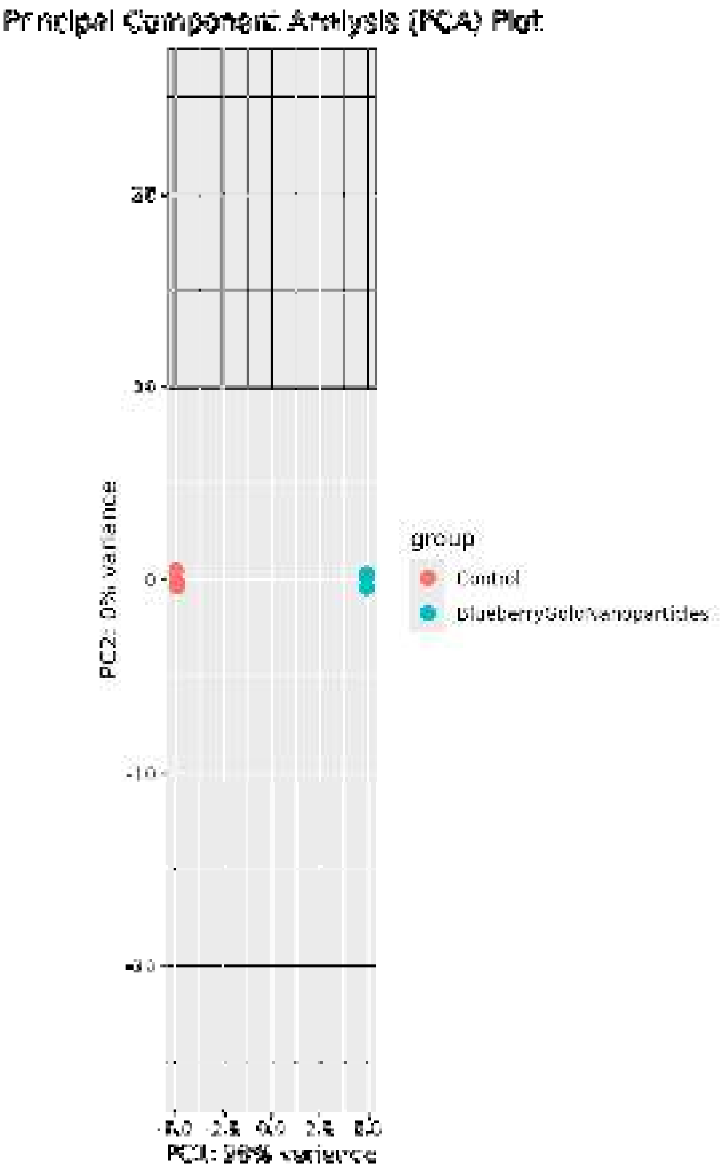

864691-5 Vs 864691-1

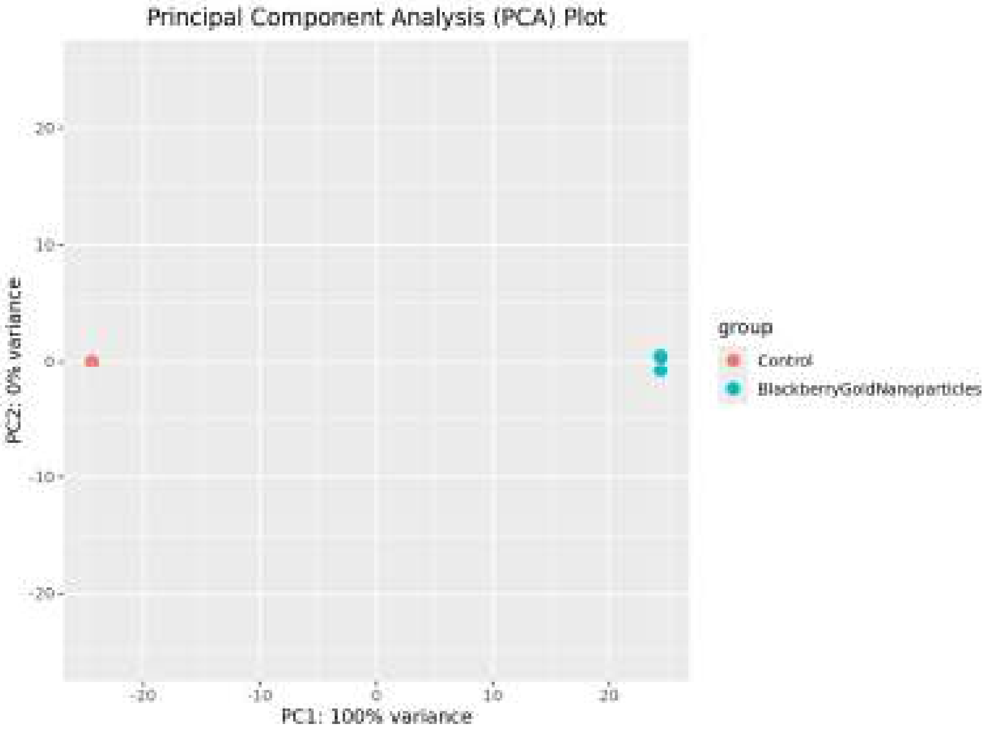

Figure 4: Gene Expression Levels Across Samples

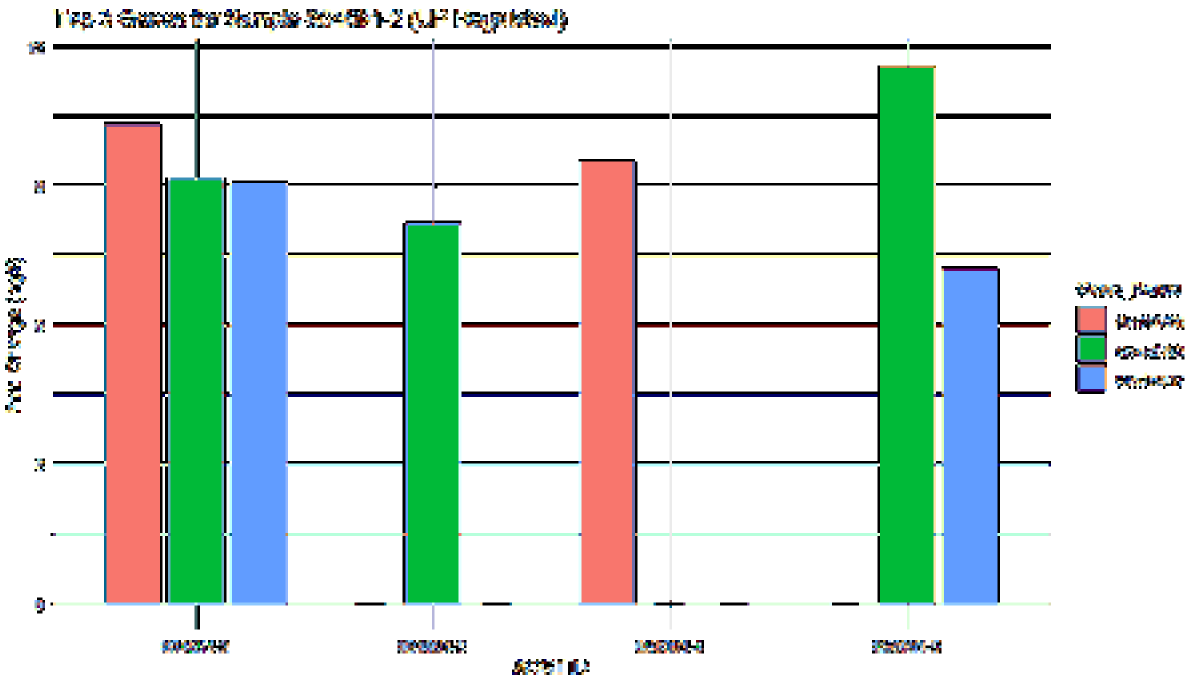

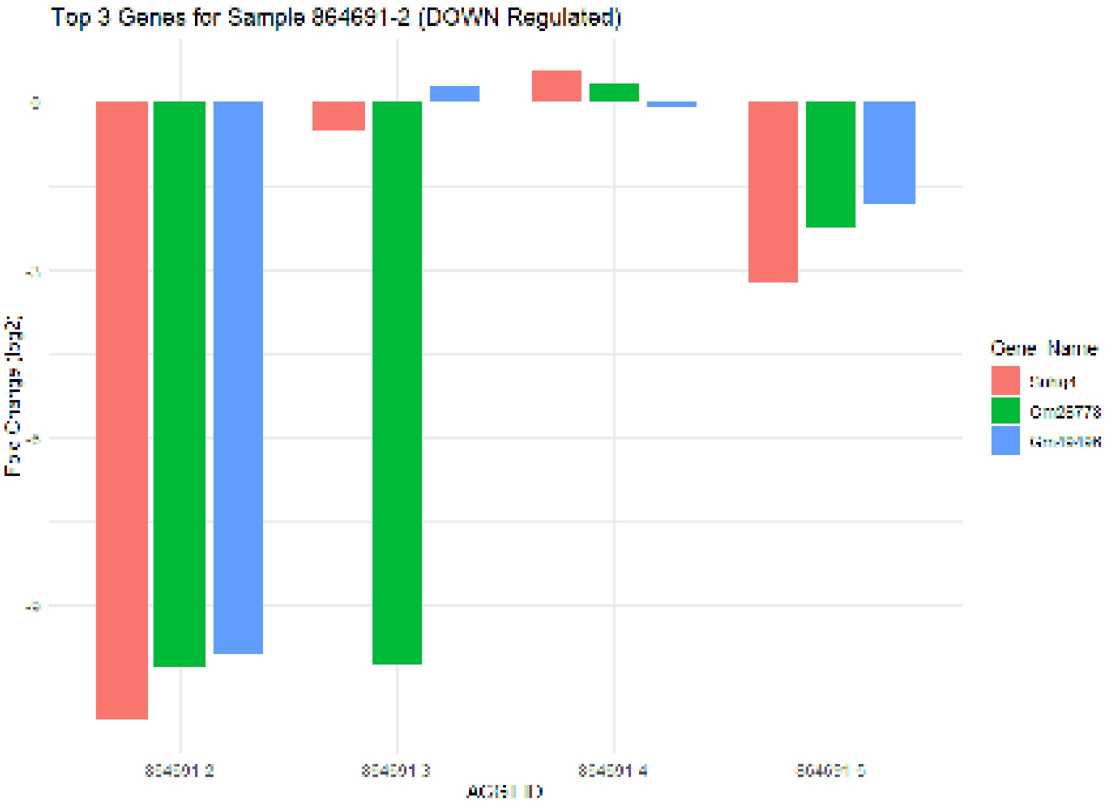
Top 3 Genes for Sample 864691-2 (DOWN Regulated)
Gene Name

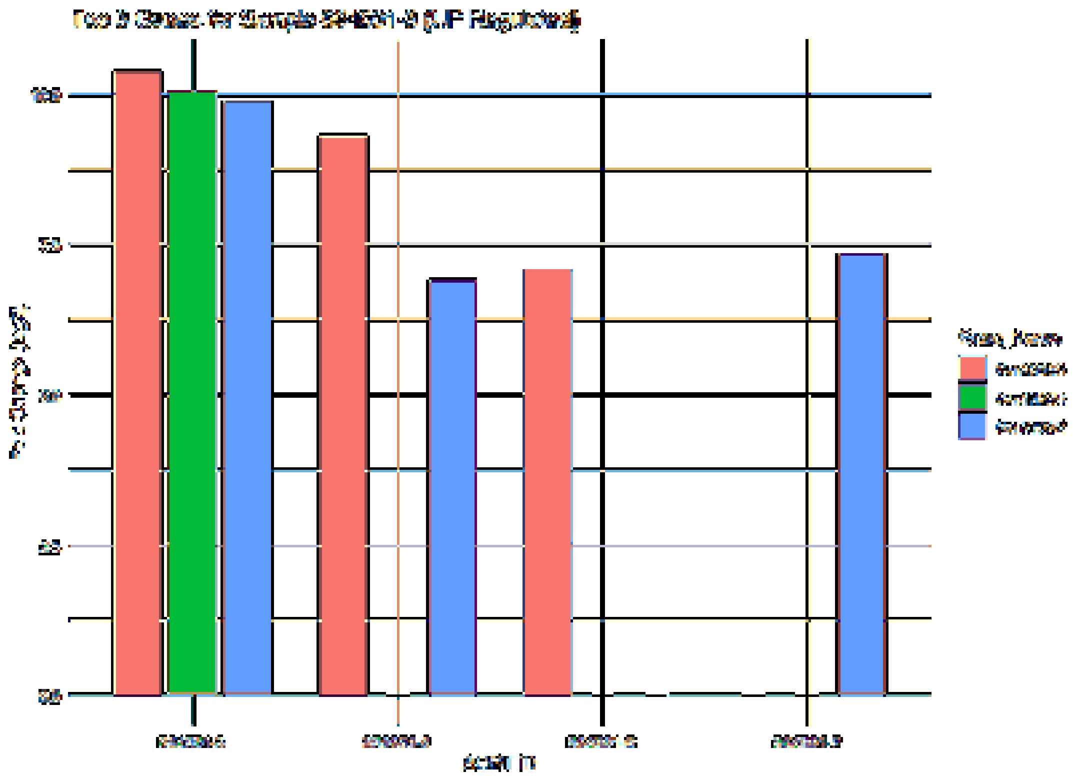

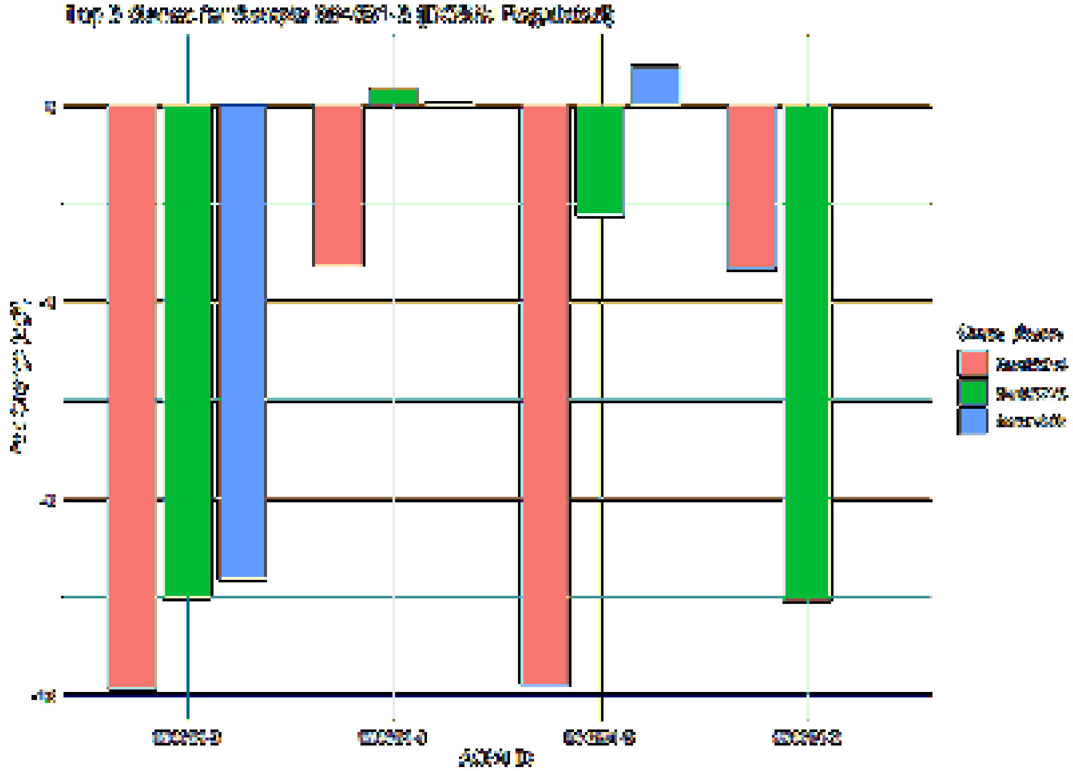

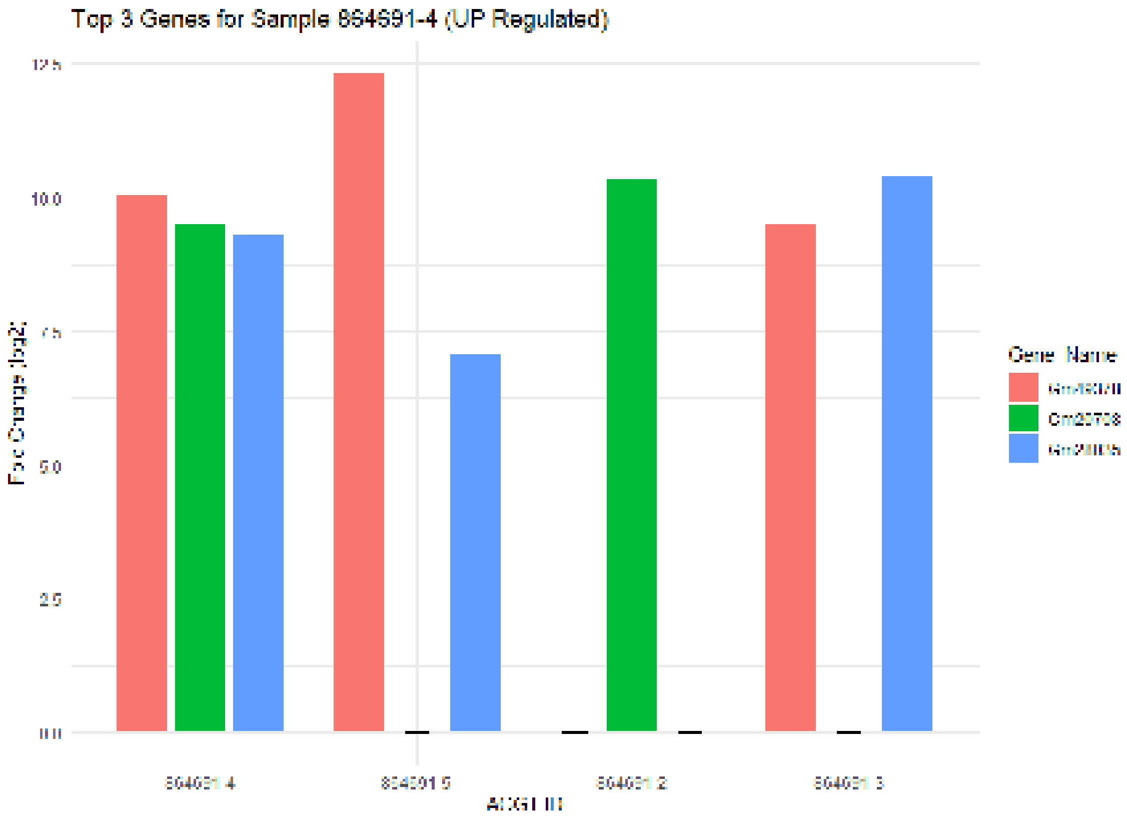
Top 3 Genes for Sample 864691-4 (UP Regulated)
12.5
10.0
7.5
5.0
2.5
0.0
Gene_Name
864691-4
864691-5
864691-2
864691-3

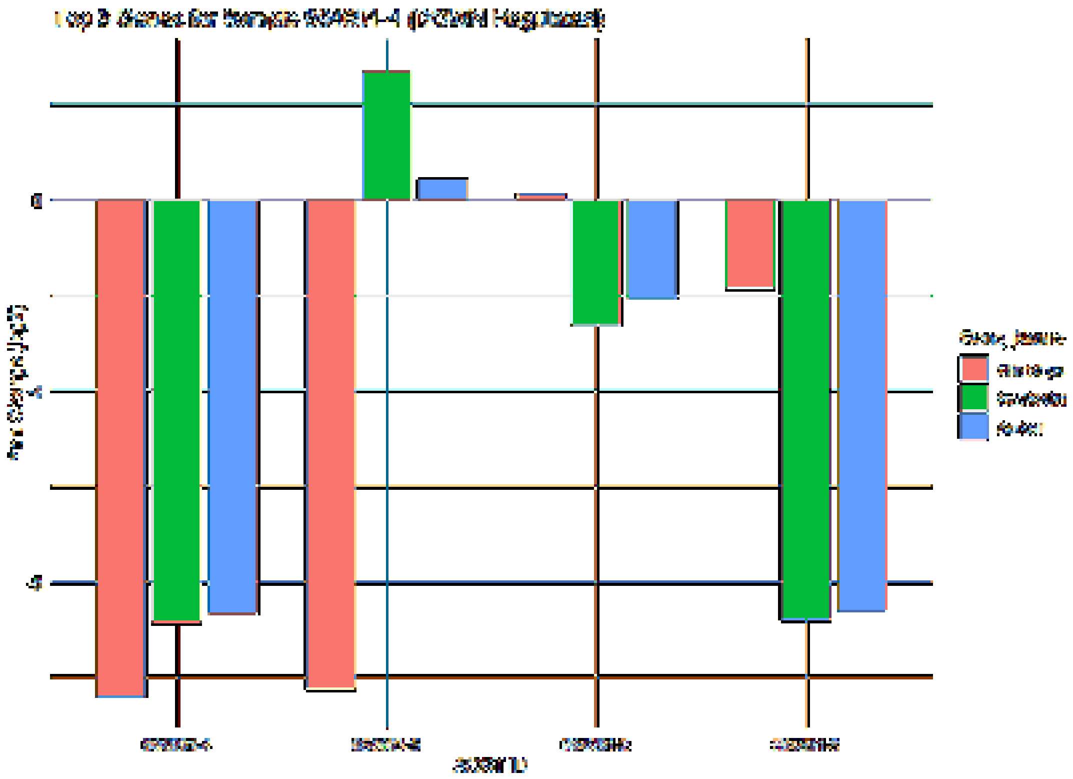
0

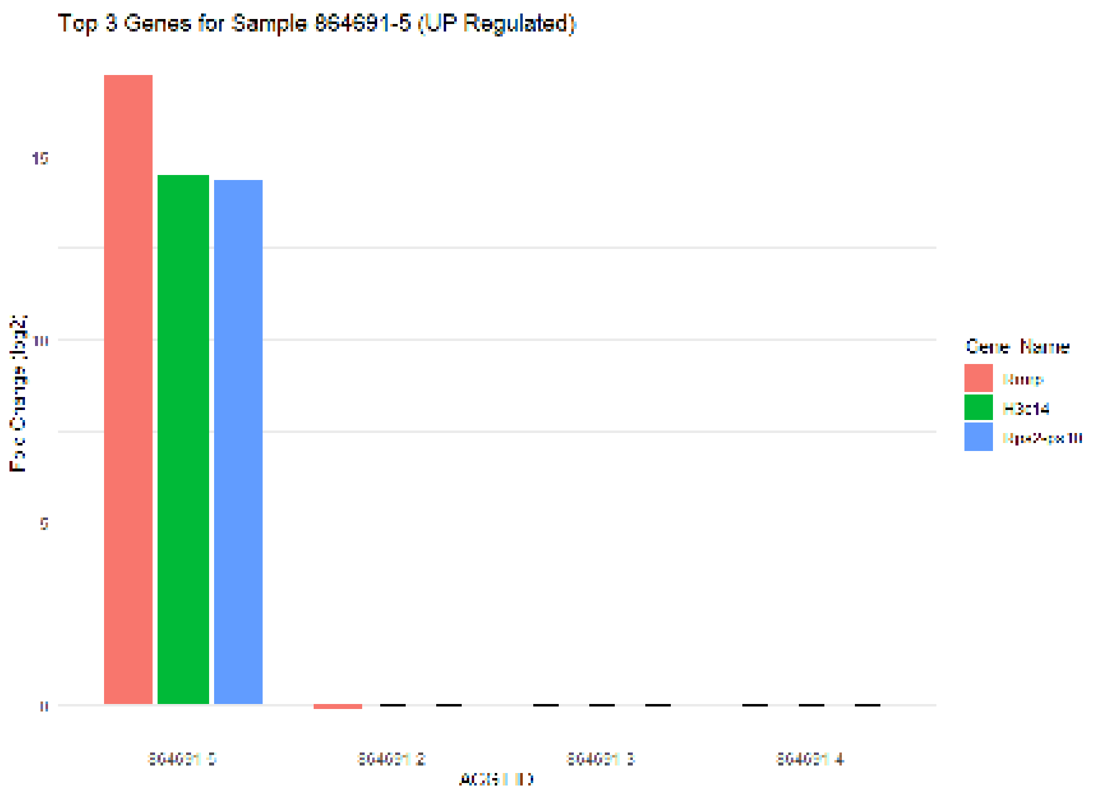
Top 3 Genes for Sample 864691-5 (UP Regulated)
15
10
5
0
Gene Name
H3c14

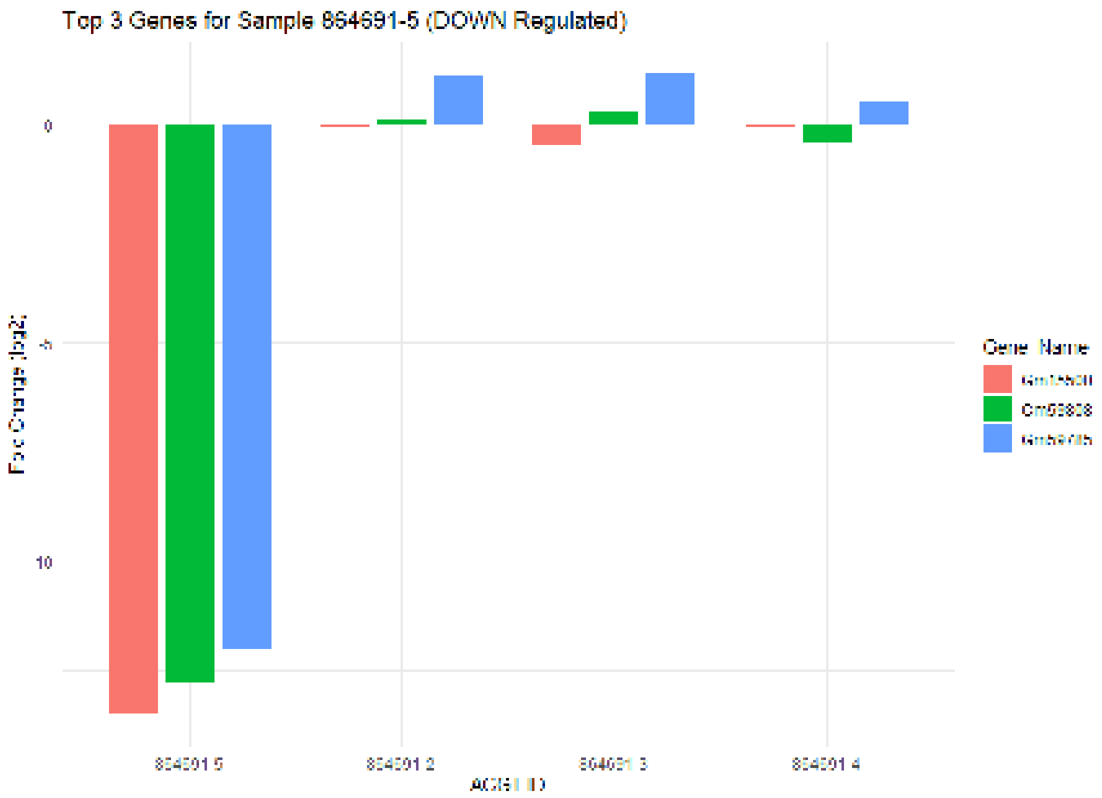
Top 3 Genes for Sample 864691-5 (DOWN Regulated)
0
-5
-10
Gene Name

Details

GO:0007156
Name
homophilic cell adhesion via plasma membrane adhesion molecules
Definition
The attachment of a plasma membrane adhesion molecule in one cell to an identical molecule in an adjacent cell.

Statistics

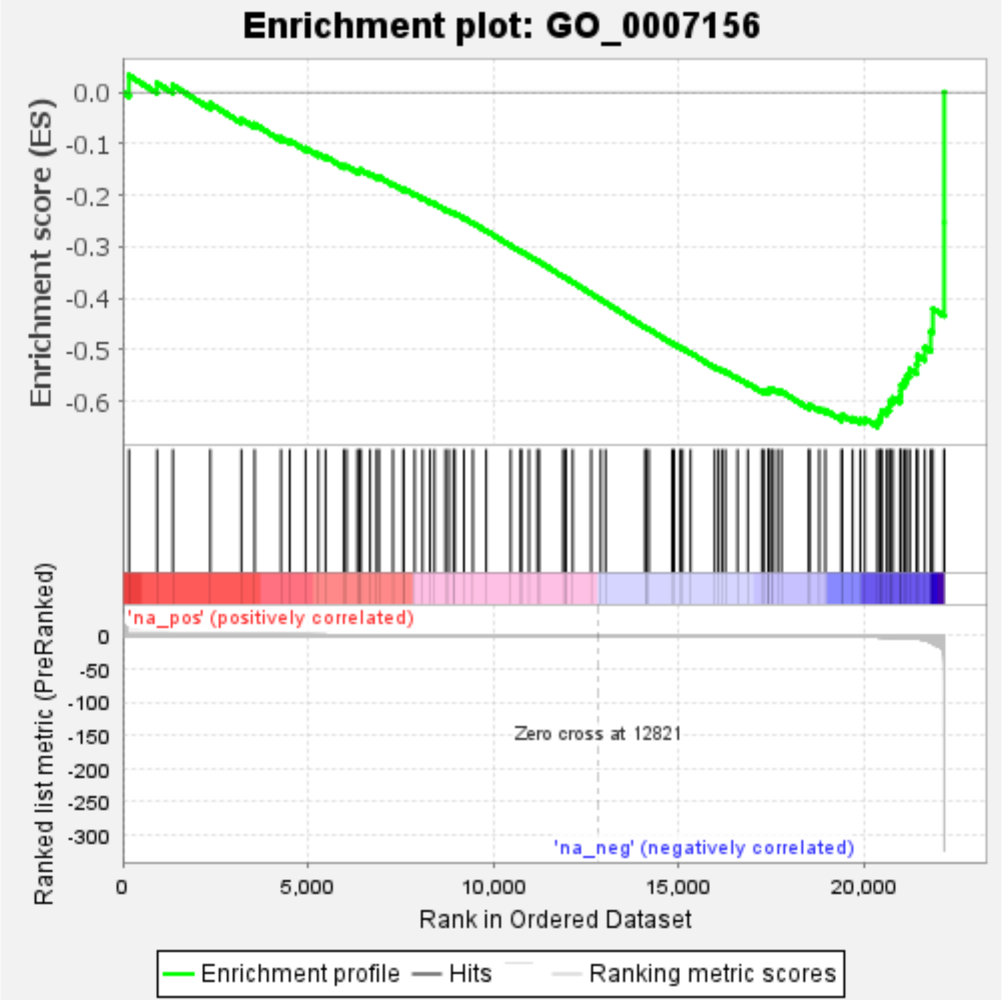
Enrichment plot: GO_0007156
Enrichment score (ES)
0.0
-0.1
-0.2
-0.3
-0.4
-0.5
-0.6
'na_pos' (positively correlated)
Ranked list metric (PreRanked)
0
-50
-100
-150
-200
-250
-300
Zero cross at 12821
'na_neg' (negatively correlated)
0
5,000
10,000
15,000
20,000
Rank in Ordered Dataset
Enrichment profile
Hits
Ranking metric scores

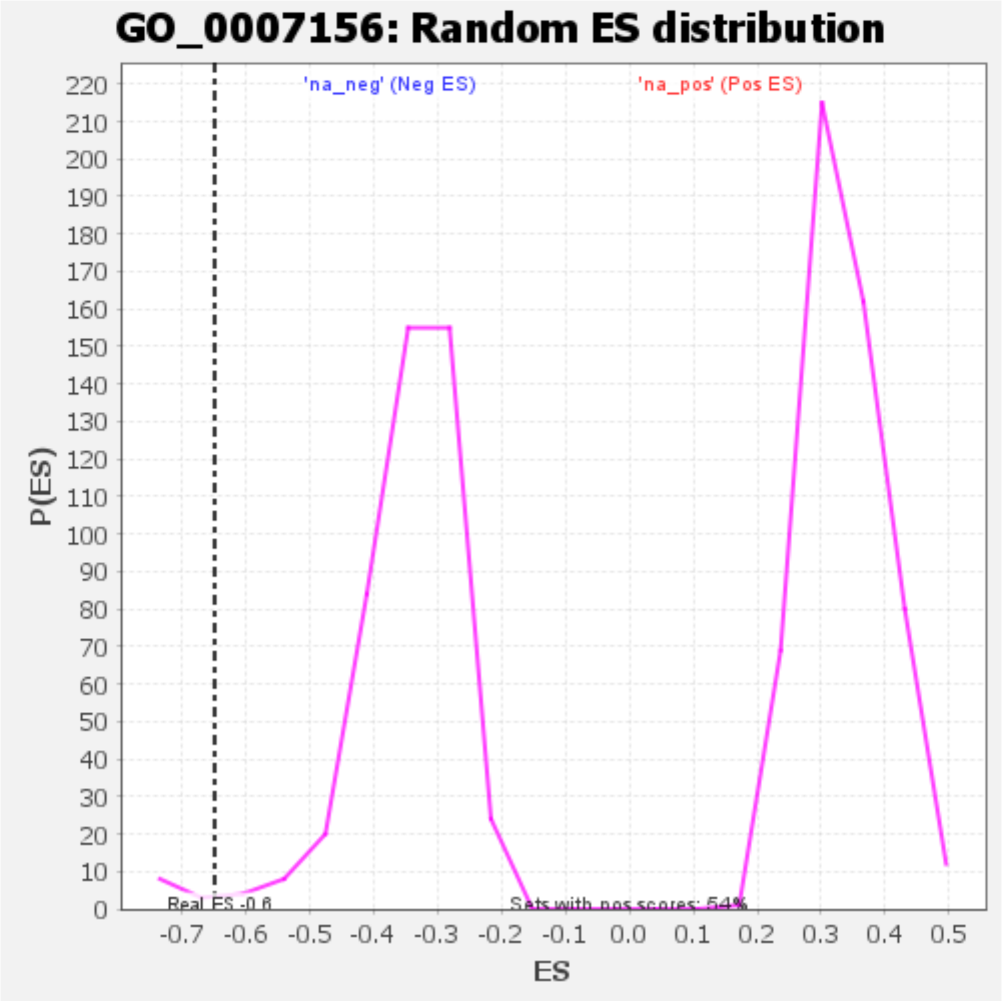

GSEA Details

Probe	Rank in Gene List	Rank Metric Score	Running ES	Core Enrichment
Kirrel3	173	10.123294	0.032587	No
Pik3cb	930	4.85911	0.017723	No
Cdh23	1357	3.819003	0.013664	No
Celsr3	2365	2.514167	-0.021943	No
Robo1	3203	1.895866	-0.052313	No
Prtg	3559	1.692483	-0.061646	No
Pvr	4271	1.404989	-0.088266	No
Cadm4	4506	1.316666	-0.093615	No
Nectin1	4935	1.170863	-0.10834	No
Nptn	5278	1.049304	-0.119653	No
Nectin3	5473	0.998085	-0.124461	No
Cdh24	5962	0.878703	-0.143073	No
Cdhr2	6040	0.865049	-0.143109	No
Cdh17	6328	0.829732	-0.152805	No
Cdh19	6391	0.816811	-0.152353	No
Cdh1	6422	0.809593	-0.15048	No
Myot	6673	0.755486	-0.158795	No
Clstn1	6848	0.719809	-0.163808	No
Pecam1	6914	0.710079	-0.163919	No
Nectin2	7288	0.637217	-0.178282	No

Ptpn23	7575	0.591455	-0.188885	No
Fat1	7580	0.590648	-0.186707	No
Celsr1	7874	0.545922	-0.197809	No
Bsg	8086	0.511762	-0.20533	No
Pcdh12	8287	0.48131	-0.212474	No
Dsg2	8410	0.463002	-0.216155	No
Celsr2	8703	0.420015	-0.227714	No
Nrcam	8796	0.410229	-0.230247	No
Pcdh1	8928	0.392957	-0.234616	No
Pcdh18	8954	0.38924	-0.234194	No
Plxnb2	9204	0.357649	-0.244053	No
Esam	9444	0.332823	-0.253558	No
Cdh26	9803	0.287436	-0.268639	No
Igsf9	10464	0.213758	-0.297704	No
Cdh16	10732	0.185294	-0.309067	No
Cdh6	10747	0.183532	-0.308968	No
Nexn	10958	0.162306	-0.31784	No
Igsf11	11185	0.142827	-0.327514	No
Cdhr5	11230	0.139512	-0.328952	No
Cdh5	11878	0.080462	-0.357959	No
Cdhr1	11932	0.0746	-0.360064	No
Ret	11952	0.072949	-0.360634	No
Pcdh10	12136	0.056198	-0.368705	No
Cdh20	12634	0.014725	-0.391176	No
Hmcn1	12891	-0.006336	-0.402755	No
Cadm3	13023	-0.017384	-0.408624	No
Fat2	14097	-0.116557	-0.456799	No
Sdk2	14105	-0.117942	-0.456645	No
Dsg1a	14191	-0.129399	-0.459981	No
Emb	14817	-0.2015	-0.487508	No
Cdh2	14872	-0.208112	-0.489125	No
Amigo1	15029	-0.230283	-0.495277	No
Clstn3	15099	-0.241203	-0.497441	No
Plxnb3	15314	-0.2717	-0.506057	No
Pcdh9	15964	-0.369592	-0.534001	No
Dsg4	16066	-0.38789	-0.53703	No
Cdh3	16162	-0.401621	-0.539733	No
Cdh7	16253	-0.416912	-0.542147	No
Dsg1c	16581	-0.489901	-0.555014	No
Ceacam5	16859	-0.543044	-0.565402	No
Cdhr18	17239	-0.632461	-0.580056	No
Cntn6	17288	-0.643554	-0.579662	No
Dsc2	17407	-0.679514	-0.582297	No
Pcdh7	17416	-0.681209	-0.57994	No
Cadm1	17423	-0.683322	-0.577482	No
Robo2	17496	-0.701195	-0.577946	No
Cntn5	17539	-0.70994	-0.577014	No
Nectin4	17661	-0.739877	-0.579545	No
Pcdh15	17766	-0.770728	-0.581181	No
Cdhr3	18507	-1.006063	-0.610708	No
Ceacam1	18517	-1.009571	-0.607084	No
Pcdh17	18784	-1.09947	-0.614751	No

Clstn2	18948	-1.176091	-0.617443	No
Cdh10	19386	-1.412188	-0.631613	No
Amigo2	19405	-1.42138	-0.626752	No
Fat4	19683	-1.576533	-0.633012	No
Dscam	19890	-1.715078	-0.635501	No
Cdhr17	20014	-1.802226	-0.633879	No
Sdk1	20349	-2.09322	-0.64066	Yes
Ptprt	20410	-2.160777	-0.63475	Yes
Ptprm	20438	-2.183562	-0.627254	Yes
Cdh12	20468	-2.221251	-0.619697	Yes
Mypn	20604	-2.381055	-0.616307	Yes
Igsf9b	20680	-2.456175	-0.609898	Yes
Pcdh11x	20688	-2.473868	-0.600335	Yes
Hmcn2	20772	-2.589729	-0.593755	Yes
Cdh4	20965	-2.895601	-0.590894	Yes
Cdh11	20979	-2.931188	-0.579777	Yes
Pcdh19	20984	-2.935877	-0.568233	Yes
Dchs2	21077	-3.157019	-0.559796	Yes
Cadm2	21151	-3.364946	-0.549666	Yes
Cdh18	21228	-3.705906	-0.538311	Yes
Fat3	21408	-4.471728	-0.528566	Yes
Dchs1	21433	-4.578033	-0.511371	Yes
Cdh8	21630	-6.196056	-0.49551	Yes
Cdh13	21788	-9.705371	-0.463866	Yes
Cdh22	21847	-11.584922	-0.420228	Yes
Palld	22153	-45.408546	-0.252705	Yes
Dsg3	22154	-63.320728	0.000181	Yes

Details

GO:0006303
Name
double-strand break repair via nonhomologous end joining
Definition
The repair of a double-strand break in DNA in which the two broken ends are rejoined with little or no sequence complementarity. Information at the DNA ends may be lost due to the modification of broken DNA ends. This term covers instances of separate pathways, called classical (or canonical) and alternative nonhomologous end joining (C-NHEJ and A-NHEJ). These in turn may further branch into sub-pathways, but evidence is still unclear.

Statistics

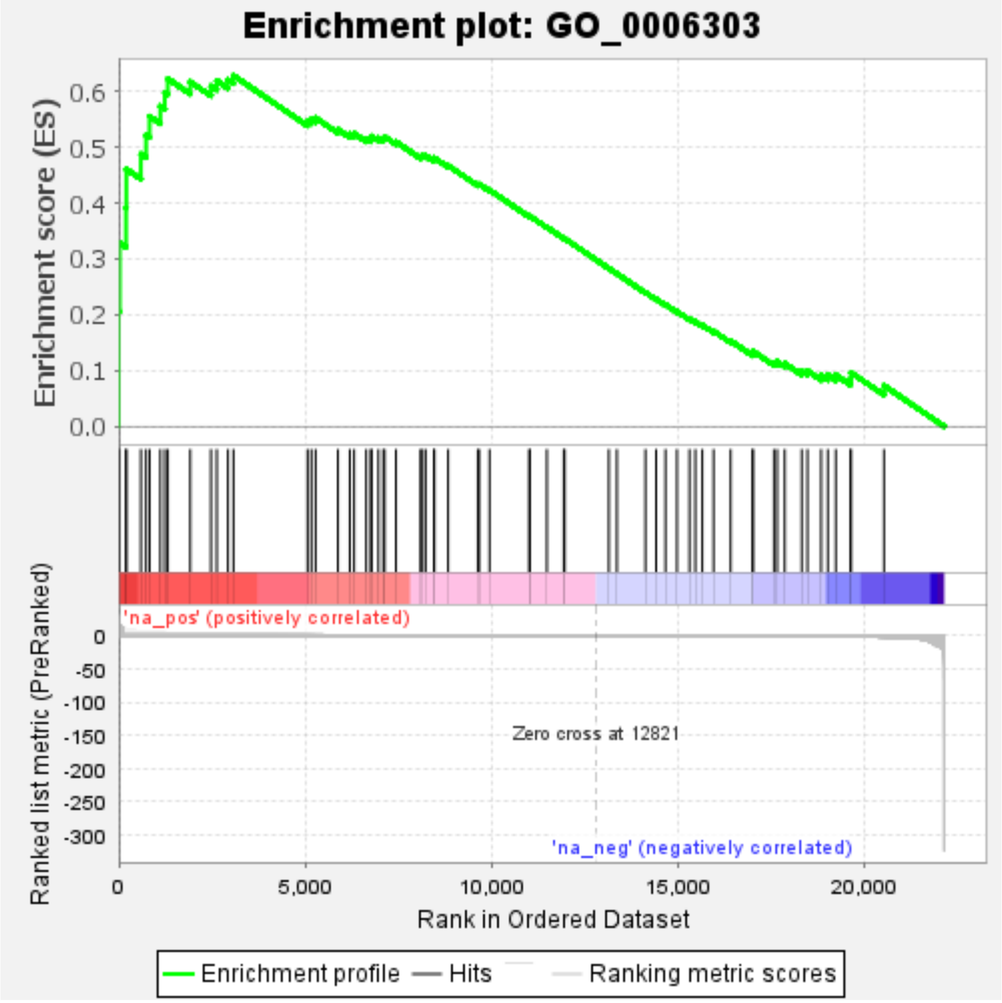
Enrichment plot: GO_0006303
Enrichment score (ES)
0.6
0.5
0.4
0.3
0.2
0.1
0.0
'na_pos' (positively correlated)
Zero cross at 12821
'na_neg' (negatively correlated)
Ranked list metric (PreRanked)
0
-50
-100
-150
-200
-250
-300
0
5,000
10,000
15,000
20,000
Rank in Ordered Dataset
Enrichment profile
Hits
Ranking metric scores

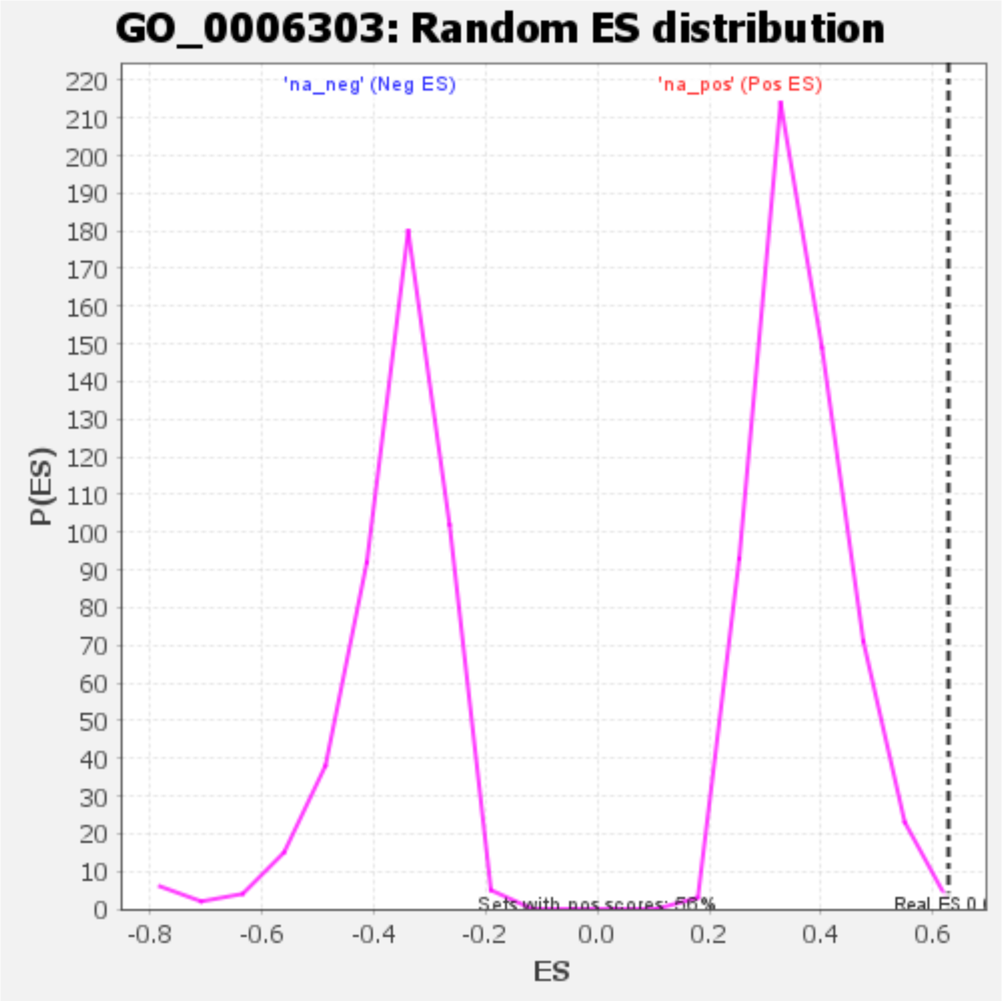

GSEA Details

Probe	Rank in Gene List	Rank Metric Score	Running ES	Core Enrichment
Nhej1	1	28.342026	0.20699	Yes
Hmga2	29	16.841778	0.328796	Yes
Shld1	198	9.639752	0.391609	Yes
Uvrag	206	9.489728	0.460613	Yes
Xrcc4	597	6.123104	0.487689	Yes
Dyrk1a	736	5.512959	0.521715	Yes
Mrnip	831	5.144987	0.555043	Yes
Mbtd1	1119	4.319115	0.573604	Yes
Cyren	1239	4.05015	0.597803	Yes
Rnf169	1320	3.87753	0.622507	Yes
Kmt5c	1922	2.960896	0.616933	Yes
Dntt	2477	2.404936	0.609425	Yes
Polm	2634	2.277156	0.618998	Yes
Pola1	2935	2.056203	0.62044	Yes
Psmd14	3082	1.964598	0.628182	Yes
Ercc4	5069	1.121125	0.546479	No
Mlh1	5171	1.085112	0.549834	No
Aste1	5286	1.047475	0.552326	No
Poll	5887	0.893638	0.531696	No
Smarcal1	6204	0.848907	0.523594	No

Wrap53	6321	0.830431	0.52441	No
Dclre1c	6646	0.761109	0.515304	No
Helq	6768	0.734776	0.515195	No
Kdm2a	6790	0.730568	0.519581	No
Epc1	6974	0.697421	0.516393	No
Parp3	7105	0.670439	0.515406	No
Kmt5b	7137	0.665179	0.518862	No
Hsf1	7445	0.609466	0.509418	No
Paxx	8102	0.509892	0.48345	No
Trp53bp1	8153	0.500953	0.484846	No
Prkdc	8238	0.48781	0.484608	No
Nudt16l1	8462	0.456445	0.477848	No
Lig3	8474	0.454384	0.480669	No
Smchd1	8845	0.404035	0.466873	No
Xrcc6	9641	0.308002	0.433139	No
Aplf	9675	0.303755	0.433864	No
Prpf19	9955	0.270469	0.423212	No
Mre11a	11023	0.156469	0.376059	No
Zbtb7a	11036	0.155334	0.37665	No
Top2b	11495	0.114373	0.356755	No
Ercc6	11949	0.0731	0.336785	No
Polq	11970	0.071739	0.336404	No
Kat5	13158	-0.028692	0.282886	No
Ercc8	13374	-0.045371	0.273486	No
Ercc6l2	14140	-0.122418	0.239754	No
Iffo1	14426	-0.154663	0.227984	No
Xrcc5	14678	-0.186171	0.217982	No
Ercc1	14987	-0.225123	0.205686	No
Nsd2	15322	-0.272922	0.192562	No
Dclre1b	15479	-0.295383	0.187658	No
Aunip	15667	-0.322793	0.181552	No
Shld2	15968	-0.370455	0.170679	No
Topbp1	16427	-0.451115	0.153244	No
Pnkp	17016	-0.576187	0.130838	No
Polb	17025	-0.578954	0.134705	No
Hmces	17590	-0.721502	0.114447	No
Plk1	17675	-0.744615	0.116085	No
Lig4	17879	-0.805527	0.11278	No
Xrcc1	18335	-0.950164	0.099127	No
Rnf168	18488	-1.001248	0.099561	No
Nbn	18855	-1.130422	0.091252	No
Mad2l2	19035	-1.21732	0.092042	No
Kdm4d	19249	-1.327296	0.092097	No
Ppp1ca	19644	-1.55086	0.085592	No
Rhno1	19654	-1.553201	0.096531	No
Dek	20542	-2.294792	0.073145	No

Details

GO:0045687
Name
positive regulation of glial cell differentiation
Definition
Any process that activates or increases the frequency, rate or extent of glia cell differentiation.

Statistics

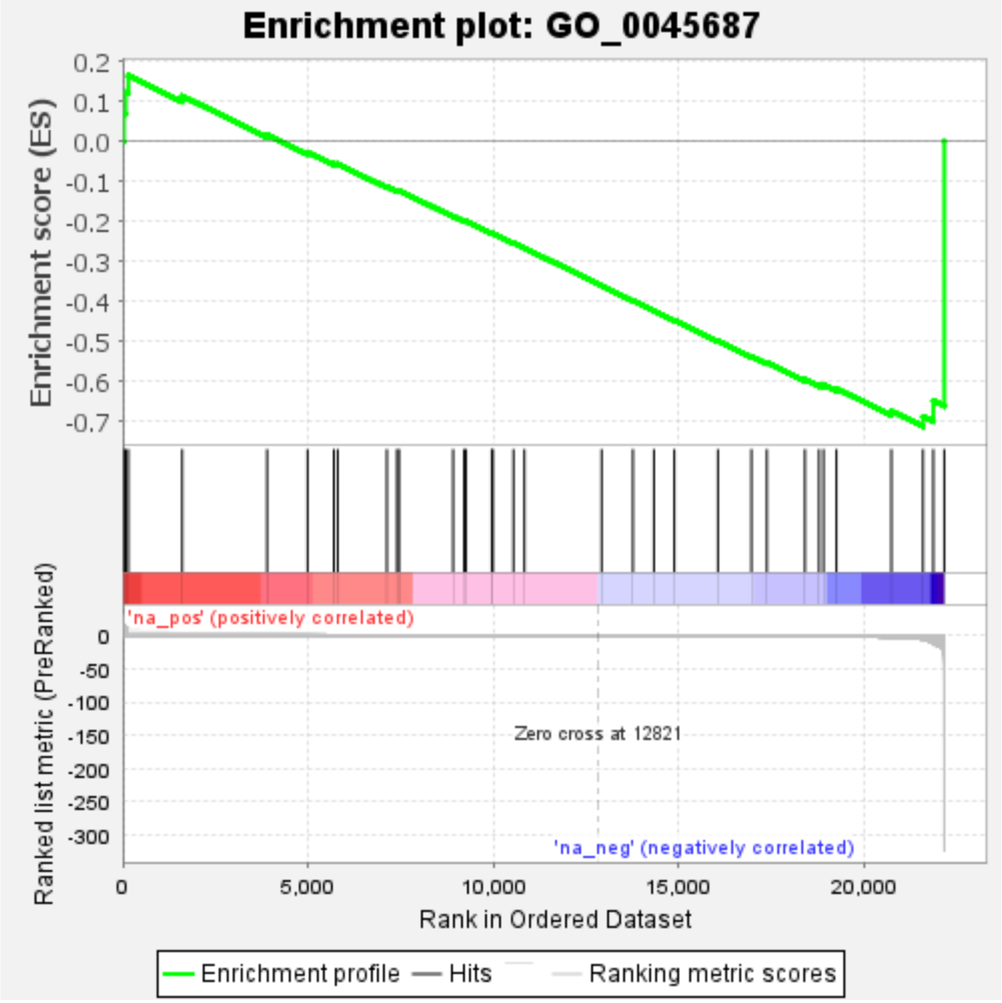
Enrichment plot: GO_0045687
Enrichment score (ES)
0.2
0.1
0.0
-0.1
-0.2
-0.3
-0.4
-0.5
-0.6
-0.7
Ranked list metric (PreRanked)
'na_pos' (positively correlated)
0
-50
-100
-150
-200
-250
-300
Zero cross at 12821
'na_neg' (negatively correlated)
0
5,000
10,000
15,000
20,000
Rank in Ordered Dataset
Enrichment profile
Hits
Ranking metric scores

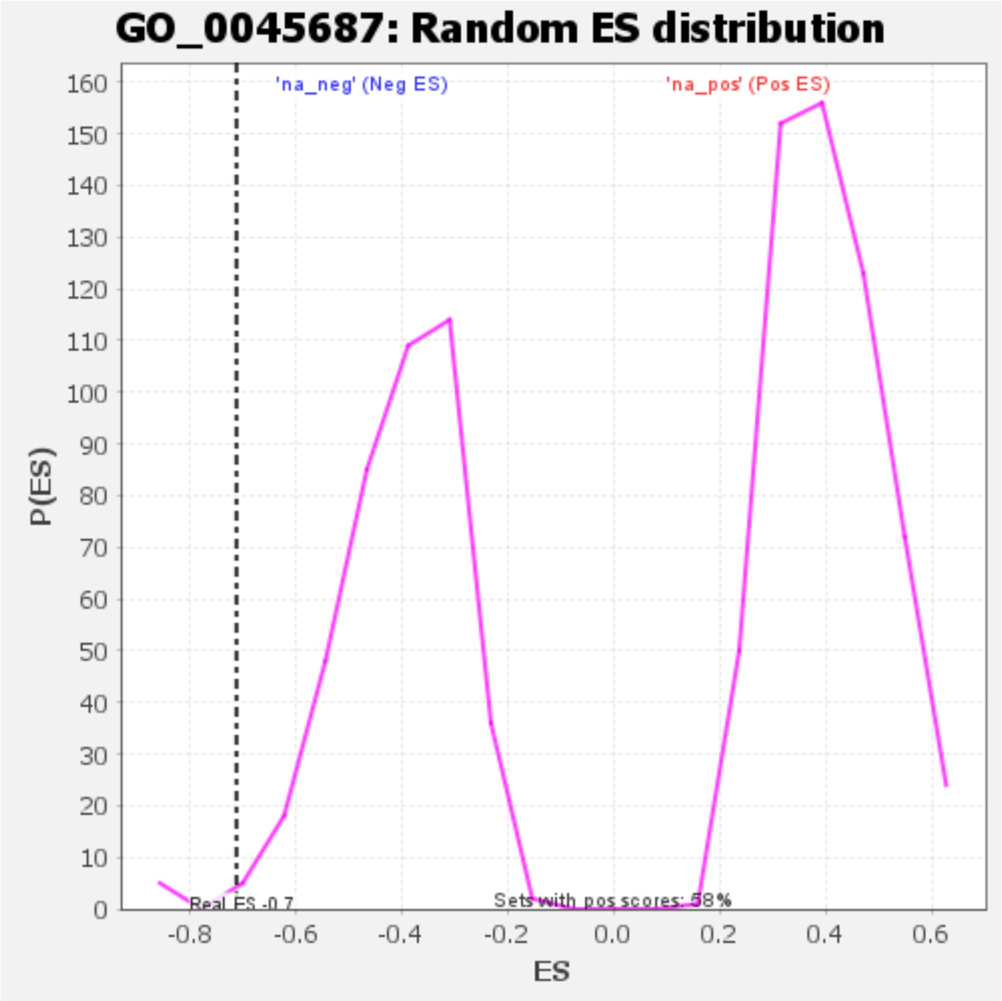
GO_0045687: Random ES distribution
'na_neg' (Neg ES)
'na_pos' (Pos ES)
P(ES)
160
150
140
130
120
110
100
90
80
70
60
50
40
30
20
10
0
Real ES -0.7
Sets with pos scores: 58%
-0.8
-0.6
-0.4
-0.2
0.0
0.2
0.4
0.6
ES

GSEA Details

Probe	Rank in Gene List	Rank Metric Score	Running ES	Core Enrichment
Il34	32	16.557499	0.070225	No
Tenm4	81	12.5979	0.122587	No
Myrf	155	10.371505	0.164182	No
Nkx6-2	1606	3.380059	0.113279	No
Tnfrsf1b	3895	1.556401	0.016609	No
Qki	4987	1.150033	-0.027722	No
Mir23a	5696	0.943057	-0.055638	No
Mtor	5800	0.913735	-0.056338	No
Nkx2-2	7125	0.666345	-0.113293	No
Notch1	7398	0.61685	-0.122916	No
Lif	7453	0.608222	-0.122724	No
Tgfb1	8911	0.394845	-0.186865	No
Id2	9209	0.357187	-0.198742	No
Dag1	9261	0.351513	-0.199525	No
Prpf19	9955	0.270469	-0.229675	No
Clcn2	9983	0.267036	-0.22974	No
Serpine2	10539	0.206722	-0.253928	No
Egr2	10834	0.173912	-0.266463	No
Spint1	12916	-0.008247	-0.36048	No
Zfp365	13760	-0.082579	-0.398222	No
Nkx2-2os	14327	-0.144121	-0.423179	No
Rheb	14877	-0.208553	-0.447089	No
Prmt5	16052	-0.385848	-0.498478	No
Bin1	16955	-0.562351	-0.536811	No
Hes1	17372	-0.668719	-0.552717	No
Ttbk1	18399	-0.971366	-0.594884	No
Hdac1	18778	-1.098518	-0.607213	No
Trp73	18899	-1.150795	-0.607655	No
Hdac2	19248	-1.326252	-0.617642	No
Actr3	20729	-2.527728	-0.67359	No
Pparg	21581	-5.66605	-0.687525	Yes
Ptn	21859	-12.066743	-0.647812	Yes
Ptprz1	22155	-152.769394	0.000136	Yes

Details

GO:0007179
Name
transforming growth factor beta receptor signaling pathway
Definition
The series of molecular signals initiated by an extracellular ligand binding to a transforming growth factor beta receptor on the surface of a target cell, and ending with the regulation of a downstream cellular process, e.g. transcription.

Statistics

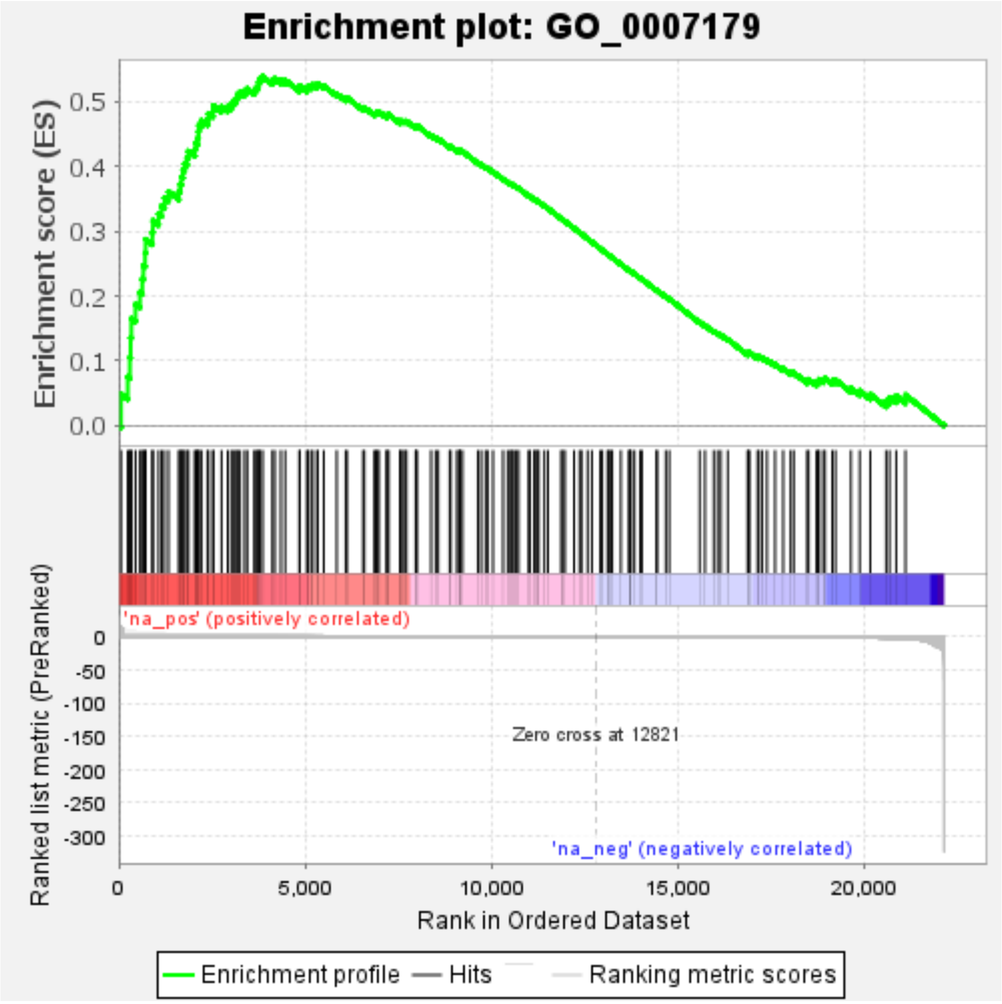
Enrichment plot: GO_0007179
Enrichment score (ES)
0.5
0.4
0.3
0.2
0.1
0.0
'na_pos' (positively correlated)
Ranked list metric (PreRanked)
0
-50
-100
-150
-200
-250
-300
Zero cross at 12821
'na_neg' (negatively correlated)
0
5,000
10,000
15,000
20,000
Rank in Ordered Dataset
Enrichment profile
Hits
Ranking metric scores

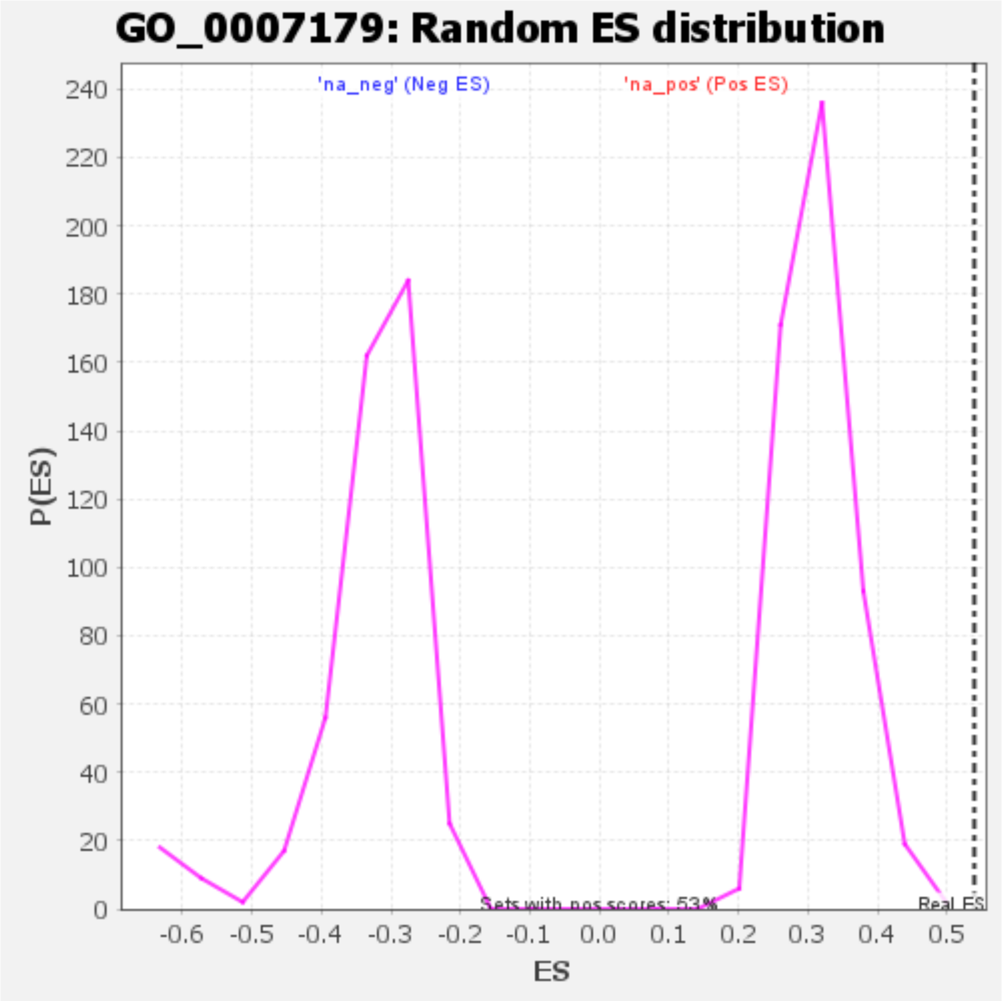

GSEA Details

Probe	Rank in Gene List	Rank Metric Score	Running ES	Core Enrichment
Ptprk	63	13.42469	0.048763	Yes
Acvr1	246	8.993923	0.075074	Yes
Aspn	302	8.473662	0.10516	Yes
Ldlrad4	321	8.197858	0.135868	Yes
Chst11	342	7.906844	0.165366	Yes
Nrros	453	7.025787	0.187383	Yes
Smurf1	573	6.264233	0.206062	Yes
Spred2	634	5.944603	0.226194	Yes
Nlk	665	5.805145	0.247155	Yes
Flcn	711	5.592781	0.266617	Yes
Smad1	725	5.554887	0.287388	Yes
Nodal	904	4.924191	0.29823	Yes
Wfikkn1	920	4.878657	0.31631	Yes
Zeb2	1071	4.448988	0.326598	Yes
Zmiz1	1153	4.235529	0.339203	Yes
Fut8	1231	4.060919	0.351318	Yes
Tsc22d1	1340	3.852747	0.361223	Yes
Ogt	1613	3.371674	0.361819	Yes
Hipk2	1654	3.314187	0.372746	Yes
Vasn	1685	3.263539	0.383932	Yes

Dcp1a	1727	3.21514	0.394432	Yes
Tgfbr3l	1769	3.157096	0.404709	Yes
Bmpr1a	1835	3.081489	0.413603	Yes
Rgcc	1871	3.02517	0.423645	Yes
Tgfbr3	2045	2.825573	0.426644	Yes
Atf3	2066	2.808528	0.436535	Yes
Pals1	2127	2.734398	0.444322	Yes
Arap1	2140	2.715129	0.454218	Yes
Smad3	2151	2.700476	0.464149	Yes
Brms1l	2222	2.632586	0.471089	Yes
Snx25	2384	2.501467	0.473387	Yes
Rasl11b	2410	2.476635	0.481775	Yes
Ltbp1	2530	2.362739	0.485449	Yes
Tgfbr2	2543	2.351661	0.493947	Yes
Smad4	2761	2.18647	0.492487	Yes
Smad7	2929	2.060643	0.492816	Yes
Cd109	3023	2.009701	0.496316	Yes
Gdnf	3080	1.965556	0.501328	Yes
Zfyve9	3158	1.922678	0.50522	Yes
Map3k7	3187	1.90787	0.511284	Yes
Itga8	3258	1.862903	0.515265	Yes
Sap130	3382	1.789745	0.516553	Yes
Appl1	3452	1.754031	0.520161	Yes
Zfp451	3639	1.656997	0.518074	Yes
Lemd3	3693	1.637911	0.521963	Yes
Ints9	3750	1.617455	0.525636	Yes
Crebbp	3759	1.615023	0.531483	Yes
Fos	3796	1.600049	0.535999	Yes
Rnf111	3867	1.570007	0.538854	Yes
Smad2	4126	1.467114	0.532762	No
Lrrc32	4190	1.441654	0.535441	No
Adamtsl2	4352	1.377085	0.533415	No
Creb1	4470	1.33206	0.533217	No
Ero1a	4852	1.19628	0.52049	No
Smurf2	4860	1.193609	0.524762	No
Gdf11	5055	1.126432	0.520271	No
Trim33	5076	1.118712	0.523663	No
Ccl2	5164	1.0883	0.523892	No
Pml	5207	1.07181	0.526104	No
Sirt1	5327	1.038854	0.524687	No
Stat3	5342	1.034073	0.528027	No
Bmp8a	5505	0.988295	0.52446	No
Tgfbr1	5854	0.902594	0.512104	No
Zfp703	6094	0.857976	0.504534	No
Nkx2-1	6128	0.855297	0.506323	No
Src	6563	0.776083	0.489569	No
Pxn	6594	0.771549	0.491172	No
Spry2	6864	0.717291	0.481697	No
Cilp	6906	0.712241	0.482571	No
Tgfb2	6943	0.703961	0.483641	No
Itgb5	6984	0.696237	0.484499	No
Ski	7193	0.655187	0.477559	No

Lats2	7205	0.653077	0.479571	No
Htra3	7246	0.646017	0.480236	No
Tab1	7555	0.594185	0.468513	No
Arid4b	7566	0.592366	0.470337	No
Appl2	7623	0.583666	0.470035	No
Spred3	7683	0.574573	0.469561	No
Itgb6	7724	0.566251	0.469919	No
Eng	7972	0.52866	0.460719	No
Bcl9l	7981	0.526745	0.462381	No
Cited2	8008	0.522456	0.463208	No
Itga3	8384	0.466299	0.447946	No
Lgals9	8509	0.449188	0.444034	No
Ltbp4	8579	0.439541	0.442587	No
Ltbp3	8885	0.398721	0.430249	No
Tgfb1	8911	0.394845	0.43063	No
Il17f	9080	0.37413	0.424428	No
Pmepa1	9146	0.364578	0.422874	No
Arid4a	9151	0.364011	0.424092	No
Men1	9154	0.363722	0.4254	No
Bmpr1b	9232	0.355295	0.423265	No
Wnt1	9642	0.307959	0.405848	No
Sinhcaf	9728	0.296662	0.403123	No
Usp15	9849	0.282434	0.398752	No
Tgfb3	9904	0.275467	0.397355	No
Gcnt2	10048	0.258716	0.391847	No
Adam9	10316	0.230289	0.380589	No
Spred1	10442	0.215658	0.375734	No
Brms1	10520	0.208252	0.373033	No
Onecut2	10545	0.205995	0.372733	No
Ep300	10596	0.199866	0.371228	No
Twsg1	10671	0.191441	0.368599	No
Lpxn	10736	0.184914	0.366399	No
Adam17	11005	0.157972	0.354818	No
Zbtb7a	11036	0.155334	0.354051	No
Zyx	11159	0.144677	0.349059	No
Sin3a	11184	0.142855	0.348517	No
Fermt1	11236	0.139045	0.346732	No
Pdpk1	11273	0.13556	0.345616	No
Usp9x	11429	0.121209	0.339033	No
Smad5	11523	0.112577	0.335237	No
Cdh5	11878	0.080462	0.319446	No
Dab2	11923	0.075774	0.317737	No
Amhr2	11978	0.07106	0.315554	No
Slc2a10	12225	0.048412	0.304552	No
Fbn2	12373	0.035943	0.298005	No
Zeb1	12424	0.031583	0.295853	No
Il13ra2	12587	0.017691	0.288553	No
Skil	12702	0.008936	0.283403	No
Emilin1	12929	-0.009609	0.273161	No
Hsp90ab1	12980	-0.01433	0.270942	No
Jun	13124	-0.0254	0.264537	No
Fam89b	13155	-0.028537	0.263282	No

Stk11	13204	-0.032148	0.261223	No
Glg1	13249	-0.035895	0.25936	No
Lats1	13480	-0.054864	0.24911	No
Trp53	13709	-0.077809	0.23904	No
Sap30l	13712	-0.077828	0.239249	No
Npnt	13715	-0.078424	0.239459	No
Rbbp4	13751	-0.081801	0.238182	No
Ing1	13846	-0.0908	0.234256	No
Snx6	14001	-0.107298	0.227665	No
Thbs1	14044	-0.11163	0.226184	No
Htra1	14441	-0.156114	0.208775	No
Gipc1	14450	-0.156708	0.209014	No
Bmp8b	14686	-0.186946	0.199045	No
Adissp	14781	-0.197333	0.195529	No
Selenon	15595	-0.311682	0.159753	No
Dand5	15739	-0.335151	0.154538	No
Suds3	15973	-0.370956	0.145368	No
Veph1	16077	-0.389572	0.142182	No
Cdh3	16162	-0.401621	0.139906	No
Smad6	16350	-0.434886	0.133074	No
Stub1	16883	-0.548249	0.110987	No
Ovol2	16901	-0.551379	0.112335	No
Rbbp7	16931	-0.557519	0.11316	No
Fbn1	17151	-0.610537	0.105548	No
Arrb2	17183	-0.618541	0.106517	No
Ppm1a	17270	-0.639547	0.105065	No
Fermt2	17402	-0.677405	0.101712	No
Got1	17620	-0.728551	0.094645	No
Sap30	17837	-0.790432	0.087861	No
Sdcbp	18038	-0.851778	0.082041	No
Prdm16	18120	-0.876263	0.081727	No
Strap	18480	-0.999256	0.069243	No
Spry1	18520	-1.010393	0.071355	No
Dusp22	18732	-1.082054	0.06592	No
Hdac1	18778	-1.098518	0.068098	No
Snw1	18799	-1.105637	0.071441	No
Axin1	18917	-1.162081	0.070589	No
Cav2	18955	-1.178988	0.07344	No
Myocd	19155	-1.273388	0.069287	No
Hspa5	19241	-1.324145	0.070513	No
Tet1	19655	-1.554426	0.057708	No
Itgb8	19891	-1.715178	0.053617	No
Cited1	20174	-1.932092	0.048222	No
Ppara	20599	-2.376218	0.038077	No
Ing2	20692	-2.480095	0.043431	No
Parp1	20870	-2.742377	0.045927	No
Smad9	21119	-3.277539	0.047253	No
