# Supplementary material for: Berry-derived gold nanoparticles induce integrated ROS-mediated apoptosis, immune modulation, and transcriptomic remodeling in 4T1 triple-negative cancer cells
Source: Cell Death Discov. 2026 Apr 10;12:225. doi: 10.1038/s41420-026-03023-z (PMC13184259; doi:10.1038/s41420-026-03023-z)
Supplement: Supplementary file 4 — Figure S4 [file 41420_2026_3023_MOESM4_ESM.pdf]

BD FACSDiva 9.0

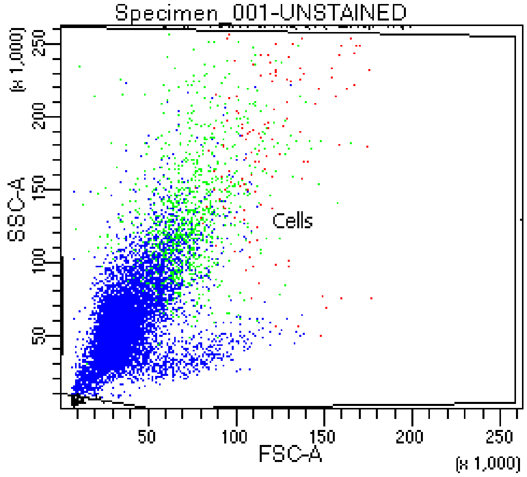

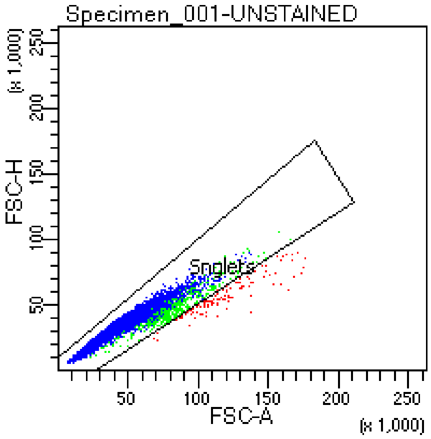

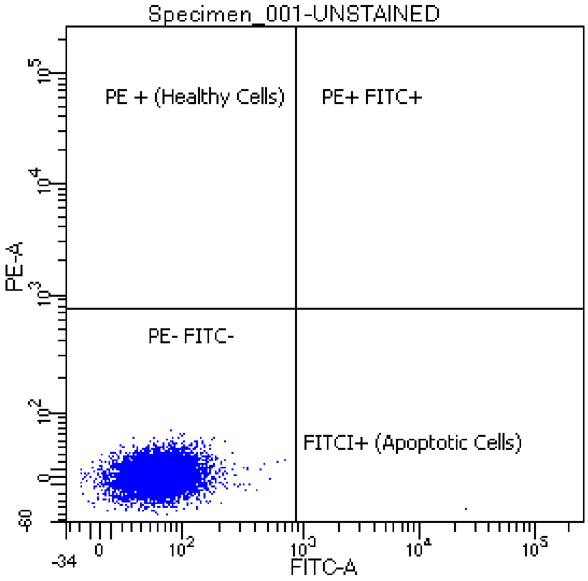

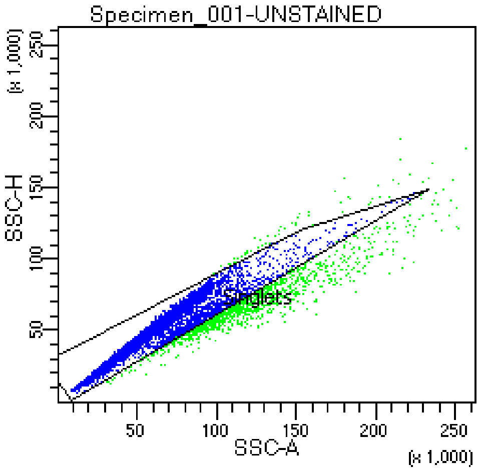

Tube: UNSTAINED

Population	#Events	%Parent	%Total
All Events	11,736	####	100.0
Cells	11,528	98.2	98.2
Snglets	11,400	98.9	97.1
Singlets	10,099	88.6	86.1
PE + (Healthy Cells)	0	0.0	0.0
PE+ FITC+	0	0.0	0.0
PE- FITC-	10,098	100.0	86.0
FITCI+ (Apoptotic Cell	1	0.0	0.0

BD FACSDiva 9.0

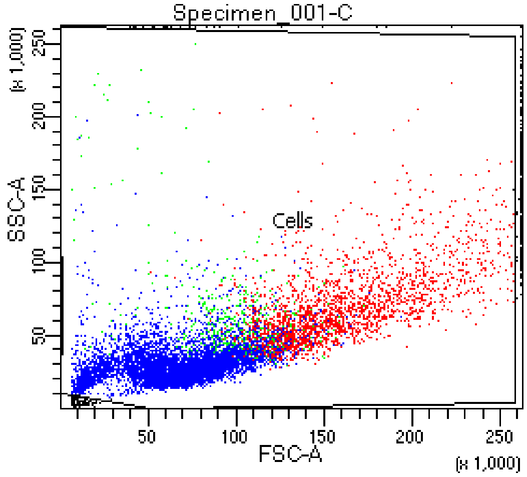

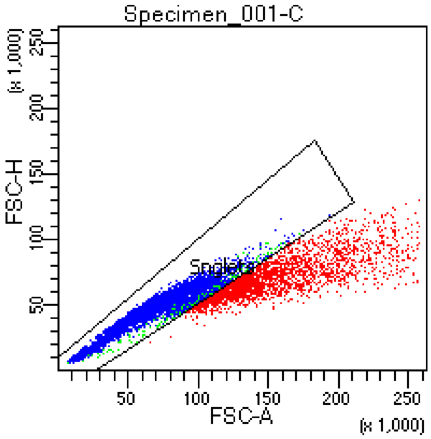

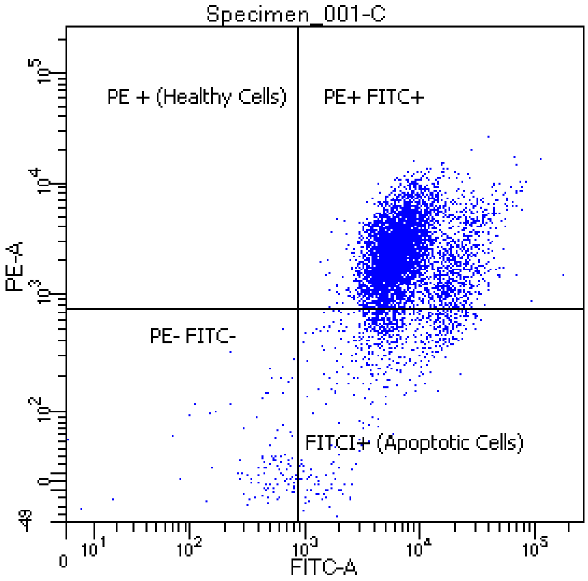

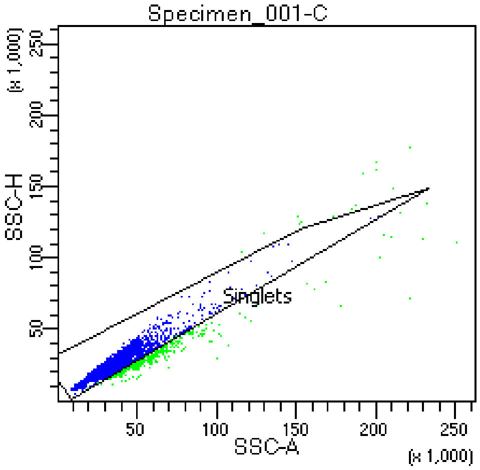

Tube: C

Population	#Events	%Parent	%Total
All Events	9,799	####	100.0
Cells	9,383	95.8	95.8
Snglets	6,822	72.7	69.6
Singlets	6,285	92.1	64.1
PE + (Healthy Cells)	0	0.0	0.0
PE+ FITC+	5,393	85.8	55.0
PE- FITC-	99	1.6	1.0
FITCI+ (Apoptotic Cell	793	12.6	8.1

BD FACSDiva 9.0

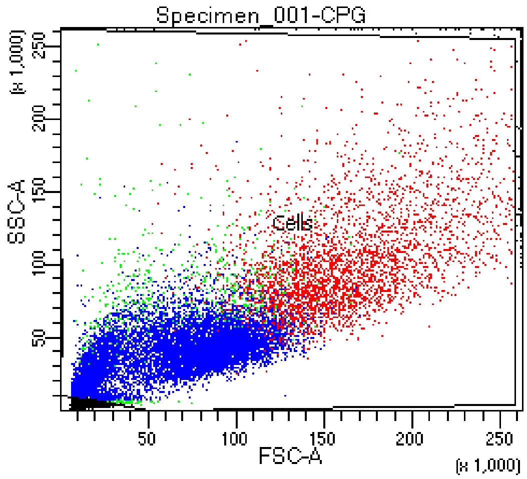

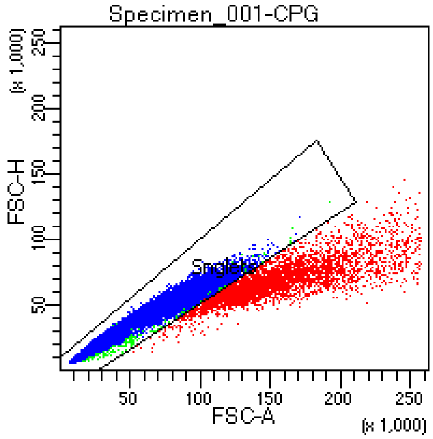

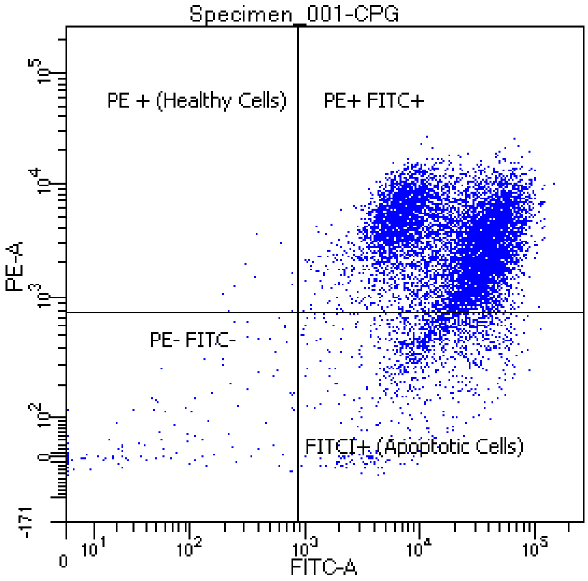

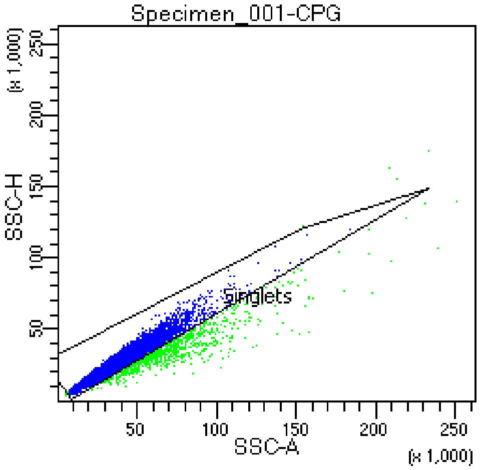

Tube: CPG

Population	#Events	%Parent	%Total
All Events	18,957	####	100.0
Cells	14,710	77.6	77.6
Snglets	10,962	74.5	57.8
Singlets	10,000	91.2	52.8
PE + (Healthy Cells)	10	0.1	0.1
PE+ FITC+	8,686	86.9	45.8
PE- FITC-	120	1.2	0.6
FITCl+ (Apoptotic Cell	1,184	11.8	6.2

BD FACSDiva 9.0

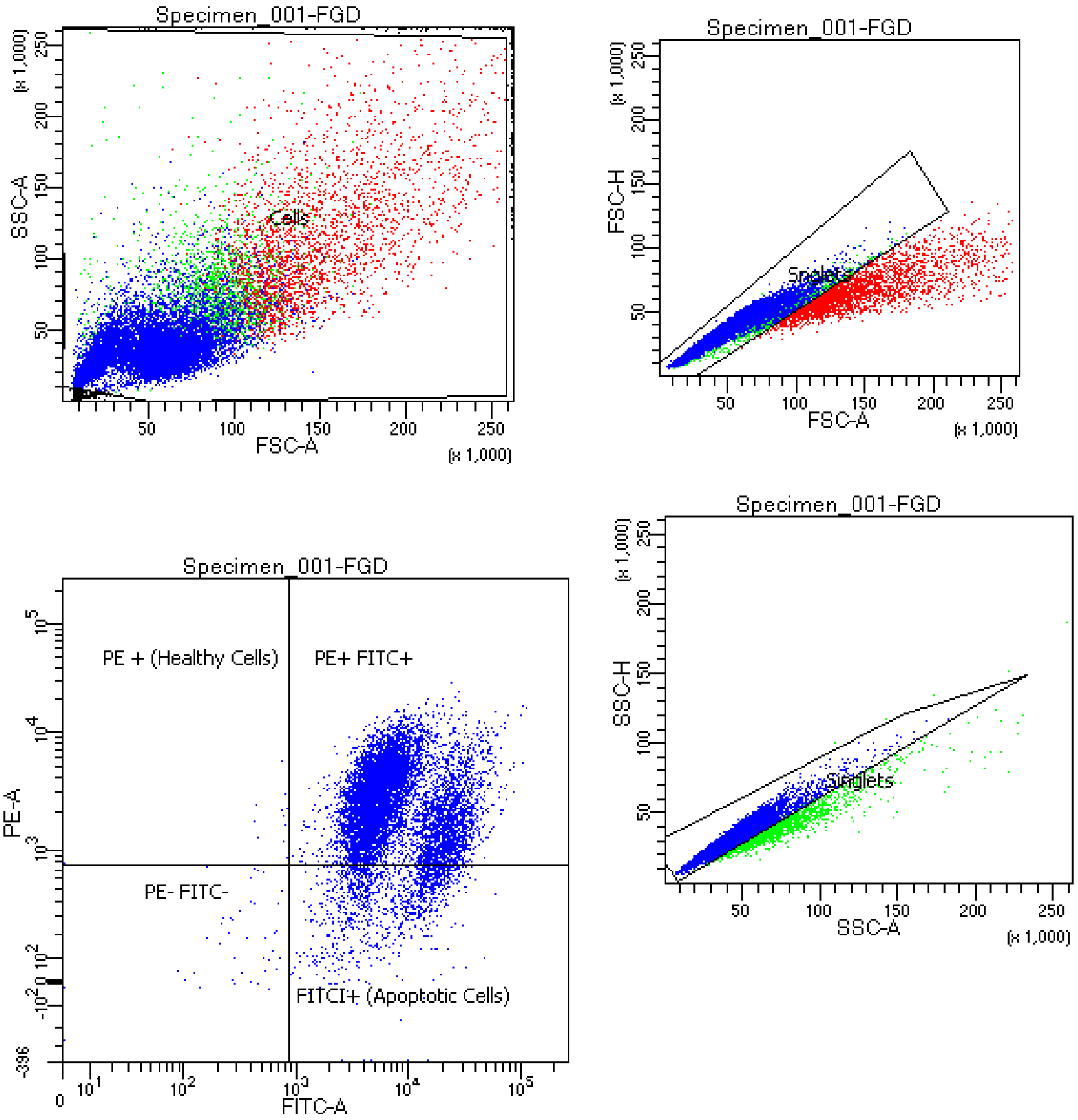

Tube: FGD

Population	#Events	%Parent	%Total
All Events	15,395	####	100.0
Cells	14,664	95.3	95.3
Snglets	11,749	80.1	76.3
Singlets	10,000	85.1	65.0
PE + (Healthy Cells)	12	0.1	0.1
PE+ FITC+	8,604	86.0	55.9
PE- FITC-	41	0.4	0.3
FITCI+ (Apoptotic Cell	1,343	13.4	8.7

BD FACSDiva 9.0

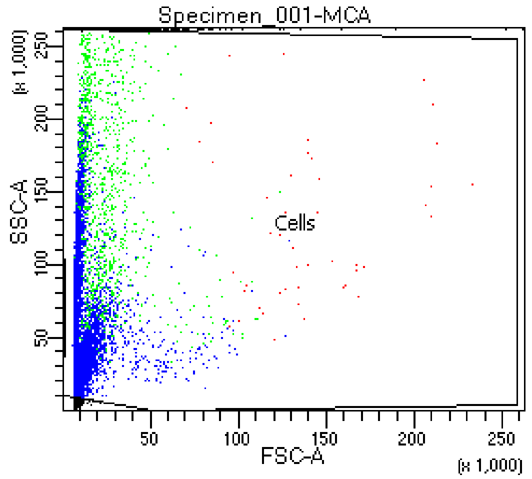

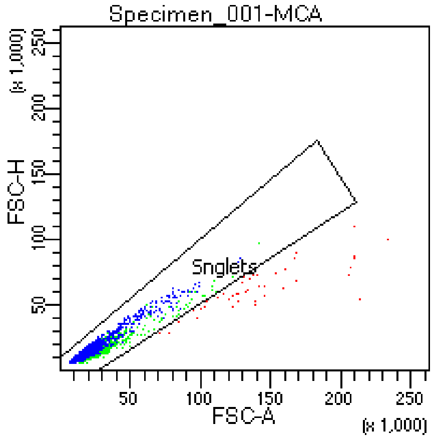

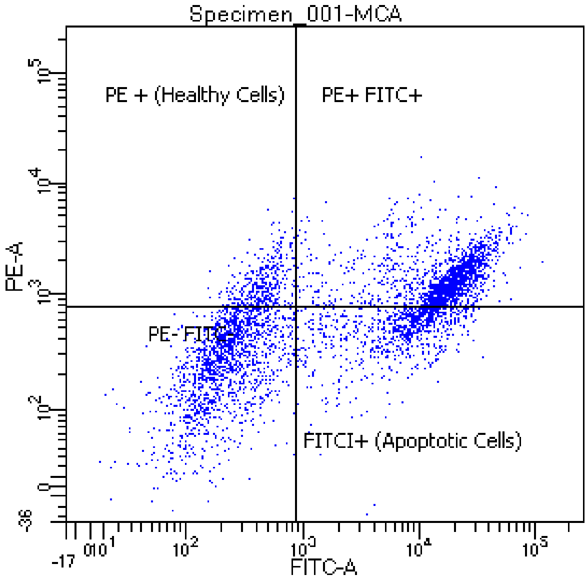

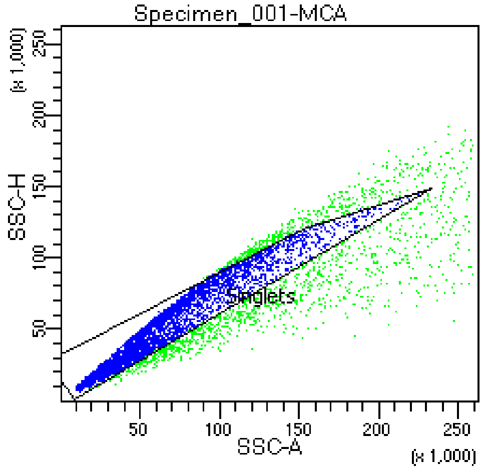

Tube: MCA

Population	#Events	%Parent	%Total
All Events	6,576	####	100.0
Cells	5,988	91.1	91.1
Snglets	5,942	99.2	90.4
Singlets	4,421	74.4	67.2
PE + (Healthy Cells)	373	8.4	5.7
PE+ FITC+	1,657	37.5	25.2
PE- FITC-	1,549	35.0	23.6
FITCI+ (Apoptotic Cell	842	19.0	12.8

BD FACSDiva 9.0

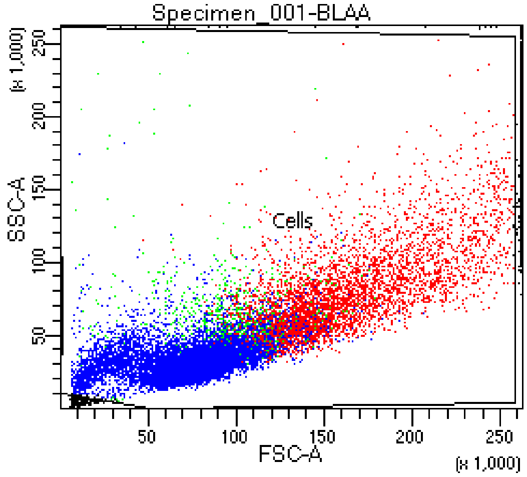

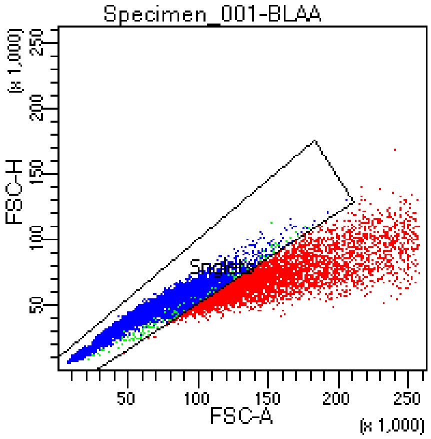

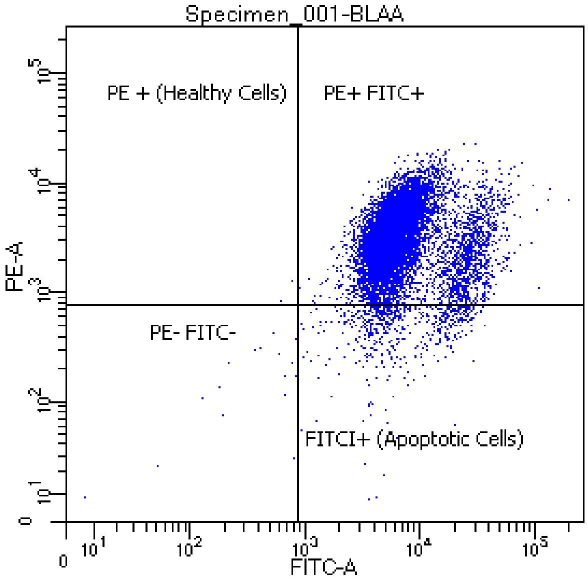

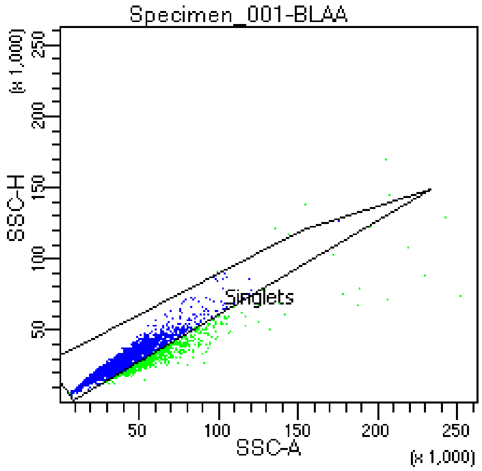

Tube: BLAA

Population	#Events	%Parent	%Total
All Events	17,156	####	100.0
Cells	15,973	93.1	93.1
Singlets	10,873	68.1	63.4
Singlets	10,000	92.0	58.3
PE + (Healthy Cells)	3	0.0	0.0
PE+ FITC+	9,404	94.0	54.8
PE- FITC-	17	0.2	0.1
FITCI+ (Apoptotic Cell	576	5.8	3.4

BD FACSDiva 9.0

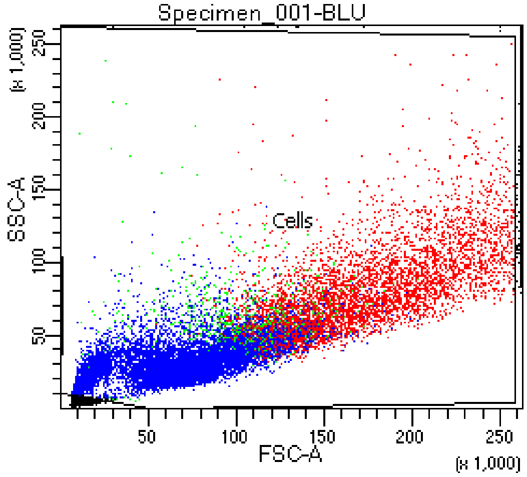

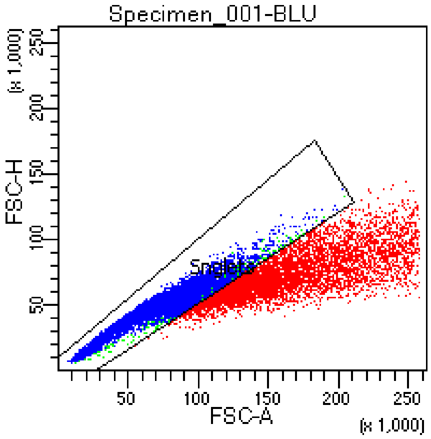

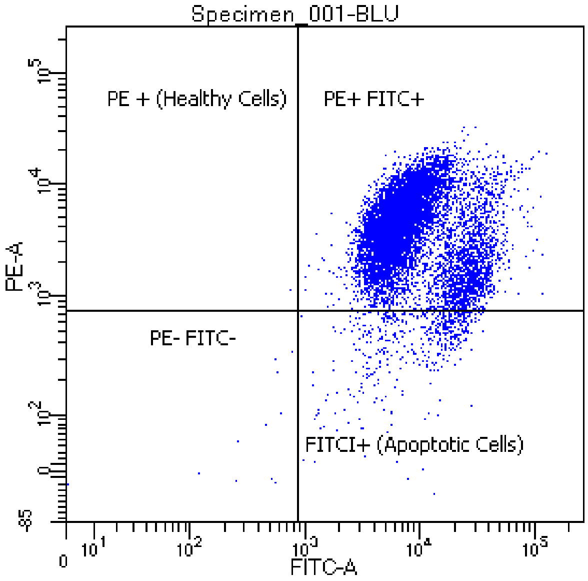

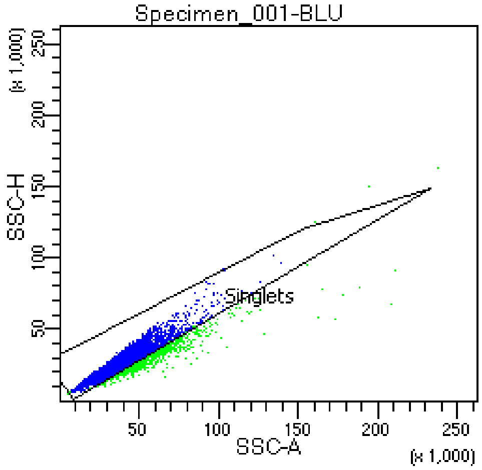

Tube: BLU

Population	#Events	%Parent	%Total
All Events	19,793	####	100.0
Cells	17,199	86.9	86.9
Snglets	11,107	64.6	56.1
Singlets	10,000	90.0	50.5
PE + (Healthy Cells)	2	0.0	0.0
PE+ FITC+	9,451	94.5	47.7
PE- FITC-	11	0.1	0.1
FITCI+ (Apoptotic Cell	536	5.4	2.7

BD FACSDiva 9.0

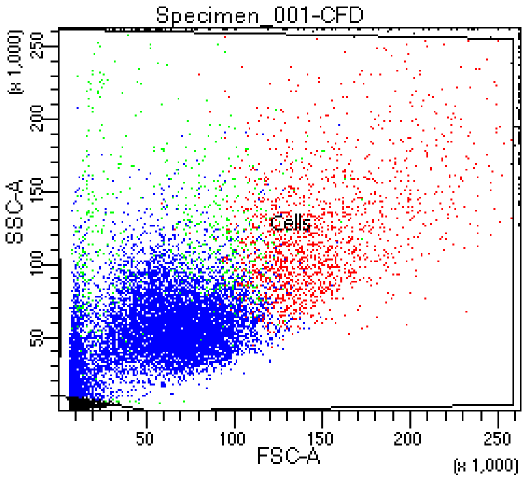

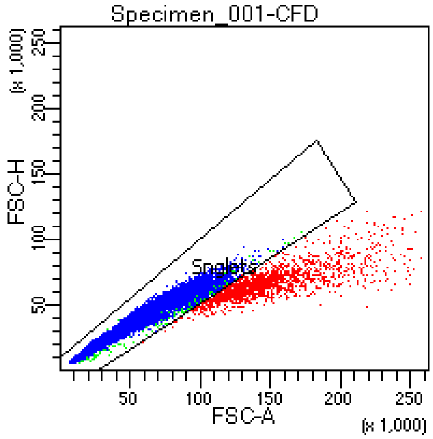

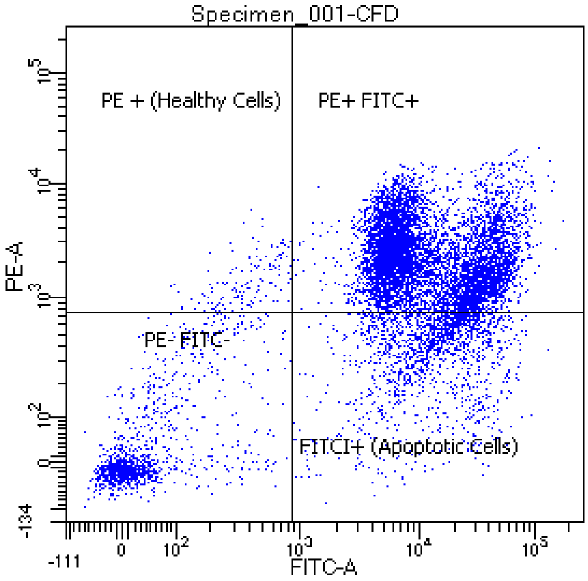

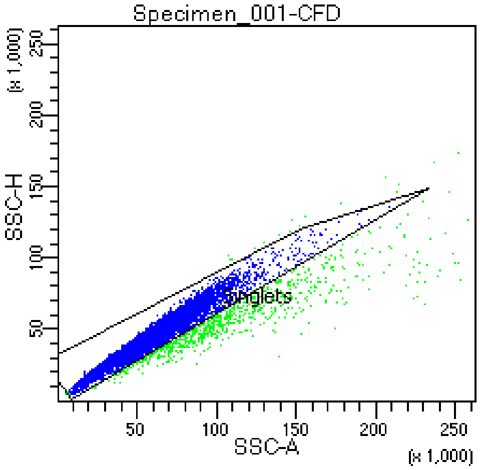

Tube: CFD

Population	#Events	%Parent	%Total
All Events	14,529	####	100.0
Cells	12,537	86.3	86.3
Snglets	10,839	86.5	74.6
Singlets	10,000	92.3	68.8
PE + (Healthy Cells)	139	1.4	1.0
PE+ FITC+	6,982	69.8	48.1
PE- FITC-	1,218	12.2	8.4
FITCI+ (Apoptotic Cell	1,661	16.6	11.4

BD FACSDiva 9.0

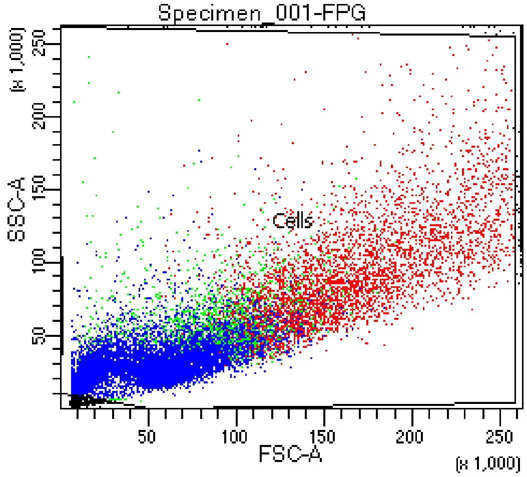

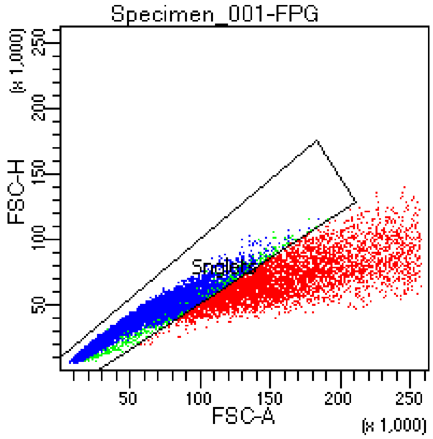

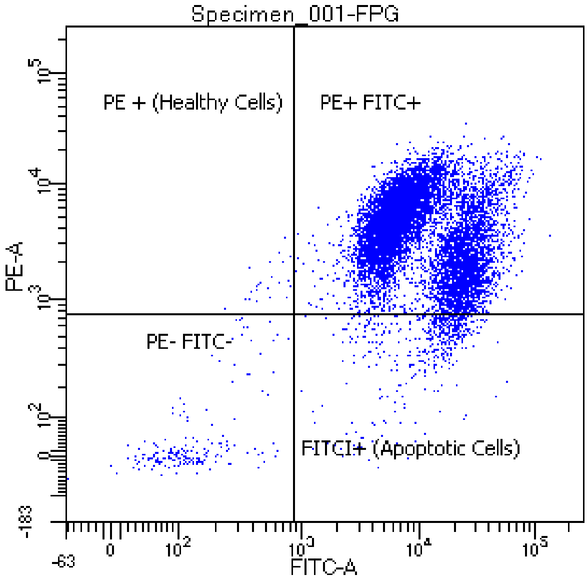

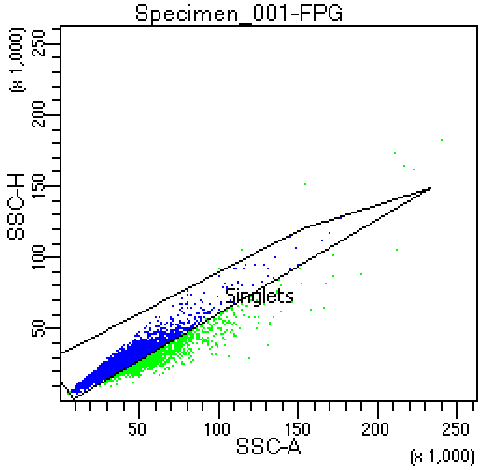

Tube: FPG

Population	#Events	%Parent	%Total
All Events	18,604	####	100.0
Cells	16,588	89.2	89.2
Snglets	11,851	71.4	63.7
Singlets	10,000	84.4	53.8
PE + (Healthy Cells)	22	0.2	0.1
PE+ FITC+	9,226	92.3	49.6
PE- FITC-	186	1.9	1.0
FITCI+ (Apoptotic Cell	566	5.7	3.0

BD FACSDiva 9.0

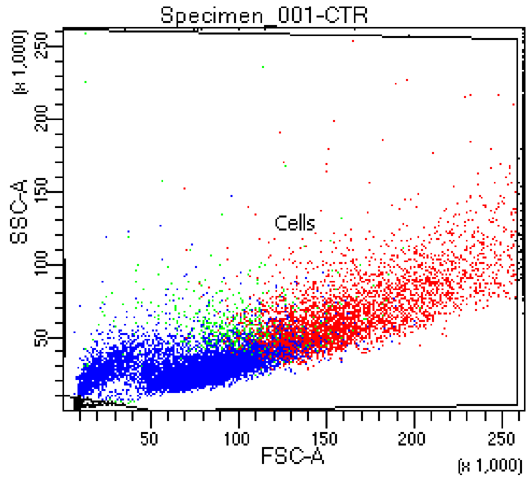

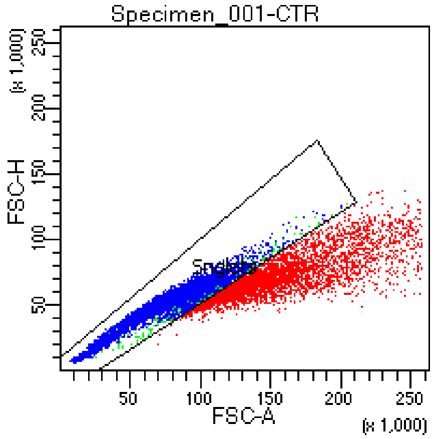

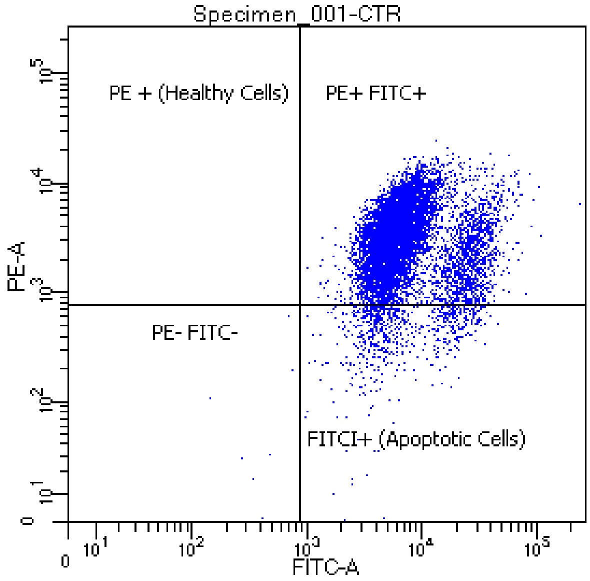

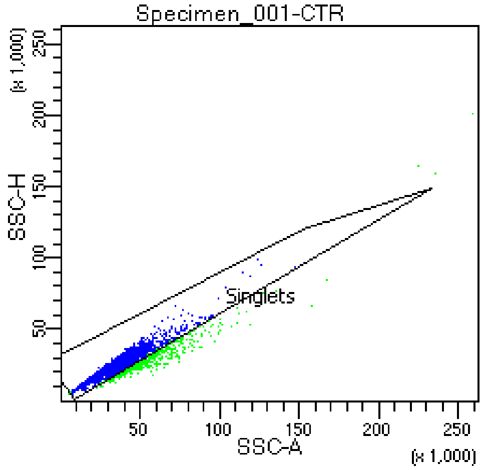

Tube: CTR

Population	#Events	%Parent	%Total
All Events	16,068	####	100.0
Cells	15,138	94.2	94.2
Snglets	10,698	70.7	66.6
Singlets	10,000	93.5	62.2
PE + (Healthy Cells)	0	0.0	0.0
PE+ FITC+	9,256	92.6	57.6
PE- FITC-	7	0.1	0.0
FITCI+ (Apoptotic Cell	737	7.4	4.6

BD FACSDiva 9.0

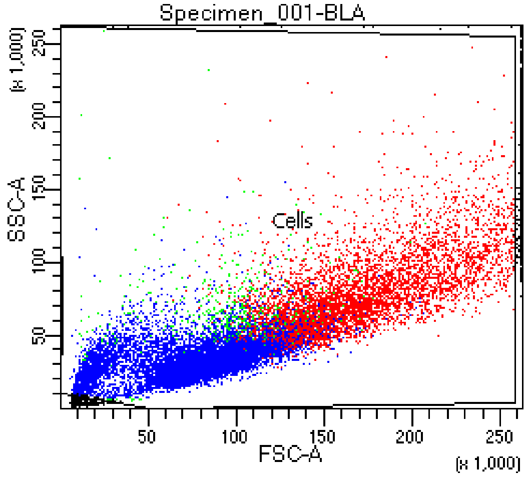

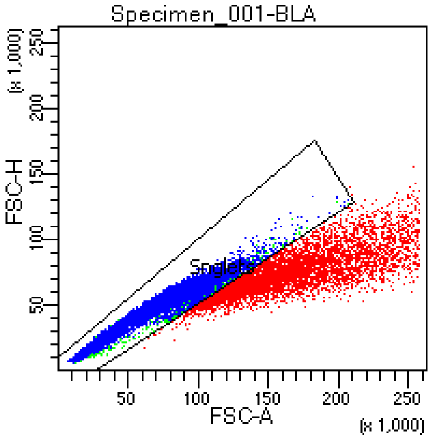

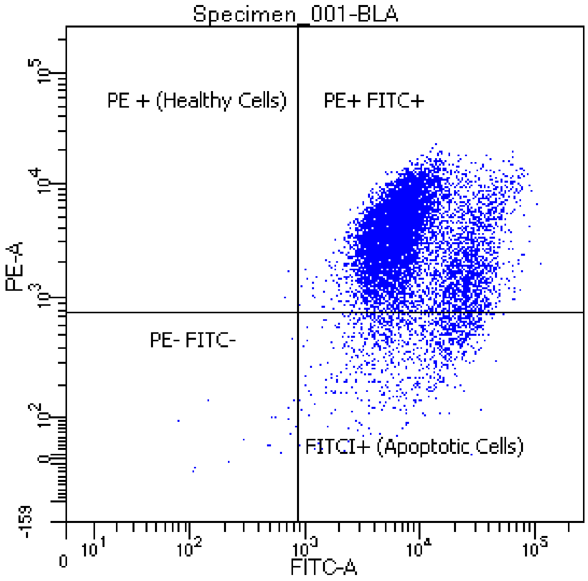

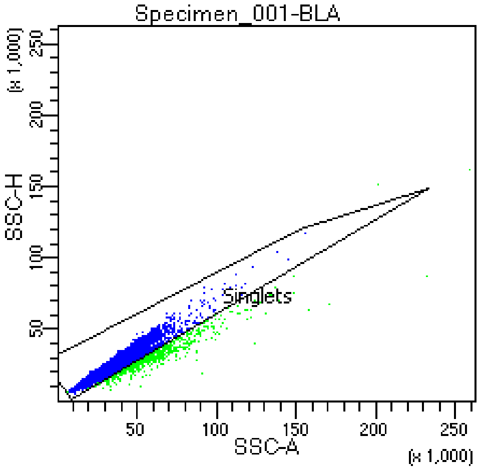

Tube: BLA

Population	#Events	%Parent	%Total
All Events	19,059	####	100.0
Cells	17,191	90.2	90.2
Singlets	10,775	62.7	56.5
Singlets	10,000	92.8	52.5
PE + (Healthy Cells)	2	0.0	0.0
PE+ FITC+	8,945	89.4	46.9
PE- FITC-	19	0.2	0.1
FITCI+ (Apoptotic Cell	1,034	10.3	5.4

BD FACSDiva 9.0

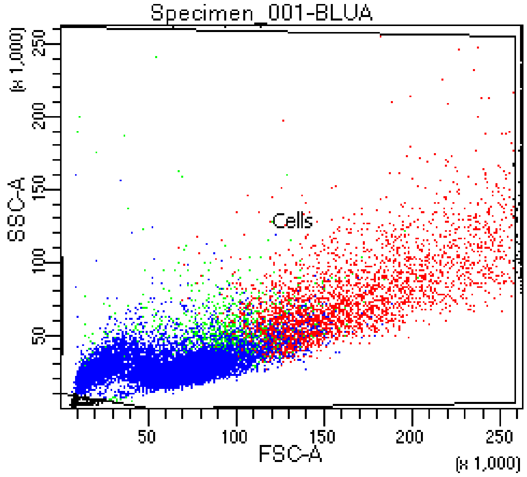

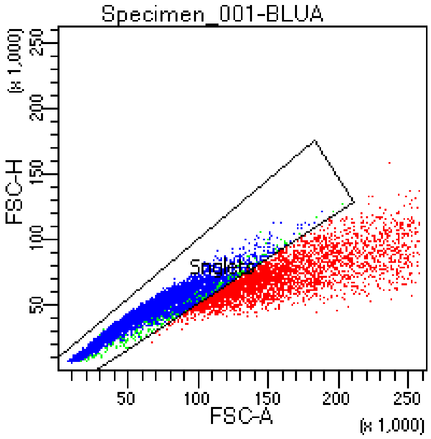

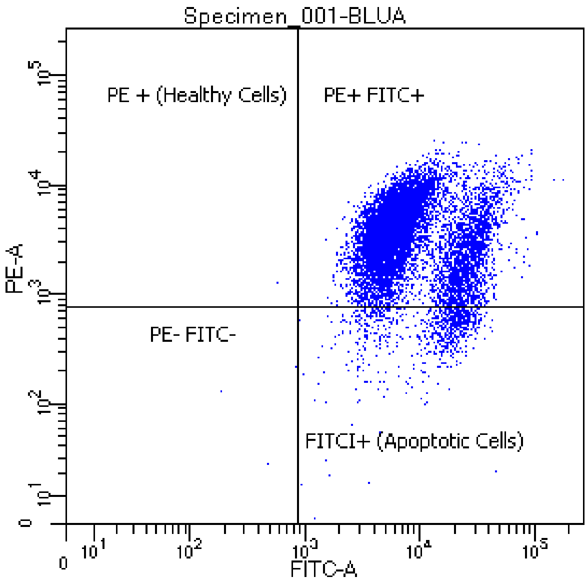

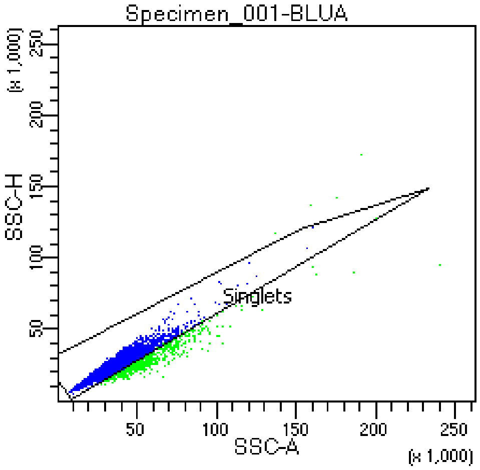

Tube: BLUA

Population	#Events	%Parent	%Total
All Events	15,463	####	100.0
Cells	14,382	93.0	93.0
Snglets	10,967	76.3	70.9
Singlets	10,000	91.2	64.7
PE + (Healthy Cells)	1	0.0	0.0
PE+ FITC+	9,215	92.2	59.6
PE- FITC-	3	0.0	0.0
FITCI+ (Apoptotic Cell	781	7.8	5.1

BD FACSDiva 9.0

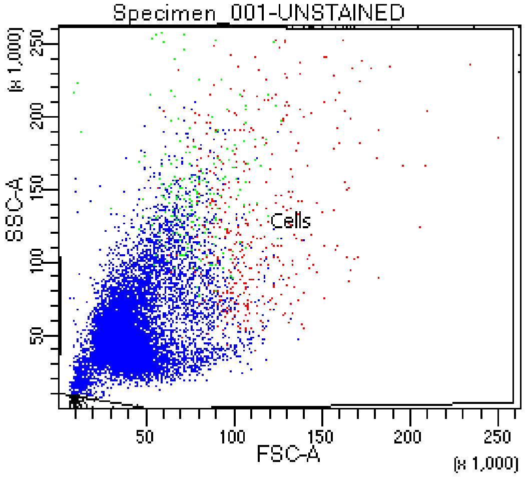

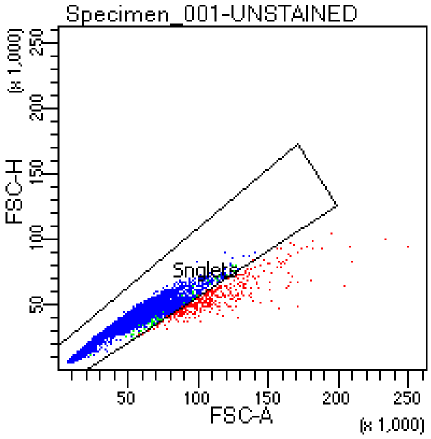

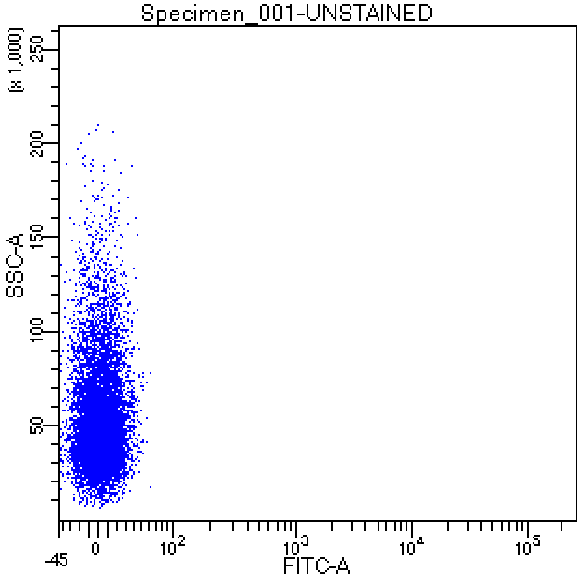

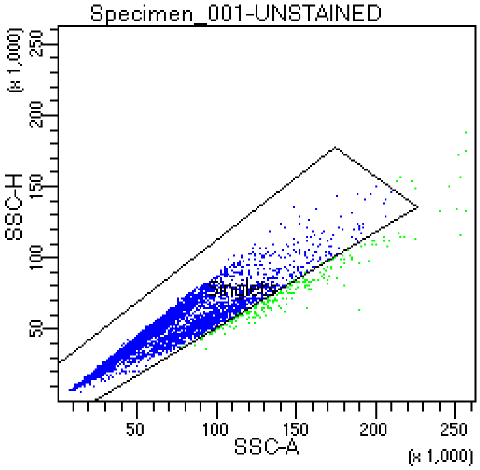

Tube: UNSTAINED

Population	#Events	%Parent	%Total
All Events	10,876	####	100.0
Cells	10,657	98.0	98.0
Snglets	10,242	96.1	94.2
Singlets	10,000	97.6	91.9

BD FACSDiva 9.0

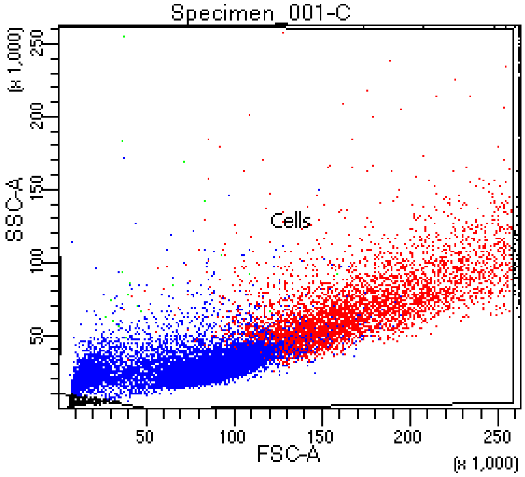

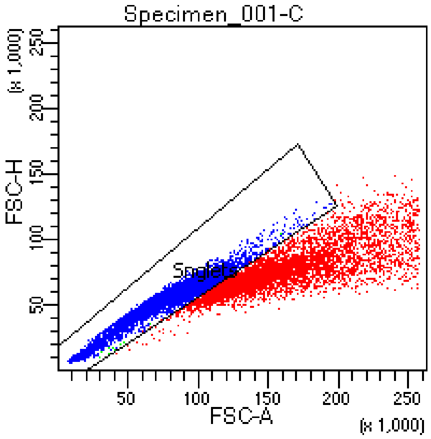

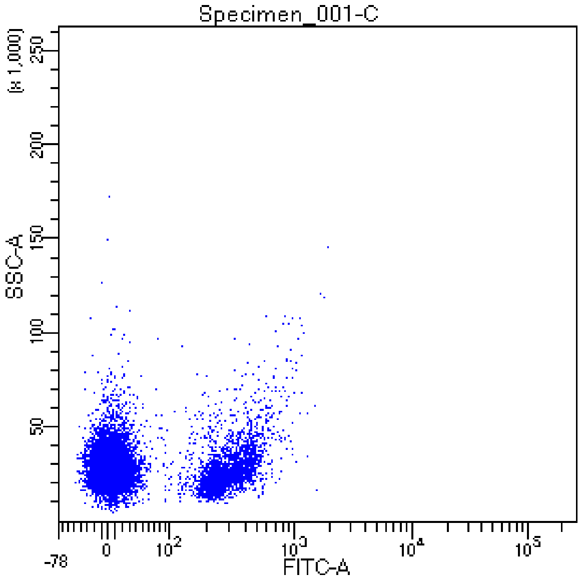

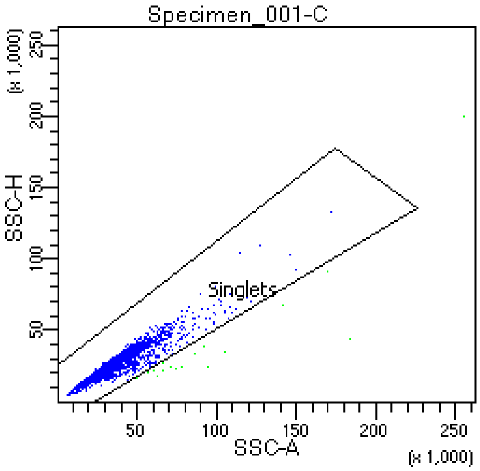

Tube: C

Population	#Events	%Parent	%Total
All Events	16,957	####	100.0
Cells	15,398	90.8	90.8
Snglets	10,019	65.1	59.1
Singlets	10,000	99.8	59.0

BD FACSDiva 9.0

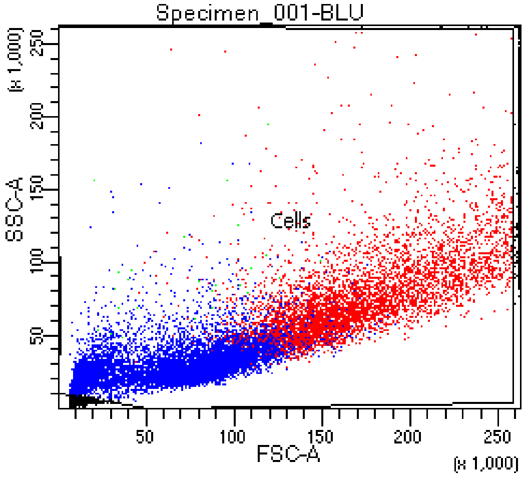

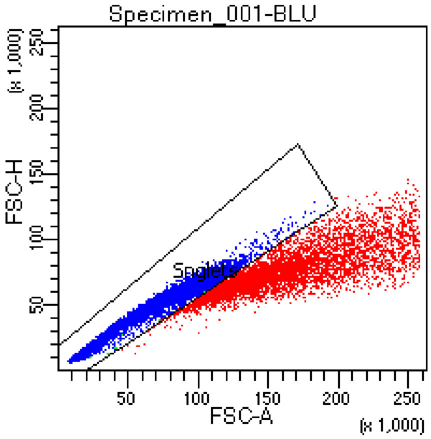

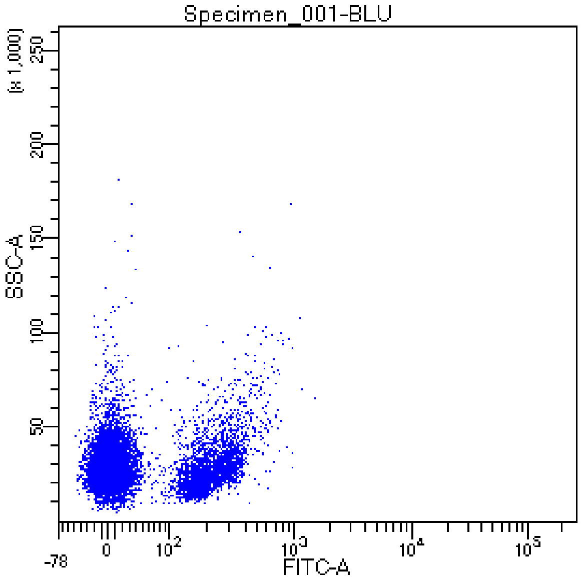

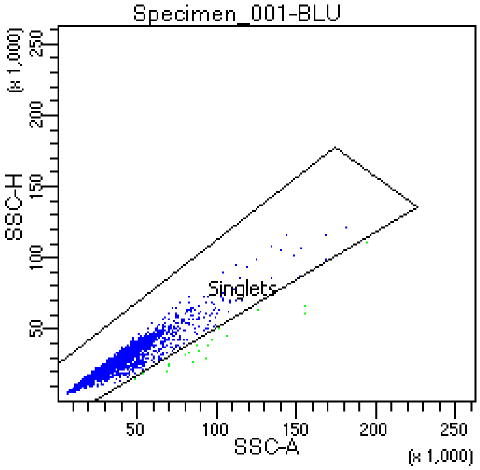

Tube: BLU

Population	#Events	%Parent	%Total
All Events	18,061	####	100.0
Cells	16,058	88.9	88.9
Snglets	10,025	62.4	55.5
Singlets	10,000	99.8	55.4

BD FACSDiva 9.0

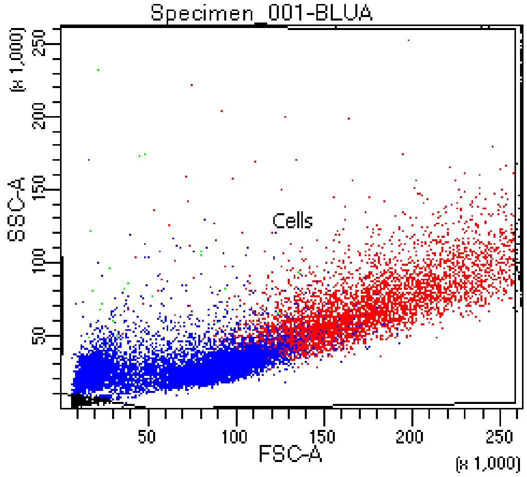

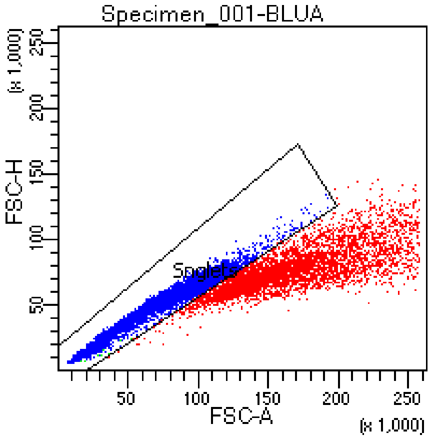

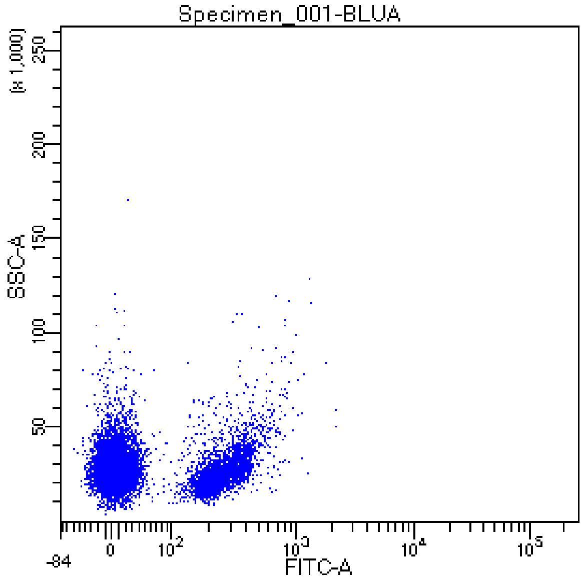

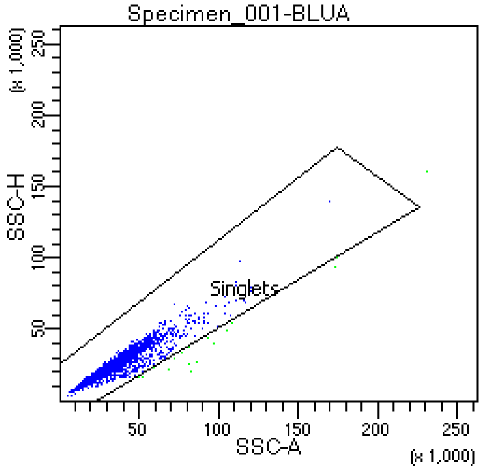

Tube: BLUA

Population	#Events	%Parent	%Total
All Events	16,879	####	100.0
Cells	15,252	90.4	90.4
Snglets	10,022	65.7	59.4
Singlets	10,000	99.8	59.2

BD FACSDiva 9.0

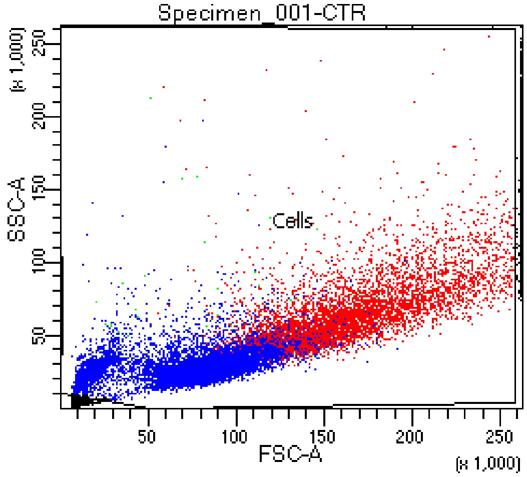

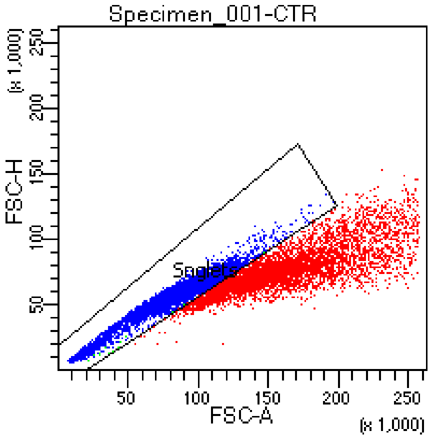

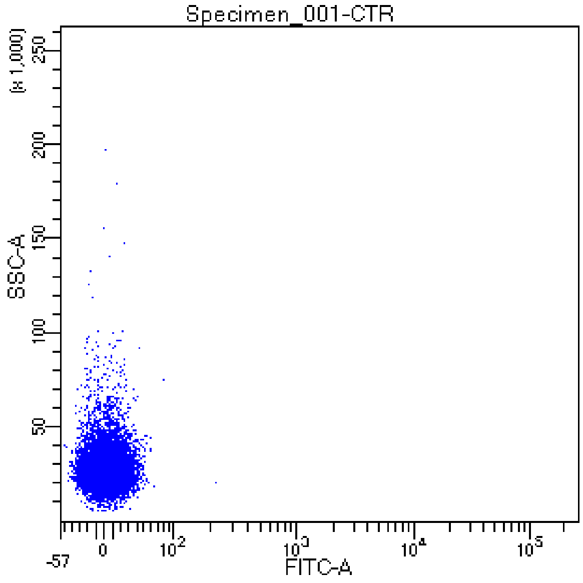

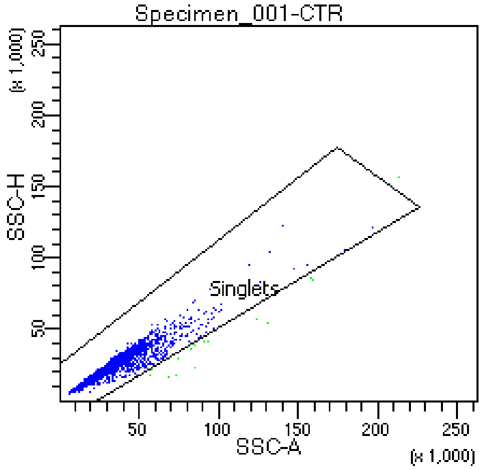

Tube: CTR

Population	#Events	%Parent	%Total
All Events	17,547	####	100.0
Cells	16,135	92.0	92.0
Snglets	10,023	62.1	57.1
Singlets	10,000	99.8	57.0

BD FACSDiva 9.0

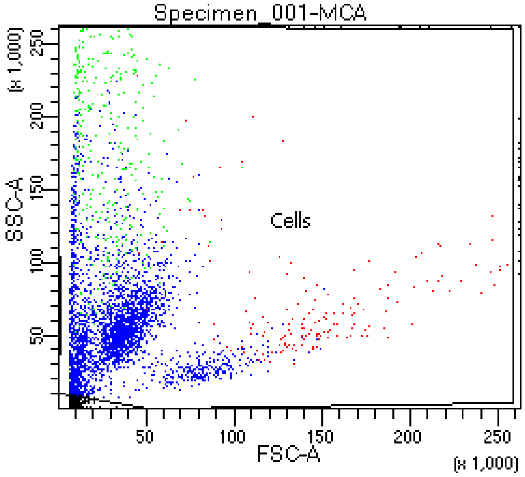

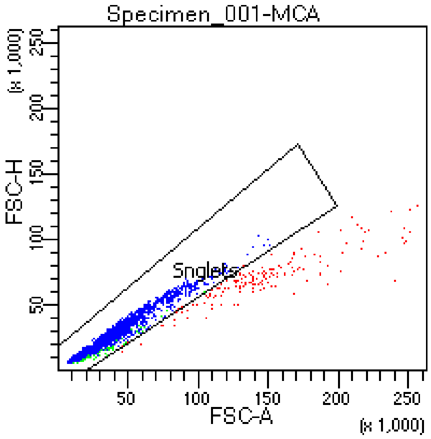

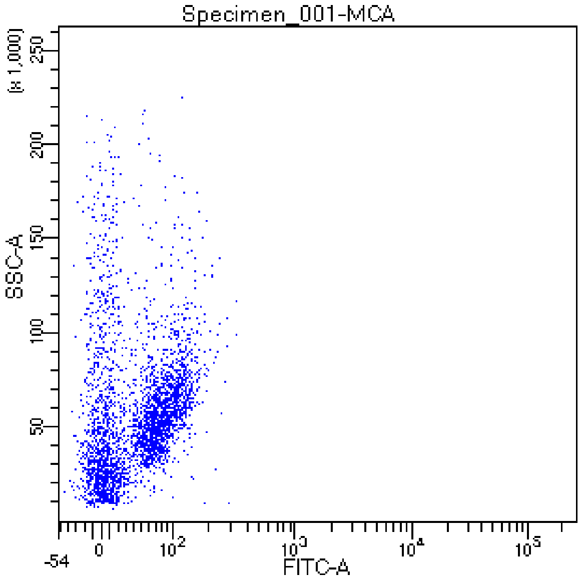

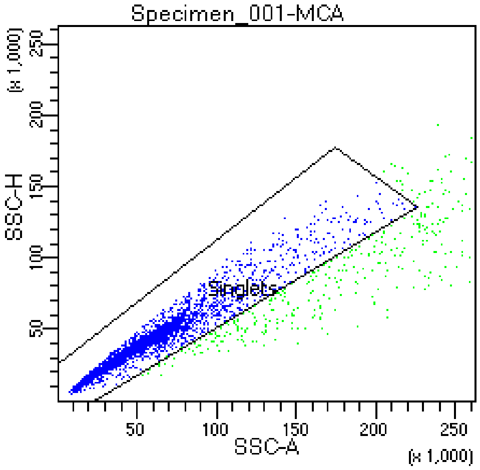

Tube: MCA

Population	#Events	%Parent	%Total
All Events	4,089	####	100.0
Cells	3,512	85.9	85.9
Snglets	3,351	95.4	82.0
Singlets	3,016	90.0	73.8

BD FACSDiva 9.0

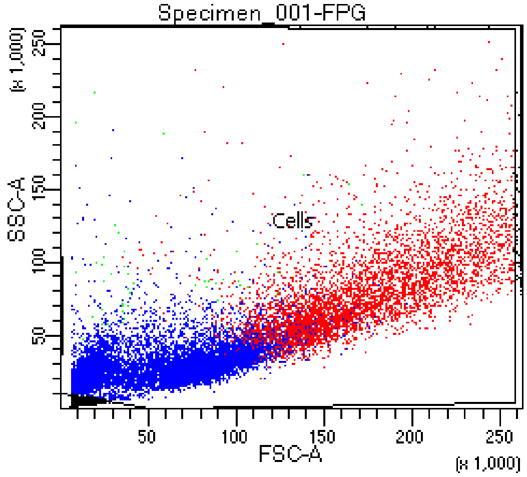

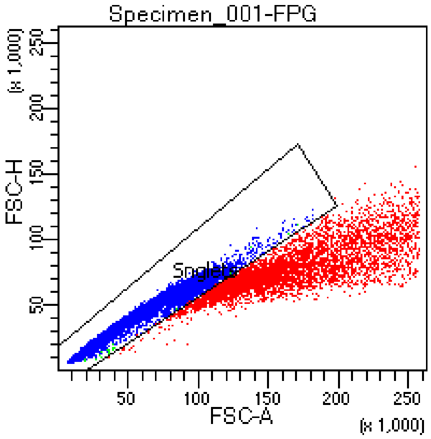

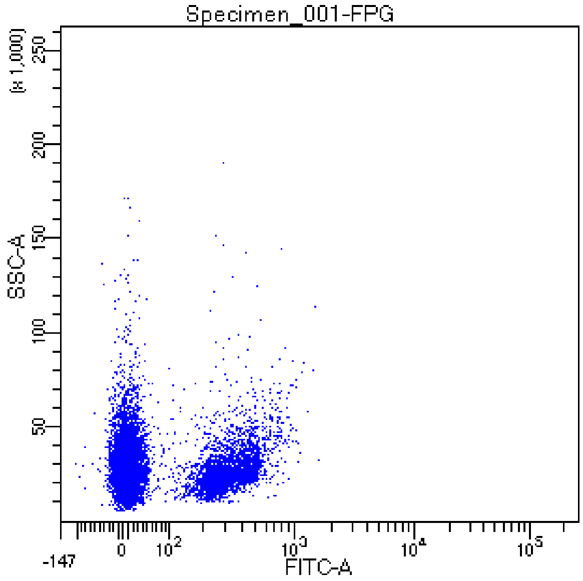

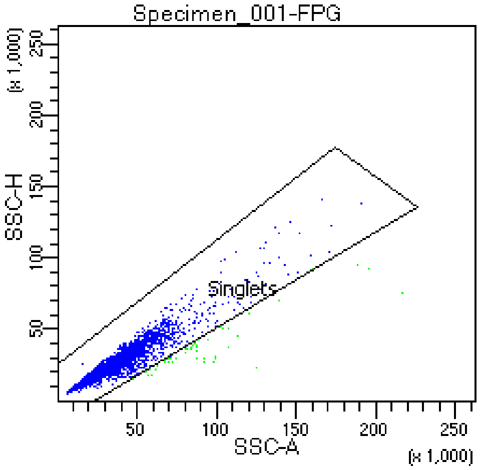

Tube: FPG

Population	#Events	%Parent	%Total
All Events	18,346	####	100.0
Cells	15,758	85.9	85.9
Snglets	10,052	63.8	54.8
Singlets	10,000	99.5	54.5

BD FACSDiva 9.0

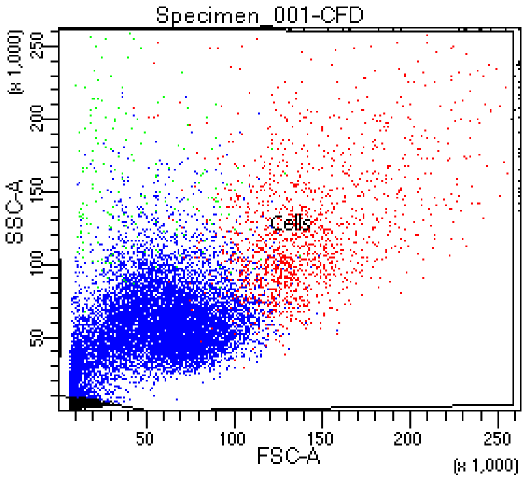

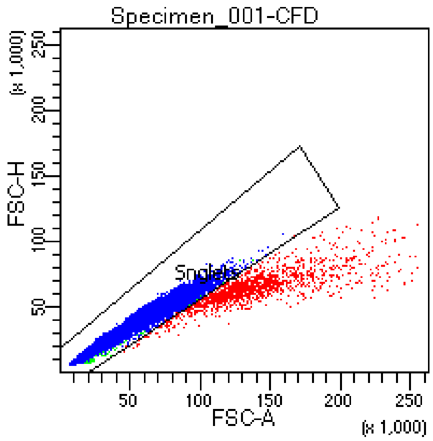

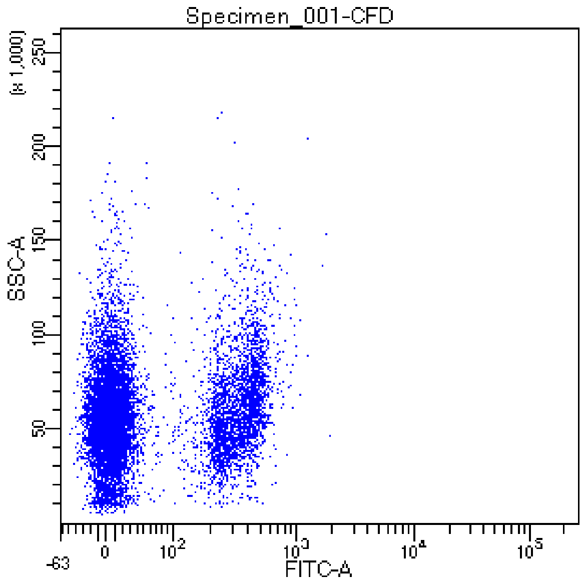

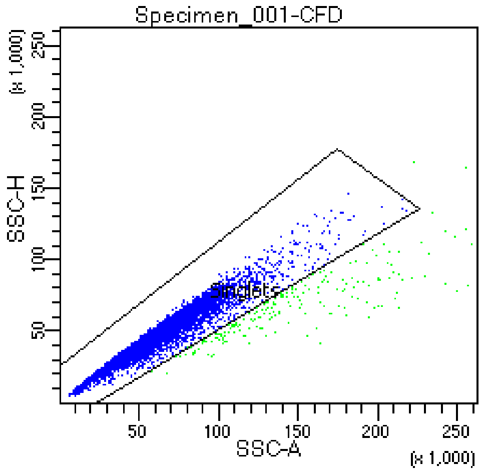

Tube: CFD

Population	#Events	%Parent	%Total
All Events	13,157	####	100.0
Cells	11,758	89.4	89.4
Snglets	10,215	86.9	77.6
Singlets	10,000	97.9	76.0

BD FACSDiva 9.0

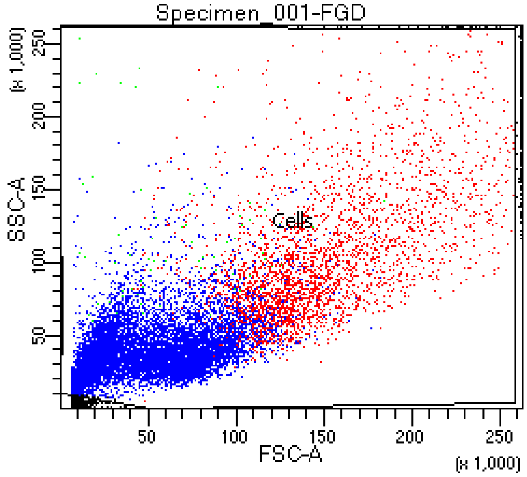

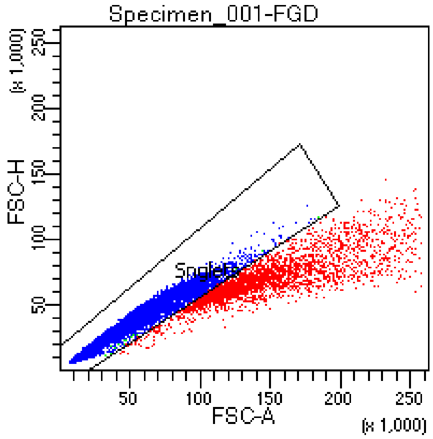

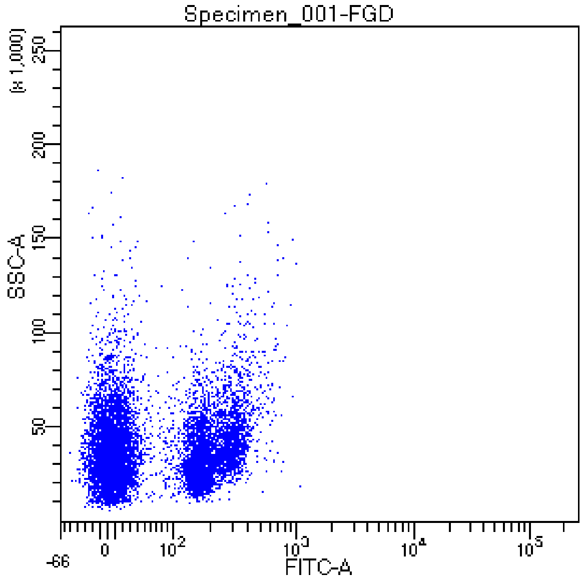

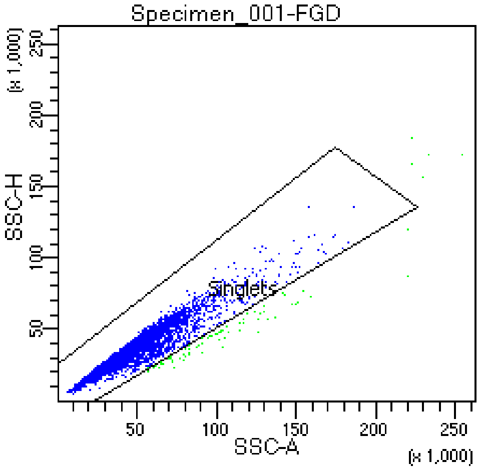

Tube: FGD

Population	#Events	%Parent	%Total
All Events	14,265	####	100.0
Cells	13,077	91.7	91.7
Snglets	10,082	77.1	70.7
Singlets	10,000	99.2	70.1

BD FACSDiva 9.0

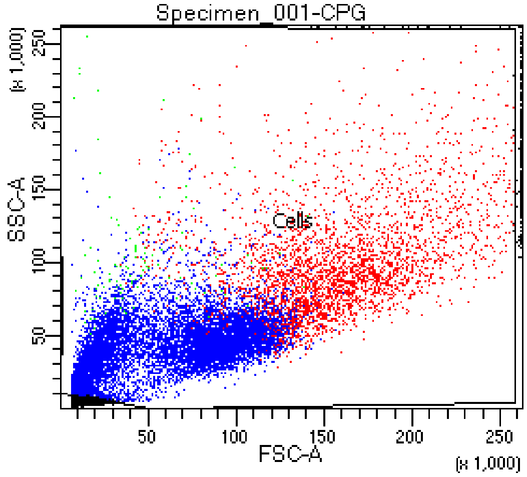

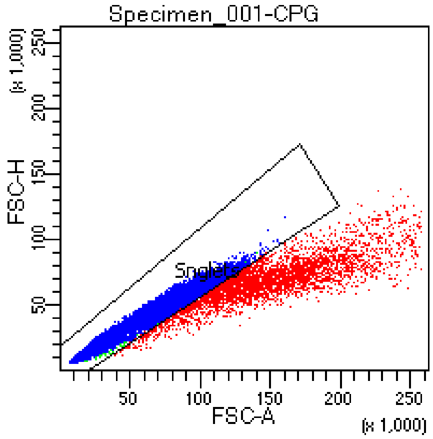

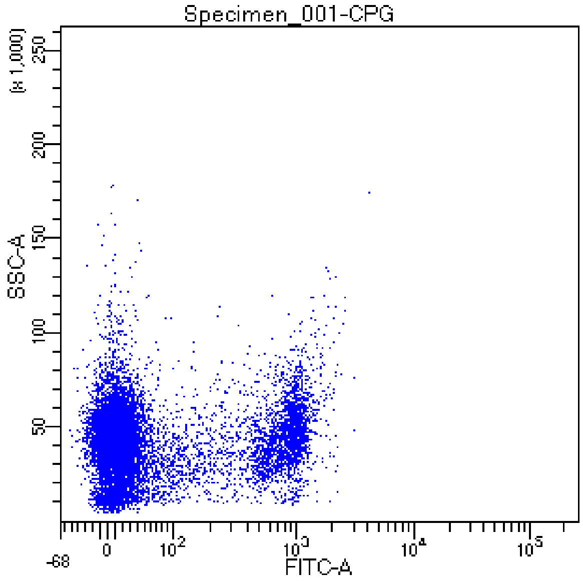

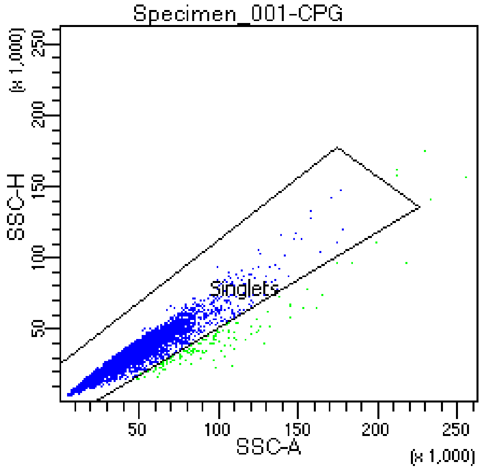

Tube: CPG

Population	#Events	%Parent	%Total
All Events	17,579	####	100.0
Cells	14,034	79.8	79.8
Singlets	10,145	72.3	57.7
Singlets	10,000	98.6	56.9

BD FACSDiva 9.0

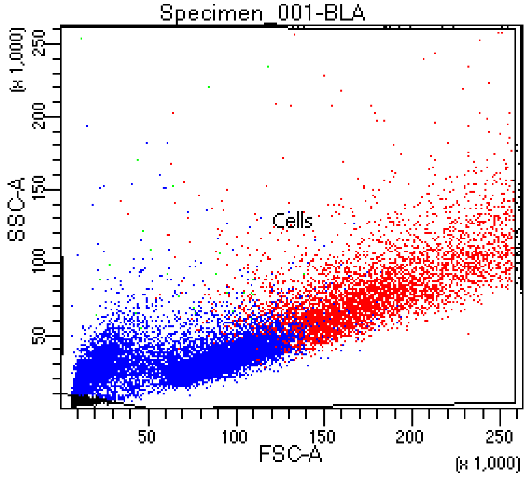

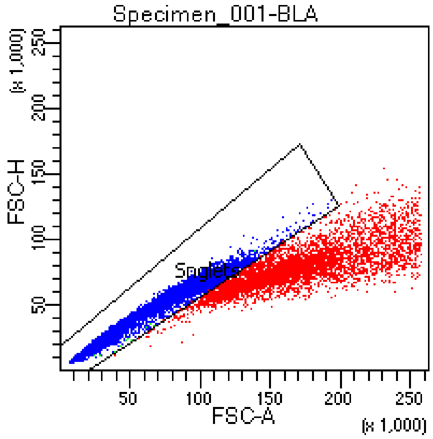

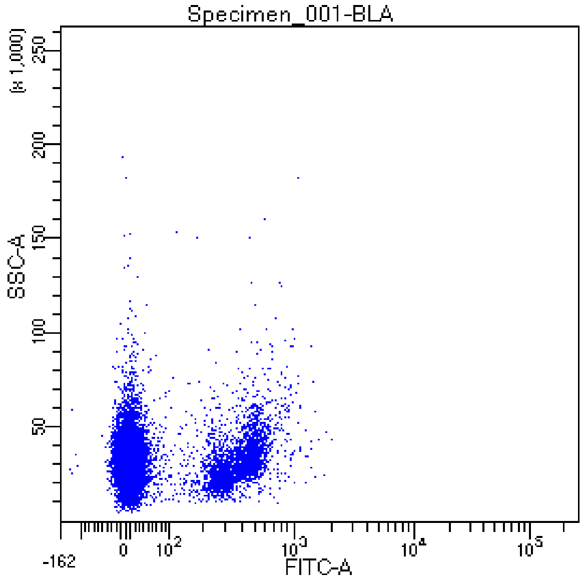

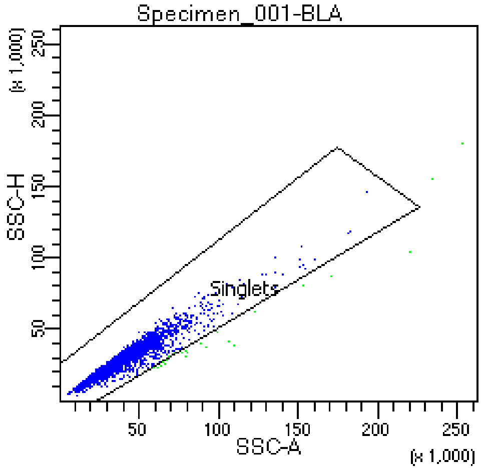

Tube: BLA

Population	#Events	%Parent	%Total
All Events	17,888	####	100.0
Cells	15,408	86.1	86.1
Snglets	10,030	65.1	56.1
Singlets	10,000	99.7	55.9

BD FACSDiva 9.0

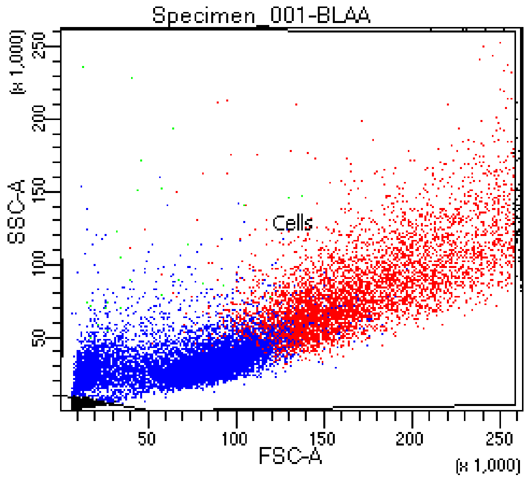

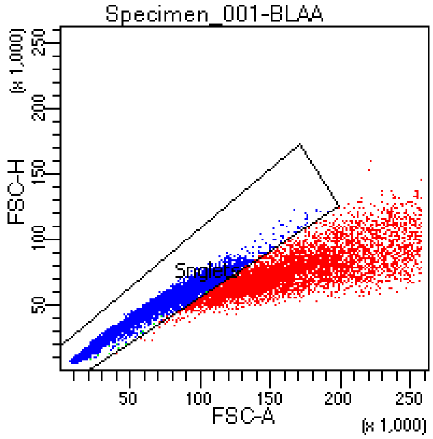

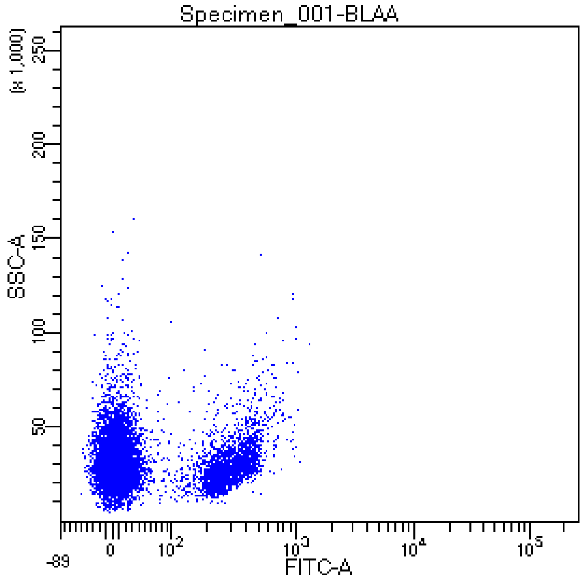

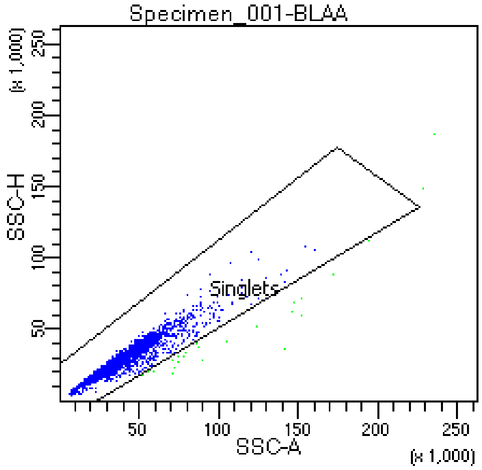

Tube: BLAA

Population	#Events	%Parent	%Total
All Events	18,152	####	100.0
Cells	15,863	87.4	87.4
Snglets	10,039	63.3	55.3
Singlets	10,012	99.7	55.2

BD FACSDiva 9.0

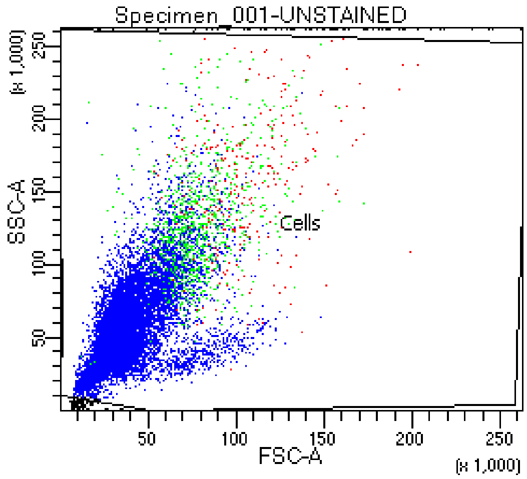

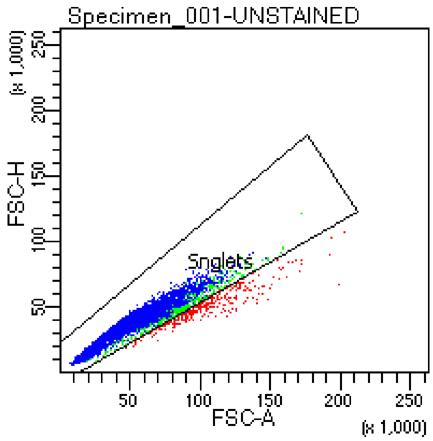

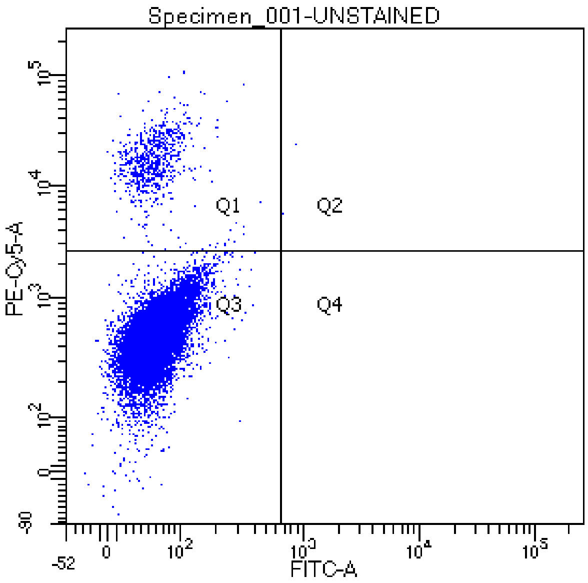

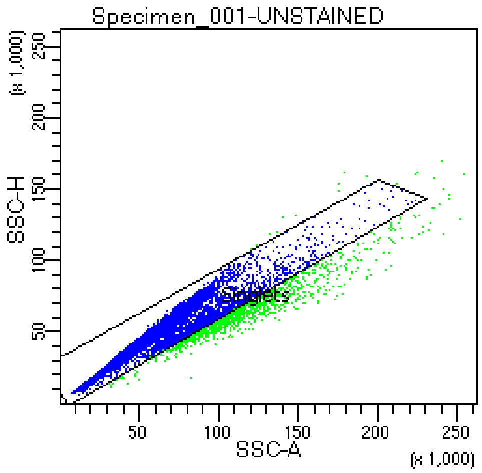

Tube: UNSTAINED

Population	#Events	%Parent	%Total
All Events	15,436	####	100.0
Cells	15,168	98.3	98.3
Snglets	14,848	97.9	96.2
Singlets	13,556	91.3	87.8
Q1	745	5.5	4.8
Q2	2	0.0	0.0
Q3	12,809	94.5	83.0
Q4	0	0.0	0.0

BD FACSDiva 9.0

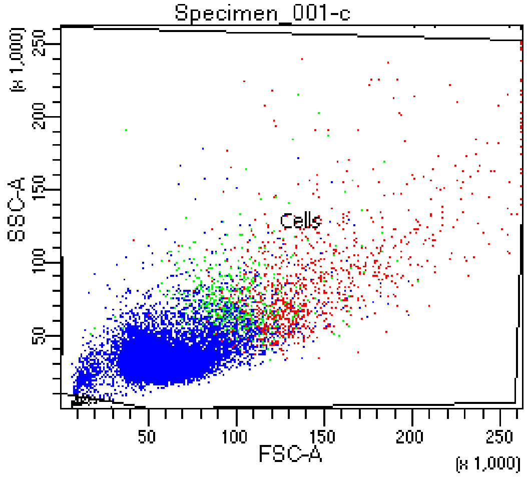

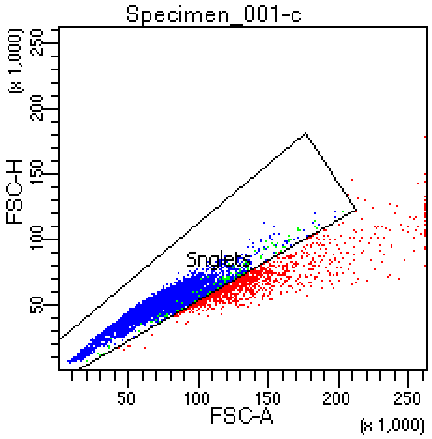

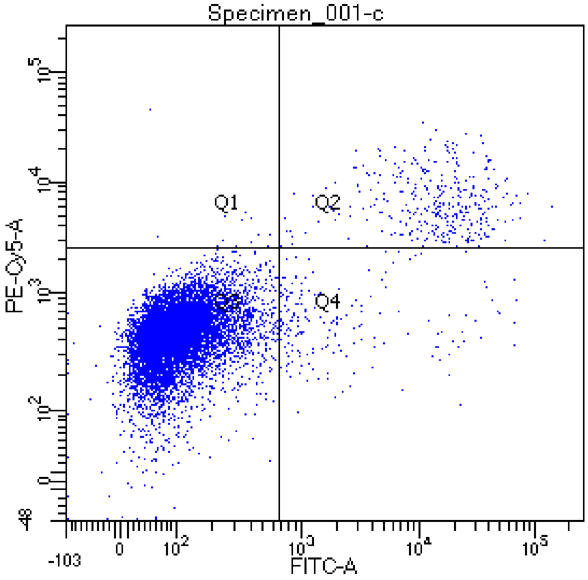

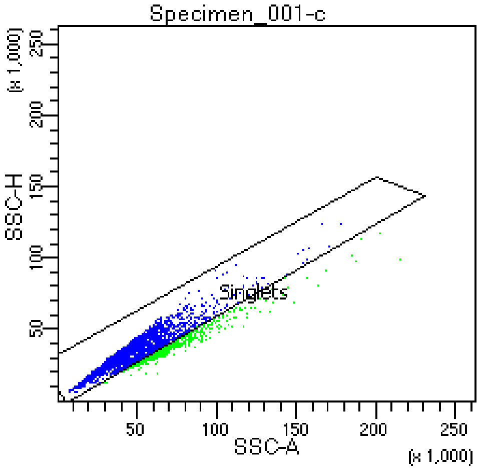

Tube: c

Population	#Events	%Parent	%Total
All Events	11,921	####	100.0
Cells	11,800	99.0	99.0
Snglets	10,650	90.3	89.3
Singlets	10,014	94.0	84.0
Q1	14	0.1	0.1
Q2	354	3.5	3.0
Q3	9,459	94.5	79.3
Q4	187	1.9	1.6

BD FACSDiva 9.0

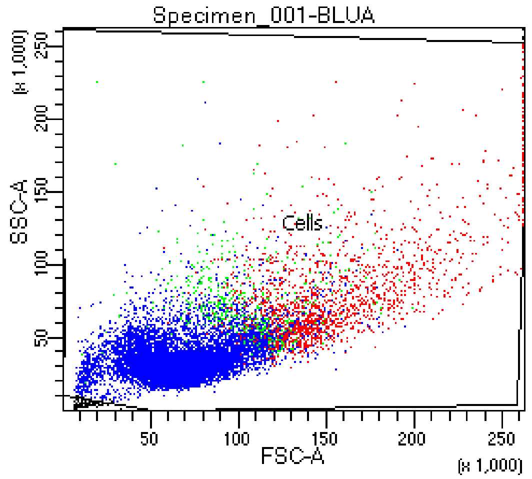

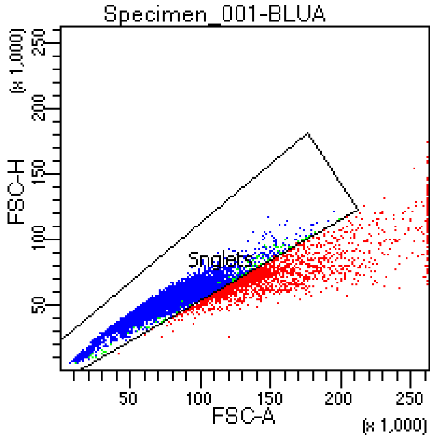

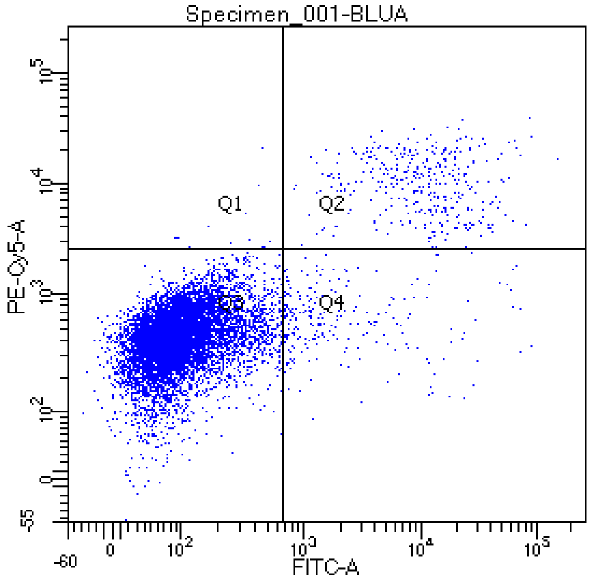

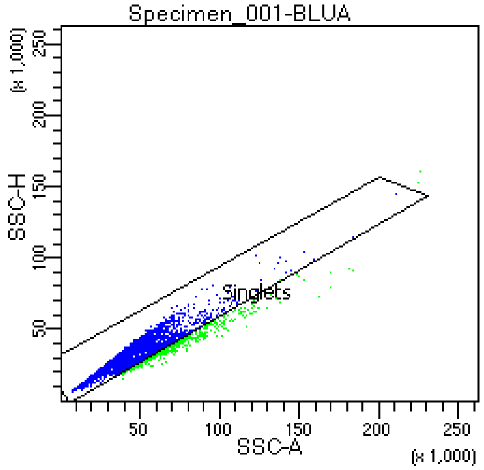

Tube: BLUA

Population	#Events	%Parent	%Total
All Events	12,727	####	100.0
Cells	12,592	98.9	98.9
Snglets	10,618	84.3	83.4
Singlets	10,023	94.4	78.8
Q1	15	0.1	0.1
Q2	362	3.6	2.8
Q3	9,409	93.9	73.9
Q4	237	2.4	1.9

BD FACSDiva 9.0

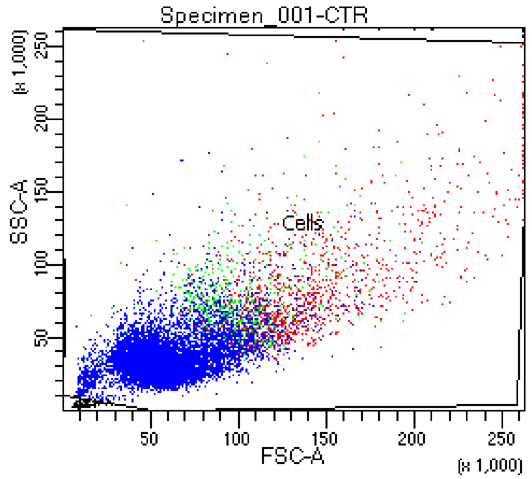

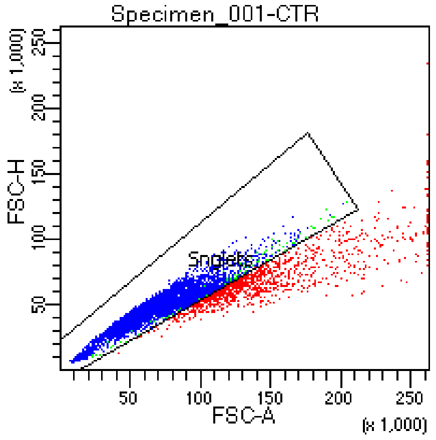

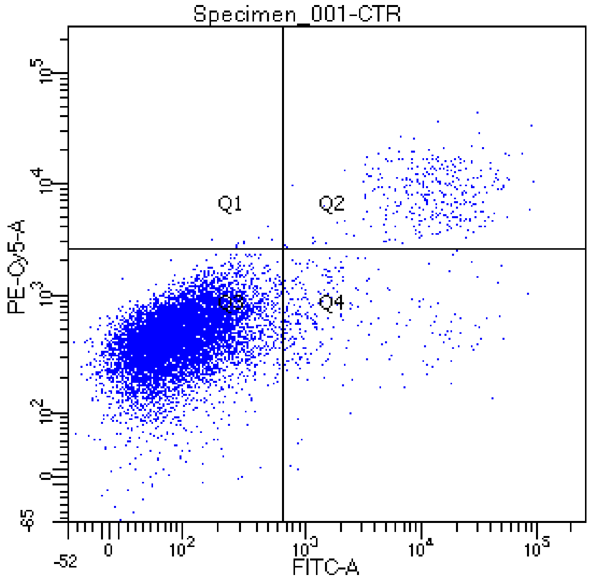

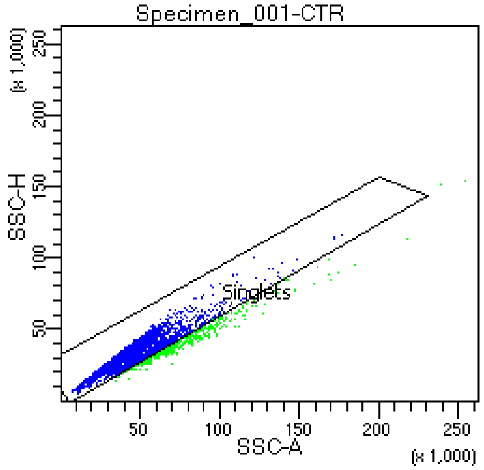

Tube: CTR

Population	#Events	%Parent	%Total
All Events	12,032	####	100.0
Cells	11,884	98.8	98.8
Snglets	10,642	89.5	88.4
Singlets	10,000	94.0	83.1
Q1	12	0.1	0.1
Q2	330	3.3	2.7
Q3	9,383	93.8	78.0
Q4	275	2.8	2.3

BD FACSDiva 9.0

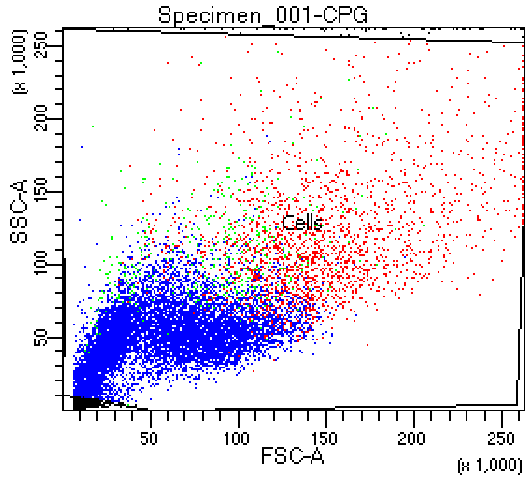

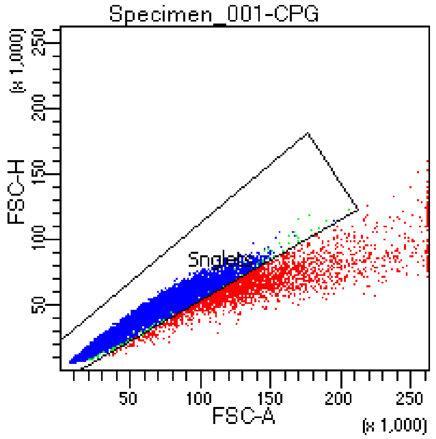

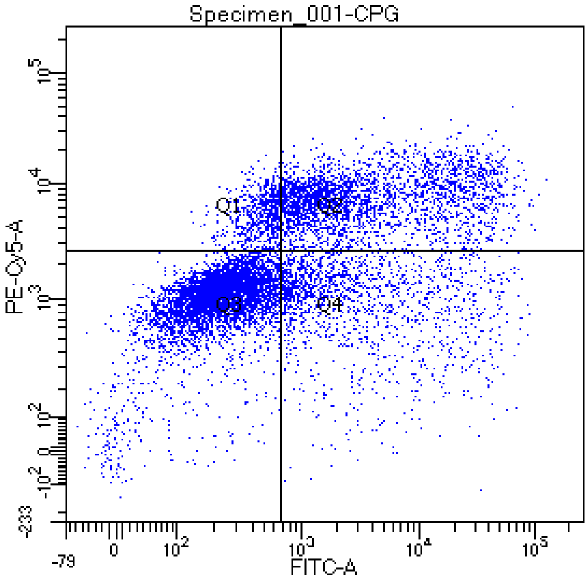

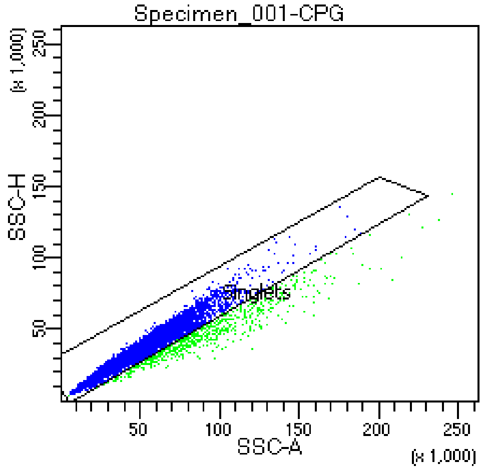

Tube: CPG

Population	#Events	%Parent	%Total
All Events	14,269	####	100.0
Cells	12,943	90.7	90.7
Snglets	10,655	82.3	74.7
Singlets	10,000	93.9	70.1
Q1	682	6.8	4.8
Q2	2,820	28.2	19.8
Q3	4,893	48.9	34.3
Q4	1,605	16.0	11.2

BD FACSDiva 9.0

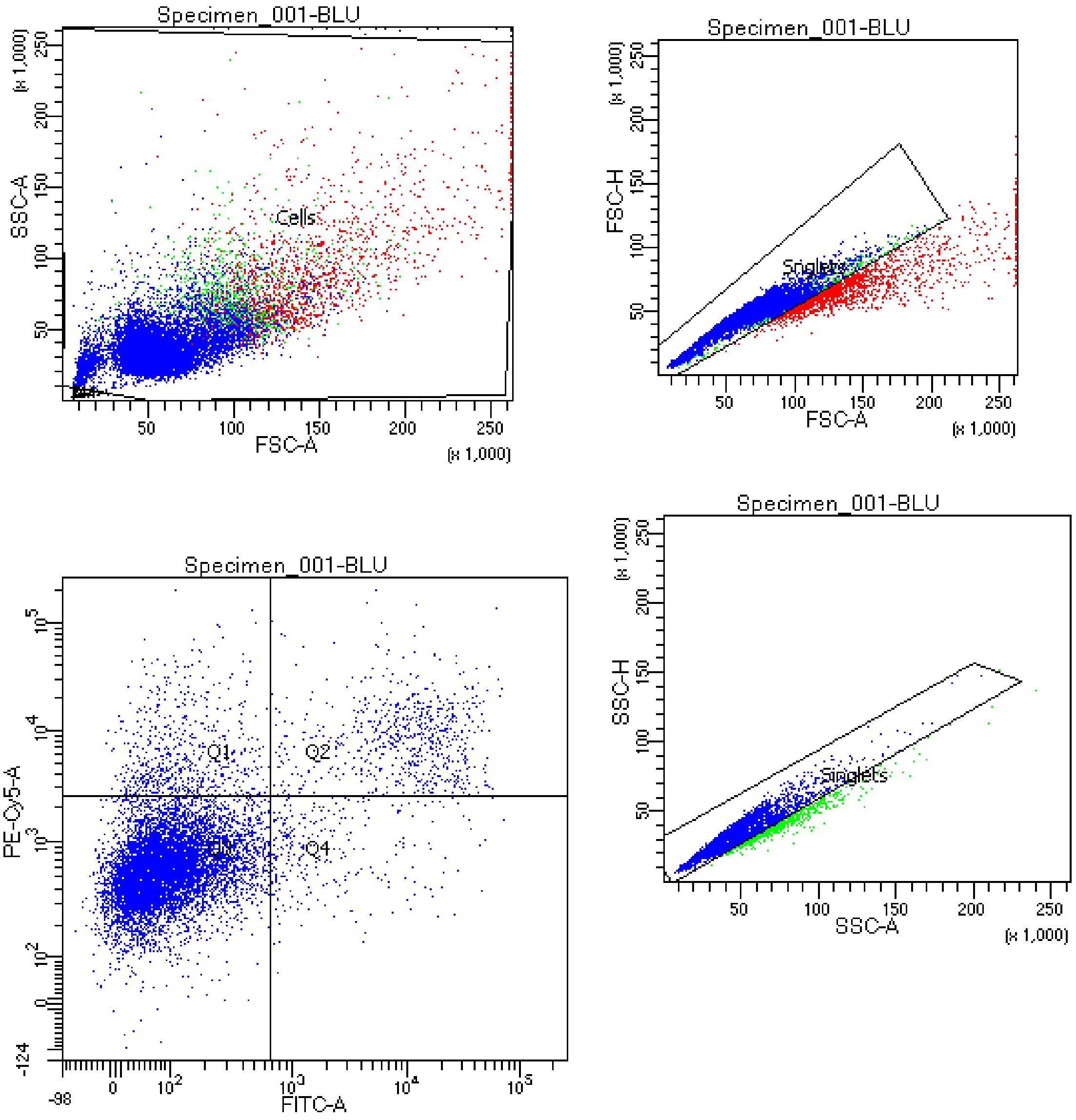

Tube: BLU

Population	#Events	%Parent	%Total
All Events	12,747	####	100.0
Cells	12,494	98.0	98.0
Snglets	10,820	86.6	84.9
Singlets	10,000	92.4	78.4
Q1	616	6.2	4.8
Q2	671	6.7	5.3
Q3	8,433	84.3	66.2
Q4	280	2.8	2.2

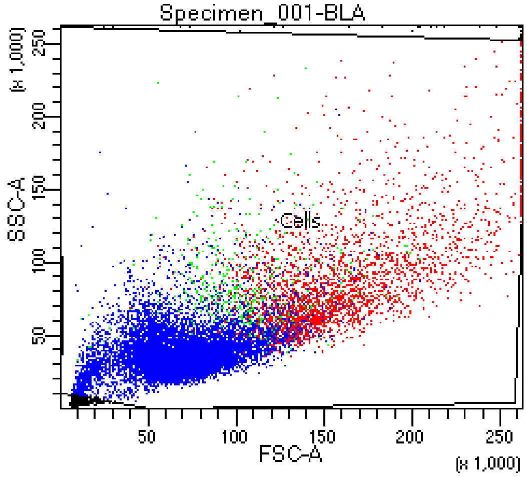

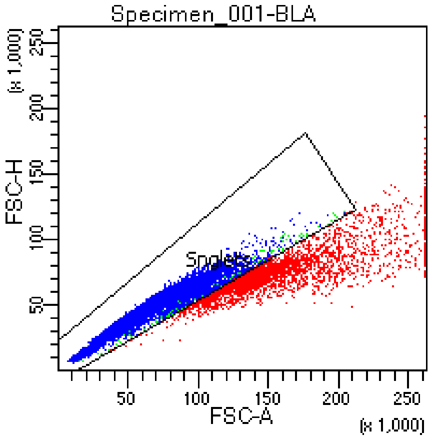

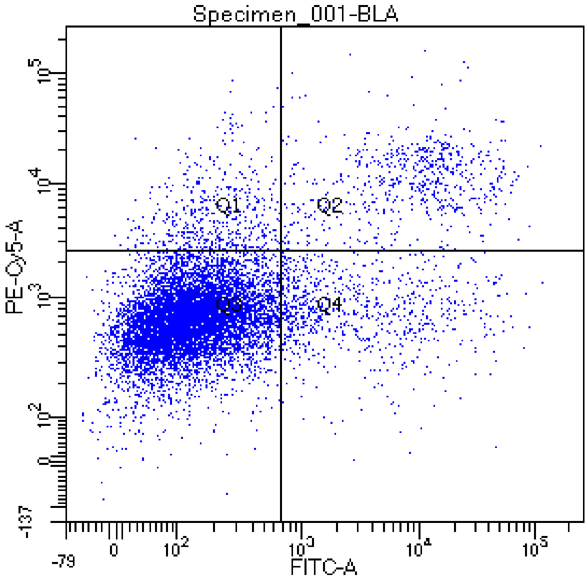

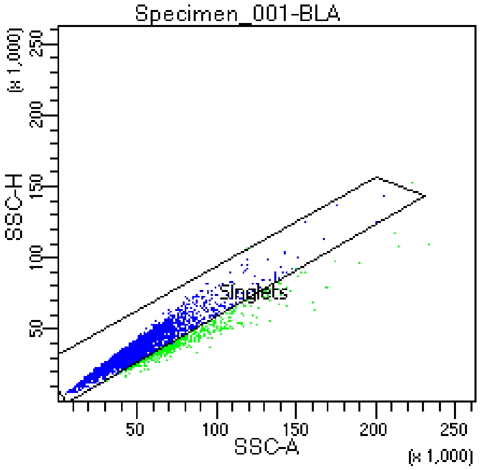

Tube: BLA

Population	#Events	%Parent	%Total
All Events	14,091	####	100.0
Cells	13,669	97.0	97.0
Snglets	10,497	76.8	74.5
Singlets	10,000	95.3	71.0
Q1	494	4.9	3.5
Q2	627	6.3	4.4
Q3	8,068	80.7	57.3
Q4	811	8.1	5.8

BD FACSDiva 9.0

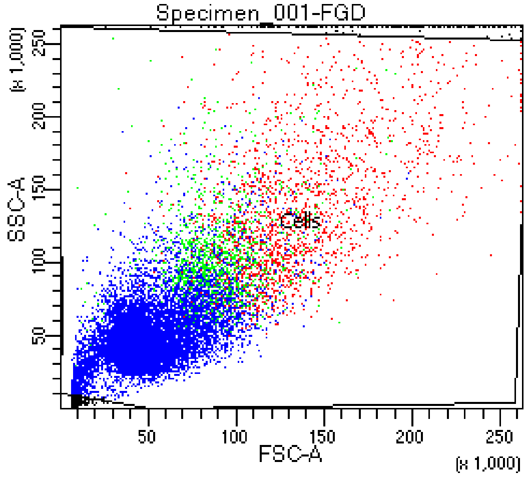

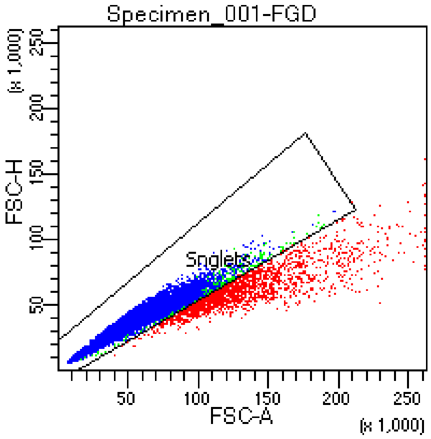

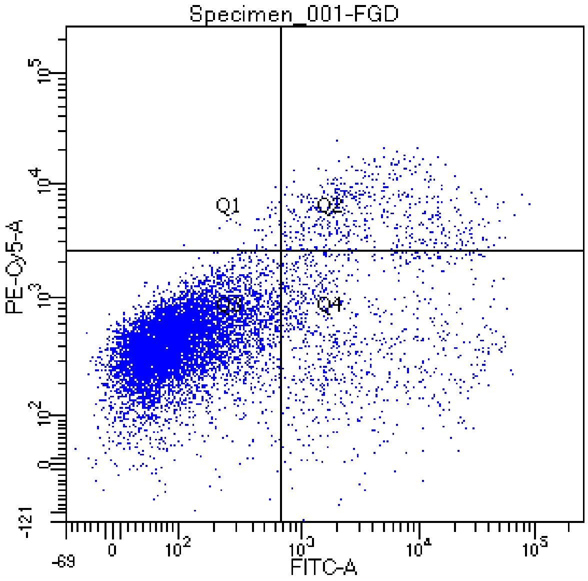

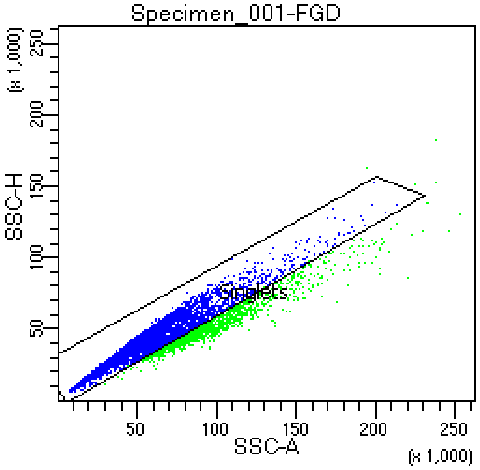

Tube: FGD

Population	#Events	%Parent	%Total
All Events	13,854	####	100.0
Cells	13,406	96.8	96.8
Snglets	11,468	85.5	82.8
Singlets	10,000	87.2	72.2
Q1	38	0.4	0.3
Q2	547	5.5	3.9
Q3	8,500	85.0	61.4
Q4	915	9.2	6.6

BD FACSDiva 9.0

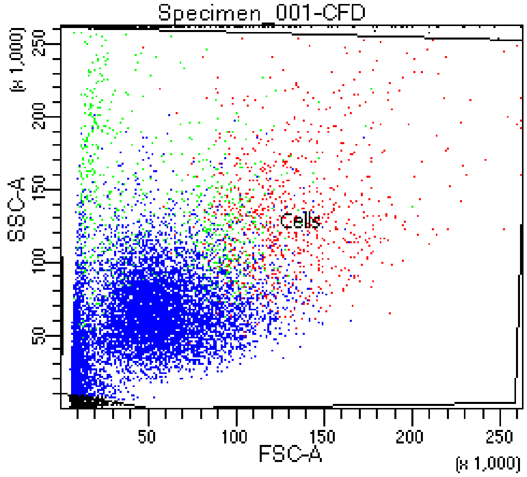

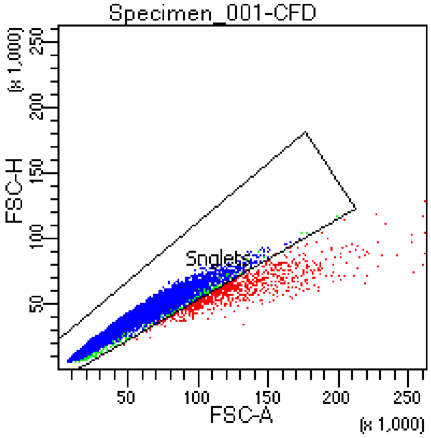

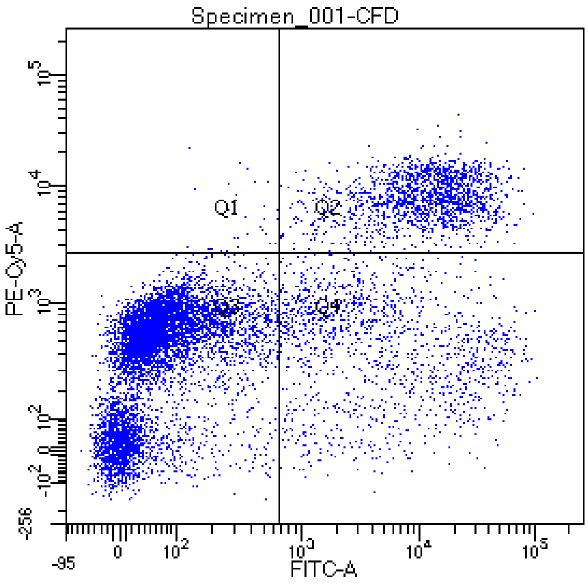

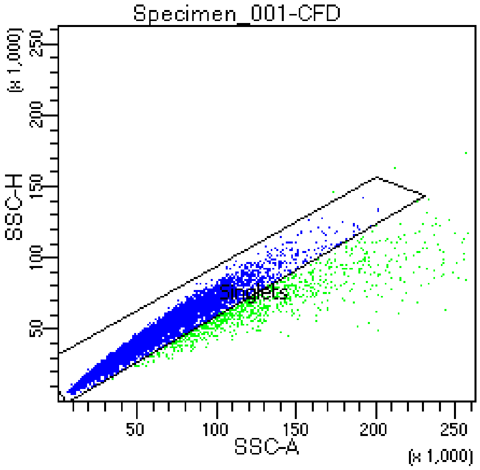

Tube: CFD

Population	#Events	%Parent	%Total
All Events	13,056	####	100.0
Cells	11,696	89.6	89.6
Snglets	10,855	92.8	83.1
Singlets	10,000	92.1	76.6
Q1	34	0.3	0.3
Q2	1,701	17.0	13.0
Q3	6,888	68.9	52.8
Q4	1,377	13.8	10.5

BD FACSDiva 9.0

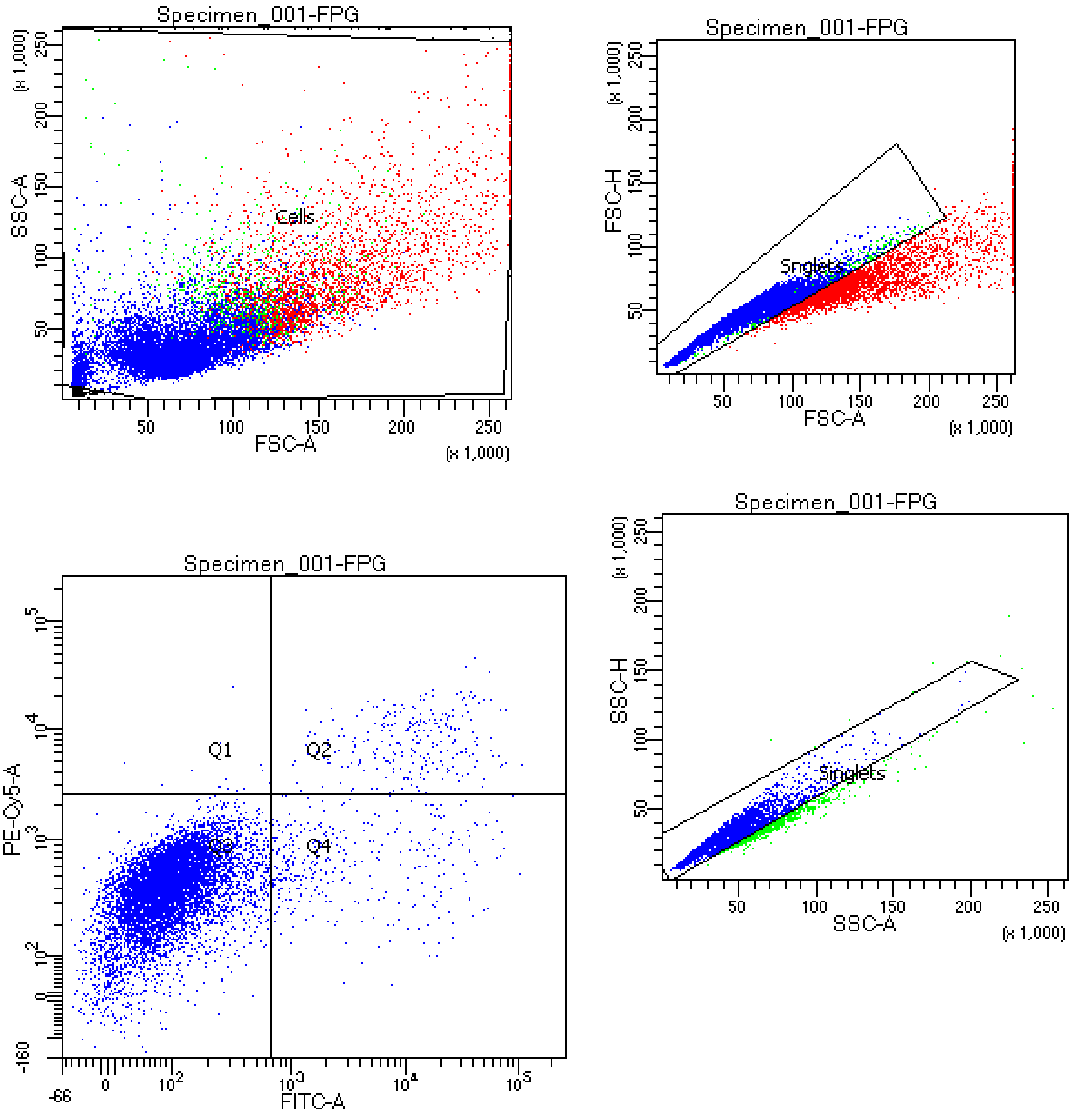

Tube: FPG

Population	#Events	%Parent	%Total
All Events	14,974	####	100.0
Cells	14,575	97.3	97.3
Singlets	10,893	74.7	72.7
Singlets	10,000	91.8	66.8
Q1	15	0.2	0.1
Q2	264	2.6	1.8
Q3	9,364	93.6	62.5
Q4	357	3.6	2.4

BD FACSDiva 9.0

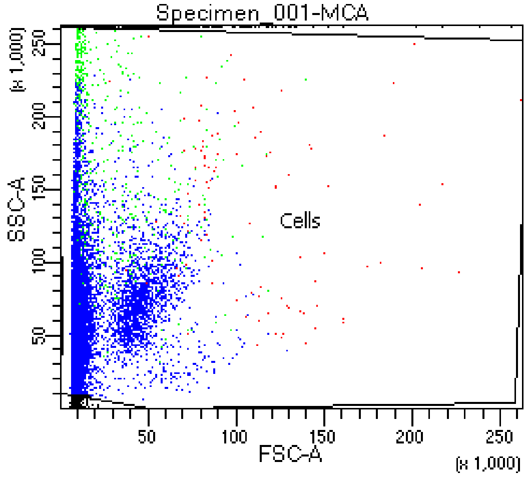

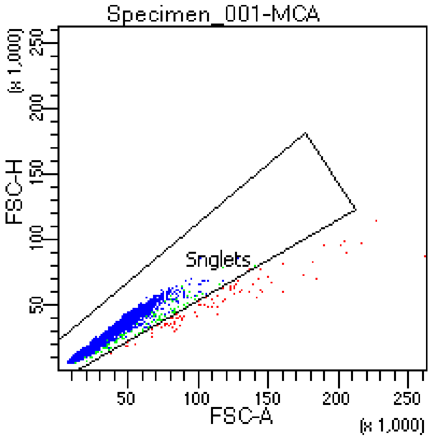

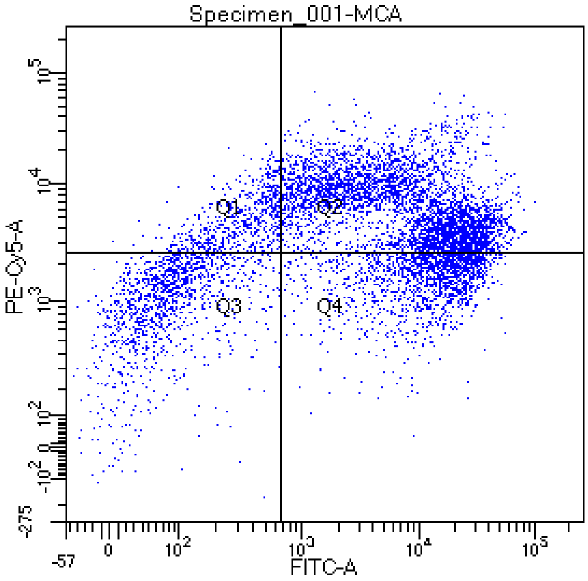

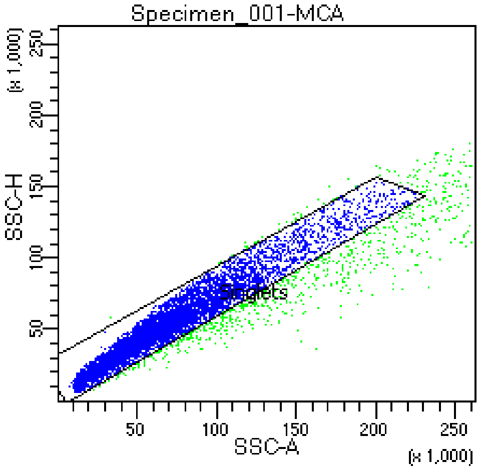

Tube: MCA

Population	#Events	%Parent	%Total
All Events	9,488	####	100.0
Cells	8,226	86.7	86.7
Snglets	8,126	98.8	85.6
Singlets	7,349	90.4	77.5
Q1	625	8.5	6.6
Q2	3,912	53.2	41.2
Q3	1,248	17.0	13.2
Q4	1,564	21.3	16.5

BD FACSDiva 9.0

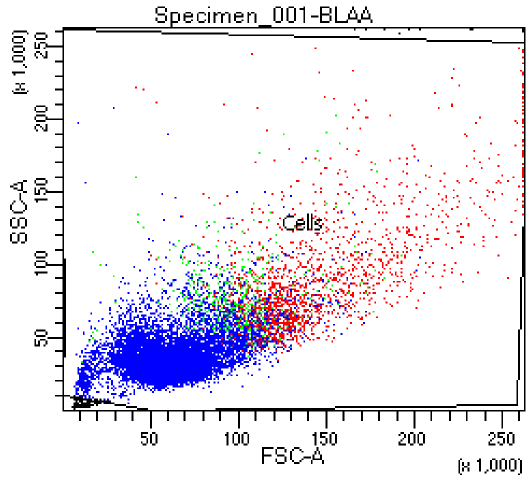

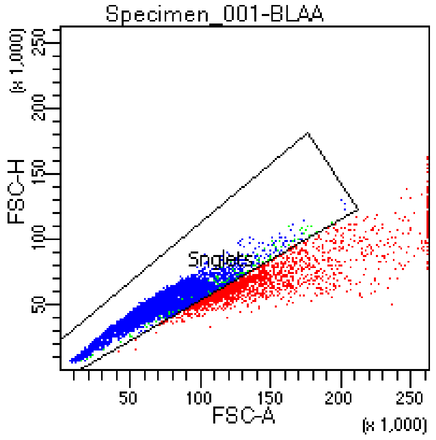

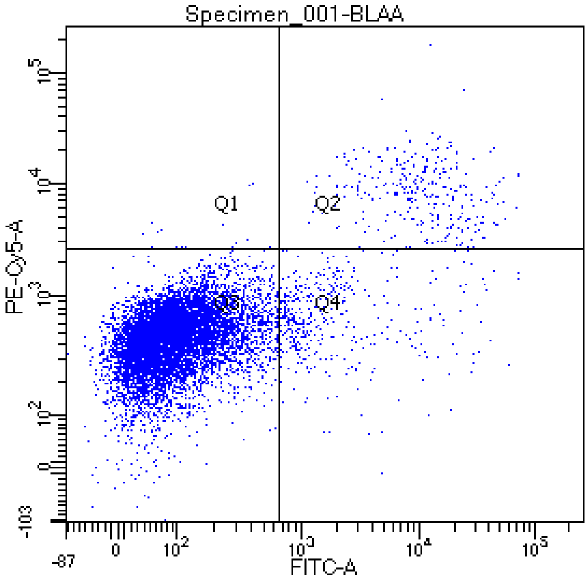

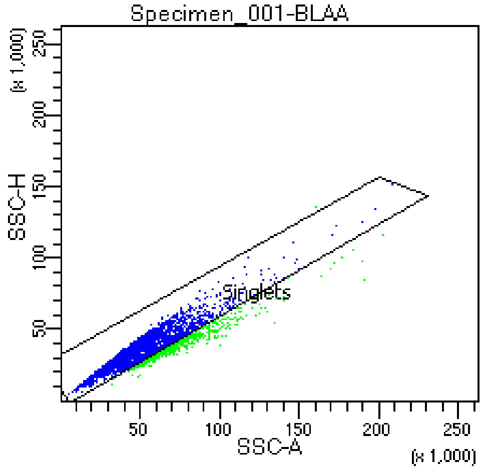

Tube: BLAA

Population	#Events	%Parent	%Total
All Events	12,713	####	100.0
Cells	12,527	98.5	98.5
Singlets	10,620	84.8	83.5
Singlets	10,000	94.2	78.7
Q1	17	0.2	0.1
Q2	305	3.0	2.4
Q3	9,278	92.8	73.0
Q4	400	4.0	3.1

BD FACSDiva 9.0

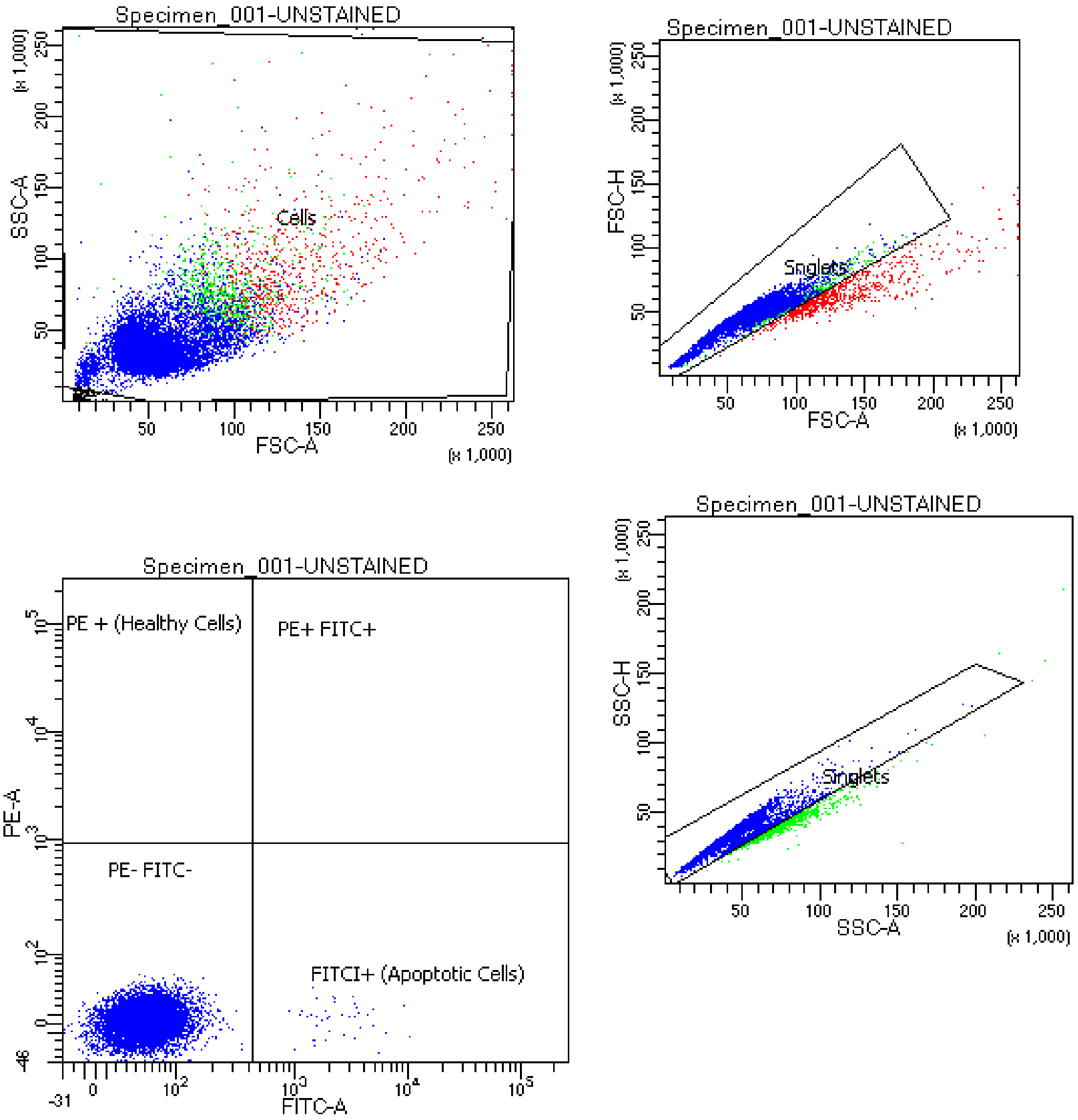

Tube: UNSTAINED

Population	#Events	%Parent	%Total
All Events	11,890	####	100.0
Cells	11,677	98.2	98.2
Singlets	10,894	93.3	91.6
Singlets	10,113	92.8	85.1
PE + (Healthy Cells)	0	0.0	0.0
PE+ FITC+	0	0.0	0.0
PE- FITC-	10,074	99.6	84.7
FITCI+ (Apoptotic Cell	39	0.4	0.3

BD FACSDiva 9.0

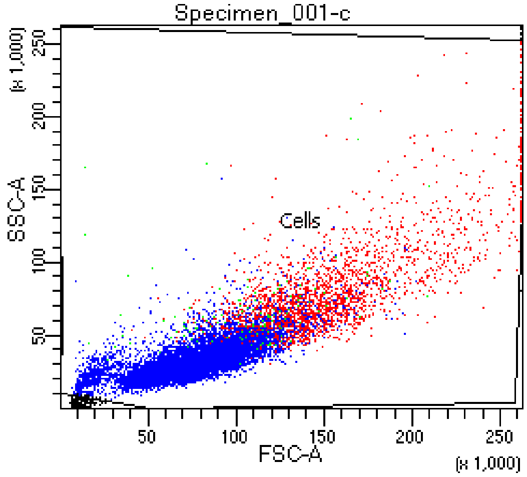

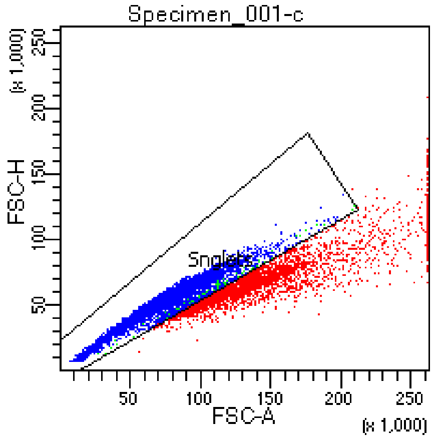

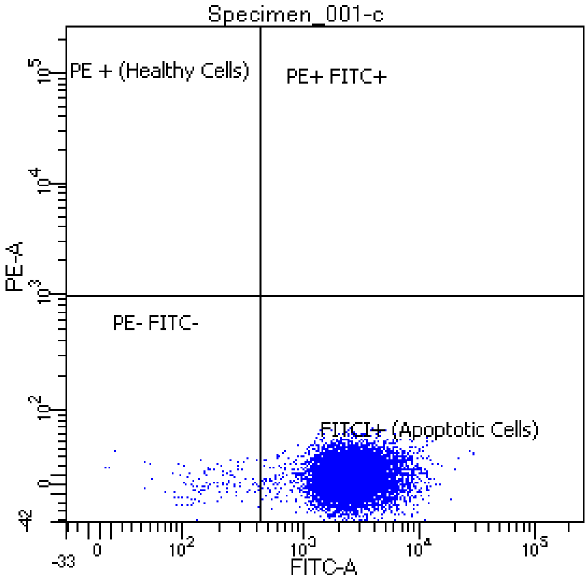

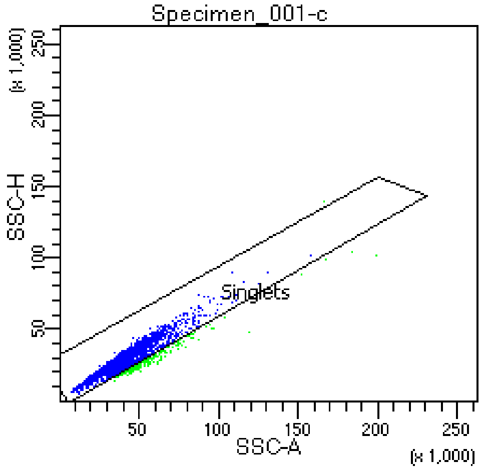

Tube: c

Population	#Events	%Parent	%Total
All Events	13,628	####	100.0
Cells	13,377	98.2	98.2
Snglets	10,326	77.2	75.8
Singlets	9,977	96.6	73.2
PE + (Healthy Cells)	0	0.0	0.0
PE+ FITC+	0	0.0	0.0
PE- FITC-	109	1.1	0.8
FITCI+ (Apoptotic Cell	9,868	98.9	72.4

BD FACSDiva 9.0

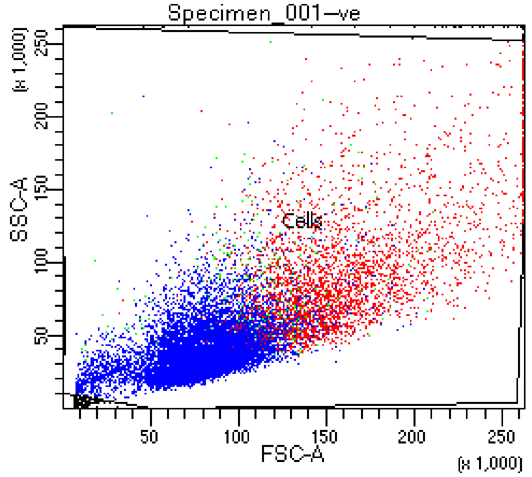

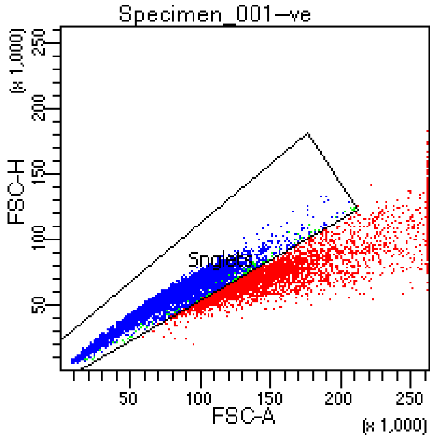

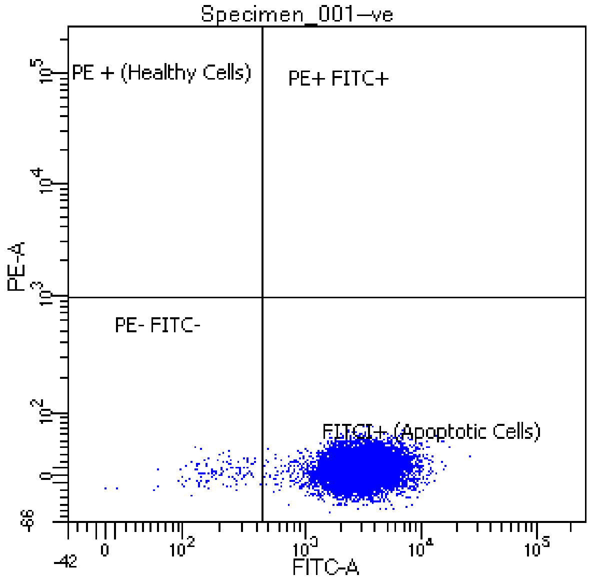

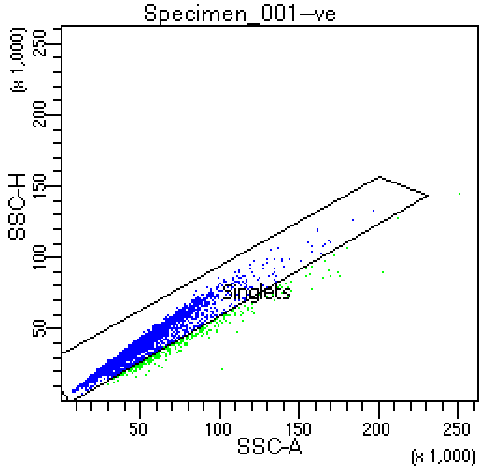

Tube: -ve

Population	#Events	%Parent	%Total
All Events	14,422	####	100.0
Cells	14,137	98.0	98.0
Singlets	10,376	73.4	71.9
Singlets	10,012	96.5	69.4
PE + (Healthy Cells)	0	0.0	0.0
PE+ FITC+	0	0.0	0.0
PE- FITC-	111	1.1	0.8
FITCI+ (Apoptotic Cell	9,901	98.9	68.7

BD FACSDiva 9.0

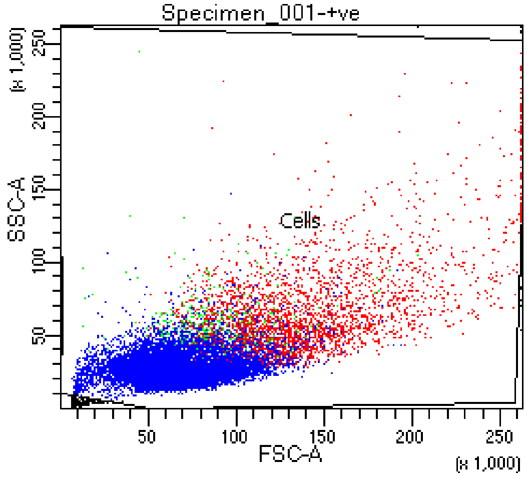

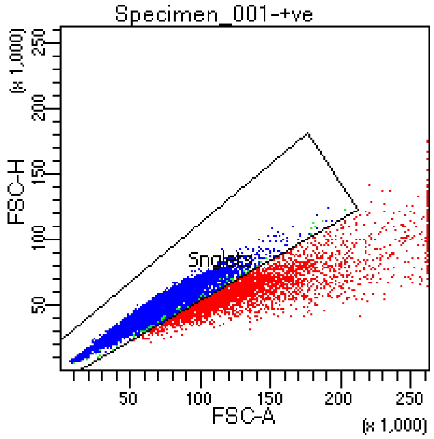

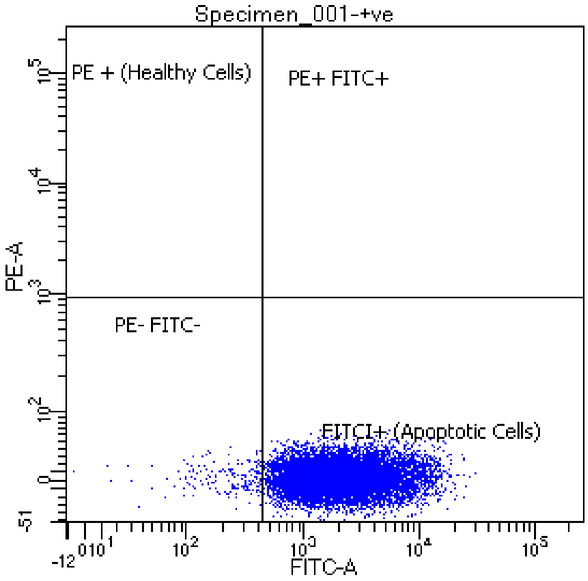

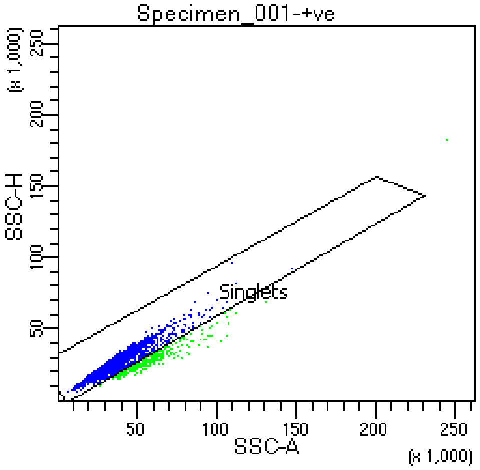

Tube: +ve

Population	#Events	%Parent	%Total
All Events	13,881	####	100.0
Cells	13,662	98.4	98.4
Singlets	10,380	76.0	74.8
Singlets	9,954	95.9	71.7
PE + (Healthy Cells)	0	0.0	0.0
PE+ FITC+	0	0.0	0.0
PE- FITC-	202	2.0	1.5
FITCI+ (Apoptotic Cell	9,752	98.0	70.3

BD FACSDiva 9.0

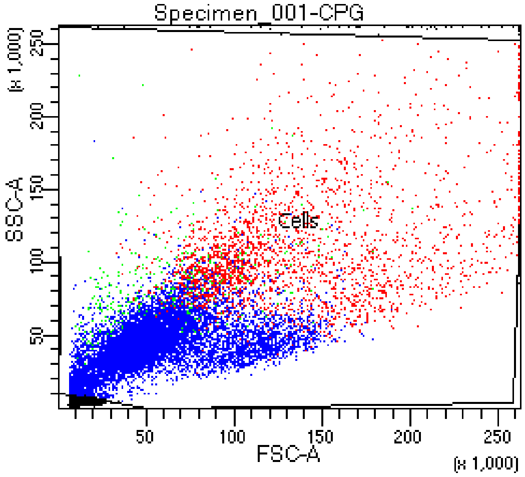

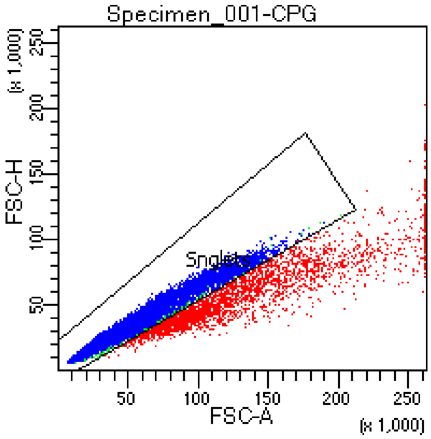

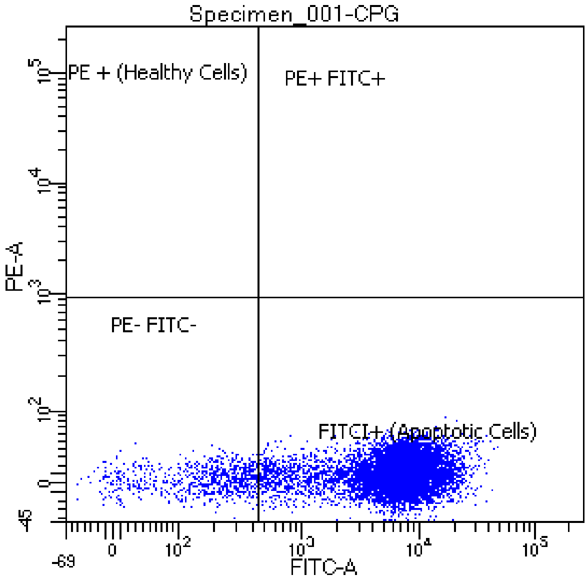

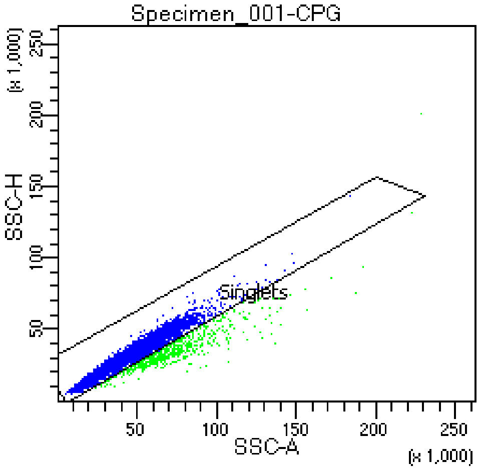

Tube: CPG

Population	#Events	%Parent	%Total
All Events	15,254	####	100.0
Cells	13,519	88.6	88.6
Snglets	10,669	78.9	69.9
Singlets	10,045	94.2	65.9
PE + (Healthy Cells)	0	0.0	0.0
PE+ FITC+	0	0.0	0.0
PE- FITC-	775	7.7	5.1
FITCI+ (Apoptotic Cell	9,270	92.3	60.8

BD FACSDiva 9.0

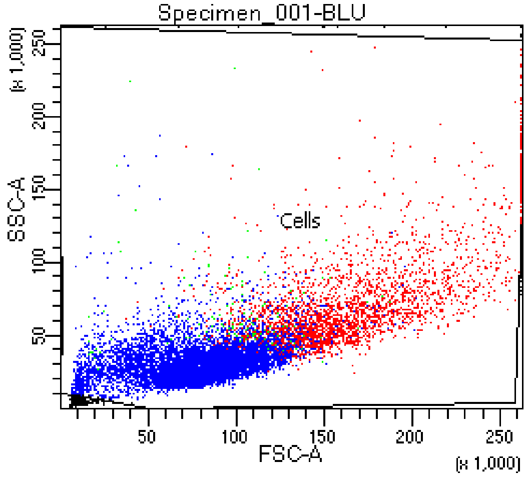

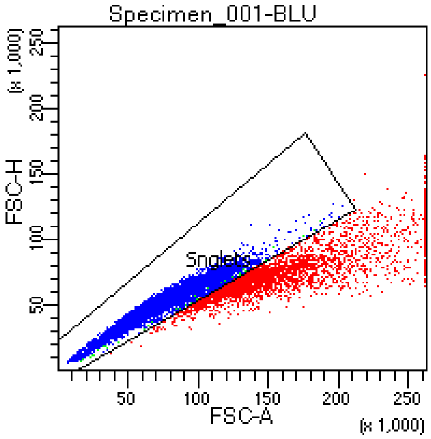

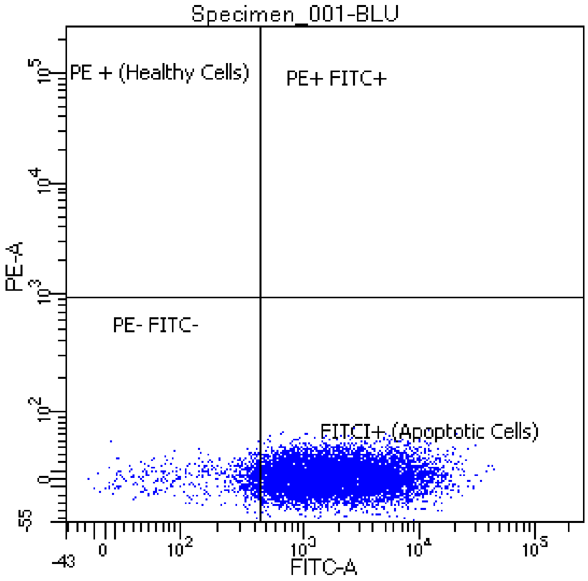

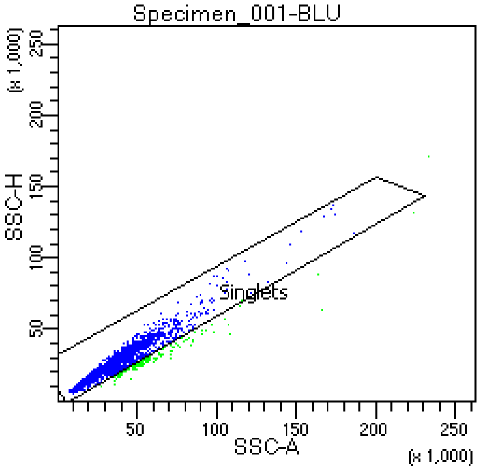

Tube: BLU

Population	#Events	%Parent	%Total
All Events	13,799	####	100.0
Cells	13,389	97.0	97.0
Singlets	10,185	76.1	73.8
Singlets	9,992	98.1	72.4
PE + (Healthy Cells)	0	0.0	0.0
PE+ FITC+	0	0.0	0.0
PE- FITC-	628	6.3	4.6
FITCI+ (Apoptotic Cell	9,364	93.7	67.9

BD FACSDiva 9.0

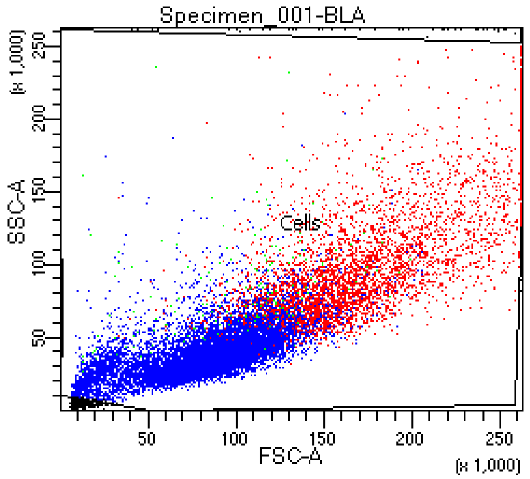

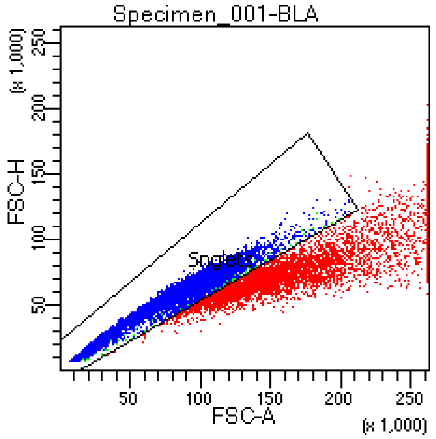

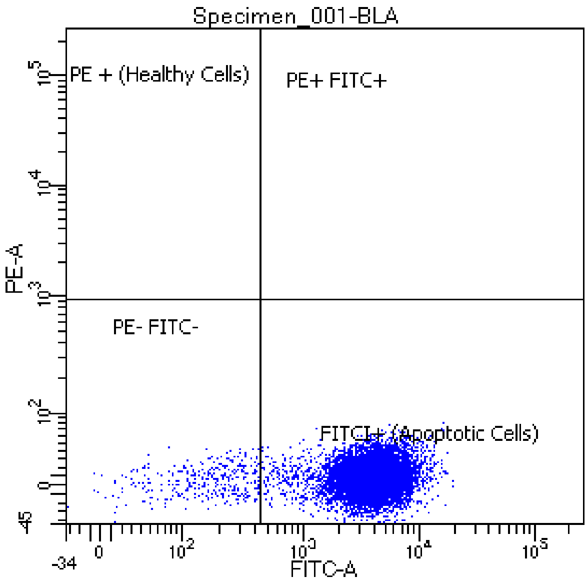

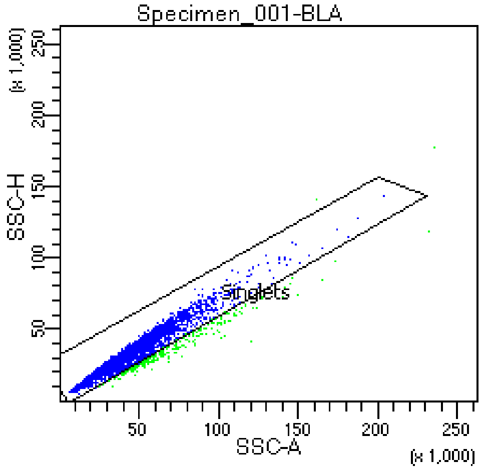

Tube: BLA

Population	#Events	%Parent	%Total
All Events	16,381	####	100.0
Cells	15,568	95.0	95.0
Singlets	10,322	66.3	63.0
Singlets	9,994	96.8	61.0
PE + (Healthy Cells)	0	0.0	0.0
PE+ FITC+	0	0.0	0.0
PE- FITC-	342	3.4	2.1
FITCI+ (Apoptotic Cell	9,652	96.6	58.9

BD FACSDiva 9.0

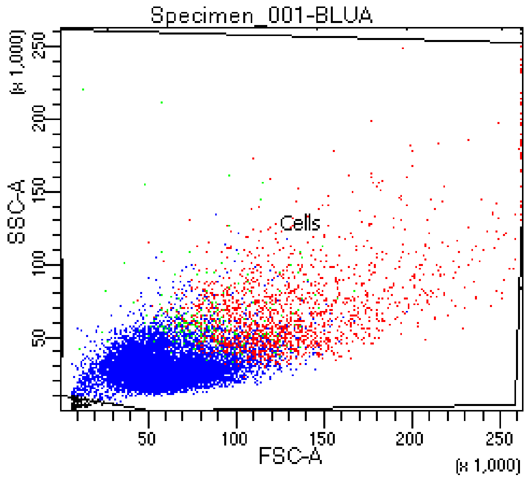

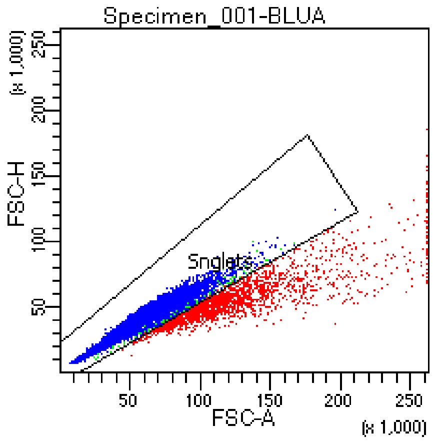

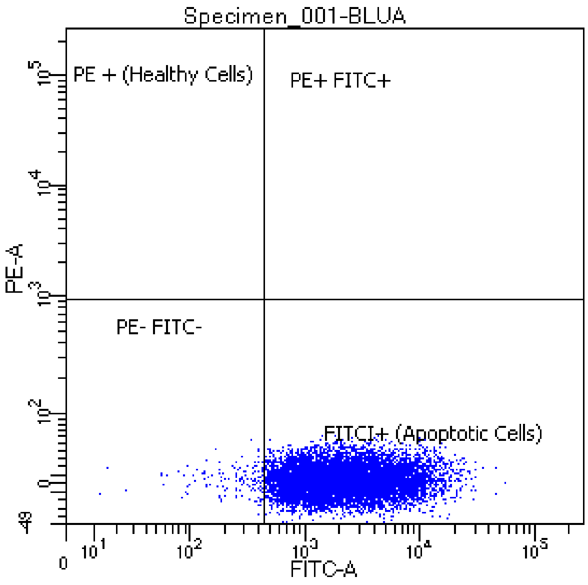

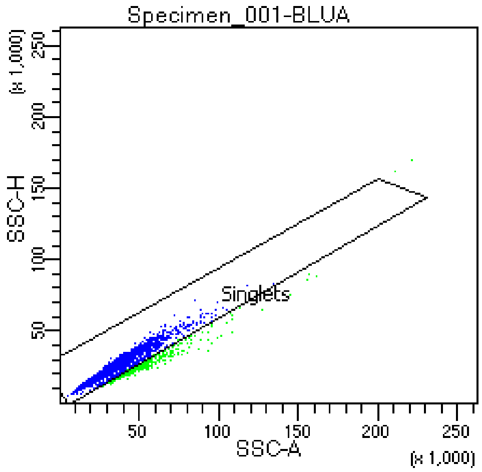

Tube: BLUA

Population	#Events	%Parent	%Total
All Events	12,411	####	100.0
Cells	12,256	98.8	98.8
Singlets	10,297	84.0	83.0
Singlets	9,985	97.0	80.5
PE + (Healthy Cells)	0	0.0	0.0
PE+ FITC+	0	0.0	0.0
PE- FITC-	164	1.6	1.3
FITCI+ (Apoptotic Cell	9,821	98.4	79.1

BD FACSDiva 9.0

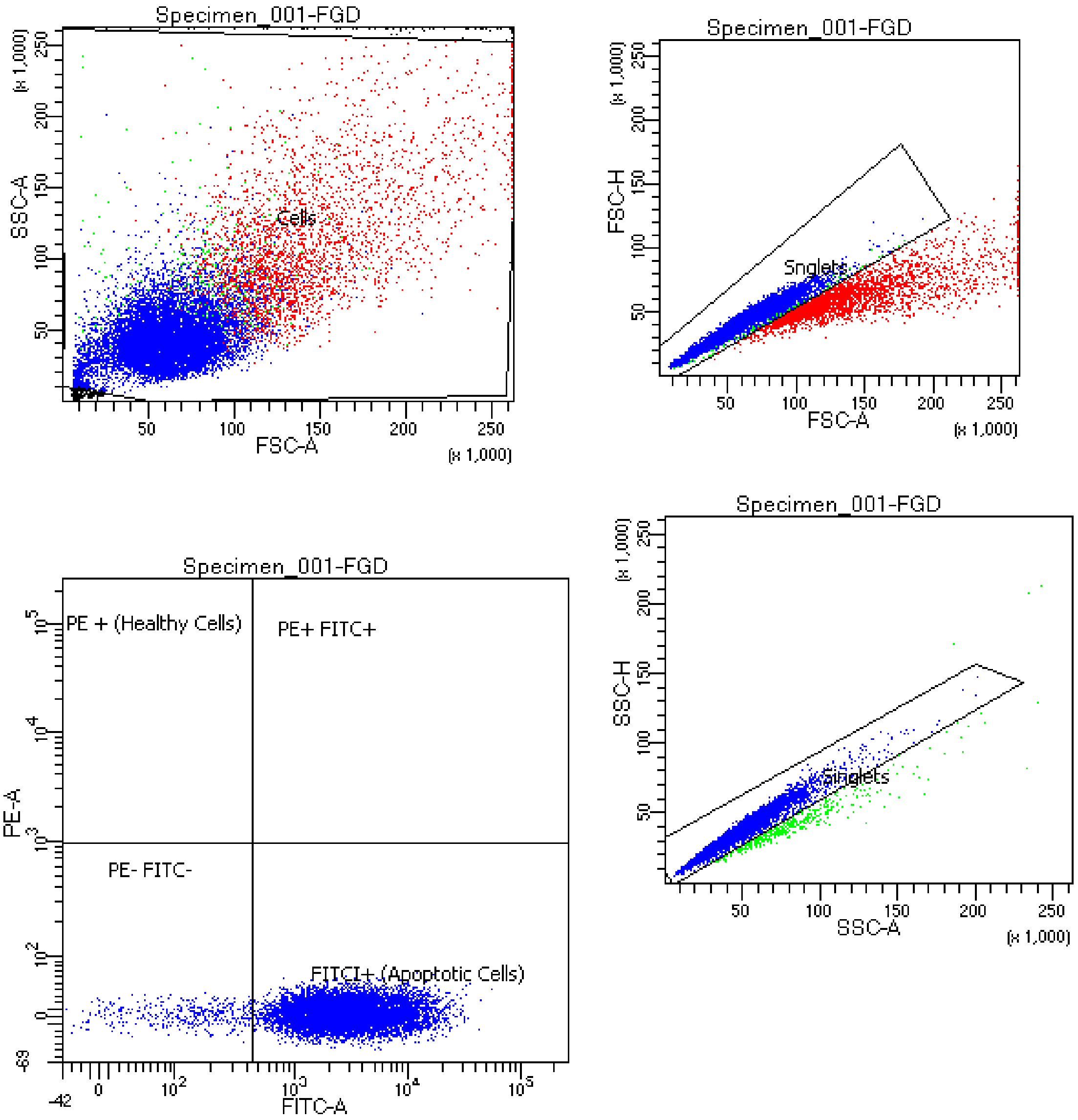

Tube: FGD

Population	#Events	%Parent	%Total
All Events	14,117	####	100.0
Cells	13,658	96.7	96.7
Snglets	10,473	76.7	74.2
Singlets	10,054	96.0	71.2
PE + (Healthy Cells)	0	0.0	0.0
PE+ FITC+	0	0.0	0.0
PE- FITC-	392	3.9	2.8
FITCI+ (Apoptotic Cell	9,662	96.1	68.4

BD FACSDiva 9.0

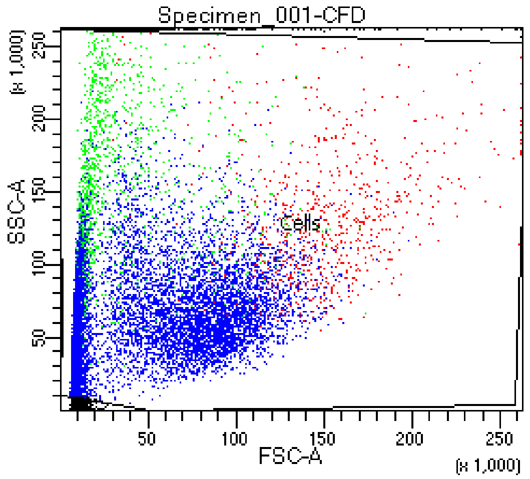

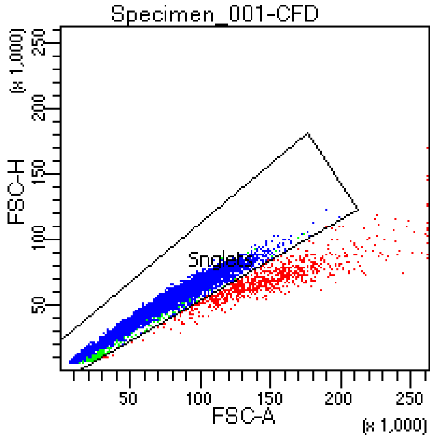

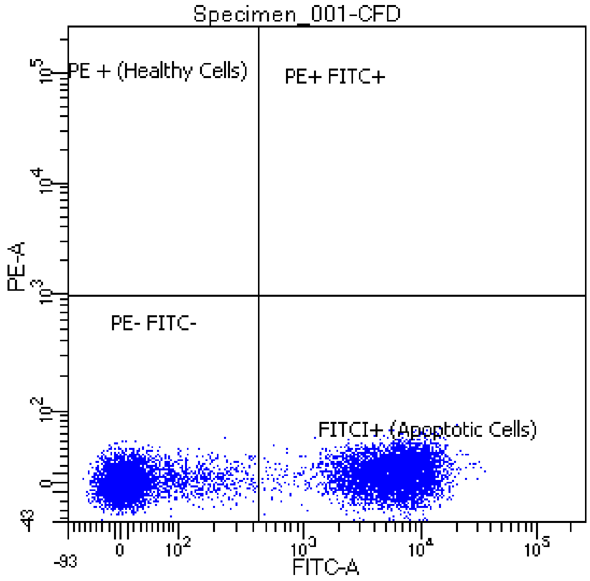

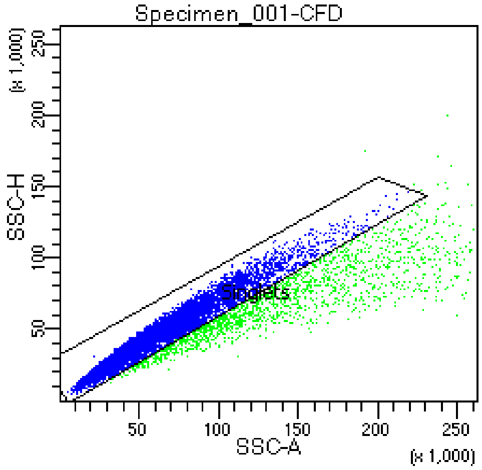

Tube: CFD

Population	#Events	%Parent	%Total
All Events	13,587	####	100.0
Cells	12,533	92.2	92.2
Snglets	11,741	93.7	86.4
Singlets	10,121	86.2	74.5
PE + (Healthy Cells)	0	0.0	0.0
PE+ FITC+	0	0.0	0.0
PE- FITC-	4,617	45.6	34.0
FITCI+ (Apoptotic Cell	5,504	54.4	40.5

BD FACSDiva 9.0

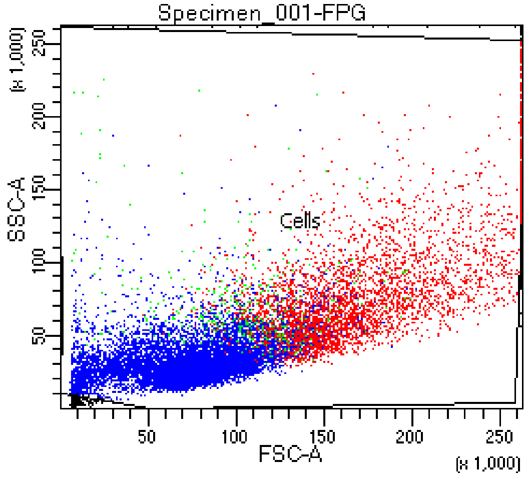

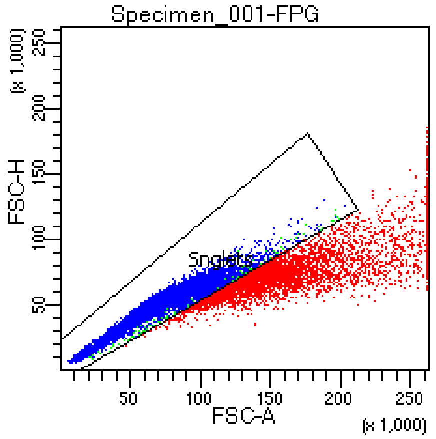

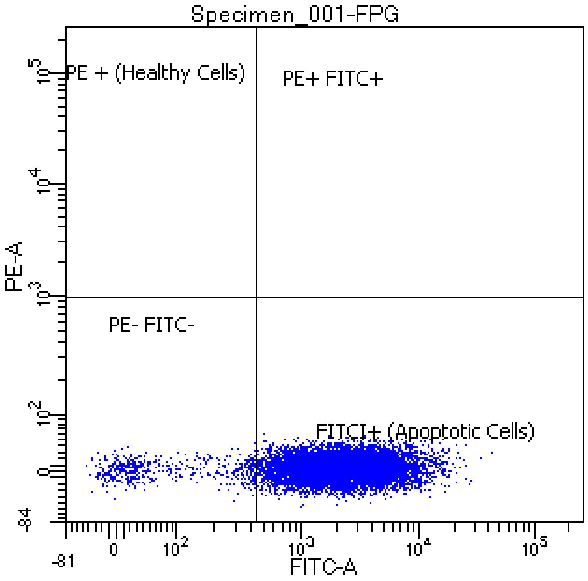

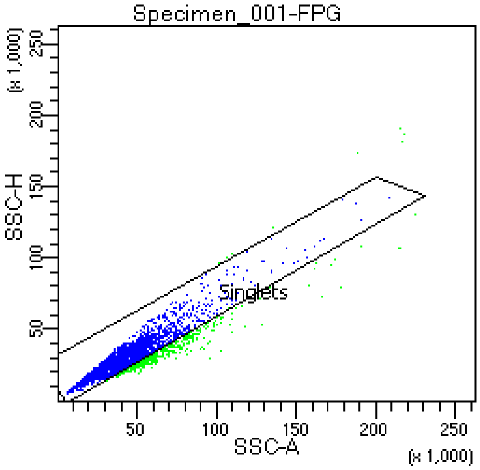

Tube: FPG

Population	#Events	%Parent	%Total
All Events	15,804	####	100.0
Cells	15,426	97.6	97.6
Snglets	10,584	68.6	67.0
Singlets	10,022	94.7	63.4
PE + (Healthy Cells)	0	0.0	0.0
PE+ FITC+	0	0.0	0.0
PE- FITC-	594	5.9	3.8
FITCI+ (Apoptotic Cell	9,428	94.1	59.7

BD FACSDiva 9.0

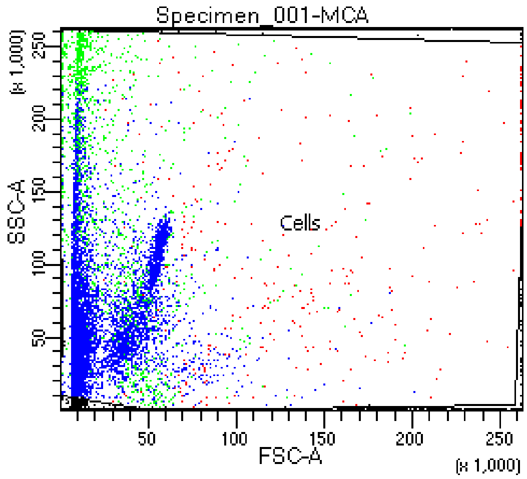

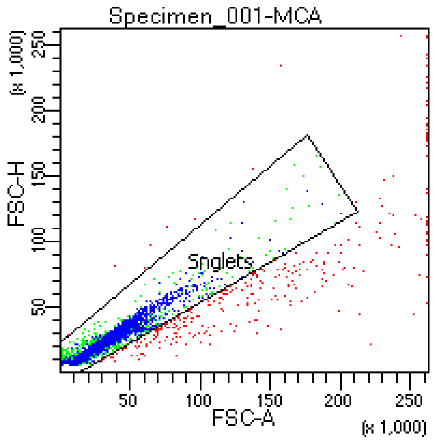

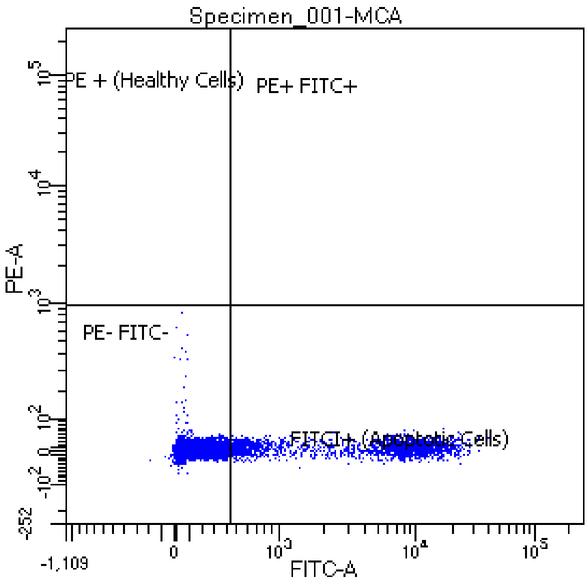

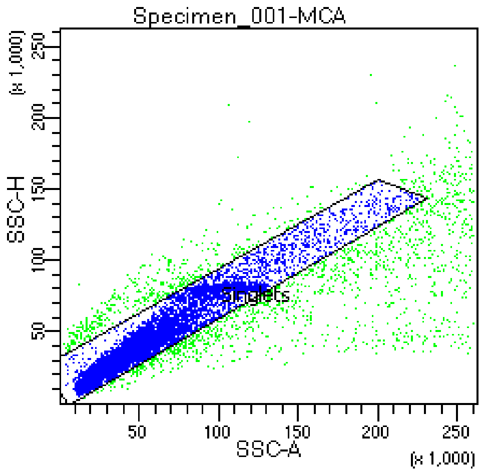

Tube: MCA

Population	#Events	%Parent	%Total
All Events	29,954	####	100.0
Cells	9,236	30.8	30.8
Snglets	8,878	96.1	29.6
Singlets	7,110	80.1	23.7
PE + (Healthy Cells)	0	0.0	0.0
PE+ FITC+	0	0.0	0.0
PE- FITC-	5,149	72.4	17.2
FITCI+ (Apoptotic Cell	1,961	27.6	6.5

BD FACSDiva 9.0

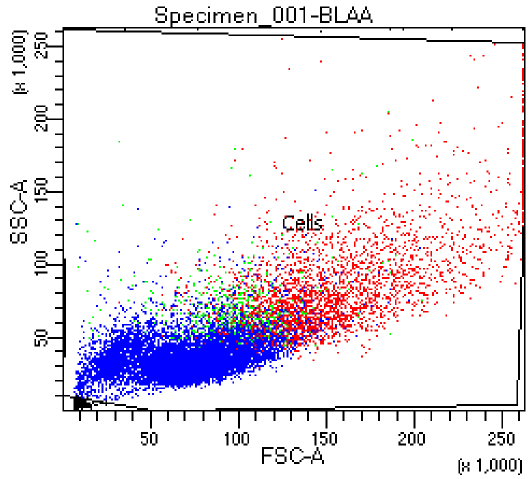

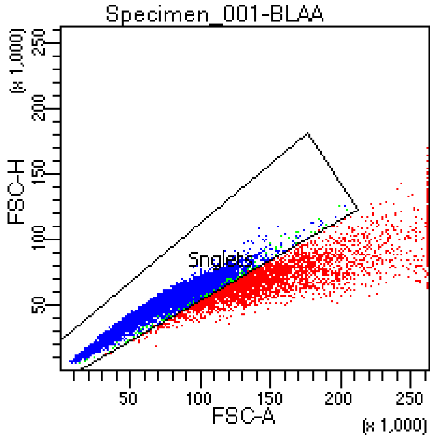

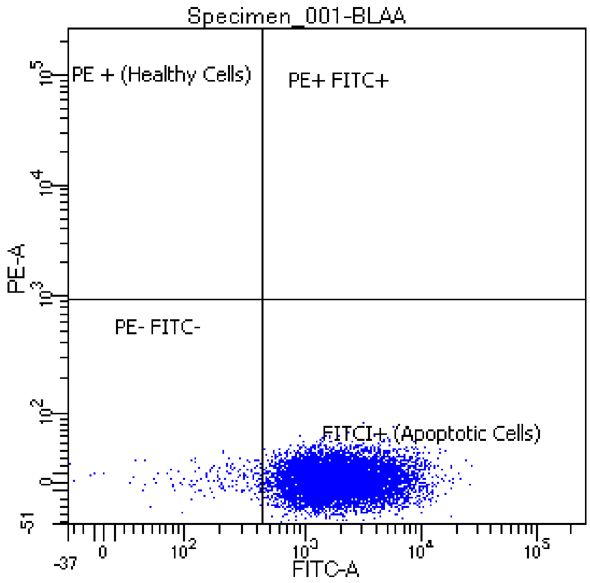

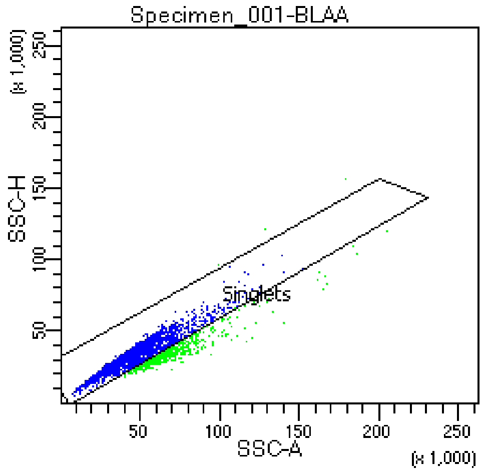

Tube: BLAA

Population	#Events	%Parent	%Total
All Events	14,314	####	100.0
Cells	14,094	98.5	98.5
Snglets	10,694	75.9	74.7
Singlets	10,036	93.8	70.1
PE + (Healthy Cells)	0	0.0	0.0
PE+ FITC+	0	0.0	0.0
PE- FITC-	178	1.8	1.2
FITCI+ (Apoptotic Cell	9,858	98.2	68.9

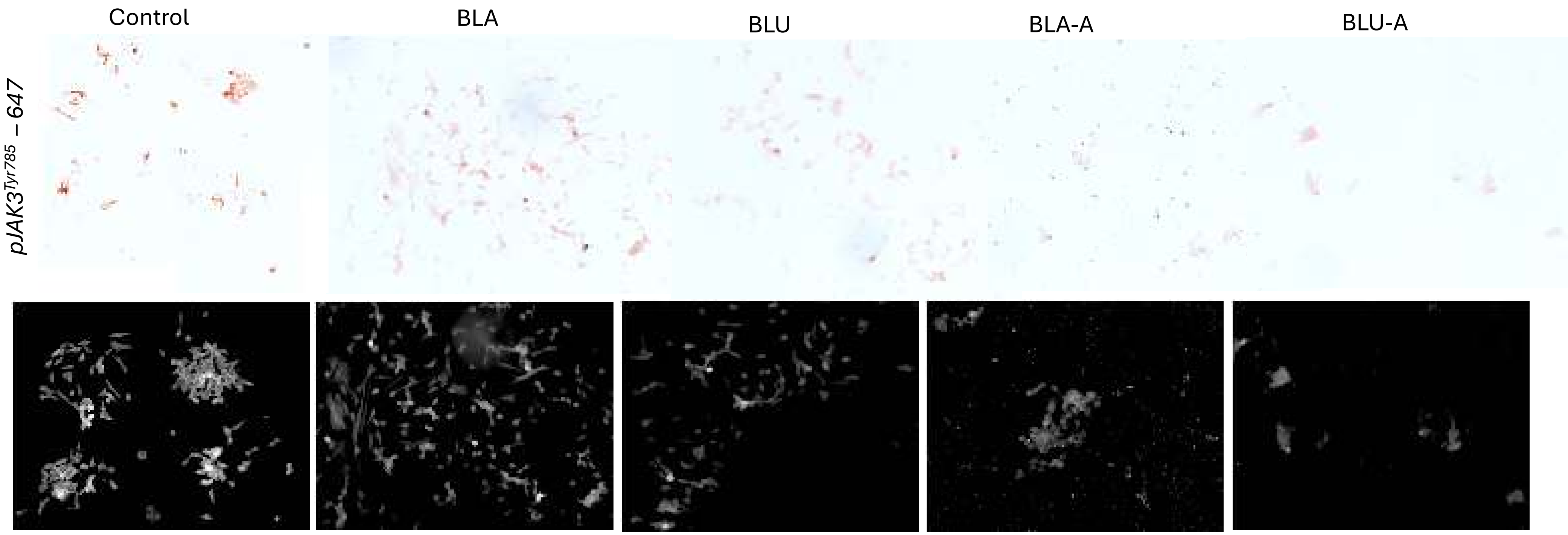
Control
BLA
BLU
BLA-A
BLU-A
pJAK3Tyr785 – 647

f

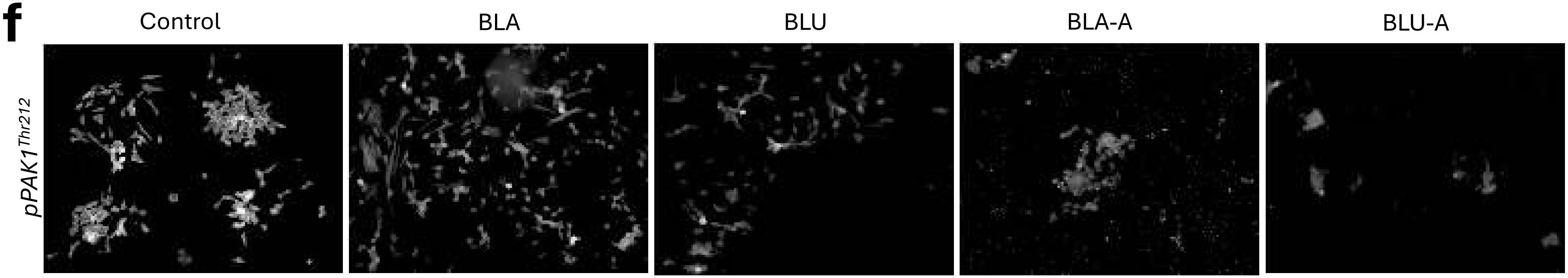
Control
BLA
BLU
BLA-A
BLU-A
pPAK1^{Thr212}

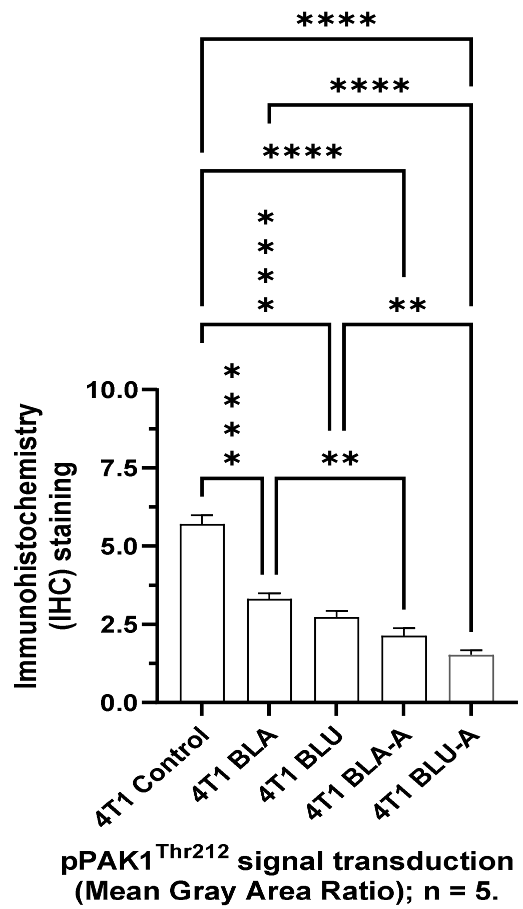
****
****
****
****
**
****
**
10.0
7.5
5.0
2.5
0.0
Immunohistochemistry (IHC) staining
4T1 Control
4T1 BLA
4T1 BLU
4T1 BLA-A
4T1 BLU-A
pPAK1^{Thr212} signal transduction
(Mean Gray Area Ratio); n = 5.

g

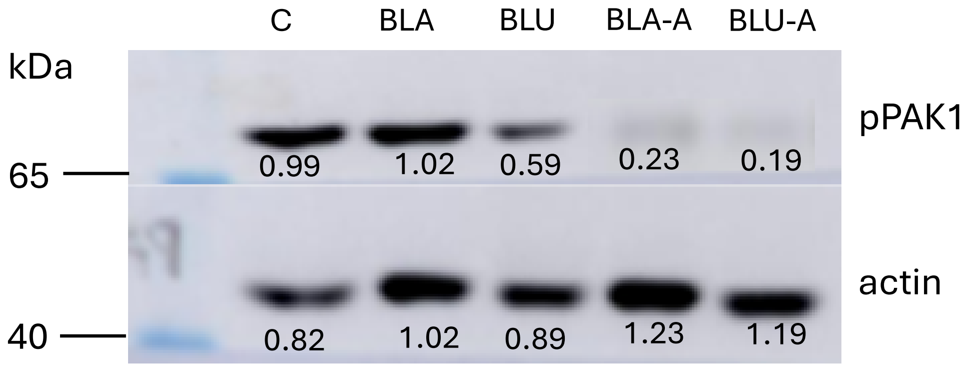
C
BLA
BLU
BLA-A
BLU-A
kDa
65
40
pPAK1
actin
0.99
1.02
0.59
0.23
0.19
0.82
1.02
0.89
1.23
1.19
