# Supplementary material for: Berry-derived gold nanoparticles induce integrated ROS-mediated apoptosis, immune modulation, and transcriptomic remodeling in 4T1 triple-negative cancer cells
Source: Cell Death Discov. 2026 Apr 10;12:225. doi: 10.1038/s41420-026-03023-z (PMC13184259; doi:10.1038/s41420-026-03023-z)
Supplement: Supplementary file 5 — Figure S5 [file 41420_2026_3023_MOESM5_ESM.pdf]

Condition Notes

NS-Control	
mTOR Control	DAPI and 647 colocalized, nucleus defined within cytoplasm
mTOR BLU	Bright signals, 647 much brighter and evident
mTOR BLU-A	Dimmer signals than BLU, nucleus and cytoplasm clearly defined

Link to the Images

https://buckeyemailosu.sharepoint.com/:f:/s/CMIFStaff2/EkOw1Pl-wZRAqYJlCxAHLy8B4xFrnm9ely7FWIveBPs0FQ?email=oladapo.fagbohun%40wilmington.edu&e=GGEh0q&xsdata=MDV8MDJ8b2xhZGFwby5mYWdib2h1bkB3aWxtaW5ndG9uLmVkdXw4ODk1OWM1NWUzMDM0YjFiMGRjYTA4ZGUxMzFiZTk4YXw4YjU3MTA3MjY0OTc0YmI1OTQxYTk2Yjg4OTBjNTg0NnwwfDB8NjM4OTY5MjA3ODY3NDYwOTMwfFVua25vd258VFdGcGJHWnNiM2Q4ZXlKRmJYQjBlVTFoY0draU9uUnlkV1VzSWxZaU9pSXdMakF1TURBd01DSXNJbEFpT2lKWGFXNHpNaUlzSWtGT0lqb2lUV0ZwYkNJc0lsZFVJam95ZlE9PXwwfHx8&sdata=OXJrZ2xSTEQ0MXg0alpBZ2JsVFVGOW1GNzBSd3JOcWxFb1FjajJodmxxYz0%3d

Bla AKT: Lots of cell aggregates within the slide
BLU AKT: Few cells in slide, very dim signal
BLA A AKT: Lost of cells around edge of slide cover, not many throughout the middle. Some areas on slide darker than others
HIGHEST BLU A AKT: Majority of cells around edge of cover slip. Varying 647 intensity between cells.

PI3K:
BLU-A (BRIGHTEST): Numerous cells, many aggreagtes
BLA-A: Numerous cells, aggregates present
BLA: Numerous cells, aggregates present
