# Supplementary material for: Berry-derived gold nanoparticles induce integrated ROS-mediated apoptosis, immune modulation, and transcriptomic remodeling in 4T1 triple-negative cancer cells
Source: Cell Death Discov. 2026 Apr 10;12:225. doi: 10.1038/s41420-026-03023-z (PMC13184259; doi:10.1038/s41420-026-03023-z)
Supplement: Supplementary file 6 — Supplementary methods [file 41420_2026_3023_MOESM6_ESM.pdf]

ERK 1/2 Polyclonal Antibody

Catalog Number:E-AB-31374 3 Publications

Elabscience®

Note: Centrifuge before opening to ensure complete recovery of vial contents.

Description

**Reactivity**	Human,Mouse,Rat
**Immunogen**	Synthesized peptide derived from the C-terminal region of human ERK 1/2
**Host**	Rabbit
**Isotype**	IgG
**Purification**	Affinity purification
**Conjugation**	Unconjugated
**Formulation**	PBS with 0.02% sodium azide, 0.5% protective protein and 50% glycerol, pH7.4

Applications	Recommended Dilution
**WB**	1:500-1:2000
**IHC**	1:100-1:300
**IF**	1:50-1:200
**ELISA**	1:10000

Data

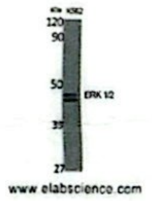

Western Blot analysis of K562 cells using ERK 1/2 Polyclonal Antibody at dilution of 1:2000.
Observed Mw:42,44kDa
Calculated Mw:43kDa

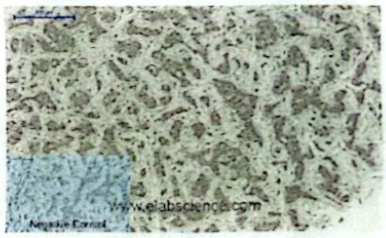

Immunohistochemistry of paraffin-embedded Human liver cancer tissue using ERK 1/2 Polyclonal Antibody at dilution of 1:200.

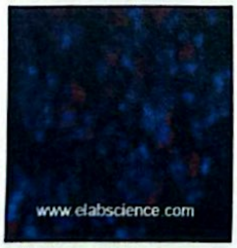

Immunofluorescence analysis of Rat lung tissue using ERK 1/2 Polyclonal Antibody at dilution of 1:200.

Preparation & Storage

**Storage**	Store at -20°C. Avoid freeze / thaw cycles.

Background

Involved in both the initiation and regulation of meiosis, mitosis, and postmitotic functions in differentiated cells by

TLR4

CD284 Polyclonal Antibody

Catalog Number:E-AB-30816

Elabscience®

Note: Centrifuge before opening to ensure complete recovery of vial contents.

Description

**Reactivity**	Human,Mouse
**Immunogen**	Synthesized peptide derived from the Internal region of human CD284.
**Host**	Rabbit
**Isotype**	IgG
**Purification**	Affinity purification
**Conjugation**	Unconjugated
**Formulation**	PBS with 0.02% sodium azide, 0.5% protective protein and 50% glycerol, pH7.4

Applications	Recommended Dilution
**WB**	1:500-1:2000
**IHC**	1:100-1:300
**IF**	1:50-1:200
**ELISA**	1:40000

Data

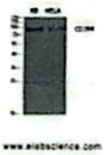

Western Blot analysis of KB, Hela cells using CD284 Polyclonal Antibody at dilution of 1:1000.
Observed Mw:95kDa
Calculated Mw:96kDa

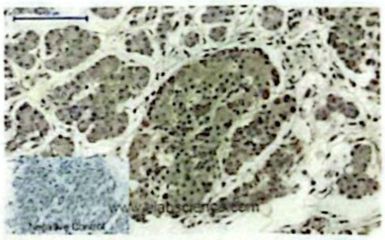

Immunohistochemistry of paraffin-embedded Human stomach cancer tissue using CD284 Polyclonal Antibody at dilution of 1:200.

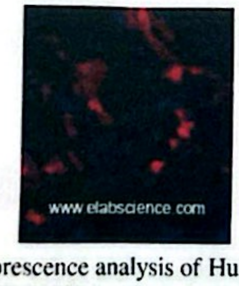

Immunofluorescence analysis of Human stomach tissue using CD284 Polyclonal Antibody at dilution of 1:200.

Preparation & Storage

**Storage**	Store at -20℃. Avoid freeze / thaw cycles.

Background

For Research Use Only

A Reliable Research Partner in Life Science and Medicine
Toll-free: 1-888-852-8623
Tel: 1-832-243-6086
Fax: 1-832-243-6017
Web: www.elabscience.com
Email: techsupport@elabscience.com

CD14 Polyclonal Antibody

Catalog Number:E-AB-70113

Note: *Centrifuge before opening to ensure complete recovery of vial contents.*

Description

**Reactivity**	Human;Mouse;Rat
**Immunogen**	KLH conjugated Synthetic peptide corresponding to Mouse CD14
**Host**	Rabbit
**Isotype**	IgG
**Purification**	Affinity purification
**Conjugation**	Unconjugated
**Buffer**	PBS with 0.02% sodium azide, 1% protective protein and 50% glycerol, pH7.4

Applications	Recommended Dilution
**WB**	1:500-1:2000
**IHC**	1:200-1:800

Data

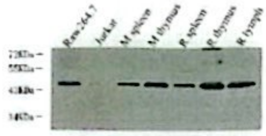

Western Blot analysis of various samples using CD14 Polyclonal Antibody at dilution of 1:1000.
Observed-MV:45kDa
Calculated-MV:40-55kDa

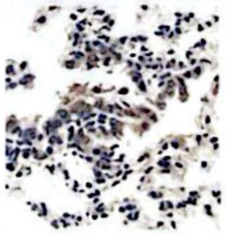

Immunohistochemistry analysis of paraffin-embedded mouse lung using CD14 Polyclonal Antibody at dilution of 1:400.

Preparation & Storage

**Storage**	Store at -20°C Valid for 12 months. Avoid freeze / thaw cycles.
**Shipping**	The product is shipped with ice pack,upon receipt,store it immediately at the temperature recommended.

Background

CD14 is a 50-55 kDa glycosylphosphatidylinositol-anchored glycoprotein. CD14 is preferentially expressed on monocytes and macrophages. CD14 acts as a co-receptor (along with TLR4 and MD-2) for bacterial liposaccharides (LPS). It plays a major role in the inflammatory response of monocytes to LPS.

For Research Use Only

Toll-free: 1-888-852-8623
Web: www.elabscience.com

Tel: 1-832-243-6086
Email: techsupport@elabscience.com

Fax: 1-832-243-6017

PI 3 kinase p85 alpha Monoclonal Antibody

Catalog Number:E-AB-22164 1 Publications

Elabscience®

Note: Centrifuge before opening to ensure complete recovery of vial contents.

Description

**Reactivity**	Mouse,Rat
**Immunogen**	Recombinant Protein
**Host**	Mouse
**Isotype**	IgG
**Purification**	Protein A purification
**Formulation**	PBS with 0.02% sodium azide, 0.5% protective protein and 50% glycerol, pH7.4

Applications	Recommended Dilution
**WB**	1:1000-2000
**IHC**	1:100-200

Data

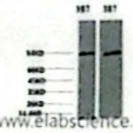

Western Blot analysis of 1) 3T3, 2) Rat liver using PI 3 kinase p85 alpha Monoclonal Antibody at dilution of 1:2000.
Observed Mw:85kDa

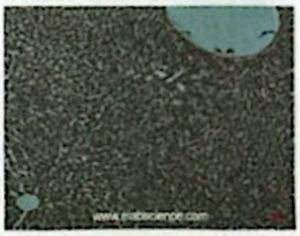

Immunohistochemistry of paraffin-embedded Rat liver tissue using PI 3 kinase p85 alpha Monoclonal Antibody at dilution of 1:200.

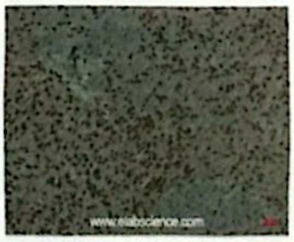

Immunohistochemistry of paraffin-embedded Mouse brain tissue using PI 3 kinase p85 alpha Monoclonal Antibody at dilution of 1:200.

Preparation & Storage

**Storage**	Store at -20°C. Avoid freeze / thaw cycles.

Background

Binds to activated (phosphorylated) protein-Tyr kinases, through its SH2 domain, and acts as an adapter, mediating the association of the p110 catalytic unit to the plasma membrane. Necessary for the insulin-stimulated increase in glucose uptake and glycogen synthesis in insulin-sensitive tissues.

For Research Use Only

A Reliable Research Partner in Life Science and Medicine

Toll-free: 1-888-852-8623 Tel: 1-832-243-6086 Fax: 1-832-243-6017

Web: www.elabscience.com Email: techsupport@elabscience.com

Elabscience®

Elabscience Bionovation Inc.
A Reliable Research Partner in Life Science and Medicine

MYD88 Polyclonal Antibody

Catalog Number:D-AB-10290L

Note: *Centrifuge before opening to ensure complete recovery of vial contents.*

Description

**Reactivity**	Human;Mouse;Rat
**Immunogen**	Recombinant Human MYD88 Protein expressed by E.coli
**Host**	Rabbit
**Isotype**	IgG
**Purification**	Antigen Affinity Purification
**Conjugation**	Unconjugated
**Buffer**	PBS with 0.02% sodium azide,1% protective protein and 50% glycerol,pH7.4

Applications	Recommended Dilution
**WB**	1:500-1:1000

Data

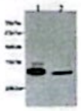

Western blot with MYD88 Polyclonal antibody at dilution of 1:1000.lane 1:Raw264.7 whole cell lysate,lane 2:Mouse lung,lane 3:Rat lung
Observed-MV:33 kDa
Calculated-MV:33 kDa

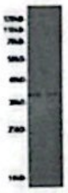

Western blot with MYD88 Polyclonal antibody at dilution of 1:600.lane 1:HepG2 whole cell lysate,lane 2:K562 whole cell lysate
Observed-MV:33 kDa
Calculated-MV:33 kDa

Preparation & Storage

**Storage**	Store at -20°C Valid for 12 months. Avoid freeze / thaw cycles.
**Shipping**	The product is shipped with ice pack,upon receipt,store it immediately at the temperature recommended.

Background

Adapter protein involved in the Toll-like receptor and IL-1 receptor signaling pathway in the innate immune response. Acts via IRAK1,IRAK2,IRF7 and TRAF6,leading to NF-kappa-B activation,cytokine secretion and the inflammatory response. Increases IL-8 transcription. Involved in IL-18-mediated signaling pathway.

For Research Use Only

Toll-free: 1-888-852-8623
Web: www.elabscience.com
Tel: 1-832-243-6086
Email: techsupport@elabscience.com
Fax: 1-832-243-6017

NFκB-p105/p50 Polyclonal Antibody

Catalog Number:E-AB-32226 2 Publications

Elabscience®

Note: Centrifuge before opening to ensure complete recovery of vial contents.

Description

**Reactivity**	Human,Mouse
**Immunogen**	Synthesized peptide derived from human NFκB-p105/p50 around the non-phosphorylation site of Ser337.
**Host**	Rabbit
**Isotype**	IgG
**Purification**	Affinity purification
**Conjugation**	Unconjugated
**Formulation**	PBS with 0.02% sodium azide, 0.5% protective protein and 50% glycerol, pH7.4

Applications	Recommended Dilution
**WB**	1:500-1:2000
**IHC**	1:100-1:300
**IF**	1:200-1:1000
**ELISA**	1:40000

Data

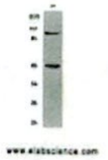

Western Blot analysis of 293T cells using NFκB-p105/p50 Polyclonal Antibody at dilution of 1:500.
Observed Mw:50,110kDa
Calculated Mw:105kDa

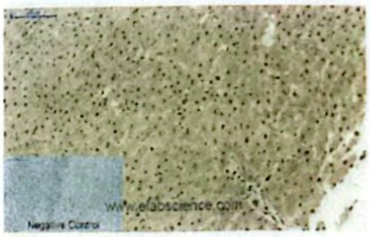

Immunohistochemistry of paraffin-embedded Human liver tissue using NFκB-p105/p50 Polyclonal Antibody at dilution of 1:200.

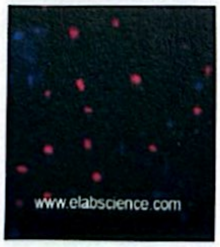

Immunofluorescence analysis of Rat heart tissue using NFκB-p105/p50 Polyclonal Antibody at dilution of 1:200.

Preparation & Storage

**Storage**	Store at -20°C. Avoid freeze / thaw cycles.

For Research Use Only

A Reliable Research Partner in Life Science and Medicine

Toll-free: 1-888-852-8623 Tel: 1-832-243-6086 Fax: 1-832-243-6017

Web: www.elabscience.com Email: techsupport@elabscience.com

JAK3 Polyclonal Antibody

Catalog Number:E-AB-31846

Elabscience®

Note: Centrifuge before opening to ensure complete recovery of vial contents.

Description

**Reactivity**	Human,Mouse,Rat
**Immunogen**	Synthesized peptide derived from human JAK3 around the non-phosphorylation site of Tyr785.
**Host**	Rabbit
**Isotype**	IgG
**Purification**	Affinity purification
**Conjugation**	Unconjugated
**Formulation**	PBS with 0.02% sodium azide, 0.5% protective protein and 50% glycerol, pH7.4

Applications	Recommended Dilution
**WB**	1:500-1:2000
**IHC**	1:100-1:300
**IF**	1:200-1:1000
**ELISA**	1:20000

Data

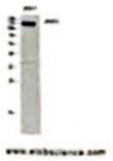

Western Blot analysis of 293T cells using JAK3 Polyclonal Antibody at dilution of 1:1000.
Observed Mw:125kDa
Calculated Mw:125kDa

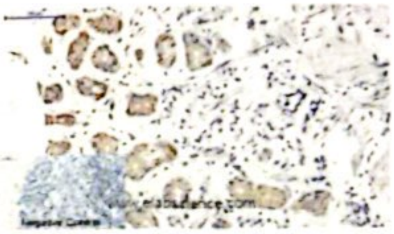

Immunohistochemistry of paraffin-embedded Human stomach tissue using JAK3 Polyclonal Antibody at dilution of 1:200.

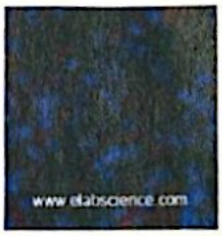

Immunofluorescence analysis of Rat kidney tissue using JAK3 Polyclonal Antibody at dilution of 1:200.

Preparation & Storage

**Storage**	Store at -20°C. Avoid freeze / thaw cycles.

Background

For Research Use Only

A Reliable Research Partner in Life Science and Medicine
Toll-free: 1-888-852-8623 Tel: 1-832-243-6086 Fax: 1-832-243-6017
Web: www.elabscience.com Email: techsupport@elabscience.com

Elabscience®

Elabscience Bionovation Inc.
A Reliable Research Partner in Life Science and Medicine

Phospho-AKT1 (Thr450) Polyclonal Antibody

Catalog Number:E-AB-20804

Note: *Centrifuge before opening to ensure complete recovery of vial contents.*

Description

**Reactivity**	Human;Mouse;Rat
**Immunogen**	Synthesized peptide derived from human Akt1 around the phosphorylation site of Thr450
**Host**	Rabbit
**Isotype**	IgG
**Purification**	Affinity purification
**Conjugation**	Unconjugated
**Buffer**	PBS with 0.02% sodium azide, 0.5% protective protein and 50% glycerol, pH7.4

Applications	Recommended Dilution
**WB**	1:500-1:2000
**IHC**	1:100-1:300

Data

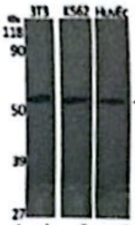

Western Blot analysis of various cells with Phospho-Akt1 (Thr450) Polyclonal Antibody at dilution of 1:1000
Observed-MV:55 kDa
Calculated-MV:56 kDa

Preparation & Storage

**Storage**	Store at -20°C Valid for 12 months. Avoid freeze / thaw cycles.
**Shipping**	The product is shipped with ice pack,upon receipt,store it immediately at the temperature recommended.

Background

The serine-threonine protein kinase AKT1 is catalytically inactive in serum-starved primary and immortalized fibroblasts. AKT1 and the related AKT2 are activated by platelet-derived growth factor. The activation is rapid and specific, and it is abrogated by mutations in the pleckstrin homology domain of AKT1. It was shown that the activation occurs through phosphatidylinositol 3-kinase. In the developing nervous system AKT is a critical mediator of growth factor-induced neuronal survival. Survival factors can suppress apoptosis in a transcription-independent manner by activating the serine/threonine kinase AKT1, which then phosphorylates and inactivates components of the apoptotic machinery.

For Research Use Only

Toll-free: 1-888-852-8623
Web: www.elabscience.com
Tel: 1-832-243-6086
Email: techsupport@elabscience.com
Fax: 1-832-243-6017

No	Gene	Forward	Reverse	Accession No.	References
1	GAPDH	GGGAAGCCCATCACCATCTT	GCCTTCTCCATGGTGGTGAA	NM_008084.3	(Cho et al., 2011; Mueller-Steiner et al., 2006)
2	ACTIN	CTCTGGCTCCTAGCACCATGAAGA	GTAAAACGCAGCTCAGTAACAGTCCG	NM_007393.5	(Fairfield et al., 2021)
3	NF-κB	AAGAACAGAGACCGCTGGTG	CAGGTTCTGCATCCCCTCTG	XM_006509023.5	(Fagbohun et al., 2024; Wang et al., 2022)
4	IL-6	CTGCAAGAGACTTCCATCCAG	AGTGGTATAGACAGGTCTGTTGG	NM_031168	(Li et al., 2015)
5	TNF-α	CAGGCGGTGCCTATGTCTC	CGATCACCCCGAAGTTCAGTAG	NM_013693	(Li et al., 2015)
6	TLR4	CGCTCTGGCATCATCTTCAT	GTTGCCGTTTCTTGTTCTTCC	NM_021297.3	(Monmai et al., 2020)
7	iNOS	TTCCAGAATCCCTGGACAAG	TGGTCAAACTCTTGGGGTTC	BC062378.1	(Monmai et al., 2020)
8	JNK1 (MAPK8)	CTTCAGAAGCAGAAGCCCCA	TGTGCTAAAGGAGACGGCTG	NM_016700.4	(Dai et al., 2022)
9	ERK1 (MAPK3)	ACACTGGCTTTCTGACGGAG	TGATGCGCTTGTTTGGGTTG	NM_011952.2	(Nam et al., 2024)
10	ERK2 (MAPK1)	AATTGGTCAGGACAAGGGCTC	GAGTGGGTAAGCTGAGACGG	NM_011949.3	(Nam et al., 2024)
11	Jun (AP-1)	ATGACTGCAAAGATGGAAACGACCTTCTAC	TCAAAACGTTTGCAACTGCTGCGTTAG	NM_010591.2	(Hatori et al., 2023)
12	FOS (AP-1)	ATGATGTTCTCGGGTTTCAACGCCGACTAC	TTCTCTGACTGCTCACAGGGCCAGCA	NM_010234.2	(Hatori et al., 2023)
13	IL-1β	GGGCCTCAAAGGAAAGAATC	TACCAGTTGGGGAACTCTGC	NM_008361.4	(Monmai et al., 2020)
14	NRF2	TCTGACTCCGGCATTTCACT	GGCACTGTCTAGCTCTTCCA	NM_031789	(Fan et al., 2017)
15	mTOR	CGCTCACTGCTGTGCTCTAT	GTAGCGGATATCAGGGTCAGG	NG_033239.1	(Nam et al., 2024)
16	AKT	CTGAGATTGTGTCAGCCCTG	CACAGCCCGAAGTCTGTGATCTTA	NG_012188.1	(Al-Rawashde et al., 2022)
17	PI3K	TTAGCTATTCCCACGCAGGA	CACAATAGTGTCTGTGACTC	NG_012113.2	(Al-Rawashde et al., 2022)
18	PCSK9	ATCACCGACTTCAACAGCGT	GCCCTTCCCTTGACAGTTGA	NM_153565.2	(Han et al., 2022)
19	CAMKII α	AGGCTTCAATTCCCAGCTCT	TGTGTCAGCCAATGAAAGGC		(Sim et al., 2020)
20	AMPKα1	TATTGCTGCCATTAGGCTAC	GACCTGACAGAATAGGATATGCCCAACCTC		(MacMillan & Evans, 2023)
21	m-GLUT4	AAAAGTGCCTGAAACCAGAG	TCACCTCCTGCTCTAAAAGG		(Atkinson et al., 2013)
22	KEAP1	TTCGCCTACACGGCCTC	GAAGTTGGCGATGCCGATG	NM_057152	(Fan et al., 2017)
23	NQO1	AGCCCAGATATTGTGGCCG	CCTTTCAGAATGGCTGGCAC	NM_000903	(Xiong et al., 2015)
24	IFN-γ	CTCAAGTGGCATAGATGT	GAGATAATCTGGCTCTGCAGGATT	NM_008337.3	(Monmai et al., 2020)
25	LDLR	TTGGGTTGATTCCAAACTCC	GATTGGCACTGAAAATGGCT	NM_010700	(Li et al., 2020)

26					
27					
28					
29					
30	LXRα	GCAGGACCAGCTCCAAGTAG	ATTAGCATCCGTGGGAACAT	NM_013839	
31	LXRβ	CTTCCCCCACAAGTTCTCTG	GGCTCATCCTCTGGCTCTAA	NM_009473	
32	SREBP1	GCACTTTTTGACACGTTTCTTC	CTGTACAGGCTCTCCTGTGG	NM_001244003	
33	SREBP2	GAGAGCTGTGAATTTTCCAGTG	CTACAGATGATATCCGGACCAA	NM_001244004	
34					
35					

Al-Rawashde, F. A., Al-Wajeeh, A. S., Vishkaei, M. N., Saad, H. K. M., Johan, M. F., Taib, W. R. W., Ismail, I., & Al-Jamal, H. A. N. (2022). Thymoquinone Inhibits JAK/STAT and PI3K/Akt/ mTOR Signaling Pathways in MV4-11 and K562 Myeloid Leukemia Cells. *Pharmaceuticals (Basel)*, *15*(9). https://doi.org/10.3390/ph15091123

Atkinson, B. J., Griesel, B. A., King, C. D., Josey, M. A., & Olson, A. L. (2013). Moderate GLUT4 overexpression improves insulin sensitivity and fasting triglyceridemia in high-fat diet-fed transgenic mice. *Diabetes*, *62*(7), 2249-2258. https://doi.org/10.2337/db12-1146

Cho, S. H., Sun, B., Zhou, Y., Kauppinen, T. M., Halabisky, B., Wes, P., Ransohoff, R. M., & Gan, L. (2011). CX3CR1 protein signaling modulates microglial activation and protects against plaque-independent cognitive deficits in a mouse model of Alzheimer disease. *J Biol Chem*, *286*(37), 32713-32722. https://doi.org/10.1074/jbc.M111.254268

Dai, X.-Y., Zhu, S.-Y., Chen, J., Li, M.-Z., Talukder, M., & Li, J.-L. (2022). Role of Toll-like Receptor/MyD88 Signaling in Lycopene Alleviated Di-2-ethylhexyl Phthalate (DEHP)-Induced Inflammatory Response. *Journal of Agricultural and Food Chemistry*, *70*(32), 10022-10030. https://doi.org/10.1021/acs.jafc.2c03864

Fagbohun, O. F., Thilakarathna, W. P. D. W., Zhou, J., Lehmann, C., Jiao, G., & Rupasinghe, H. P. V. (2024). Sea Cucumber and Blueberry Extracts Suppress Inflammation and Reduce Acute Lung Injury through the Regulation of NF-κB/MAPK/JNK Signaling Pathway in Lipopolysaccharide-Treated C57BL/6 Mice. *Molecules*, *29*(7), 1511. https://www.mdpi.com/1420-3049/29/7/1511

Fairfield, H., Dudakovic, A., Khatib, C. M., Farrell, M., Costa, S., Falank, C., Hinge, M., Murphy, C. S., DeMambro, V., Pettitt, J. A., Lary, C. W., Driscoll, H. E., McDonald, M. M., Kassem, M., Rosen, C., Andersen, T. L., van Wijnen, A. J., Jafari, A., & Reagan, M. R. (2021). Myeloma-Modified Adipocytes Exhibit Metabolic Dysfunction and a Senescence-Associated Secretory Phenotype. *Cancer Research*, *81*(3), 634-647. https://doi.org/10.1158/0008-5472.Can-20-1088

Fan, Z., Wirth, A. K., Chen, D., Wruck, C. J., Rauh, M., Buchfelder, M., & Savaskan, N. (2017). Nrf2-Keap1 pathway promotes cell proliferation and diminishes ferroptosis. *Oncogenesis*, *6*(8), e371. https://doi.org/10.1038/oncsis.2017.65

Han, H., Wang, M., Zhong, R., Yi, B., Schroyen, M., & Zhang, H. (2022). Depletion of Gut Microbiota Inhibits Hepatic Lipid Accumulation in High-Fat Diet-Fed Mice. *International Journal of Molecular Sciences*, *23*(16), 9350. https://www.mdpi.com/1422-0067/23/16/9350

Hatori, T., Maeda, T., Suzuki, A., Takahashi, K., & Kato, Y. (2023). SPARC is a decoy counterpart for c-Fos and is associated with osteoblastic differentiation of bone marrow stromal cells by inhibiting adipogenesis. *Mol Med Rep*, *27*(2). https://doi.org/10.3892/mmr.2023.12937

Li, H. H., Li, J., Zhang, X. J., Li, J. M., Xi, C., Wang, W. Q., Lu, Y. L., & Xuan, L. J. (2020). 23,24-Dihydrocucurbitacin B promotes lipid clearance by dual transcriptional regulation of LDLR and PCSK9. *Acta Pharmacol Sin*, *41*(3), 327-335. https://doi.org/10.1038/s41401-019-0274-0

Li, J., Xia, Y., Liu, T., Wang, J., Dai, W., Wang, F., Zheng, Y., Chen, K., Li, S., Abudumijiti, H., Zhou, Z., Wang, J., Lu, W., Zhu, R., Yang, J., Zhang, H., Yin, Q., Wang, C., Zhou, Y., . . . Guo, C. (2015). Protective Effects of Astaxanthin on ConA-Induced Autoimmune Hepatitis by the JNK/p-JNK Pathway-Mediated Inhibition of Autophagy and Apoptosis. *PLoS One*, *10*(3), e0120440. https://doi.org/10.1371/journal.pone.0120440

MacMillan, S., & Evans, A. M. (2023). AMPK facilitates the hypoxic ventilatory response through non-adrenergic mechanisms at the brainstem. *Pflugers Arch*, *475*(1), 89-99. https://doi.org/10.1007/s00424-022-02713-8

Monmai, C., Rod-In, W., Jang, A. Y., Lee, S. M., Jung, S. K., You, S., & Park, W. J. (2020). Immune-enhancing effects of anionic macromolecules extracted from Codium fragile coupled with arachidonic acid in RAW264.7 cells. *PLoS One*, *15*(10), e0239422. https://doi.org/10.1371/journal.pone.0239422

Mueller-Steiner, S., Zhou, Y., Arai, H., Roberson, E. D., Sun, B., Chen, J., Wang, X., Yu, G., Esposito, L., Mucke, L., & Gan, L. (2006). Antiamyloidogenic and Neuroprotective Functions of Cathepsin B: Implications for Alzheimer's Disease. *Neuron*, *51*(6), 703-714. https://doi.org/https://doi.org/10.1016/j.neuron.2006.07.027

Nam, D. E., Park, S. J., Omole, S., Um, E., Hakami, R. M., & Hahn, Y. S. (2024). Activated Gab1 drives hepatocyte proliferation and anti-apoptosis in liver fibrosis via potential involvement of the HGF/c-Met signaling axis. *PLoS One*, *19*(6), e0306345. https://doi.org/10.1371/journal.pone.0306345

Sim, K. M., Lee, Y. S., Kim, H. J., Cho, C. H., Yi, G. S., Park, M. J., Hwang, E. M., & Park, J. Y. (2020). Suppression of CaMKIIβ Inhibits ANO1-Mediated Glioblastoma Progression. *Cells*, *9*(5). https://doi.org/10.3390/cells9051079

Wang, C., Fan, L., Khawaja, R. R., Liu, B., Zhan, L., Kodama, L., Chin, M., Li, Y., Le, D., Zhou, Y., Condello, C., Grinberg, L. T., Seeley, W. W., Miller, B. L., Mok, S. A., Gestwicki, J. E., Cuervo, A. M., Luo, W., & Gan, L. (2022). Microglial NF-κB drives tau spreading and toxicity in a mouse model of tauopathy. *Nat Commun*, *13*(1), 1969. https://doi.org/10.1038/s41467-022-29552-6

Xiong, L., Xie, J., Song, C., Liu, J., Zheng, J., Liu, C., Zhang, X., Li, P., & Wang, F. (2015). The Activation of Nrf2 and Its Downstream Regulated Genes Mediates the Antioxidative Activities of Xueshuan Xinmaining Tablet in Human Umbilical Vein Endothelial Cells. *Evid Based Complement Alternat Med*, *2015*, 187265. https://doi.org/10.1155/2015/187265

PCR PROTOCOL FROM IDT

Thank you for the final reaction concentration needed for your primers. I've provided guidance on how to resuspend your primers to a 20X mix of forward and reverse primer below.

Unless you requested your oligos be shipped to you already resuspended, your oligos will arrive dried and will need to be resuspended.

1) Resuspend your dried primers from IDT to 100 uM using the volume listed on the spec sheet. This will be the delivered nmol quantity x 10 in uL. For example, if you received 18.9 nmol, you'd resuspend in 189 uL to get to 100 uM.
2) Make a 20X working stock using:
-10 uL of 100 uM forward primer
-10 uL of 100 uM reverse primer
-80 uL of nuclease-free water or buffer

You may then use 1 uL of this 20X stock of your mixed primers in a 20 uL reaction.

20 uL Reaction Mix
10 uL Master Mix
1 uL of the 20X primer stock created above (contains both forward and reverse primers together)
1-2 uL of template
7-8 uL of nuclease-free water

Regarding your freeze-thaw concerns, IDT has found that our oligos are stable up to 30 freeze/thaw cycles. However, if you'd like to minimize the number of freeze/thaws your assays undergo, you may aliquot your 100 uM primers as well as your 100 uL 20X mix into smaller aliquots.
