# Supplementary material for: Berry-derived gold nanoparticles induce integrated ROS-mediated apoptosis, immune modulation, and transcriptomic remodeling in 4T1 triple-negative cancer cells
Source: Cell Death Discov. 2026 Apr 10;12:225. doi: 10.1038/s41420-026-03023-z (PMC13184259; doi:10.1038/s41420-026-03023-z)
Supplement: Supplementary file 7 — Uncropped western blot [file 41420_2026_3023_MOESM7_ESM.pdf]

Western blotting Protein lane

07/21-07/28/2025
Frank G

Protein molecular weigh

	150			
pJAK3				
	100			
TLR4			pPI3K	STAT3
	75			
			pPAK1	
	50			
Actin			pERK	
	37			
	25			

Western blotting results

07/21-07/28/2025
Frank G

P-ERK
P-PAK1
TLR4
P-JAK3

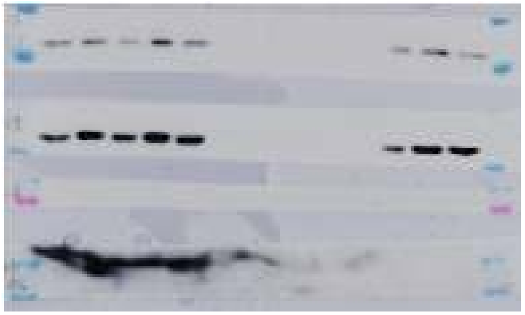

1 2 3 4 5 6 7 8 9 10 11 12 13

07232025\07232025-1 2025.07.23_15.09.10_Ch

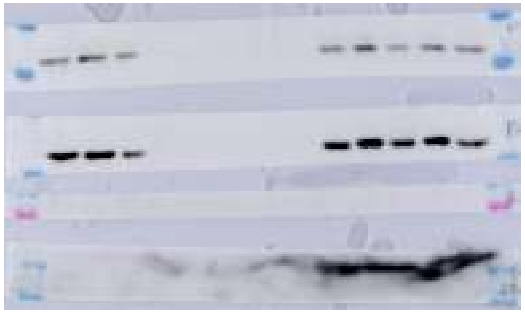

NO FLIPPING expose 18 mins
07232025\07232025-4 2025.07.23_15.18.41_Ch

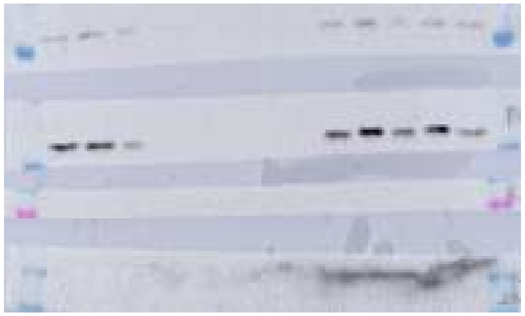

NO FLIPPING expose 25 s
2025.07.23_15.16.45_Ch

Stripping membrane1 **Stat3**

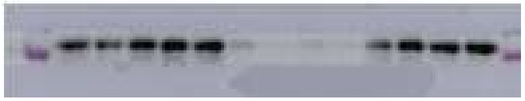

1 2 3 4 5 6 7 8 9 10 11 12 13

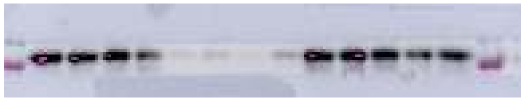

NO FLIPPING
2025.07.25_13.43.06_Ch

Stripping membrane2

P-PI3K
b-actin

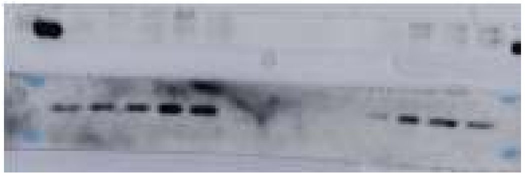

1 2 3 4 5 6 7 8 9 10 11 12 13

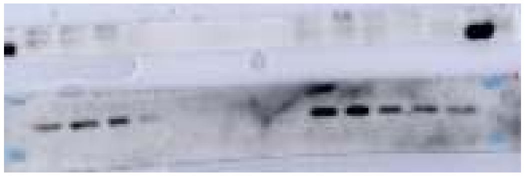

NO FLIPPING
2025.07.28_15.12.52_Ch

Lane 1--- Lane 13: C, BLA, BLU, BLA-A, BLU-A, R2P, R4P, T2P, T4P, CFO, CFOPG, FGD, FGDPG

Precision Plus Protein Dual Color Standards, 500 µl #1610374: Precision Plus Protein Dual Color Standards, 500 µl #1610374 | Bio-Rad

Western blotting results for 13 cell samples to detect 7 antibodies (b-actin +6 antibody)
